# Rosiglitazone and AS601245 Decrease Cell Adhesion and Migration through Modulation of Specific Gene Expression in Human Colon Cancer Cells

**DOI:** 10.1371/journal.pone.0040149

**Published:** 2012-06-28

**Authors:** Angelo Cerbone, Cristina Toaldo, Rosalba Minelli, Eric Ciamporcero, Stefania Pizzimenti, Piergiorgio Pettazzoni, Guglielmo Roma, Mario Umberto Dianzani, Chiara Ullio, Carlo Ferretti, Chiara Dianzani, Giuseppina Barrera

**Affiliations:** 1 MerckSerono Ivrea – RBM S.p.A., Istituto di Ricerche Biomediche "A. Marxer", Colleretto Giacosa, Turin, Italy; 2 Section of General Pathology, Department of Medicine and Experimental Oncology, University of Turin, Turin, Italy; 3 Department of Science and Pharmaceutical Technology, University of Turin, Turin, Italy; Aix-Marseille University, France

## Abstract

PPARs are nuclear receptors activated by ligands. Activation of PPARγ leads to a reduction of adhesion and motility in some cancer models. PPARγ transcriptional activity can be negatively regulated by JNK-mediated phosphorylation. We postulated that the use of agents able to inhibit JNK activity could increase the effectiveness of PPARγ ligands. We analysed the effects of rosiglitazone (PPARγ ligand) and AS601245 (a selective JNK inhibitor) alone or in association on adhesion and migration of CaCo-2, HT29, and SW480 human colon cancer cells and investigated, through microarray analysis, the genes involved in these processes. Cell adhesion and migration was strongly inhibited by rosiglitazone and AS601245. Combined treatment with the two compounds resulted in a greater reduction of the adhesion and migration capacity. Affymetrix analysis in CaCo-2 cells revealed that some genes which were highly modulated by the combined treatment could be involved in these biological responses. Rosiglitazone, AS601245 and combined treatment down-regulated the expression of fibrinogen chains in all three cell lines. Moreover, rosiglitazone, alone or in association with AS601245, caused a decrease in the fibrinogen release. ARHGEF7/β-PIX gene was highly down-regulated by combined treatment, and western blot analysis revealed that β-PIX protein is down-modulated in CaCo-2, HT29 and SW480 cells, also. Transfection of cells with β-PIX gene completely abrogated the inhibitory effect on cell migration, determined by rosiglitazone, AS601245 and combined treatment. Results demonstrated that β-PIX protein is involved in the inhibition of cell migration and sustaining the positive interaction between PPARγ ligands and anti-inflammatory agents in humans.

## Introduction

PPARγ belongs to the nuclear receptor superfamily consisting of a group of approximately 50 transcription factors involved in many different biological processes and considered as important targets in the development of new drugs [1]. The PPARγ activation by agonists regulates lipid storage in adypocytes [2], inhibits proliferation and induces differentiation and apoptosis in a number of cancer cells [3]. Thus, the PPARγ ligands have been considered as potential drugs for different types of cancer. Endogenous PPARγ ligands include unsaturated fatty acids and several prostanoids such as 15-deoxy-prostaglandin J2 (15d-PGJ2) and 15-hydroxy-eicosatetranoic acid (HETE), which are metabolites of arachidonic acid [4]. Synthetic ligands comprise the insulin-sensitizing thiazolindinedione (TZD) class (troglitazone, pioglitazone and rosiglitazone) that are used to treat diabetes mellitus [5]–[6] and several non-steroidal anti-inflammatory drugs (NSAIDs), in particular indomethacin and ibuprofen, that are weak PPARγ agonists at high micromolar concentrations [7].

The sensitivity of the different cell types to PPARγ ligands mostly depends on the PPARγ expression and activity. High levels of PPARγ expression have been reported in adipose and colon tissues. The latter is the major tissue expressing PPARγ in epithelial tissues [1]. Despite this observation, the number of studies investigating PPARγ in human subjects with colon cancer is limited. In specimens from colon cancer patients, immunohistochemical analysis demonstrated a correlation between PPARγ and cell cycle-related molecules but no association was detected between PPARγ and patient survival [8]. More recently, Ogino and collaborators demonstrated, in colorectal cancer patients, that the expression of PPARγ is associated with a good prognosis [9], in accordance with the previous data reported by Jackson and collaborators [10], which demonstrated that PPARγ (mRNA and protein) expression levels were significantly depressed in colorectal cancer cells compared with matched non-malignant tissue. In animal studies, a deficiency in intestinal PPARγ was associated with enhanced tumorigenicity in small intestine and colon of ApcMin/+ mice [11]. Similarly, in mouse models of colon cancer, PPARγ agonists inhibited tumor growth or colon carcinogenesis [12]–[14]. These data support the hypothesis that PPARγ ligands may inhibit colorectal tumour progression and may be an important therapeutic target.

**Figure 1:**
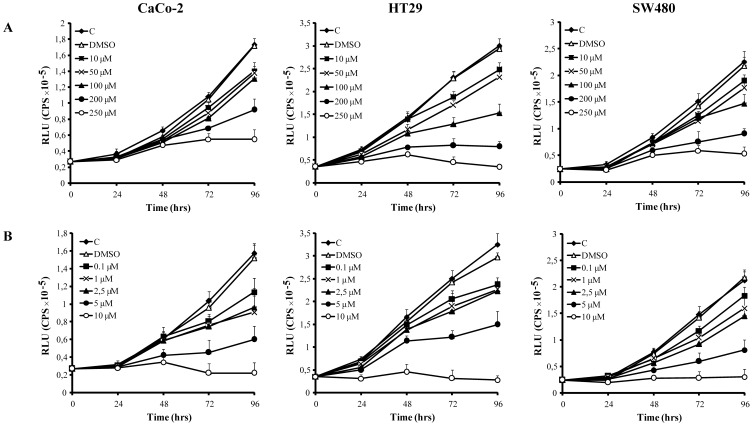
Cell proliferation. A. Effect of different doses of rosiglitazone (10, 50, 100, 200, 250 µM) on CaCo-2, HT29 and SW480 cell proliferation; B. effect of different doses of AS601245 (0.1, 1, 2.5, 5, 10 µM) on CaCo-2, HT29 and SW480 cell proliferation. Values are detected by measuring the luminescence released by the metabolically active cells. The values, expressed in RLUs are the means S.D. of three separate experiments.

One of the most important aspects in cancer progression, is the acquisition of invasive behaviour, a multi-stage process which involves cancer cells adhesion to the vassel endothelium and the motility through the extracellular matrix. It has been demonstrated that PPARγ agonists affect these parameters not only in the control of cell inflammation [15] but also in several types of cancer cells [16], [17].

Besides ligand binding, PPARγ genomic activity can be modulated by various cellular processes.

Indeed, mitogenic hormones, growth factors and pro-inflammatory signals are all known to reduce the ability of PPARγ to respond to ligand stimulation. The mechanisms that control this down regulation are complex and comprise phosphorylation [18], ubiquitination [19], sumoylation [20] and cytoplasmic shuttling [21]. A key down-regulating mechanism involves phosphorylation by various mitogen activated kinases (MAPKs), which are central signalling components in the regulation of cell proliferation, differentiation survival, stress response and apoptosis [22]. In particular, it has been reported that c-Jun-terminal kinase (JNK) phosphorylates PPARγ and negatively regulates its transcriptional activity [23]. On the basis of these observations we postulated that the use of agents able to inhibit JNK activity could increase the effectiveness of PPARγ ligands. AS601245 [1,3-Benzothiazol-2-yl-(2-{[2-(3-pyridinyl)ethyl]amino}-4-pyrimidinyl) acetonitrile; JNK inhibitor V] has been selected as a potent and selective JNK inhibitor with anti-inflammatory properties [24], and has been used in the present work to assess its involvement with anti-carcinogenic effects displayed by rosiglitazone in colon cancer cells. In particular, we examined the effects of rosiglitazone and AS601245, alone or in association, on adhesion and migration of human colon cancer cells, and investigated the genes involved in the these processes, through microarray analysis (Affimetryx GeneChip).

**Figure 2:**
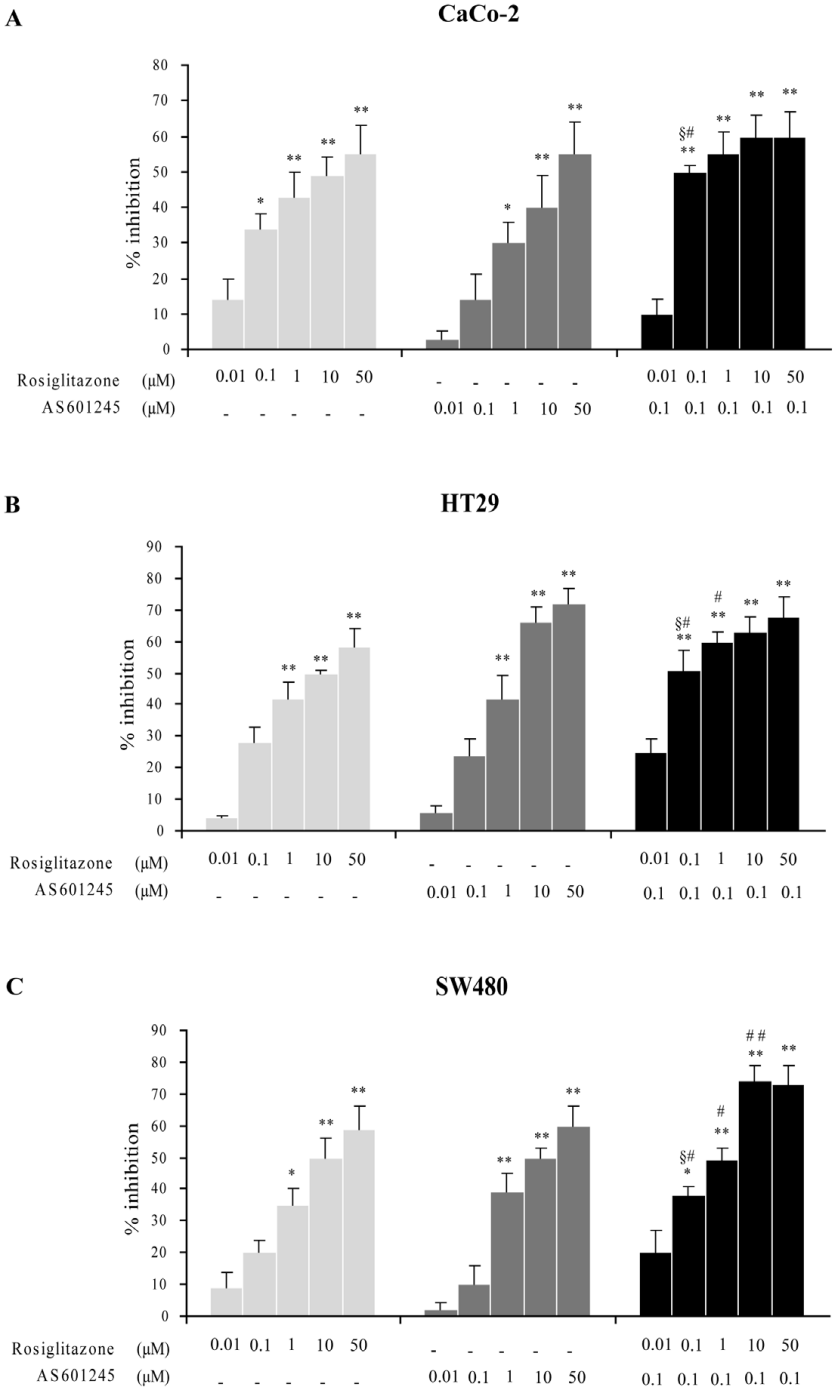
Cell adhesion. Effect of different doses of rosiglitazone (0.01, 0.1, 1, 10, 50 µM), AS601245 (0.01, 0.1, 1, 10, 50 µM) and combined treatment on CaCo-2, HT-28 and SW480 on cell adhesion to HUVECs. Cells were treated or not with the drugs for 24 hours, harvested and incubated for 1 hour on HUVEC monolayers. Data are expressed as percentage of adhesion inhibition versus untreated control cells. The control value of adhesion was about 55±6 cells per microscope field (n  = 6) for all cell lines. The values is the mean ± SD, of 3 separated experiments. Variance analysis: *p<0.05, **p<0.01 vs control; # p<0.05 vs rosiglitazone; ## p<0.01 vs rosiglitazone; § <0.01 vs both compounds.

## Materials and Methods

### Ethics Statement

The use of HUVEC was approved by the Ethics Committee of the “Presidio Ospedaliero Martini” of Turin and conducted in accordance with the Declaration of Helsinki. Written informed consent was obtained from all patients.

### Cell Culture and Treatments

CaCo-2, SW480 and HT29 colon cancer cells were obtained from European Collection of Cell Cultures (ECACC) and cultured at 37°C in a humidified atmosphere of 5% CO_2_-air. ECACC provides full quality control and authentication procedures for the cell lines. These cell lines have been selected because they represent three cell models with different potentials of invasiveness: higher in HT29 cells, middle in Caco-2 cells and lower in SW480 cells. Cells were grown in D-MEM medium (CaCo-2 and SW480 cells) or McCoy’s medium (HT29 cells) supplemented with 10% fetal calf serum (FCS, HyClone, Italy), 2 mM glutamine, 1% non essential amino acids solution and 1% antibiotic mixture (penicillin-streptomycin) (Sigma, Milano, Italy).

**Figure 3:**
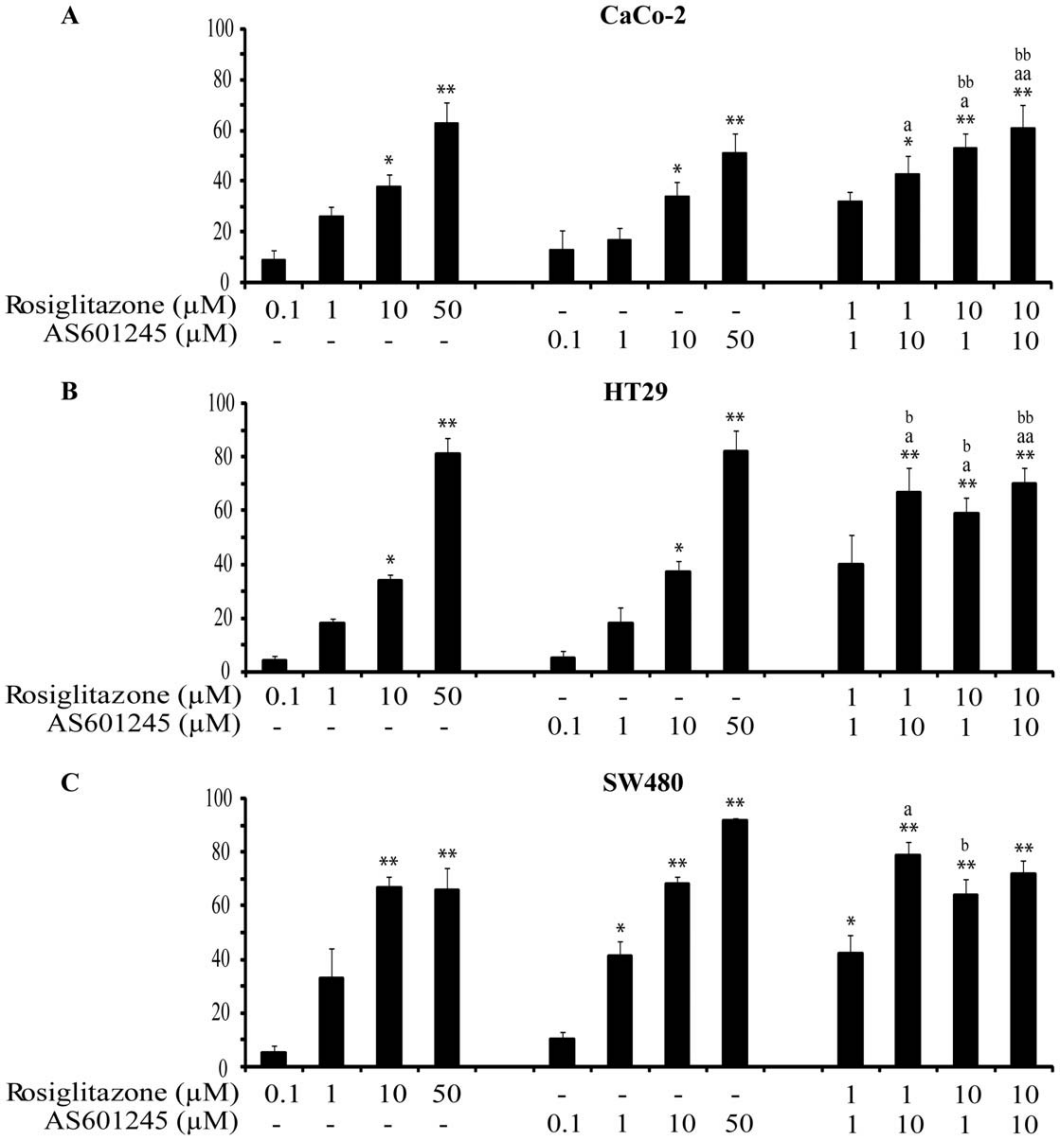
Cell migration. Inhibition of tumour migration by a Boyden chamber assay. CaCo-2, HT29 and SW480 cells were plated onto the apical side of Matrigel-coated filters in serum-free medium supplemented with drugs at different concentrations (0.1, 1, 10, 50 µM rosiglitazone; 0.1, 1, 10, 50 µM AS601245) and with the association of the drugs at different concentrations for 24 hours. Chemoattractant utilized was 20% FCS supplemented medium, placed in the basolateral chamber. The cells migrated to the bottom of the filters were stained using crystalviolet and counted (5 fields of each triplicate filters) using an inverted microscope. Control migration was 45±8 cells/microscope fields for CaCo-2 cells, 50±5 cells/microscope fields for HT29 and 40±3 cells/microscope fields for SW480. Data are expressed as mean±SEM (n = 5) of the percentage of inhibition versus the migration of cells exposed to vehicle (1% DMSO). Variance analysis *p<0.05, **p<0.01 vs control; a *p*<0.05, aa *p*<0.01 vs rosiglitazone; b *p*<0.05, bb *p*<0.01 vs AS601245.

Treatments with rosiglitazone and AS601245 [1,3-Benzothiazol-2-yl-(2-{[2-(3-pyridinyl)ethyl]amino}-4-pyrimidinyl) acetonitrile; JNK inhibitor V] (SPRI, Geneva, Switzerland) were performed by resuspending the drugs in DMSO. The concentration of vehicle in culture did not exceed 1%. Moreover, cultures, treated with 1% DMSO alone, were performed to exclude the vehicle’s effects.

HUVEC were isolated as described elsewhere [25] and cultured on gelatin-coated culture dishes in M199 medium supplemented with 20% heat-inactivated FCS, 100 U/ml penicillin, 100 µg/ml streptomycin, 5 U.I./ml heparin, 12 µg/ml bovine brain extract and 200 mM glutamine. HUVEC were utilized at II-V passages.

### Cell Proliferation and Viability

Cell proliferation was evaluated by the kit “CellTiter-Glo Luminescent Cell Viability Assay” (Promega, Milano, Italy). This assay detects the luminescence released by the metabolically active cells. Quantification of luminescence was expressed as RLU (Relative Light Unit). For the proliferation experiments, treatments were performed by adding the drugs (at different concentrations) to the cells seeded at about 4,000 cells/well in a 96-well plate.

**Figure 4:**
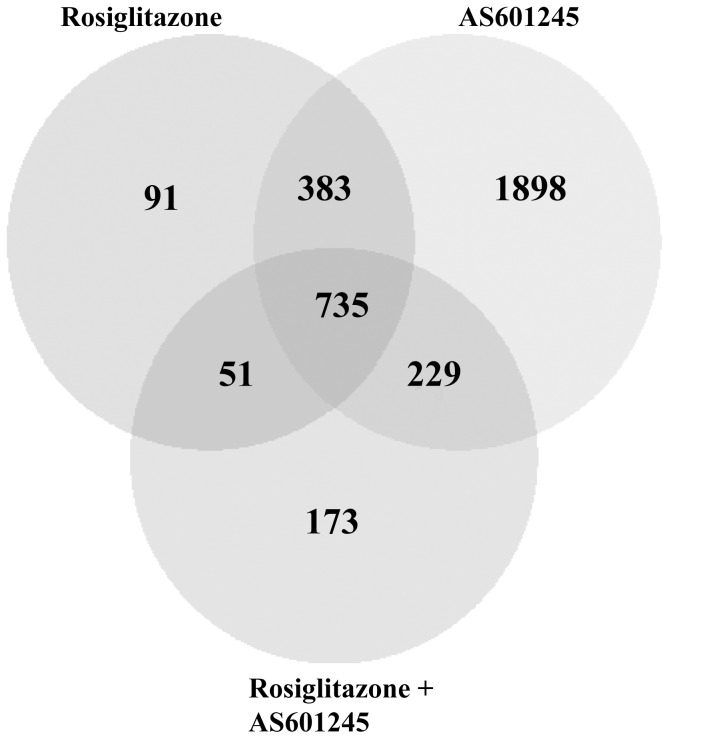
Venn Diagram. Gene expression in CaCo-2 cells treated with 50 µM rosiglitazone, 0.1 µM AS601245 and the association of these two compounds (Rosiglitazone + AS601245) was detected by microarray analysis 24 hours after the treatment. Venn diagram shows the number of genes modulated by the treatments.

**Table 1:** Biological functions.

ROSIGLITAZONE		
Biofunction	Genes p-value range	#Genes
Cancer	1.20E−12-1.84E−02	304
Genetic Disorder	2.71E−10-1.84E−02	328
Gastrointestinal Disease	3.98E−10-1.84E−02	114
Cell Death	1.26E−08-1.84E−02	213
Cell Cycle	5.04E−08-1.84E−02	135
Cellular Assembly and Organization	1.30E−05-1.84E−02	103
Cellular Growth and Proliferation	1.38E−05-1.84E−02	233
Reproductive System Disease	1.69E−05-1.84E−02	150
Neurological Disease	1.98E−05-1.69E−02	175
Protein Trafficking	2.05E−05-8.60E−03	33
**AS601245**		
**Biofunction**	**Genes p-value range**	**#Genes**
Cell Cycle	7.75E−15-1.24E−02	301
RNA Post-Transcriptional Modification	1.02E−14-7.64E−03	98
Cancer	3.54E−14-1.24E−02	658
Cell Death	1.46E−13-1.07E−02	511
Gastrointestinal Disease	4.37E−11-1.13E−02	214
Genetic Disorder	1.22E−02-4.33E−10	718
Cellular Growth and Proliferation	1.23E−09-1.21E−02	539
Molecular Transport	9.69E−09-1.01E−02	124
Respiratory Disease	1.09E−08-7.87E–03	128
Neurological Disease	4.32E−08-1.13E−02	410
**ROSIGLITAZONE +AS601245**		
**Biofunction**	**Genes p-value range**	**#Genes**
Cancer	8.05E−13-1.60E−02	314
Cell Death	9.65E−13-1.60E−02	229
Genetic Disorder	9.47E−10-1.53E−02	305
Gastrointestinal Disease	2.10E−08-1.38E−02	114
Cell Cycle	1.74E−07-1.60E−02	119
Cellular Growth and Proliferation	2.51E−07-1.60E−02	228
Amino Acid Metabolism	1.18E−05-9.89E−03	18
Small Molecule Biochemistry	1.18E−05-1.53E−02	85
Neurological Disease	1.81E−05-1.55E−02	179
Connective Tissue Disorders	2.78E−05-1.60E−02	51

Genes affected by 50 µM rosiglitazone, by 0.1 µM AS601245, and by the combined treatment with rosiglitazone and AS601245, with respect to DMSO treated CaCo-2 cells, arranged according to the relative biological functions.

### Adhesion Assay

HUVEC were grown to confluence in 24-well plates, washed, and left in place for one day in M199 medium plus 10% FCS. Commercial fluorescent cell linker mini kit PKH67 (Sigma) was used for membrane labeling of colon cancer cells. The staining efficiency was monitored by fluorescent microscopy. Colon cancer cells (CaCo-2, SW480 and HT29 cells) treated or untreated (24 hours) with rosiglitazone or AS601245 (50-0.01 µM) or both substances, were harvested and labeled as described above, and plated (1−3×10^5^ cells/wells) in a final volume of 0.25 ml M199 medium on untreated HUVEC and left in place for 1 h at 37°C in 5% CO_2_. After incubation, non-adherent cells were removed by washing 3 times with 1 ml M199 medium. The center of each well was analyzed by fluorescence image analysis [26]. Adherent cells were counted using Image Pro Plus Software for micro-imaging (Media Cybernetics, version 5.0). Single experimental points were assayed in quadruplicate, and the standard error of the four replicates was below 10% in all cases.

**Figure 5:**
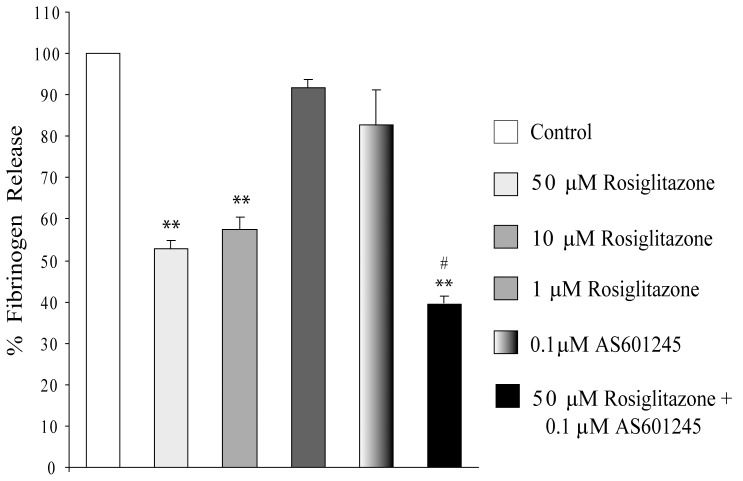
Fibrinogen release. Fibrinogen release in CaCo-2 cells exposed to different concentration (1-10-50 µM) of rosiglitazone, 0.1 µM AS601245 or both substances (50 µM rosiglitazone and 0.1µM AS601245). Data are expressed as percentage of fibrinogen release with respect to the control value and is the mean ± SD of 3 separate experiments. Variance analysis: **p<0.01 vs control, # p<0.05 vs rosiglitazone.

**Table 2:** The top ten genes modulated by rosiglitazone.

Gene ID	Gene name	Fold-Change
CYP1A1	cytochrome P450, family 1, subfamily A, polypeptide 1	+9.691
MT1X	metallothionein 1X	+6.064
MT1E	metallothionein 1E	+6.004
MT1G	metallothionein 1G	+5.627
MT1H	metallothionein 1H	+4.919
MT2A	metallothionein 2A	+4.446
CPT1A	carnitine palmitoyltransferase 1A	+4.247
HMGCS2	3-hydroxy-3-methylglutaryl-Coenzyme Asynthase 2	+3.707
GANAB	glucosidase, alpha; neutral AB	+3.196
MT1M	metallothionein 1M	+2.799
FGA	fibrinogen alpha chain	−3.426
GAS2	growth arrest-specific 2	−3.402
HNRNPA1	heterogeneous nuclear ribonucleoprotein A1	−2.925
SSH3	slingshot homolog 3	−2.758
STIP1	stress-induced-phosphoprotein 1	−2.594
SORBS2	sorbin and SH3 domain containing 2	−2.572
EPN1	epsin 1	−2.562
FGFR2	fibroblast growth factor receptor 2	−2.551
RPS27A	ribosomal protein S27a	−2.494
MACF1	microtubule-actin crosslinking factor 1	−2.458

The top ten genes up-regulated and down-regulated the most by the 50 µM rosiglitazone treatment, with respect to DMSO-treated CaCo-2 cells.

### Matrigel Migration Assay

Chemotaxis assay of cancer cells was performed by the Boyden chamber method using a filter of 8.2 mm diameter and 5.0 µm pore size (Neuro Probe, Inc.; BIOMAP snc, Milan Italy) coated with 0.1 µl/ml Matrigel (BD Matrigel™ Matrix; BD Biosciences, Oxford, UK). Briefly, medium containing 20% FCS as a chemoattractant was placed in the lower wells. Initially, CaCo-2, HT29 and SW480 cells (at final concentration of 5 x 10^4^ cells/ml) were suspended in the upper wells (about 4000 cells/well for HT29 and 8000 cell/well for SW480 and CaCo-2) in serum-free medium supplemented with drugs at different concentrations (from 0.1 µM to 50 µM for both substances) and with the association of the drugs at different concentrations. The migration of control untreated cells, cell exposed to the vehicle (1% DMSO) and of control and treated cells exposed to mitomycin C (50 µg/ml) (Sigma) was also analyzed. The chamber was incubated at 37°C under 5% CO_2_ for 24 hours. Chemotaxis was quantified by counting the stained cells that migrated to the lower side of the filter by using an optical microscope (magnification x 100). The stained cells were counted as the mean number of cells per 5 random fields for each assay. Results are expressed as percentage of the inhibition of treated cells versus the migration measured in cell exposed to 1% DMSO. These conditions were maintained for transfected cells, also.

**Table 3:** The top ten genes modulated by AS601245.

Gene ID	Gene name	Fold-Change
CYP1A1	cytochrome P450, family 1, subfamily A, polypeptide 1	+5.329
AP3D1	adaptor-related protein complex 3, delta 1 subunit	+3.634
NFAT5	nuclear factor of activated T-cells 5, tonicity-responsive	+3.048
BMP2K	bone morphogenetic protein 2 inducible kinase	+2.889
DLST	dihydrolipoamide S-succinyltransferase (E2 component of 2-oxo-glutarate complex)	+2.839
IL6ST	interleukin 6 signal transducer (gp130, oncostatin M receptor)	+2.830
MGA	MAX gene associated	+2.361
THRAP3	thyroid hormone receptor associated protein 3	+2.360
GANAB	glucosidase, alpha; neutral AB	+2.321
TWF1	twinfilin, actin-binding protein, homolog 1	+2.175
RPS27A	ribosomal protein S27a	−4.163
HNRNPA1	heterogeneous nuclear ribonucleoprotein A1	−4.084
WDR33	WD repeat domain 33	−3.869
EPN1	epsin 1	−3.662
KLC2	kinesin light chain 2	−3.657
STIP1	stress-induced-phosphoprotein 1	−3.591
SEPT9	septin 9	−3.409
TFDP1	transcription factor Dp-1	−3.141
LAD1	ladinin 1	−3.134
C16ORF58	chromosome 16 open reading frame 58	−3.121

The top ten genes up-regulated and down-regulated the most by the 0.1 µM AS601245 treatment, with respect to DMSO-treated CaCo-2 cells.

### Fibrinogen Release Determination

Fibrinogen (FBG) release in culture media was analysed by using the AssayMax Human Fibrinogen (FBG) ELISE Kit from Assaypro (St. Charles, MO, USA) according to the manufacturer’s protocol.

CaCo-2 cells were grown in flasks and seeded at the concentration of 4 x 10^6/^flask. Cells, exposed to rosiglitazone (50, 10 and 1 µM), AS601245 (0.1 µM) or both substances (50 µM rosiglitazone and 0.1 µM AS601245), were harvested after 24 hours from the beginning of experiment and centrifuged. The collected supernatants were incubated in a 96-well plate coated with a polyclonal antibody specific for human FBG. FBG in samples was sandwiched by the immobilized polyclonal antibody and biotinylated polyclonal antibody specific for human FBG, which was recognized by a streptavidin-peroxidase conjugate. All unbound material was washed away and a peroxidase enzyme substrate was added. The colour development was stopped and the intensity of the colour was measured in a microplate reader at a wavelength of 450 nm.

**Table 4:** The top ten genes modulated by the treatment with rosiglitazone + AS601245.

Gene ID	Gene name	Fold-Change
CYP1A1	cytochrome P450, family 1, subfamily A, polypeptide 1	+11.092
IL6ST	interleukin 6 signal transducer (gp130, oncostatin M receptor)	+4.150
MT1X	metallothionein 1X	+4.047
AP3D1	adaptor-related protein complex 3, delta 1 subunit	+3.945
NFAT5	nuclear factor of activated T-cells 5, tonicity-responsive	+3.891
GANAB	glucosidase, alpha; neutral AB	+3.693
CPT1A	carnitine palmitoyltransferase 1A	+3.677
MT1G	metallothionein 1G	+3.651
MT1E	metallothionein 1E	+3.527
PLAUR	plasminogen activator, urokinase receptor	+3.412
FGA	fibrinogen alpha chain	−5.578
ARHGEF7	Rho guanine nucleotide exchange factor (GEF) 7	−4.917
SI	sucrase-isomaltase (alpha-glucosidase	−3.944
HNRNPA1	heterogeneous nuclear ribonucleoprotein A1	−3.848
FGG	fibrinogen gamma chain	−3.733
ANXA9	annexin A9	−3.665
G0S2	G0/G1switch 2	−3.506
FGB	fibrinogen beta chain	−3.174
DEFB1	defensin, beta 1	−3.137
FGFR2	fibroblast growth factor receptor 2	−3.183

The top ten genes up-regulated and down-regulated the most by combined treatment with 50 µM rosiglitazone and 0.1 µM AS601245, with respect to DMSO-treated CaCo-2 cells.

### RNA Extraction and Array Hybridization

CaCo-2 cells were treated with vehicle (1% DMSO) or 50 µM rosiglitazone or 0.1 µM AS601245 or rosiglitazone plus AS601245 for 24 hours, and total RNA from 3 biological replicates of each treatment was isolated using TRIzol reagent (Invitrogen, Milano, Italy). Samples were treated with DNase in order to avoid any genomic contamination. The quality of the resulting RNA was determined by using the Agilent 2100 Bioanalyzer (Agilent Technologies). The RNA content was normalized by using the Thermo Scientific NanoDrop™ ND-1000 spectrophotometer. RNA samples of each replicate were analyzed by using Affymetrix GeneChip Human Genome U133A plus 2.0 chips (Affymetrix). RNA amplification, double-stranded cDNA synthesis and generation of biotin-labeled cRNA by in vitro transcription (IVT) were performed according to the manufacturer’s protocol using Affymetrix kits. The final cDNA was checked for quality and quantity before fragmentation and chip hybridization. Each chip was then washed and stained using a Fluidics station 450 (Affymetrix). Fluorescence intensity for each chip was captured with an Affymetrix GeneChip Scanner 3000 (Affymetrix). GCOS software version 1.2 (Affymetrix) was used to define the probe cell and calculates the intensity for each cell. CEL files generated, containing the summary intensities for each probe, were analyzed through the following bioinformatics approaches. We firstly assessed the overall data quality using R/Bioconductor. Then, we loaded the dataset into the Rosetta Resolver for data normalization, generation of expression values, and statistical analysis. Expression values of all treatment groups were obtained as ratio versus the negative control (1% DMSO). Differential analyses between pairs of groups were performed with 1-way ANOVA followed by the Benjamini-Hochberg multiple testing correction (False Discovery Rate-FDR cut-off of 1%) and a Student-Newman-Keuls post hoc analysis. Finally, differentially expressed genes were analysed in Ingenuity Pathway Analysis software version 7.5 (Ingenuity® Systems, www.ingenuity.com).

**Table 5:** Up-regulated genes containing PPRE sequences.

Rosiglitazone		
Gene ID	Gene name	Fold-Change
CYP1A1	cytochrome P450, family 1, subfamily A, polypeptide 1	9.691
HMGCS2	3-hydroxy-3-methylglutaryl-Coenzyme A synthase 2	3.707
DUSP6	dual specificity phosphatase 6	1.987
KLF4	Kruppel-like factor 4	1.92
HOXB8	homeobox B8	1.703
ADFP	adipose differentiation-related protein	1.584
FABP1	fatty acid binding protein 1, liver	1.441
**AS601245**		
**Gene ID**	**Gene name**	**Fold-Change**
CYP1A1	cytochrome P450, family 1, subfamily A, polypeptide 1	5.329
**Rosiglitazone + AS601245**		
**Gene ID**	**Gene name**	**Fold-Change**
CYP1A1	cytochrome P450, family 1, subfamily A, polypeptide 1	11.092
PANK2	pantothenate kinase 2	2.966
DUSP6	dual specificity phosphatase 6	2.933
MARCKS	myristoylated alanine-rich protein kinase C substrate	2.275
HMGCS2	3-hydroxy-3-methylglutaryl-Coenzyme A synthase 2	2.264
KLF4	Kruppel-like factor 4	2.201
TBL1XR1	transducin (beta)-like 1 X-linked receptor 1	2.114
ABCG2	ATP-binding cassette, sub-family G (WHITE), member 2	2.082
ANKRD12	ankyrin repeat domain 12	1.975
SPEN	spen homolog, transcriptional regulator	1.953
LMCD1	LIM and cysteine-rich domains 1	1.875
ADFP	adipose differentiation-related protein	1.864
FOSL1	FOS-like antigen 1	1.844
FMNL2	formin-like 2	1.751
KLF5	Kruppel-like factor 5	1.526
ITGA2	integrin, alpha 2 (CD49B, alpha 2 subunit of VLA-2 receptor)	1.521
LNPEP	leucyl/cystinyl aminopeptidase	1.406

The genes harbouring PPRE sequences among the up-regulated genes by treatment with 50 µM rosiglitazone, 0.1 µM AS601245 and combined treatment versus DMSO-treated CaCo-2 cells.

### RealTime RT-PCR

To confirm the Affymetrix results, we selected 5 genes that had shown a >2-fold change in expression for further study by real time PCR in a separate experiment. Two of these (GANAB and MT2A) were found to be increased in Affymetrix analysis, whereas the others two were found to be decreased (FGA and FGFR2). Finally, one gene (ARHGEF7) was found to be highly decreased in combined treatment, only. Moreover, we analysed the expression of the three chains of fibrinogen in CaCo-2, HT29 and SW480 cells. Cells were treated with 1% DMSO as the vehicle or with 50 µM rosiglitazone, 0.1 µM AS601245, or rosiglitazone plus AS601245 for 24 hours; total RNA from 3 biological replicates of each treatment was isolated using TRIzol reagent (Invitrogen, Milano, Italy), then total RNA was quantified with a NanoDrop (Thermo scientific) spectrophotometer to measure RNA concentration and analyzed on an Agilent 2100 Bioanalyser to monitor RNA quality and integrity. Total RNA was reverse transcribed into cDNA in a 20 µl reaction using the TaqMan® High Capacity cDNA Reverse Transcription Kit provided by Applied Biosystems. 500 ng of total RNA was used as the starting material from each sample. For each RealTime PCR reaction, the reverse transcribed sample was used as a template. To test assay linearity, a cDNA pool was first serially diluted. Reactions were carried out in the presence of the commercial TaqMan® Gene Expression Assays and a 1× concentration of the TaqMan® Universal PCR Master Mix (Applied Biosystems). Target mRNA was quantified using an ABI 7900 HT Fast RealTime PCR system (Applied Biosystems). Primers were purchased from Applied Biosystems: GANAB (glucosidase, alpha; neutral AB, Hs00929274_m1), MT2A (metallothionein 2A, Hs02379661_g1), FGA (fibrinogen alpha chain, Hs00241027__m1_), FGB (fibrinogen beta chain Hs00905942__m1_) FGG (fibrinogen gamma chain Hs00241037__m1_) FGFR2 (fibroblast growth factor receptor 2, Hs01552926_m1) and ARHGEF7 (Rho guanine nucleotide exchange factor (GEF) 7, Hs00388776_m1). The TaqMan probes were labelled with a 5' reporter dye (FAM, 6-carboxyfluorescein) and a 3' quencher dye (TAMRA, 6-carboxytetramethylrhodamine). RealTime PCR reactions were carried out in triplicate in microamp optical 384-well plates in a total volume of 10 µl/well. Data were analyzed by the ABI Sequence Detection System (SDS) software using the relative quantification. The fold changes are determined by the ΔΔCt method as described in Applied Biosystems User Bulletin No. 2. Briefly, the level of each target mRNA was normalized to the level of the 18S ribosomal RNA (housekeeping gene) in order to obtain the ΔCt and then the ΔΔCt was calculated against the DMSO treatment.

**Figure 6:**
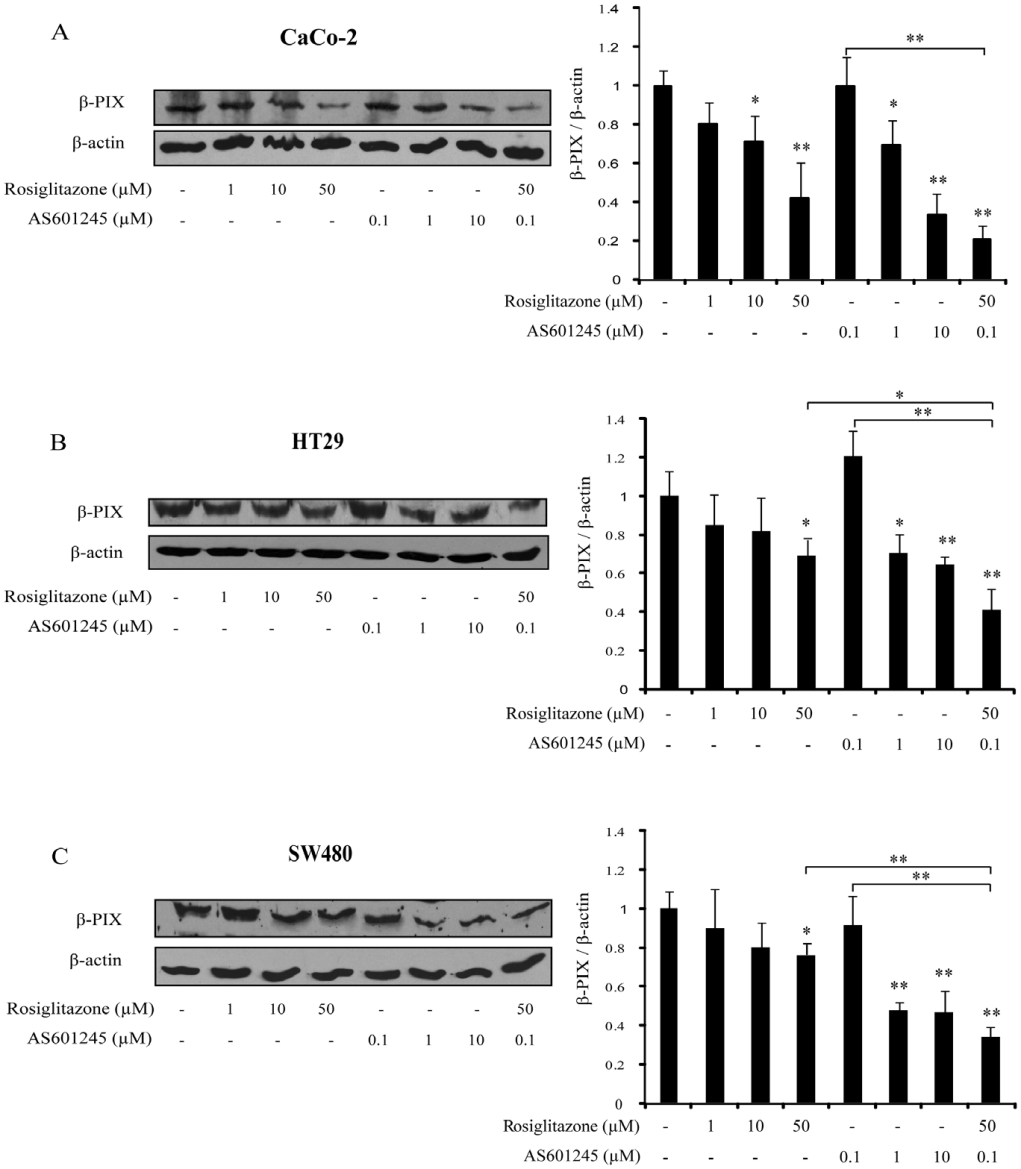
β-PIX protein expression. Western blot analysis of β-PIX expression in CaCo-2, HT29 and SW480 cells, treated for 24 hours with different concentrations of rosiglitazone (1, 10, 50 µM), AS601245 (0.1, 1, 10) and combined treatment with 50 µM rosiglitazone and 0.1 µM AS601245. Equal protein loading was confirmed by exposure of the membranes to the anti-β-actin antibody. Quantification of protein products (on the right) was performed by densitometric scanning. Data are normalized using the β-actin signal and are indicated as means ± SD from three independent experiments. (A) Western blot analysis of CaCo-2 cells and relative densitometric values. (B) Western blot analysis of HT29 cells and relative densitometric values. (C) Western blot analysis of SW480 cells and relative densitometric values. Variance analysis: * p<0.05, **p<0.01 vs control or rosiglitazone treated cells.

### β-PIX Transfection

The β-PIX plasmide was purchased by Qiagen (EIM0195303), propagated in Escherichia coli Competent Cells (Promega) following standard procedures and purified employing the EndoFree Plasmide Midi Kit (Qiagen). For transient transfection experiments, CaCo-2, HT29 and SW-480 cells were grown in six-wells plate at 80% confluence, 6 µg of purified plasmide constructs were transfected using Lipofectamine 2000 reagent (Invitrogen), according to the manufacturer's instruction. β-PIX expression was evaluated 24 hours after transfection, by western blot. For negative control, pQE-TriSystem-6 Vector (Qiagen) was used. At least three independent experiments for each condition were performed.

### Statistical Analysis

The 2-way ANOVA was performed in the proliferation, adhesion, migration, fibrinogen release tests and densitometric analysis of western blots.

**Table 6:** Gene expression detected by affymetrix and quantitative Real-Time PCR.

	Rosiglitazone		AS601245		Rosiglitazone+AS601245	
Gene	Affymetrix	qPCR (TaqMan)	Affymetrix	qPCR (TaqMan)	Affymetrix	qPCR (TaqMan)
**GANAB**	**3.2**	**2.5**	**2.3**	**2.8**	**3.7**	**2.8**
**MT2A**	**4.4**	**5.1**	**unchanged**	−**1.2**	**3.0**	**5.1**
**FGA**	−**3.4**	−**2.8**	**unchanged**	−**2.0**	−**5.6**	−**4.7**
**FGFR2**	−**2.6**	−**2.2**	−**2.1**	−**1.7**	−**3.2**	−**3.5**
**ARHGEF7**	−**1.4**	−**2.1**	−**1.7**	−**1.8**	−**4.9**	−**4.2**

Gene expression detected by affymetrix and quantitative real-time reverse transcription PCR in CaCo-2 cells treated with 50 µM rosiglitazone, 0.1 µM AS601245 and combined treatment. Results are expressed as fold change versus DMSO-treated control cells.

## Results

### Rosiglitazone and AS601245 Affect Cell Proliferation

Preliminary experiments demonstrated that cell proliferation was affected by rosiglitazone and by AS601245 until 96 hours, in a concentration-dependent way in all three lines of colon cancer cells tested (Fig. 1). However, the drug doses effective in inhibiting cell proliferation by 50% (IC50) were rather high. In CaCo-2 cells, IC50 was 150±25 µM for rosiglitazone and 2.5±1 µM for AS601245. The doses able to reduce cell proliferation by 20% (IC20) were: 50 µM ±12 for rosiglitazone and 0.1 µM ±0.1 for AS601245**.** IC50 and IC20 doses were very similar in all three cell lines tested. The IC20 doses of rosiglitazone and AS601245, defined in CaCo-2 cells, were then used for the microarray experiments in this cell line.

**Table 7:** Expression of fibrinogen chains detected by quantitative Real Time PCR.

Cell line	Gene	Rosiglitazione	AS601245	Rosiglitazone + AS601245
**CaCo-2**	**FGA**	−2,8	−2,0	−4.7
	**FGB**	−2.2	−1.5	−3.0
	**FGG**	−1.8	−1.9	−3.6
**HT29**	**FGA**	−1.4	−2.4	−3.8
	**FGB**	−1.4	−1.6	−2.4
	**FGG**	−1.6	−1.5	−2.8
**SW480**	**FGA**	n.d.	n.d.	n.d.
	**FGB**	−1.4	−2.9	−3.1
	**FGG**	−1.6	−1.2	−3.7

Expression of fibrinogen chains (FGA: fibrinogen chain α; FGB: fibrinogen chain β; FGG: fibrinogen chain γ) detected by quantitative real time reverse transcription PCR, in cells treated with 50 µM rosiglitazone, 0.1 µM AS601245 and combined treatment. Results are expressed as fold change versus DMSO-treated control cells. n.d.: not detectable.

### Cell Adhesion and Migration After Rosiglitazone and AS601245 Treatments

In the adhesion assay, performed after 24 hours from the start of treatment, the addition of 50 µM rosiglitazone alone resulted in a reduction of cell adhesion to HUVEC by about 55% in CaCo-2 cells and by 58% and 59% in HT29 and SW480 cells, respectively (Fig. 2). The level of inhibition was gradually reduced by treating the cells with lower doses of rosiglitazone. In a first set of experiments, cell adhesion capacity was assayed in HCT116 cells, also. In this cell line, cell adhesion was inhibited by 71% after treatment with 50 µM rosiglitazone and by 59% after treatment with 10 µM rosiglitazone (data not shown). The treatment with 50 µM AS60124 reduced the adhesion by about 55, 72 and 60% in CaCo-2, HT29 and SW480 cells, respectively. The percentage of inhibition was gradually reduced by treating the cells with lower doses of AS601245 and the treatment with 0.1 µM AS601245 did not significantly affect this parameter. To verify whether the combined treatment of rosiglitazone and AS601245 could have an additive effect in inhibiting cell adhesion, 0.1 µM AS601245 was associated with increasing doses of rosiglitazone. In all three cell lines the combination of 0.1 µM rosiglitazone with 0.1 µM AS601245 showed an additive effect in reducing cell adhesion. The combined treatment of 0.1 µM AS601245 with the higher doses of rosiglitazone increased the effect of rosiglitazone alone reaching the maximum level of inhibition. All three types of cells showed a similar response to the treatments.

**Figure 7:**
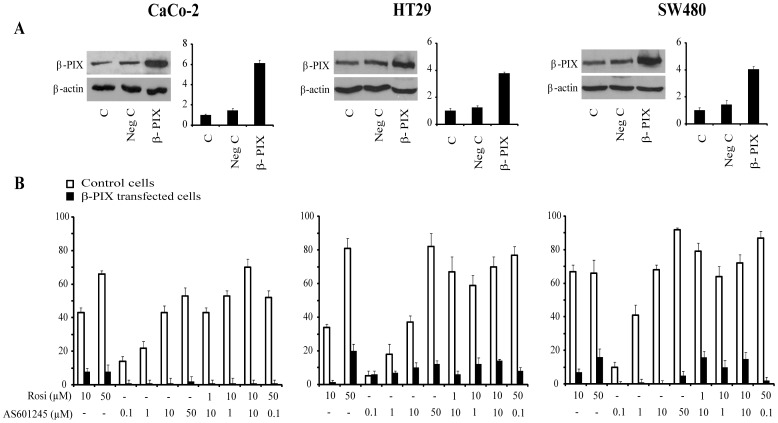
Migration of β-PIX transfected colon cancer cells. A. Western blot expression of CaCo-2, HT29 and SW480 cells of control and transfected with the empty plasmide and with the plasmide carrying β-PIX gene. B. Inhibition of tumour cell invasion by a Boyden chamber assay in cells tranfected with β-PIX carrying plasmide. Control cells and transfected cells were plated onto the apical side of Matrigel-coated filters in serum-free medium supplemented with drugs at different concentrations (10, 50 µM rosiglitazone; 0.1, 1, 10, 50 µM AS601245) and with the association of different drug concentrations for 24 hours. Chemoattractant utilized was 20% FCS supplemented medium, placed in the basolateral chamber. The cells migrated to the bottom of the filters were stained using crystalviolet and counted (5 fields of each triplicate filters) using an inverted microscope. Control migration was 45±8, 51±8 and 33±4 cells/microscope field for CaCo-2, HT29, SW480 cells, respectively, and 49±5, 50±6 and 30±6 cells/microscope field for CaCo-2, HT29 and SW480 cells, respectively, after transfection with β-PIX carrying plasmide and empty plasmide. Data are expressed as mean ± SEM (n = 5) of the percentage of inhibition versus the control migration.

Migration was examined in CaCo-2, HT29, and SW480 cell lines. The number of migrated cells/microscope field in untreated control cells was 45±8 for CaCo-2 cells, 50±3 for HT29 cells and 40±3 for SW480 cells. The value of migration of control cells exposed to the vehicle (1% DMSO) was 44±6 for CaCo-2 cells, 51±8 for HT29 cells and 40±5 for SW480 cells. Moreover, to exclude that the results obtained in treated cells may depend on cell proliferation, we analysed cell migration in untreated cells and in cells treated for 24 hours with the highest concentrations of rosiglitazone or AS601245 (50 µM) in presence of mitomycin C. Results obtained demonstrated that the migration values were similar in absence or in presence of mitomycin C (cells/microscope field was 45±8 for control CaCo-2 cells and 44±6 for CaCo-2 control cells treated with mitomycin; 50±3 for control HT29 cells and 49±5 for cells treated with mitomycin; 40±3 for control SW480 cells and 42±8 for cells treated with mitomycin). The percentages of inhibition in rosiglitazone treated cells were: 65% and 64% for CaCo-2 cells, 79% and 80% for HT29 cells and 60% and 63% for SW480, with and without mitomycin, respectively; the percentages of inhibition of AS601245 treated cells were: 55% and 50% for CaCo-2 cells, 80% and 75% for HT29 cells and 92% and 87% for SW480 cells, with and without mitomycin, respectively).

In Fig. 3 are reported the migration results obtained in cells treated with rosiglitazone, AS601245 and both substances, expressed as the percentage of inhibition with respect the migration of cells exposed to the vehicle only (1% DMSO). The migration was significantly inhibited by 10 and 50 µM rosiglitazone and AS601245 in all three cell lines. The treatments with different combinations of concentrations under 50 µM of both compounds produced an additive effect in inhibiting cell migration. The combined treatment with concentrations of 50 µM rosiglitazone or AS601245 did not produce additive effects (data not shown), probably because these concentrations, alone, produced a percentage of inhibition close to the plateau.

### Microarray Analysis of Gene Expression in CaCo-2 Cells

To analyze whether the cell responses to the treatments with rosiglitazone, AS601245 or with both substances were a consequence of a specific gene pathway modulation, we performed the microarray analysis by using the Affimetryx GeneChip platform. Since the inhibition of cell adhesion and migration by rosiglitazone and AS601245 was very similar in all three cell lines of colon cancer examined, we select CaCo-2 cell line to perform this analysis, 24 hours after the treatments. The doses used in this study were those able to induce an IC20 inhibition of CaCo-2 cell proliferation (50 µM rosiglitazone and 0.1 µM AS601245).

The number of genes significantly modulated by rosiglitazone and by AS601245 was very high. The complete list of the genes modulated by rosiglitazone, AS601245 and by the combined treatment is reported in the Supporting Information S1. The Venn diagram (Fig. 4) shows that rosiglitazone modulated 1260 genes, AS601245 modulated 3245 genes and, the combined treatments modulated 1188 genes. Among the genes affected by rosiglitazone alone or AS601245 alone, 1118 were common in both of the two groups. The combined treatment affected 735 genes which were present in both the rosiglitazone and AS601245 groups, and 173 genes which were not affected by either rosiglitazone or AS601245, alone. Table 1 indicates the genes affected by rosiglitazone, by AS601245, and by the combined treatment with rosiglitazone and AS601245, arranged with respect to the relative biological functions and listed on the basis of the p-value. The genes mainly affected by both individual treatments belong to cancer, genetic disorders, cell cycle, cell death and gastrointestinal disease groups. The combined treatments affected mainly genes belonging to cancer and cell death functions.

The top ten genes changed the most by the rosiglitazone treatment, with respect to 1% DMSO treated cells, are reported in Tab. 2. Rosiglitazone treatment mainly increased the CYP1A1 (9.6 fold change) gene expression which encodes a member of the cytochrome P450 superfamily of enzymes and increased (from 6.06 to 2.79 fold changes) the expression of a group of genes coding for metallothioneins (MT1X, MT1E, MT1G, MT1H, MT2A, MT1M). Among genes down-regulated by rosiglitazone, the first was the FGA gene (−3.42 fold change). The other genes which codify for the fibrinogen chains (FGB and FGG) were down regulated by −2.29 and −2.02 fold, respectively. Other genes down-regulated by rosiglitazone belonged mainly to two functional groups: “cancer” (RPS27A, SORBS2, STIP1, FGA, FGFR2, SSH3, EPN1) and cell death (SORBS2, STIP1, GAS2, EPN1).

Tab.3 illustrates the genes modulated by the treatments with AS601245 with respect to 1% DMSO treated cells. In this case also, the gene most up-regulated was CYP1A1 (5.329 fold change). The other genes up-regulated belonged mainly to the “growth of cells” function (BMO2K, DLST, IL6ST, MGA) and the “transcription” function (NFAT5 and THRAP3). The down-regulated genes belonged mainly to the “cancer” biofunction (RPS27A, HNRNPA1, STIP1 and TFDP1).

After combined treatment with rosiglitazone and AS601245 (Tab. 4) the major part of the top ten up-regulated genes were up-regulated by treatments with the single substances, also. CYP1A1 was up-regulated by 11.09 fold. IL6ST was induced by AS601245 (2.8 fold change) and by the combined treatments (4.1 fold change). Metallothionein genes were induced by the combined treatment to a lesser extent than that determined by rosiglitazone treatment. Conversely, the up-regulation of AP3D1 and NFAT5 after combined treatment was similar to that observed after treatment with AS601245 alone. Among the down-regulated genes, the three genes codifying the fibrinogen chains (α, β, γ ) reached the top ten positions, while they were down-regulated to a lesser extent by rosiglitazone alone. Moreover, ARHGEF7 (Rho guanine nucleotide exchange factor)/βPIX gene was highly down-regulated by the combined treatment.

Among the genes up regulated by the rosiglitazone treatment, only a few have a PPRE putative sequence in the promoter, indicating that the major part of rosiglitazone functions were developed in a PPARγ-independent way. Since the inhibition of JNK could increase the affinity of activated PPARγ for the PPRE sequences, we investigated, among the genes activated by rosiglitazone, by AS601245 and by the combined treatment, the genes having PPRE sequences by using the genome-wide library of high-confidence predicted PPAR target genes as published by Lemay and collaborators [27] (Tab. 5). It is noteworthy that, after combined treatments, the number of activated genes, containing PPRE sequences, greatly increased (7 genes were up-regulated, after treatment with rosiglitazone alone, and 17 genes were up-regulated, after the combined treatment).

### Gene Expression Evaluation by RealTime PCR

To confirm results obtained by microarray analysis, RealTime PCR of 5 selected genes (2 up-regulated and 3 down-regulated) was performed. Results, reported in Tab. 6, indicated that the up or down fold changes obtained by microarray analysis were similar to those obtained in RealTime PCR. Some discrepancies were found only for MT2A and FGA that did not change in AS601245 treated cells if evaluated in microarray analysis, whereas they were decreased by −1.2 and −2 fold, respectively, if analysed in RealTime PCR.

To assess whether the inhibition of the expression of the three chains of fibrinogen, observed after rosiglitazone, AS601245 and combined treatment with 50 µM rosiglitazone and 0.1 µM AS601245, was a common response of the colon cancer cells, we determined by RealTime PCR the expression of the three chains of fibrinogen in CaCo-2, HT29 and SW480 cells. Results obtained are reported in Tab 7 and demonstrated that the inhibition of the expression of fibrinogen chains (except for SW480 cells, in which the α chain of fibrinogen is not detectable) was a common response to the treatments of all three cell lines.

### Fibrinogen Release

Since the expression of all three chains of fibrinogen are highly down-regulated by combined treatment with rosiglitazone and AS601245, the release of fibrinogen from CaCo-2 cells in the culture medium was determined in control cells, in cells treated with a single compound (50, 10 and 1 µM rosiglitazone or 0.1 µM AS601245) and in cells treated with both substances (50 µM rosiglitazone plus 0.1 µM AS601245). Results, reported in Fig. 5, indicate that rosiglitazone reduced in a dose-dependent way the amount of fibrinogen released in the culture medium (a 47.2% reduction after treatment with 50 µM rosiglitazone and a 42% reduction after treatment with 10 µM rosiglitazone), whereas 1 µM rosiglitazone or 0.1 µM AS601245 did not significantly affect this parameter. Interestingly, the reduction of fibrinogen release after combined treatment with 50 µM rosiglitazone and 0.1 µM AS601245 was significantly higher (a 60% reduction) than that produced by the treatment with 50 µM rosiglitazone, alone.

### Evaluation of β-PIX Protein Expression in CaCo-2, HT29 and SW480 Colon Cancer Cells

Since the microarray analysis and real-time PCR indicated that ARHGEF7/β-PIX gene was highly down-regulated after combined treatment with rosiglitazone and AS601245, we analysed the expression of β-PIX protein in CaCo-2 cells, and in the other cell lines of colon cancer previously examined in this study: HT29 and SW480 cells. The β-PIX expression was analysed after treatment with 1, 10 and 50 µM rosiglitazone, 0.1, 1 and 10 µM AS601245, and after combined treatment with 50 µM rosiglitazone and 0.1 µM AS601245. Results indicated that both rosiglitazone and AS601245 decreased in a dose-dependent way the β-PIX expression, starting from 10 µM rosiglitazone and 1 µM AS601245 in CaCo-2 cells, while in the other two lines the dose-dependence was not so evident (Fig. 6). Combined treatment was more effective in decreasing the β-PIX expression than the treatments with the highest dose of rosiglitazone in HT29 and SW480 cells, whereas in CaCo-2 cells the effect of combined treatment was not so evident.

### Evaluation of Cell Migration in β-PIX Transfected Cells

To asses the role of β-PIX protein as a target for the rosiglitazone and AS601245 inhibitory effect on cell invasiveness, we performed transient transfection of CaCo-2, HT20 and SW480 cells with plasmide constructs containing the β-PIX gene, as described under “Materials and Methods. Fig. 7 (panel A) shows β-PIX expression in CaCo-2, HT29 and SW480 cells and in cells transfected with the plasmide empty or containing β-PIX gene. The transfection with β-PIX gene resulted in a 6 fold increase of β-PIX protein with respect to the CaCo-2 control cells and about a 4 fold increase of β-PIX protein with respect to the HT29 an SW480 control cells.

To verify whether the endogenous increase of β-PIX protein could affect the response to the rosiglitazone and AS60124 treatment, we analysed the migration capacity after drug treatment in control and transfected cells. Fig. 7 (panel B) reports the percentage of migration inhibition, with respect to the untreated cells, 24 hours after treatment with different concentrations of rosiglitazone and AS601245 and the combinations of two drugs. The β-PIX transfection abrogated the inhibition of cell migration determined by rosiglitazone, AS601245 and combined treatment in all three cell lines, thus indicating that β-PIX protein was an important target for rosiglitazone and AS601245 inhibitory action.

## Discussion

Results obtained demonstrated that the combined treatment with rosiglitazone and AS601245 increases the anticancer effects of the two substances in colon cancer cells. In particular cell adhesion and migration were reduced by the rosiglitazone alone and they were further reduced by the combined treatment of rosiglitazone and AS601245.

In this paper we demonstrated that rosiglitazone strongly inhibited cell adhesion at doses (1 µM) ineffective in modulating other parameters. This important datum may be related to the inhibition of expression of all fibrinogen chains (FGA, FGB and FGG) caused by rosiglitazone alone (FGA was inhibited by −3.426 fold, FGB by −2.09 fold and FGG by −2.02 fold). Interestingly, both inhibition of cell adhesion and the inhibition of fibrinogen chain expressions were enhanced by the combined treatment with the JNK inhibitor and rosiglitazone. Although little literature data is available about the effect of PPAR ligands in cell adhesion, Reddy and collaborators [28] reported that PPARγ ligands inhibited chemotaxis of PMN suggesting that PPAR ligands influence cell adhesion and migration. Moreover, we demonstrated that rosiglitazone not only inhibited fibrinogen chain expressions, but also reduced the amount of fibrinogen released by the cells. It is well known that the increase of fibrin(ogen) is correlated with an increase of risk of metastasis [29]. These results may suggest that PPARγ ligands could effectively inhibit the first steps of the metastatic process.

The results obtained about the inhibition of migration by rosiglitazone and AS601245 also support the hypothesis that PPARγ ligands and anti-inflammatory drugs can reduce cancer cell invasiveness. Recently it has been demonstrated that PPAR γ agonists 15d-PGJ(2) and rosiglitazone significantly reduced eosinophil migration into the peritoneal cavity [30] and that ciglitazone reduced both wound-induced migration and chemotaxis of breast cancer cells [31] in a PPARγ-dependent and PPARγ-independent manner. As far as it regards JNK inhibitors in the control of cell migration and invasion, it has been reported that JNK2-selective peptide inhibitors inhibited breast cancer cell migration [32] and that JNK suppression inhibited cell migration in human LoVo colon cancer cells [33].

The results obtained in the microarray experiments suggested that the ARHGEF7/β-PIX gene could be an important target for rosiglitazone and AS601245 action. The inhibition of β-PIX expression was confirmed by the RealTime PCR and western blot analysis. Interestingly, β-PIX protein content was decreased, by rosiglitazone and AS601245, in all three lines of colon cancer, suggesting that this effect could be a common feature of rosiglitazone and AS601245 action.

β-PIX protein belongs to a family of cytoplasmic proteins that activate the Ras-like family of Rho proteins by exchanging bound GDP for GTP. It forms a complex with the small GTP binding protein Rac1 and recruits Rac1 to membrane ruffles and to focal adhesions [34]. The small GTPase Rac1 is a well-characterized modulator of cell migration [34]. In addition, the role of β-PIX in cell migration has recently been stressed by the results demonstrating that the restoration of β-PIX expression by genetic manipulation, restored the migratory ability of mesenchymal stromal cells (MDCs) from patients of amyotrophic lateral sclerosis, and the inhibition of β-PIX expression with shRNA, reduced the migration of healthy MSCs [35]. On the basis of these results, we postulated that β-PIX protein could be involved in the rosiglitazone and AS601245 inhibition of cell migration. Indeed, the results obtained by the transfection experiments confirmed the role played by β-PIX protein in this contest, since our data demonstrated, for the first time, that β-PIX transfection completely abrogates the inhibition of colon cancer cell migration caused by rosiglitazone, AS601245 or by combined treatment with both compounds.

Although the treatment with 0.1 µM AS601245 increased the number of PPRE containing genes activated by 50 µM rosiglitazone, the most quantitatively important genes up-modulated by rosiglitazone are the metallothionein genes (MT1X, MT1E, MT1G, MT1H, MT2A) which do not contain PPRE sequences. Metallothionein genes can be induced by anti-inflammatory agents such as dexamethasone [36] and nonsteroidal anti-inflammatory drugs, such as chloroquina, diclofenac and indometacin [37]. Metallothioneins provide protection against metal toxicity [38] and oxidative stress [39]. In cancer cells, metallothionein expressions are increased, decreased or not changed in relation to the cancer types [40]. In particular, a significant decrease in the amount of metallothionein proteins in colorectal adenoma and carcinoma, as compared with normal colorectal mucosa, has been reported [41]. Thus, the increase of metallothionein expression by rosiglitazone may be ascribed to both the anti-neoplastic and anti-inflammatory effects exerted by rosiglitazone in colon cancer cells [42].

Taken together, our data demonstrated, in colon cancer cells, the effectiveness of combined treatments with PPARγ agonists and a JNK inhibitor in reducing cell adhesion and migration, and are in agreement to the data indicating a positive interaction between PPARγ ligands and anti-inflammatory agents in humans [43].

## Supporting Information

Supporting Information S1 **List of genes up-regulated and down-regulated at 24 hours, by 50 µM rosiglitazone, 0.1 µM AS601245 and combined treatment.** Values represent P-values and fold changes versus 1% DMSO-treated CaCo-2 cells.
(DOC)
