# Supplementary material for: Rosiglitazone and AS601245 Decrease Cell Adhesion and Migration through Modulation of Specific Gene Expression in Human Colon Cancer Cells
Source: PLoS One. 2012 Jun 28;7(6):e40149. doi: 10.1371/journal.pone.0040149 (PMC3386191; doi:10.1371/journal.pone.0040149)
Supplement: Supporting Information S1 — List of genes up-regulated and down-regulated at 24 hours, by 50 µM rosiglitazone, 0.1 µM AS601245 and combined treatment. Values represent P-values and fold changes versus 1% DMSO-treated CaCo-2 cells. (DOC) [file pone.0040149.s001.doc]

| S1:AFFIMETRIX ANALYSIS OF GENES MODULATED BY ROSIGLITAZONE, AS601245 AND COMBINED TREATMENT. | | | | | | | | | |
| --- | --- | --- | --- | --- | --- | --- | --- | --- | --- |
| Probe Set ID | Gene Symbol | Entrez Gene Name | Type | p-value Rosi-DMSO | Fold Change Rosi-DMSO | p-value 1245-DMSO | Fold Change 1245-DMSO | p-value Rosi_1245-DMSO | Fold Change Rosi_1245-DMSO |
| 1053_at | RFC2 | replication factor C (activator 1) 2, 40kDa | other |  |  | 4,55E-10 | -1,718 |  |  |
| 1552256_a_at | SCARB1 | scavenger receptor class B, member 1 | transporter |  |  | 2,99E-10 | -1,957 |  |  |
| 1552279_a_at | SLC46A1 | solute carrier family 46 (folate transporter), member 1 | transporter |  |  | 3,47E-13 | -1,759 |  |  |
| 1552310_at | C15ORF40 | chromosome 15 open reading frame 40 | other |  |  | 1,44E-08 | -1,697 | 1,44E-08 | -1,474 |
| 1552344_s_at | CNOT7 | CCR4-NOT transcription complex, subunit 7 | transcription regulator |  |  | 9,11E-09 | -1,885 |  |  |
| 1552617_a_at | RFWD2 | ring finger and WD repeat domain 2 | other |  |  | 9,83E-09 | -1,719 |  |  |
| 1552619_a_at | ANLN | anillin, actin binding protein | other |  |  |  |  | 1,24E-07 | -1,394 |
| 1552627_a_at | ARHGAP5 | Rho GTPase activating protein 5 | enzyme |  |  | 8,70E-08 | -1,760 |  |  |
| 1552658_a_at | NAV3 | neuron navigator 3 | other |  |  |  |  | 2,62E-08 | 2,278 |
| 1552976_at | DNAJB13 | DnaJ (Hsp40) related, subfamily B, member 13 | other | 4,37E-09 | -2,082 | 4,37E-09 | -2,748 |  |  |
| 1552977_a_at | CNPY3 | canopy 3 homolog (zebrafish) | other | 4,02E-09 | -1,557 | 4,02E-09 | -2,002 |  |  |
| 1553113_s_at | CDK8 | cyclin-dependent kinase 8 | kinase | 4,53E-11 | -1,599 | 4,53E-11 | -1,732 | 4,53E-11 | -1,582 |
| 1553530_a_at | ITGB1 | integrin, beta 1 (fibronectin receptor, beta polypeptide, antigen CD29 includes MDF2, MSK12) | transmembrane receptor |  |  | 2,70E-11 | -1,676 |  |  |
| 1553538_s_at | COX1 | cytochrome c oxidase I | enzyme |  |  | 6,37E-08 | -1,404 |  |  |
| 1553587_a_at | POLE4 | polymerase (DNA-directed), epsilon 4 (p12 subunit) | enzyme | 2,55E-10 | -1,471 | 2,55E-10 | -1,707 | 2,55E-10 | -1,530 |
| 1553678_a_at | ITGB1 | integrin, beta 1 (fibronectin receptor, beta polypeptide, antigen CD29 includes MDF2, MSK12) | transmembrane receptor |  |  | 2,09E-08 | -1,459 |  |  |
| 1553749_at | FAM76B | family with sequence similarity 76, member B | other |  |  | 1,83E-14 | 2,363 | 1,83E-14 | 2,098 |
| 1553764_a_at | JUB | jub, ajuba homolog (Xenopus laevis) | other |  |  | 4,59E-09 | -1,475 | 4,59E-09 | -1,580 |
| 1553918_at | C21ORF129 | chromosome 21 open reading frame 129 | other | 1,50E-13 | -2,425 | 1,50E-13 | -1,946 | 1,50E-13 | -2,369 |
| 1553954_at | ALG14 | asparagine-linked glycosylation 14 homolog (S. cerevisiae) | other |  |  | 5,40E-08 | -1,832 |  |  |
| 1553956_at | ALS2CR4 | amyotrophic lateral sclerosis 2 (juvenile) chromosome region, candidate 4 | other |  |  | 8,47E-08 | -1,709 |  |  |
| 1553972_a_at | CBS | cystathionine-beta-synthase | enzyme |  |  | 5,73E-08 | -2,072 |  |  |
| 1553974_at | C22ORF39 | chromosome 22 open reading frame 39 | other |  |  | 3,23E-10 | -1,959 | 3,23E-10 | -1,506 |
| 1553987_at | C12ORF47 | chromosome 12 open reading frame 47 | other | 1,83E-14 | -1,461 | 1,83E-14 | -2,140 | 1,83E-14 | -1,835 |
| 1553990_at | C16ORF79 | chromosome 16 open reading frame 79 | enzyme | 3,95E-09 | -1,786 | 3,95E-09 | -2,900 |  |  |
| 1553993_s_at | MED25 | mediator complex subunit 25 | other |  |  | 3,81E-08 | -1,691 |  |  |
| 1554020_at | BICD1 | bicaudal D homolog 1 (Drosophila) | other | 2,46E-10 | -1,442 | 2,46E-10 | -1,627 |  |  |
| 1554021_a_at | ZNF12 | zinc finger protein 12 | other |  |  | 3,29E-09 | -1,614 |  |  |
| 1554052_at | CNOT1 | CCR4-NOT transcription complex, subunit 1 | other |  |  | 1,81E-07 | -2,102 |  |  |
| 1554078_s_at | DNAJA3 | DnaJ (Hsp40) homolog, subfamily A, member 3 | other |  |  | 5,53E-08 | -1,631 |  |  |
| 1554167_a_at | GOLGA7 | golgi autoantigen, golgin subfamily a, 7 | other | 2,57E-10 | -1,308 | 2,57E-10 | -1,650 | 2,57E-10 | -1,411 |
| 1554241_at | COCH | coagulation factor C homolog, cochlin (Limulus polyphemus) | other |  |  | 1,67E-11 | -1,419 |  |  |
| 1554242_a_at | COCH | coagulation factor C homolog, cochlin (Limulus polyphemus) | other |  |  | 1,99E-12 | -1,864 |  |  |
| 1554341_a_at | HEL308 | DNA helicase HEL308 | enzyme | 1,79E-08 | -1,923 |  |  |  |  |
| 1554345_a_at | GIN1 | gypsy retrotransposon integrase 1 | other | 1,29E-06 | -1,483 |  |  |  |  |
| 1554365_a_at | PPP2R5C | protein phosphatase 2, regulatory subunit B', gamma isoform | peptidase | 1,92E-09 | -1,364 | 1,92E-09 | -1,593 | 1,92E-09 | -1,568 |
| 1554408_a_at | TK1 | thymidine kinase 1, soluble | kinase | 1,83E-14 | -1,462 | 1,83E-14 | -2,152 | 1,83E-14 | -1,452 |
| 1554433_a_at | ZNF146 | zinc finger protein 146 | other |  |  | 4,03E-08 | -1,870 |  |  |
| 1554441_a_at | WAPAL | wings apart-like homolog (Drosophila) | other |  |  | 2,01E-09 | -1,727 |  |  |
| 1554462_a_at | DNAJB9 | DnaJ (Hsp40) homolog, subfamily B, member 9 | other | 1,66E-09 | -1,447 | 1,66E-09 | -1,715 | 1,66E-09 | -1,519 |
| 1554464_a_at | CRTAP | cartilage associated protein | other | 1,17E-11 | -1,612 | 1,17E-11 | -1,684 | 1,17E-11 | -1,762 |
| 1554470_s_at | ZBTB44 | zinc finger and BTB domain containing 44 | other |  |  | 2,72E-08 | -1,549 | 2,72E-08 | -1,394 |
| 1554482_a_at | SAR1B | SAR1 homolog B (S. cerevisiae) | enzyme | 4,41E-11 | -1,390 | 4,41E-11 | -1,619 | 4,41E-11 | -1,319 |
| 1554485_s_at | TMEM37 | transmembrane protein 37 | ion channel |  |  | 8,86E-09 | -2,012 |  |  |
| 1554489_a_at | CEP70 | centrosomal protein 70kDa | other | 3,92E-07 | -1,493 |  |  |  |  |
| 1554574_a_at | CYB5R3 | cytochrome b5 reductase 3 | enzyme | 2,85E-11 | -1,439 | 2,85E-11 | -2,031 | 2,85E-11 | -1,719 |
| 1554679_a_at | LAPTM4B | lysosomal protein transmembrane 4 beta | other |  |  | 2,76E-09 | -1,729 |  |  |
| 1554696_s_at | TYMS | thymidylate synthetase | enzyme |  |  | 3,29E-13 | -1,999 |  |  |
| 1554747_a_at | SEPT2 | septin 2 | enzyme |  |  | 3,42E-09 | -1,697 |  |  |
| 1554794_a_at | UBE3C | ubiquitin protein ligase E3C | enzyme |  |  | 3,33E-09 | -2,407 |  |  |
| 1554868_s_at | PCNP | PEST proteolytic signal containing nuclear protein | other |  |  | 3,51E-08 | -1,597 |  |  |
| 1554878_a_at | ABCD3 | ATP-binding cassette, sub-family D (ALD), member 3 | transporter |  |  | 2,10E-11 | -1,841 |  |  |
| 1554943_at | VIL1 | villin 1 | other | 2,55E-10 | -1,583 | 2,55E-10 | -2,179 | 2,55E-10 | -1,801 |
| 1554945_x_at | VIL1 | villin 1 | other | 1,94E-11 | -1,651 | 1,94E-11 | -2,162 | 1,94E-11 | -1,798 |
| 1554966_a_at | FILIP1L | filamin A interacting protein 1-like | other | 1,98E-10 | -1,774 | 1,98E-10 | -1,716 | 1,98E-10 | -1,641 |
| 1555037_a_at | IDH1 | isocitrate dehydrogenase 1 (NADP+), soluble | enzyme |  |  | 1,19E-08 | -1,466 |  |  |
| 1555058_a_at | LPGAT1 | lysophosphatidylglycerol acyltransferase 1 | other | 1,95E-10 | 1,755 | 1,95E-10 | 1,927 | 1,95E-10 | 2,166 |
| 1555225_at | C1ORF43 | chromosome 1 open reading frame 43 | other |  |  | 3,20E-09 | -1,711 | 3,20E-09 | -1,400 |
| 1555278_a_at | CKAP5 | cytoskeleton associated protein 5 | other |  |  | 1,25E-10 | -1,657 |  |  |
| 1555434_a_at | SLC39A14 | solute carrier family 39 (zinc transporter), member 14 | transporter | 2,06E-13 | -1,431 | 2,06E-13 | -2,024 | 2,06E-13 | -2,261 |
| 1555618_s_at | SAE1 | SUMO1 activating enzyme subunit 1 | enzyme | 1,83E-14 | -1,541 | 1,83E-14 | -2,023 | 1,83E-14 | -1,488 |
| 1555724_s_at | TAGLN | transgelin | other |  |  | 9,26E-09 | -1,708 |  |  |
| 1555731_a_at | AP1S3 (includes EG:130340) | adaptor-related protein complex 1, sigma 3 subunit | transporter | 1,56E-12 | -1,332 | 1,56E-12 | -1,763 | 1,56E-12 | -1,684 |
| 1555733_s_at | AP1S3 (includes EG:130340) | adaptor-related protein complex 1, sigma 3 subunit | transporter |  |  | 2,55E-10 | -1,519 | 2,55E-10 | -1,550 |
| 1555734_x_at | AP1S3 (includes EG:130340) | adaptor-related protein complex 1, sigma 3 subunit | transporter |  |  | 2,79E-09 | -1,623 | 2,79E-09 | -1,546 |
| 1555751_a_at | GEMIN7 | gem (nuclear organelle) associated protein 7 | other |  |  | 1,18E-09 | -2,083 |  |  |
| 1555758_a_at | CDKN3 | cyclin-dependent kinase inhibitor 3 | phosphatase | 1,83E-14 | -1,562 | 1,83E-14 | -2,033 | 1,83E-14 | -1,805 |
| 1555764_s_at | TIMM10 | translocase of inner mitochondrial membrane 10 homolog (yeast) | transporter |  |  | 3,93E-12 | -1,875 |  |  |
| 1555780_a_at | RHEB | Ras homolog enriched in brain | enzyme |  |  | 2,67E-13 | -1,928 |  |  |
| 1555814_a_at | RHOA | ras homolog gene family, member A | enzyme | 8,10E-12 | -1,278 | 8,10E-12 | -1,670 |  |  |
| 1555826_at | EPR1 | effector cell peptidase receptor 1 (non-protein coding) | transmembrane receptor |  |  | 6,26E-10 | -2,205 |  |  |
| 1555832_s_at | KLF6 | Kruppel-like factor 6 | transcription regulator |  |  |  |  | 1,83E-14 | 1,540 |
| 1555841_at | C9ORF30 | chromosome 9 open reading frame 30 | other |  |  | 2,13E-11 | -1,974 |  |  |
| 1555864_s_at | PDHA1 (includes EG:5160) | pyruvate dehydrogenase (lipoamide) alpha 1 | enzyme |  |  | 2,99E-10 | -1,722 | 2,99E-10 | -1,365 |
| 1555889_a_at | CRTAP | cartilage associated protein | other |  |  | 8,24E-09 | -1,600 |  |  |
| 1555920_at | CBX3 | chromobox homolog 3 (HP1 gamma homolog, Drosophila) | other |  |  | 1,96E-11 | -1,914 |  |  |
| 1555948_s_at | FAM120A | family with sequence similarity 120A | other | 1,83E-14 | -1,696 | 1,83E-14 | -2,274 | 1,83E-14 | -1,391 |
| 1556128_a_at | RASGRF2 | Ras protein-specific guanine nucleotide-releasing factor 2 | other | 6,94E-11 | -1,926 | 6,94E-11 | -2,098 |  |  |
| 1556285_s_at | PPA2 | pyrophosphatase (inorganic) 2 | enzyme | 6,26E-14 | -1,280 | 6,26E-14 | -1,853 |  |  |
| 1556499_s_at | COL1A1 | collagen, type I, alpha 1 | other | 3,70E-12 | -1,386 | 3,70E-12 | -1,979 | 3,70E-12 | -1,678 |
| 1556821_x_at | DLEU2 | deleted in lymphocytic leukemia 2 (non-protein coding) | other |  |  | 3,28E-11 | -1,755 | 3,28E-11 | -1,956 |
| 1557053_s_at | UBE2G2 | ubiquitin-conjugating enzyme E2G 2 (UBC7 homolog, yeast) | enzyme |  |  | 1,02E-08 | -1,817 |  |  |
| 1557094_at | LOC728449 | hypothetical protein LOC728449 | other | 1,83E-14 | -4,024 | 1,83E-14 | -5,156 | 1,83E-14 | -6,123 |
| 1557227_s_at | TPR | translocated promoter region (to activated MET oncogene) | other | 7,04E-08 | 1,569 |  |  |  |  |
| 1557411_s_at | SLC25A43 | solute carrier family 25, member 43 | transporter |  |  | 1,33E-07 | -1,643 |  |  |
| 1557613_at | FLJ39534 | hypothetical protein FLJ39534 | other | 3,55E-06 | -3,058 |  |  |  |  |
| 1557910_at | HSP90AB1 | heat shock protein 90kDa alpha (cytosolic), class B member 1 | other |  |  | 3,39E-08 | -1,455 | 3,39E-08 | -1,285 |
| 1557915_s_at | GSTO1 | glutathione S-transferase omega 1 | enzyme |  |  | 1,29E-09 | -1,615 |  |  |
| 1557918_s_at | SLC16A1 | solute carrier family 16, member 1 (monocarboxylic acid transporter 1) | transporter |  |  | 2,18E-08 | -2,204 |  |  |
| 1557954_at | CXORF15 | chromosome X open reading frame 15 | other |  |  | 2,16E-07 | -1,821 |  |  |
| 1558014_s_at | FAR1 | fatty acyl CoA reductase 1 | enzyme |  |  | 2,05E-09 | -1,620 | 2,05E-09 | -1,685 |
| 1558136_s_at | TAF11 | TAF11 RNA polymerase II, TATA box binding protein (TBP)-associated factor, 28kDa | transcription regulator |  |  | 2,17E-08 | -1,670 |  |  |
| 1558214_s_at | CTNNA1 | catenin (cadherin-associated protein), alpha 1, 102kDa | other |  |  | 1,41E-10 | -2,102 |  |  |
| 1558233_s_at | ATF1 | activating transcription factor 1 | transcription regulator |  |  | 4,08E-08 | -1,630 |  |  |
| 1558508_a_at | C1ORF53 | chromosome 1 open reading frame 53 | other |  |  | 2,91E-10 | -2,280 |  |  |
| 1558678_s_at | MALAT1 | metastasis associated lung adenocarcinoma transcript 1 (non-protein coding) | other | 5,77E-12 | 1,414 | 5,77E-12 | 1,482 | 5,77E-12 | 1,646 |
| 1558693_s_at | C1ORF85 | chromosome 1 open reading frame 85 | other |  |  | 3,49E-09 | -1,959 | 3,49E-09 | -1,486 |
| 1558703_at | SLC46A1 | solute carrier family 46 (folate transporter), member 1 | transporter |  |  | 2,03E-10 | -1,617 |  |  |
| 1558747_at | SMCHD1 | structural maintenance of chromosomes flexible hinge domain containing 1 | other | 2,36E-13 | 2,344 | 2,36E-13 | 2,448 | 2,36E-13 | 2,293 |
| 1558956_s_at | IFT80 | intraflagellar transport 80 homolog (Chlamydomonas) | other | 1,30E-08 | -1,384 | 1,30E-08 | -1,472 |  |  |
| 1559132_at | TMEM80 | transmembrane protein 80 | other |  |  | 7,15E-09 | -1,984 | 7,15E-09 | -1,562 |
| 1559946_s_at | RUVBL2 | RuvB-like 2 (E. coli) | transcription regulator |  |  | 1,93E-09 | -1,636 |  |  |
| 1559954_s_at | DDX42 | DEAD (Asp-Glu-Ala-Asp) box polypeptide 42 | enzyme |  |  | 9,99E-09 | -1,877 |  |  |
| 1560019_at | MGC11082 | hypothetical LOC84777 | other |  |  |  |  | 3,72E-13 | 1,984 |
| 1560587_s_at | PRDX5 | peroxiredoxin 5 | enzyme | 1,83E-14 | -1,314 | 1,83E-14 | -1,807 |  |  |
| 1564494_s_at | P4HB | prolyl 4-hydroxylase, beta polypeptide | enzyme | 1,01E-13 | -1,450 | 1,01E-13 | -1,893 | 1,01E-13 | -1,529 |
| 1564651_at | LOC221710 | hypothetical protein LOC221710 | other | 2,31E-09 | -2,023 | 2,31E-09 | -2,388 | 2,31E-09 | -1,873 |
| 1565162_s_at | MGST1 | microsomal glutathione S-transferase 1 | enzyme |  |  | 8,64E-09 | -1,569 |  |  |
| 1565717_s_at | FUS | fusion (involved in t(12;16) in malignant liposarcoma) | transcription regulator |  |  | 1,27E-08 | -1,688 |  |  |
| 1565951_s_at | CHML | choroideremia-like (Rab escort protein 2) | enzyme |  |  |  |  | 6,84E-07 | -1,437 |
| 1567080_s_at | CLN6 | ceroid-lipofuscinosis, neuronal 6, late infantile, variant | other |  |  | 9,33E-09 | -2,005 |  |  |
| 1567458_s_at | RAC1 | ras-related C3 botulinum toxin substrate 1 (rho family, small GTP binding protein Rac1) | enzyme | 4,38E-11 | -1,257 | 4,38E-11 | -1,584 | 4,38E-11 | -1,358 |
| 1568596_a_at | TROAP | trophinin associated protein (tastin) | peptidase |  |  | 1,61E-09 | -2,061 |  |  |
| 1568618_a_at | GALNT1 | UDP-N-acetyl-alpha-D-galactosamine:polypeptide N-acetylgalactosaminyltransferase 1 (GalNAc-T1) | enzyme | 1,83E-14 | -1,676 | 1,83E-14 | -1,559 |  |  |
| 1568619_s_at | ITPRIPL2 | inositol 1,4,5-triphosphate receptor interacting protein-like 2 | other |  |  |  |  | 2,11E-12 | 1,535 |
| 1569253_at | INTS4 | integrator complex subunit 4 | other | 1,08E-09 | -1,549 | 1,08E-09 | -2,043 | 1,08E-09 | -1,746 |
| 1729_at | TRADD | TNFRSF1A-associated via death domain | other |  |  | 1,87E-10 | -1,874 |  |  |
| 1861_at | BAD | BCL2-associated agonist of cell death | other |  |  | 3,20E-08 | -1,698 |  |  |
| 200000_s_at | PRPF8 | PRP8 pre-mRNA processing factor 8 homolog (S. cerevisiae) | other |  |  | 3,02E-09 | -1,610 |  |  |
| 200001_at | CAPNS1 | calpain, small subunit 1 | peptidase | 4,87E-14 | -1,467 | 4,87E-14 | -1,964 | 4,87E-14 | -1,429 |
| 200005_at | EIF3D | eukaryotic translation initiation factor 3, subunit D | translation regulator |  |  | 6,76E-13 | -1,699 |  |  |
| 200011_s_at | ARF3 | ADP-ribosylation factor 3 | enzyme |  |  | 1,21E-10 | -1,693 |  |  |
| 200015_s_at | SEPT2 | septin 2 | enzyme |  |  | 4,75E-08 | -1,589 |  |  |
| 200042_at | C22ORF28 | chromosome 22 open reading frame 28 | other |  |  | 3,12E-09 | -1,674 |  |  |
| 200045_at | ABCF1 | ATP-binding cassette, sub-family F (GCN20), member 1 | transporter |  |  | 1,21E-08 | -1,654 |  |  |
| 200048_s_at | JTB | jumping translocation breakpoint | other |  |  | 1,67E-09 | -1,549 |  |  |
| 200052_s_at | ILF2 (includes EG:3608) | interleukin enhancer binding factor 2, 45kDa | transcription regulator |  |  | 2,02E-10 | -1,590 |  |  |
| 200055_at | TAF10 | TAF10 RNA polymerase II, TATA box binding protein (TBP)-associated factor, 30kDa | transcription regulator |  |  | 2,70E-10 | -1,696 | 2,70E-10 | -1,434 |
| 200058_s_at | SNRNP200 | small nuclear ribonucleoprotein 200kDa (U5) | enzyme |  |  | 1,59E-07 | -1,547 |  |  |
| 200064_at | HSP90AB1 | heat shock protein 90kDa alpha (cytosolic), class B member 1 | other |  |  | 1,08E-08 | -1,707 |  |  |
| 200065_s_at | ARF1 | ADP-ribosylation factor 1 | enzyme |  |  | 1,02E-09 | -1,528 |  |  |
| 200071_at | SMNDC1 | survival motor neuron domain containing 1 | other |  |  | 1,06E-08 | -1,463 |  |  |
| 200072_s_at | HNRNPM | heterogeneous nuclear ribonucleoprotein M | transmembrane receptor |  |  | 2,70E-08 | -1,503 |  |  |
| 200083_at | USP22 | ubiquitin specific peptidase 22 | peptidase | 6,88E-10 | -1,562 | 6,88E-10 | -1,493 |  |  |
| 200084_at | C11ORF58 | chromosome 11 open reading frame 58 | other |  |  | 5,25E-08 | -1,441 |  |  |
| 200085_s_at | TCEB2 | transcription elongation factor B (SIII), polypeptide 2 (18kDa, elongin B) | transcription regulator |  |  | 1,29E-07 | -1,555 |  |  |
| 200090_at | FNTA | farnesyltransferase, CAAX box, alpha | enzyme |  |  | 2,38E-08 | -1,525 |  |  |
| 200093_s_at | HINT1 (includes EG:3094) | histidine triad nucleotide binding protein 1 | enzyme |  |  | 1,77E-09 | -1,599 |  |  |
| 200097_s_at | HNRNPK | heterogeneous nuclear ribonucleoprotein K | other |  |  | 2,00E-09 | -1,605 |  |  |
| 200595_s_at | EIF3A | eukaryotic translation initiation factor 3, subunit A | translation regulator |  |  | 3,67E-10 | -1,526 |  |  |
| 200611_s_at | WDR1 | WD repeat domain 1 | other |  |  | 3,31E-12 | -1,772 |  |  |
| 200613_at | AP2M1 | adaptor-related protein complex 2, mu 1 subunit | transporter | 1,43E-12 | -1,435 | 1,43E-12 | -1,800 | 1,43E-12 | -1,306 |
| 200617_at | MLEC | malectin | other |  |  | 3,44E-11 | -1,712 |  |  |
| 200621_at | CSRP1 | cysteine and glycine-rich protein 1 | other |  |  | 9,09E-12 | -1,894 |  |  |
| 200623_s_at | CALM3 | calmodulin 3 (phosphorylase kinase, delta) | other |  |  | 5,20E-10 | -2,013 |  |  |
| 200624_s_at | MATR3 | matrin 3 | other |  |  | 3,11E-10 | -1,626 |  |  |
| 200625_s_at | CAP1 | CAP, adenylate cyclase-associated protein 1 (yeast) | other | 4,87E-14 | -1,443 | 4,87E-14 | -2,091 | 4,87E-14 | -1,561 |
| 200626_s_at | MATR3 | matrin 3 | other |  |  | 3,04E-08 | -1,517 |  |  |
| 200634_at | PFN1 | profilin 1 | other |  |  | 2,09E-09 | -1,572 |  |  |
| 200636_s_at | PTPRF | protein tyrosine phosphatase, receptor type, F | phosphatase | 6,50E-13 | -1,449 | 6,50E-13 | -1,943 | 6,50E-13 | -1,441 |
| 200640_at | YWHAZ | tyrosine 3-monooxygenase/tryptophan 5-monooxygenase activation protein, zeta polypeptide | enzyme | 3,31E-12 | -1,386 | 3,31E-12 | -1,626 |  |  |
| 200643_at | HDLBP | high density lipoprotein binding protein | transporter |  |  | 9,31E-09 | -1,983 |  |  |
| 200644_at | MARCKSL1 | MARCKS-like 1 | other |  |  | 3,24E-10 | -1,765 |  |  |
| 200645_at | GABARAP | GABA(A) receptor-associated protein | transporter | 1,83E-14 | -1,590 | 1,83E-14 | -1,804 | 1,83E-14 | -1,753 |
| 200656_s_at | P4HB | prolyl 4-hydroxylase, beta polypeptide | enzyme |  |  | 2,39E-10 | -1,712 |  |  |
| 200659_s_at | PHB (includes EG:5245) | prohibitin | transcription regulator |  |  | 3,35E-08 | -1,614 |  |  |
| 200660_at | S100A11 | S100 calcium binding protein A11 | other |  |  | 1,35E-07 | -1,626 |  |  |
| 200661_at | CTSA | cathepsin A | peptidase | 3,47E-13 | -1,440 | 3,47E-13 | -1,826 | 3,47E-13 | -1,541 |
| 200662_s_at | TOMM20 | translocase of outer mitochondrial membrane 20 homolog (yeast) | transporter |  |  | 4,87E-11 | -1,653 |  |  |
| 200668_s_at | UBE2D3 | ubiquitin-conjugating enzyme E2D 3 (UBC4/5 homolog, yeast) | enzyme |  |  | 2,43E-13 | -1,673 |  |  |
| 200681_at | GLO1 | glyoxalase I | enzyme |  |  | 5,85E-08 | -1,517 |  |  |
| 200682_s_at | UBE2L3 | ubiquitin-conjugating enzyme E2L 3 | enzyme |  |  | 9,52E-11 | -1,596 |  |  |
| 200683_s_at | UBE2L3 | ubiquitin-conjugating enzyme E2L 3 | enzyme | 1,47E-11 | -1,478 | 1,47E-11 | -1,782 | 1,47E-11 | -1,358 |
| 200687_s_at | SF3B3 | splicing factor 3b, subunit 3, 130kDa | other |  |  | 3,43E-10 | -1,705 |  |  |
| 200690_at | HSPA9 | heat shock 70kDa protein 9 (mortalin) | other |  |  | 7,75E-12 | -1,850 |  |  |
| 200693_at | YWHAQ (includes EG:10971) | tyrosine 3-monooxygenase/tryptophan 5-monooxygenase activation protein, theta polypeptide | other |  |  | 1,03E-07 | -1,458 |  |  |
| 200694_s_at | DDX24 | DEAD (Asp-Glu-Ala-Asp) box polypeptide 24 | enzyme | 2,25E-13 | -1,541 | 2,25E-13 | -1,828 | 2,25E-13 | -1,434 |
| 200704_at | LITAF | lipopolysaccharide-induced TNF factor | transcription regulator |  |  | 5,99E-12 | -1,949 |  |  |
| 200708_at | GOT2 | glutamic-oxaloacetic transaminase 2, mitochondrial (aspartate aminotransferase 2) | enzyme |  |  | 2,84E-08 | -1,569 |  |  |
| 200709_at | FKBP1A | FK506 binding protein 1A, 12kDa | enzyme |  |  | 5,77E-12 | -1,674 |  |  |
| 200710_at | ACADVL | acyl-Coenzyme A dehydrogenase, very long chain | enzyme |  |  | 9,60E-13 | -1,910 |  |  |
| 200711_s_at | SKP1 | S-phase kinase-associated protein 1 | transcription regulator |  |  | 8,95E-08 | -1,493 |  |  |
| 200714_x_at | OS9 | amplified in osteosarcoma | other | 1,83E-14 | -1,583 | 1,83E-14 | -2,122 | 1,83E-14 | -1,554 |
| 200720_s_at | ACTR1A | ARP1 actin-related protein 1 homolog A, centractin alpha (yeast) | other |  |  | 2,81E-09 | -1,772 |  |  |
| 200722_s_at | CAPRIN1 | cell cycle associated protein 1 | other |  |  | 1,52E-08 | -1,665 |  |  |
| 200734_s_at | ARF3 | ADP-ribosylation factor 3 | enzyme |  |  | 4,84E-09 | -1,647 |  |  |
| 200737_at | PGK1 | phosphoglycerate kinase 1 | kinase | 3,44E-14 | -1,552 | 3,44E-14 | -1,885 | 3,44E-14 | -1,505 |
| 200740_s_at | SUMO3 | SMT3 suppressor of mif two 3 homolog 3 (S. cerevisiae) | other | 3,06E-12 | -1,270 | 3,06E-12 | -1,618 | 3,06E-12 | -1,326 |
| 200742_s_at | TPP1 | tripeptidyl peptidase I | peptidase |  |  | 3,85E-12 | -2,385 |  |  |
| 200743_s_at | TPP1 | tripeptidyl peptidase I | peptidase | 4,41E-13 | -1,744 | 4,41E-13 | -1,674 | 4,41E-13 | -1,521 |
| 200746_s_at | GNB1 | guanine nucleotide binding protein (G protein), beta polypeptide 1 | enzyme |  |  | 9,75E-11 | -1,673 |  |  |
| 200747_s_at | NUMA1 | nuclear mitotic apparatus protein 1 | other |  |  | 1,53E-08 | -2,130 |  |  |
| 200749_at | RAN | RAN, member RAS oncogene family | enzyme | 5,45E-11 | -1,397 | 5,45E-11 | -1,564 |  |  |
| 200750_s_at | RAN | RAN, member RAS oncogene family | enzyme |  |  | 8,98E-08 | -1,446 |  |  |
| 200751_s_at | HNRNPC | heterogeneous nuclear ribonucleoprotein C (C1/C2) | other |  |  | 7,48E-10 | -1,674 |  |  |
| 200752_s_at | CAPN1 | calpain 1, (mu/I) large subunit | peptidase |  |  | 1,61E-09 | -2,686 |  |  |
| 200753_x_at | SFRS2 | splicing factor, arginine/serine-rich 2 | transcription regulator | 3,15E-12 | -1,378 | 3,15E-12 | -1,670 | 3,15E-12 | -1,437 |
| 200757_s_at | CALU | calumenin | other |  |  | 6,43E-09 | -1,610 |  |  |
| 200762_at | DPYSL2 | dihydropyrimidinase-like 2 | enzyme |  |  | 1,53E-11 | -1,573 |  |  |
| 200774_at | FAM120A | family with sequence similarity 120A | other | 1,82E-11 | -1,389 | 1,82E-11 | -1,602 |  |  |
| 200778_s_at | SEPT2 | septin 2 | enzyme |  |  | 3,07E-11 | -1,696 | 3,07E-11 | -1,305 |
| 200783_s_at | STMN1 | stathmin 1/oncoprotein 18 | other |  |  |  |  | 3,22E-07 | -1,501 |
| 200787_s_at | PEA15 | phosphoprotein enriched in astrocytes 15 | transporter |  |  | 6,76E-13 | -2,037 |  |  |
| 200788_s_at | PEA15 | phosphoprotein enriched in astrocytes 15 | transporter |  |  | 4,45E-11 | -1,681 |  |  |
| 200791_s_at | IQGAP1 | IQ motif containing GTPase activating protein 1 | other | 1,39E-13 | -1,536 | 1,39E-13 | -1,831 |  |  |
| 200793_s_at | ACO2 | aconitase 2, mitochondrial | enzyme |  |  | 2,17E-09 | -1,781 |  |  |
| 200794_x_at | DAZAP2 (includes EG:9802) | DAZ associated protein 2 | other | 2,21E-09 | -1,472 | 2,21E-09 | -1,399 | 2,21E-09 | -1,394 |
| 200802_at | SARS | seryl-tRNA synthetase | enzyme |  |  | 8,88E-10 | -1,794 |  |  |
| 200805_at | LMAN2 | lectin, mannose-binding 2 | transporter |  |  | 3,26E-08 | -1,602 |  |  |
| 200810_s_at | CIRBP | cold inducible RNA binding protein | other |  |  | 5,45E-10 | -1,737 |  |  |
| 200811_at | CIRBP | cold inducible RNA binding protein | other | 1,13E-11 | -1,496 | 1,13E-11 | -1,786 | 1,13E-11 | -1,458 |
| 200820_at | PSMD8 | proteasome (prosome, macropain) 26S subunit, non-ATPase, 8 | other |  |  | 2,13E-08 | -1,675 |  |  |
| 200822_x_at | TPI1 | triosephosphate isomerase 1 | enzyme |  |  | 1,55E-08 | -1,570 |  |  |
| 200828_s_at | ZNF207 | zinc finger protein 207 | transcription regulator |  |  | 2,75E-07 | -1,527 |  |  |
| 200831_s_at | SCD | stearoyl-CoA desaturase (delta-9-desaturase) | enzyme |  |  | 2,01E-11 | -1,747 |  |  |
| 200838_at | CTSB | cathepsin B | peptidase | 7,56E-12 | -1,472 | 7,56E-12 | -1,757 | 7,56E-12 | -1,314 |
| 200843_s_at | EPRS | glutamyl-prolyl-tRNA synthetase | enzyme |  |  | 7,86E-08 | -1,631 |  |  |
| 200846_s_at | PPP1CA | protein phosphatase 1, catalytic subunit, alpha isoform | phosphatase |  |  | 2,32E-08 | -1,609 |  |  |
| 200850_s_at | AHCYL1 | S-adenosylhomocysteine hydrolase-like 1 | enzyme |  |  | 1,27E-09 | -1,606 |  |  |
| 200852_x_at | GNB2 | guanine nucleotide binding protein (G protein), beta polypeptide 2 | enzyme |  |  | 1,28E-10 | -1,875 |  |  |
| 200862_at | DHCR24 | 24-dehydrocholesterol reductase | enzyme |  |  | 4,94E-12 | -1,780 | 4,94E-12 | -1,318 |
| 200866_s_at | PSAP | prosaposin | other | 2,49E-12 | -1,480 | 2,49E-12 | -1,827 |  |  |
| 200867_at | RNF114 | ring finger protein 114 | other |  |  | 2,18E-08 | -1,518 |  |  |
| 200870_at | STRAP | serine/threonine kinase receptor associated protein | other |  |  | 1,33E-07 | -1,591 |  |  |
| 200880_at | DNAJA1 | DnaJ (Hsp40) homolog, subfamily A, member 1 | other | 4,32E-13 | -1,508 | 4,32E-13 | -1,670 |  |  |
| 200882_s_at | PSMD4 | proteasome (prosome, macropain) 26S subunit, non-ATPase, 4 | other |  |  | 3,51E-12 | -1,695 |  |  |
| 200883_at | UQCRC2 | ubiquinol-cytochrome c reductase core protein II | enzyme |  |  | 4,79E-09 | -1,545 |  |  |
| 200885_at | RHOC | ras homolog gene family, member C | enzyme |  |  | 5,48E-11 | -1,789 |  |  |
| 200893_at | SFRS10 | splicing factor, arginine/serine-rich 10 (transformer 2 homolog, Drosophila) | other |  |  | 2,00E-07 | -1,436 |  |  |
| 200894_s_at | FKBP4 | FK506 binding protein 4, 59kDa | enzyme |  |  | 2,31E-11 | -1,949 |  |  |
| 200896_x_at | HDGF | hepatoma-derived growth factor (high-mobility group protein 1-like) | growth factor |  |  | 6,14E-09 | -1,674 |  |  |
| 200899_s_at | MGEA5 | meningioma expressed antigen 5 (hyaluronidase) | enzyme | 3,21E-09 | -1,485 | 3,21E-09 | -1,419 |  |  |
| 200901_s_at | M6PR | mannose-6-phosphate receptor (cation dependent) | transporter |  |  | 5,05E-10 | -1,624 |  |  |
| 200902_at | SEP15 | 15 kDa selenoprotein | enzyme | 1,83E-14 | -1,561 | 1,83E-14 | -1,764 | 1,83E-14 | -1,416 |
| 200903_s_at | AHCY | S-adenosylhomocysteine hydrolase | enzyme |  |  | 1,58E-09 | -1,690 |  |  |
| 200905_x_at | HLA-E | major histocompatibility complex, class I, E | transmembrane receptor | 5,93E-12 | -1,546 | 5,93E-12 | -1,896 |  |  |
| 200911_s_at | TACC1 | transforming, acidic coiled-coil containing protein 1 | other |  |  | 1,83E-14 | -2,202 | 1,83E-14 | -1,589 |
| 200916_at | TAGLN2 | transgelin 2 | other | 1,05E-11 | -1,385 | 1,05E-11 | -1,825 |  |  |
| 200921_s_at | BTG1 | B-cell translocation gene 1, anti-proliferative | transcription regulator | 1,83E-14 | -2,119 | 1,83E-14 | -2,006 | 1,83E-14 | -1,910 |
| 200923_at | LGALS3BP | lectin, galactoside-binding, soluble, 3 binding protein | transmembrane receptor | 1,83E-14 | -1,642 | 1,83E-14 | -2,673 | 1,83E-14 | -1,728 |
| 200929_at | TMED10 | transmembrane emp24-like trafficking protein 10 (yeast) | transporter | 2,05E-09 | -1,324 | 2,05E-09 | -1,506 | 2,05E-09 | -1,449 |
| 200931_s_at | VCL | vinculin | enzyme | 1,29E-11 | -1,357 | 1,29E-11 | -1,591 |  |  |
| 200940_s_at | RERE | arginine-glutamic acid dipeptide (RE) repeats | transcription regulator | 2,62E-11 | -1,680 | 2,62E-11 | -1,992 |  |  |
| 200941_at | HSBP1 | heat shock factor binding protein 1 | transcription regulator | 9,02E-11 | -1,438 | 9,02E-11 | -1,670 |  |  |
| 200945_s_at | SEC31A | SEC31 homolog A (S. cerevisiae) | other | 1,80E-12 | -1,357 | 1,80E-12 | -1,780 | 1,80E-12 | -1,393 |
| 200947_s_at | GLUD1 | glutamate dehydrogenase 1 | enzyme | 2,17E-07 | -1,346 |  |  |  |  |
| 200948_at | MLF2 | myeloid leukemia factor 2 | other | 1,83E-14 | -1,427 | 1,83E-14 | -2,103 |  |  |
| 200950_at | ARPC1A | actin related protein 2/3 complex, subunit 1A, 41kDa | other |  |  | 2,66E-09 | -1,503 |  |  |
| 200957_s_at | SSRP1 | structure specific recognition protein 1 | other |  |  | 1,56E-09 | -1,726 |  |  |
| 200959_at | FUS | fusion (involved in t(12;16) in malignant liposarcoma) | transcription regulator |  |  | 1,12E-08 | -1,631 |  |  |
| 200964_at | UBA1 | ubiquitin-like modifier activating enzyme 1 | enzyme |  |  | 9,01E-08 | -1,635 |  |  |
| 200969_at | SERP1 | stress-associated endoplasmic reticulum protein 1 | other |  |  | 4,38E-08 | -1,488 |  |  |
| 200972_at | TSPAN3 | tetraspanin 3 | other |  |  | 9,58E-08 | -1,484 |  |  |
| 200975_at | PPT1 | palmitoyl-protein thioesterase 1 | enzyme | 1,18E-09 | -1,514 | 1,18E-09 | -1,425 |  |  |
| 200977_s_at | TAX1BP1 | Tax1 (human T-cell leukemia virus type I) binding protein 1 | other |  |  | 7,07E-09 | -1,494 |  |  |
| 200978_at | MDH1 | malate dehydrogenase 1, NAD (soluble) | enzyme |  |  | 5,84E-08 | -1,470 |  |  |
| 200980_s_at | PDHA1 (includes EG:5160) | pyruvate dehydrogenase (lipoamide) alpha 1 | enzyme |  |  | 5,25E-08 | -1,732 |  |  |
| 200984_s_at | CD59 | CD59 molecule, complement regulatory protein | other | 7,97E-10 | -1,471 | 7,97E-10 | -1,561 |  |  |
| 200990_at | TRIM28 | tripartite motif-containing 28 | transcription regulator |  |  | 3,53E-08 | -1,790 |  |  |
| 200993_at | IPO7 | importin 7 | transporter |  |  | 3,17E-10 | -1,462 |  |  |
| 200995_at | IPO7 | importin 7 | transporter |  |  | 1,03E-10 | -1,549 |  |  |
| 201000_at | AARS | alanyl-tRNA synthetase | enzyme |  |  | 4,53E-10 | -1,837 |  |  |
| 201008_s_at | TXNIP | thioredoxin interacting protein | other | 1,83E-14 | -2,119 |  |  | 1,83E-14 | -2,276 |
| 201009_s_at | TXNIP | thioredoxin interacting protein | other | 1,83E-14 | -2,364 | 1,83E-14 | -1,470 | 1,83E-14 | -2,435 |
| 201010_s_at | TXNIP | thioredoxin interacting protein | other | 1,83E-14 | -2,204 | 1,83E-14 | -1,585 | 1,83E-14 | -2,532 |
| 201012_at | ANXA1 | annexin A1 | other |  |  | 7,55E-14 | -1,645 |  |  |
| 201015_s_at | JUP | junction plakoglobin | other |  |  | 1,73E-10 | -1,706 |  |  |
| 201019_s_at | EIF1AX | eukaryotic translation initiation factor 1A, X-linked | translation regulator |  |  | 8,92E-08 | -1,467 |  |  |
| 201021_s_at | DSTN | destrin (actin depolymerizing factor) | other |  |  | 1,27E-08 | -1,449 | 1,27E-08 | -1,286 |
| 201023_at | TAF7 | TAF7 RNA polymerase II, TATA box binding protein (TBP)-associated factor, 55kDa | transcription regulator | 8,23E-10 | -1,395 | 8,23E-10 | -1,474 |  |  |
| 201025_at | EIF5B | eukaryotic translation initiation factor 5B | translation regulator | 3,44E-14 | -1,679 | 3,44E-14 | -2,038 | 3,44E-14 | -1,531 |
| 201027_s_at | EIF5B | eukaryotic translation initiation factor 5B | translation regulator |  |  | 7,55E-14 | -1,865 |  |  |
| 201028_s_at | CD99 (includes EG:4267) | CD99 molecule | other |  |  | 1,23E-09 | -1,602 |  |  |
| 201032_at | BLCAP | bladder cancer associated protein | other |  |  | 1,39E-08 | -1,645 |  |  |
| 201036_s_at | HADH | hydroxyacyl-Coenzyme A dehydrogenase | enzyme |  |  | 5,81E-08 | -1,663 |  |  |
| 201042_at | TGM2 | transglutaminase 2 (C polypeptide, protein-glutamine-gamma-glutamyltransferase) | enzyme | 1,83E-14 | -1,853 | 1,83E-14 | -2,778 | 1,83E-14 | -2,293 |
| 201053_s_at | PSMF1 | proteasome (prosome, macropain) inhibitor subunit 1 (PI31) | other |  |  | 2,60E-10 | -1,914 |  |  |
| 201054_at | HNRNPA0 | heterogeneous nuclear ribonucleoprotein A0 | other |  |  | 7,20E-08 | -1,454 |  |  |
| 201055_s_at | HNRNPA0 | heterogeneous nuclear ribonucleoprotein A0 | other | 2,46E-10 | 1,857 |  |  |  |  |
| 201058_s_at | MYL9 (includes EG:10398) | myosin, light chain 9, regulatory | other |  |  | 6,07E-13 | -1,965 |  |  |
| 201059_at | CTTN | cortactin | other |  |  | 3,19E-10 | -1,641 |  |  |
| 201061_s_at | STOM | stomatin | other | 6,50E-10 | -1,470 | 6,50E-10 | -1,948 |  |  |
| 201066_at | CYC1 | cytochrome c-1 | enzyme |  |  | 2,41E-09 | -1,717 |  |  |
| 201072_s_at | SMARCC1 | SWI/SNF related, matrix associated, actin dependent regulator of chromatin, subfamily c, member 1 | transcription regulator |  |  | 1,83E-14 | 2,020 | 1,83E-14 | 2,557 |
| 201074_at | SMARCC1 | SWI/SNF related, matrix associated, actin dependent regulator of chromatin, subfamily c, member 1 | transcription regulator |  |  | 9,39E-09 | -1,594 |  |  |
| 201075_s_at | SMARCC1 | SWI/SNF related, matrix associated, actin dependent regulator of chromatin, subfamily c, member 1 | transcription regulator | 7,87E-12 | -1,470 | 7,87E-12 | -1,890 |  |  |
| 201077_s_at | NHP2L1 | NHP2 non-histone chromosome protein 2-like 1 (S. cerevisiae) | other |  |  | 8,59E-09 | -1,588 |  |  |
| 201079_at | SYNGR2 | synaptogyrin 2 | other |  |  | 1,74E-08 | -1,721 |  |  |
| 201082_s_at | DCTN1 | dynactin 1 (p150, glued homolog, Drosophila) | other |  |  | 1,07E-10 | -1,872 |  |  |
| 201084_s_at | BCLAF1 | BCL2-associated transcription factor 1 | transcription regulator |  |  | 4,13E-10 | -1,492 |  |  |
| 201095_at | DAP | death-associated protein | other |  |  |  |  | 1,04E-07 | -1,465 |
| 201097_s_at | ARF4 | ADP-ribosylation factor 4 | enzyme |  |  | 1,15E-08 | -1,481 |  |  |
| 201099_at | USP9X | ubiquitin specific peptidase 9, X-linked | peptidase |  |  | 5,09E-11 | -1,795 |  |  |
| 201115_at | POLD2 | polymerase (DNA directed), delta 2, regulatory subunit 50kDa | enzyme |  |  | 1,73E-10 | -1,893 |  |  |
| 201118_at | PGD | phosphogluconate dehydrogenase | enzyme |  |  | 7,33E-12 | -1,866 |  |  |
| 201126_s_at | MGAT1 | mannosyl (alpha-1,3-)-glycoprotein beta-1,2-N-acetylglucosaminyltransferase | enzyme |  |  | 1,35E-09 | -2,293 |  |  |
| 201131_s_at | CDH1 | cadherin 1, type 1, E-cadherin (epithelial) | other | 8,87E-10 | -1,404 | 8,87E-10 | -1,551 |  |  |
| 201135_at | ECHS1 | enoyl Coenzyme A hydratase, short chain, 1, mitochondrial | enzyme |  |  | 1,10E-09 | -1,573 |  |  |
| 201136_at | PLP2 | proteolipid protein 2 (colonic epithelium-enriched) | transporter |  |  | 3,92E-08 | -1,658 |  |  |
| 201139_s_at | SSB | Sjogren syndrome antigen B (autoantigen La) | enzyme |  |  | 1,89E-09 | -1,516 |  |  |
| 201140_s_at | RAB5C | RAB5C, member RAS oncogene family | enzyme |  |  | 7,86E-11 | -1,898 |  |  |
| 201142_at | EIF2S1 | eukaryotic translation initiation factor 2, subunit 1 alpha, 35kDa | translation regulator |  |  | 1,47E-11 | -1,906 |  |  |
| 201145_at | HAX1 | HCLS1 associated protein X-1 | other |  |  | 9,17E-08 | -1,580 |  |  |
| 201155_s_at | MFN2 | mitofusin 2 | enzyme | 1,15E-11 | -1,607 | 1,15E-11 | -2,172 |  |  |
| 201156_s_at | RAB5C | RAB5C, member RAS oncogene family | enzyme |  |  | 7,73E-11 | -1,828 |  |  |
| 201157_s_at | NMT1 | N-myristoyltransferase 1 | enzyme | 1,83E-14 | -1,386 | 1,83E-14 | -1,856 | 1,83E-14 | -1,285 |
| 201175_at | TXNDC14 | thioredoxin domain containing 14 | enzyme |  |  | 4,14E-08 | -1,546 |  |  |
| 201176_s_at | ARCN1 | archain 1 | other |  |  | 7,34E-09 | -1,573 |  |  |
| 201183_s_at | CHD4 | chromodomain helicase DNA binding protein 4 | enzyme |  |  |  |  | 4,82E-08 | 1,692 |
| 201189_s_at | ITPR3 | inositol 1,4,5-triphosphate receptor, type 3 | ion channel |  |  | 1,14E-11 | -2,012 |  |  |
| 201190_s_at | PITPNA | phosphatidylinositol transfer protein, alpha | transporter | 1,20E-10 | -1,481 | 1,20E-10 | -1,768 |  |  |
| 201198_s_at | PSMD1 | proteasome (prosome, macropain) 26S subunit, non-ATPase, 1 | other | 4,67E-12 | -1,343 | 4,67E-12 | -1,581 |  |  |
| 201204_s_at | RRBP1 | ribosome binding protein 1 homolog 180kDa (dog) | transporter | 1,83E-14 | -1,555 | 1,83E-14 | -2,120 |  |  |
| 201207_at | TNFAIP1 | tumor necrosis factor, alpha-induced protein 1 (endothelial) | ion channel |  |  | 3,80E-09 | -1,508 |  |  |
| 201209_at | HDAC1 | histone deacetylase 1 | transcription regulator |  |  | 5,39E-08 | -1,511 |  |  |
| 201210_at | DDX3X | DEAD (Asp-Glu-Ala-Asp) box polypeptide 3, X-linked | enzyme | 3,20E-12 | -1,364 | 3,20E-12 | -1,540 |  |  |
| 201212_at | LGMN | legumain | peptidase |  |  | 1,88E-09 | -1,903 |  |  |
| 201215_at | PLS3 | plastin 3 (T isoform) | other | 1,24E-10 | -1,410 | 1,24E-10 | -1,440 | 1,24E-10 | -1,365 |
| 201216_at | ERP29 | endoplasmic reticulum protein 29 | transporter |  |  |  |  | 9,56E-08 | -1,455 |
| 201221_s_at | SNRNP70 | small nuclear ribonucleoprotein 70kDa (U1) | other |  |  | 4,48E-10 | -2,128 |  |  |
| 201223_s_at | RAD23B | RAD23 homolog B (S. cerevisiae) | other |  |  | 2,67E-07 | -1,490 |  |  |
| 201225_s_at | SRRM1 | serine/arginine repetitive matrix 1 | other |  |  | 1,74E-08 | -1,535 |  |  |
| 201233_at | PSMD13 | proteasome (prosome, macropain) 26S subunit, non-ATPase, 13 | peptidase |  |  | 1,74E-08 | -1,925 |  |  |
| 201243_s_at | ATP1B1 | ATPase, Na+/K+ transporting, beta 1 polypeptide | transporter | 2,72E-09 | -1,433 | 2,72E-09 | -1,505 |  |  |
| 201244_s_at | RAF1 | v-raf-1 murine leukemia viral oncogene homolog 1 | kinase |  |  | 8,21E-08 | -1,607 |  |  |
| 201256_at | COX7A2L | cytochrome c oxidase subunit VIIa polypeptide 2 like | enzyme |  |  | 5,84E-08 | -1,550 |  |  |
| 201259_s_at | SYPL1 | synaptophysin-like 1 | transporter |  |  | 1,95E-08 | -1,527 |  |  |
| 201260_s_at | SYPL1 | synaptophysin-like 1 | transporter |  |  |  |  | 1,58E-07 | -1,329 |
| 201267_s_at | PSMC3 | proteasome (prosome, macropain) 26S subunit, ATPase, 3 | transcription regulator |  |  | 1,29E-09 | -1,604 |  |  |
| 201271_s_at | RALY | RNA binding protein, autoantigenic (hnRNP-associated with lethal yellow homolog (mouse)) | other |  |  | 7,31E-11 | -1,741 | 7,31E-11 | -1,429 |
| 201272_at | AKR1B1 | aldo-keto reductase family 1, member B1 (aldose reductase) | enzyme | 3,85E-12 | -1,462 | 3,85E-12 | -1,814 |  |  |
| 201273_s_at | SRP9 | signal recognition particle 9kDa | other |  |  | 1,76E-09 | -1,501 | 1,76E-09 | -1,266 |
| 201278_at | DAB2 | disabled homolog 2, mitogen-responsive phosphoprotein (Drosophila) | other | 1,83E-14 | -1,697 | 1,83E-14 | -1,724 | 1,83E-14 | -1,672 |
| 201279_s_at | DAB2 | disabled homolog 2, mitogen-responsive phosphoprotein (Drosophila) | other |  |  | 5,10E-08 | -1,725 |  |  |
| 201280_s_at | DAB2 | disabled homolog 2, mitogen-responsive phosphoprotein (Drosophila) | other | 1,41E-11 | -1,492 | 1,41E-11 | -1,580 | 1,41E-11 | -1,560 |
| 201281_at | ADRM1 | adhesion regulating molecule 1 | other |  |  | 1,12E-10 | -1,664 |  |  |
| 201284_s_at | APEH | N-acylaminoacyl-peptide hydrolase | peptidase |  |  | 1,97E-10 | -1,805 |  |  |
| 201285_at | MKRN1 | makorin ring finger protein 1 | other |  |  | 1,95E-08 | -1,671 |  |  |
| 201287_s_at | SDC1 | syndecan 1 | other |  |  | 5,74E-12 | -2,440 |  |  |
| 201288_at | ARHGDIB | Rho GDP dissociation inhibitor (GDI) beta | other | 2,25E-09 | -1,699 | 2,25E-09 | -2,065 | 2,25E-09 | -1,754 |
| 201289_at | CYR61 | cysteine-rich, angiogenic inducer, 61 | other | 2,02E-09 | -1,546 | 2,02E-09 | -1,493 |  |  |
| 201290_at | SEC11A | SEC11 homolog A (S. cerevisiae) | peptidase |  |  | 6,12E-08 | -1,446 |  |  |
| 201292_at | TOP2A | topoisomerase (DNA) II alpha 170kDa | enzyme | 1,83E-14 | -1,740 | 1,83E-14 | -1,966 | 1,83E-14 | -1,740 |
| 201296_s_at | WSB1 | WD repeat and SOCS box-containing 1 | other | 1,14E-13 | -1,519 | 1,14E-13 | -1,769 | 1,14E-13 | -1,380 |
| 201301_s_at | ANXA4 | annexin A4 | other |  |  | 1,06E-09 | -1,514 | 1,06E-09 | -1,442 |
| 201302_at | ANXA4 | annexin A4 | other | 2,61E-11 | -1,315 | 2,61E-11 | -1,592 | 2,61E-11 | -1,364 |
| 201304_at | NDUFA5 | NADH dehydrogenase (ubiquinone) 1 alpha subcomplex, 5, 13kDa | enzyme | 3,06E-10 | -1,478 | 3,06E-10 | -1,614 | 3,06E-10 | -1,378 |
| 201307_at | SEPT11 | septin 11 | other |  |  | 1,70E-09 | -1,601 |  |  |
| 201316_at | PSMA2 | proteasome (prosome, macropain) subunit, alpha type, 2 | peptidase | 1,23E-10 | -1,525 | 1,23E-10 | -1,712 | 1,23E-10 | -1,370 |
| 201337_s_at | VAMP3 | vesicle-associated membrane protein 3 (cellubrevin) | other | 2,93E-11 | -1,416 | 2,93E-11 | -1,948 | 2,93E-11 | -1,572 |
| 201338_x_at | GTF3A | general transcription factor IIIA | transcription regulator |  |  | 2,18E-08 | -1,625 |  |  |
| 201341_at | ENC1 | ectodermal-neural cortex (with BTB-like domain) | peptidase | 2,96E-08 | -1,527 |  |  | 2,96E-08 | -1,391 |
| 201345_s_at | UBE2D2 | ubiquitin-conjugating enzyme E2D 2 (UBC4/5 homolog, yeast) | enzyme |  |  | 2,47E-07 | -1,556 |  |  |
| 201347_x_at | GRHPR | glyoxylate reductase/hydroxypyruvate reductase | enzyme |  |  | 8,70E-09 | -1,836 |  |  |
| 201348_at | GPX3 | glutathione peroxidase 3 (plasma) | enzyme | 7,05E-10 | -1,403 | 7,05E-10 | -1,551 |  |  |
| 201350_at | FLOT2 | flotillin 2 | other |  |  | 9,47E-11 | -1,942 |  |  |
| 201360_at | CST3 | cystatin C | other | 8,26E-13 | -1,300 | 8,26E-13 | -1,811 |  |  |
| 201376_s_at | HNRNPF | heterogeneous nuclear ribonucleoprotein F | other |  |  | 4,03E-08 | -1,583 |  |  |
| 201377_at | UBAP2L | ubiquitin associated protein 2-like | other | 1,74E-10 | -1,353 | 1,74E-10 | -1,764 | 1,74E-10 | -1,463 |
| 201378_s_at | UBAP2L | ubiquitin associated protein 2-like | other |  |  | 8,44E-09 | -1,809 |  |  |
| 201379_s_at | TPD52L2 | tumor protein D52-like 2 | other |  |  | 8,88E-14 | -1,906 |  |  |
| 201384_s_at | NBR1 | neighbor of BRCA1 gene 1 | other | 4,39E-07 | -1,452 |  |  |  |  |
| 201388_at | PSMD3 | proteasome (prosome, macropain) 26S subunit, non-ATPase, 3 | other |  |  | 9,90E-11 | -2,019 |  |  |
| 201390_s_at | LY6G5B | lymphocyte antigen 6 complex, locus G5B | other |  |  | 4,42E-08 | -1,593 |  |  |
| 201391_at | TRAP1 | TNF receptor-associated protein 1 | enzyme |  |  | 2,78E-08 | -1,640 |  |  |
| 201392_s_at | IGF2R | insulin-like growth factor 2 receptor | transmembrane receptor |  |  | 1,26E-09 | -1,815 |  |  |
| 201393_s_at | IGF2R | insulin-like growth factor 2 receptor | transmembrane receptor |  |  | 9,00E-10 | -1,585 |  |  |
| 201399_s_at | TRAM1 | translocation associated membrane protein 1 | other | 9,87E-09 | -1,340 | 9,87E-09 | -1,434 |  |  |
| 201405_s_at | COPS6 | COP9 constitutive photomorphogenic homolog subunit 6 (Arabidopsis) | other |  |  | 1,40E-09 | -1,693 | 1,40E-09 | -1,346 |
| 201415_at | GSS | glutathione synthetase | enzyme |  |  | 4,40E-12 | -1,732 |  |  |
| 201427_s_at | SEPP1 | selenoprotein P, plasma, 1 | other | 1,83E-14 | -1,888 | 1,83E-14 | -1,460 | 1,83E-14 | -2,035 |
| 201430_s_at | DPYSL3 | dihydropyrimidinase-like 3 | enzyme | 1,83E-14 | -2,133 | 1,83E-14 | -2,048 | 1,83E-14 | -2,597 |
| 201431_s_at | DPYSL3 | dihydropyrimidinase-like 3 | enzyme | 7,55E-14 | -1,911 | 7,55E-14 | -1,691 | 7,55E-14 | -1,768 |
| 201433_s_at | PTDSS1 | phosphatidylserine synthase 1 | enzyme |  |  | 2,29E-11 | -1,720 |  |  |
| 201435_s_at | EIF4E | eukaryotic translation initiation factor 4E | translation regulator | 2,74E-08 | -1,371 | 2,74E-08 | -1,504 |  |  |
| 201440_at | DDX23 | DEAD (Asp-Glu-Ala-Asp) box polypeptide 23 | enzyme |  |  | 2,90E-11 | -2,035 |  |  |
| 201443_s_at | ATP6AP2 | ATPase, H+ transporting, lysosomal accessory protein 2 | transporter | 6,57E-09 | -1,360 | 6,57E-09 | -1,454 |  |  |
| 201455_s_at | NPEPPS | aminopeptidase puromycin sensitive | peptidase |  |  | 6,41E-10 | -1,871 |  |  |
| 201458_s_at | BUB3 | budding uninhibited by benzimidazoles 3 homolog (yeast) | other | 8,55E-11 | -1,435 | 8,55E-11 | -1,562 |  |  |
| 201463_s_at | TALDO1 | transaldolase 1 | enzyme |  |  | 8,55E-08 | -1,544 |  |  |
| 201472_at | VBP1 | von Hippel-Lindau binding protein 1 | other |  |  | 3,93E-08 | -1,465 |  |  |
| 201475_x_at | MARS | methionyl-tRNA synthetase | enzyme |  |  | 1,91E-09 | -1,629 |  |  |
| 201477_s_at | RRM1 | ribonucleotide reductase M1 | enzyme | 4,87E-14 | -1,433 | 4,87E-14 | -1,677 | 4,87E-14 | -1,383 |
| 201478_s_at | DKC1 | dyskeratosis congenita 1, dyskerin | enzyme |  |  | 2,06E-13 | -1,878 |  |  |
| 201479_at | DKC1 | dyskeratosis congenita 1, dyskerin | enzyme |  |  | 1,01E-13 | -1,734 |  |  |
| 201480_s_at | SUPT5H | suppressor of Ty 5 homolog (S. cerevisiae) | transcription regulator |  |  | 2,65E-08 | -1,984 |  |  |
| 201485_s_at | RCN2 | reticulocalbin 2, EF-hand calcium binding domain | other |  |  | 5,60E-09 | -1,656 |  |  |
| 201489_at | PPIF | peptidylprolyl isomerase F | enzyme |  |  | 2,23E-08 | -1,521 |  |  |
| 201493_s_at | PUM2 | pumilio homolog 2 (Drosophila) | other |  |  |  |  | 3,92E-07 | -1,359 |
| 201500_s_at | PPP1R11 | protein phosphatase 1, regulatory (inhibitor) subunit 11 | other |  |  | 3,54E-11 | -1,820 |  |  |
| 201503_at | G3BP1 | GTPase activating protein (SH3 domain) binding protein 1 | enzyme |  |  | 6,59E-11 | -1,637 |  |  |
| 201504_s_at | TSN | translin | other |  |  | 6,27E-07 | -1,346 |  |  |
| 201507_at | PFDN1 | prefoldin subunit 1 | transcription regulator |  |  | 1,02E-07 | -1,787 |  |  |
| 201509_at | IDH3B | isocitrate dehydrogenase 3 (NAD+) beta | enzyme |  |  | 5,50E-09 | -1,761 |  |  |
| 201511_at | AAMP | angio-associated, migratory cell protein | other |  |  | 4,00E-12 | -2,225 |  |  |
| 201512_s_at | TOMM70A | translocase of outer mitochondrial membrane 70 homolog A (S. cerevisiae) | other |  |  | 2,04E-12 | -1,925 |  |  |
| 201513_at | TSN | translin | other | 2,19E-12 | -1,903 | 2,19E-12 | -1,792 | 2,19E-12 | -1,634 |
| 201518_at | CBX1 | chromobox homolog 1 (HP1 beta homolog Drosophila ) | other | 9,70E-11 | -1,401 | 9,70E-11 | -1,561 | 9,70E-11 | -1,313 |
| 201520_s_at | GRSF1 | G-rich RNA sequence binding factor 1 | other |  |  | 1,01E-12 | -1,834 |  |  |
| 201522_x_at | SNRPN | small nuclear ribonucleoprotein polypeptide N | other |  |  | 2,14E-07 | -1,528 |  |  |
| 201527_at | ATP6V1F | ATPase, H+ transporting, lysosomal 14kDa, V1 subunit F | transporter |  |  | 6,95E-10 | -1,779 |  |  |
| 201529_s_at | RPA1 | replication protein A1, 70kDa | other | 3,26E-07 | -1,376 |  |  |  |  |
| 201533_at | CTNNB1 | catenin (cadherin-associated protein), beta 1, 88kDa | transcription regulator | 1,83E-14 | -1,550 | 1,83E-14 | -2,085 | 1,83E-14 | -1,359 |
| 201535_at | UBL3 | ubiquitin-like 3 | other | 2,60E-10 | -1,505 | 2,60E-10 | -1,621 | 2,60E-10 | -1,444 |
| 201536_at | DUSP3 | dual specificity phosphatase 3 | phosphatase | 1,83E-14 | -1,620 | 1,83E-14 | -1,962 |  |  |
| 201537_s_at | DUSP3 | dual specificity phosphatase 3 | phosphatase | 1,83E-14 | -1,725 | 1,83E-14 | -2,376 | 1,83E-14 | -1,484 |
| 201542_at | SAR1A | SAR1 homolog A (S. cerevisiae) | enzyme |  |  | 5,38E-08 | -1,510 |  |  |
| 201552_at | LAMP1 | lysosomal-associated membrane protein 1 | other |  |  | 2,84E-08 | -1,554 |  |  |
| 201560_at | CLIC4 | chloride intracellular channel 4 | ion channel | 1,21E-09 | -1,674 |  |  | 1,21E-09 | -1,355 |
| 201563_at | SORD | sorbitol dehydrogenase | enzyme |  |  | 6,51E-08 | -1,598 |  |  |
| 201565_s_at | ID2 | inhibitor of DNA binding 2, dominant negative helix-loop-helix protein | transcription regulator |  |  | 2,05E-10 | -1,682 |  |  |
| 201571_s_at | DCTD | dCMP deaminase | enzyme |  |  | 1,36E-07 | -1,649 |  |  |
| 201572_x_at | DCTD | dCMP deaminase | enzyme |  |  | 3,05E-08 | -1,720 |  |  |
| 201575_at | SNW1 | SNW domain containing 1 | transcription regulator |  |  | 9,98E-09 | -1,538 |  |  |
| 201576_s_at | GLB1 | galactosidase, beta 1 | enzyme |  |  | 2,88E-08 | -1,602 |  |  |
| 201583_s_at | SEC23B | Sec23 homolog B (S. cerevisiae) | transporter |  |  | 6,25E-10 | -1,640 |  |  |
| 201584_s_at | DDX39 | DEAD (Asp-Glu-Ala-Asp) box polypeptide 39 | enzyme |  |  | 5,61E-08 | -1,544 |  |  |
| 201588_at | TXNL1 | thioredoxin-like 1 | enzyme |  |  | 9,54E-11 | -1,644 |  |  |
| 201594_s_at | PPP4R1 | protein phosphatase 4, regulatory subunit 1 | phosphatase | 6,98E-11 | -1,484 | 6,98E-11 | -1,544 |  |  |
| 201595_s_at | ZC3H15 | zinc finger CCCH-type containing 15 | other |  |  | 2,22E-09 | -1,557 |  |  |
| 201599_at | OAT | ornithine aminotransferase (gyrate atrophy) | enzyme | 1,02E-07 | -1,303 |  |  |  |  |
| 201600_at | PHB2 | prohibitin 2 | transcription regulator |  |  | 1,23E-07 | -1,488 |  |  |
| 201606_s_at | PWP1 | PWP1 homolog (S. cerevisiae) | other |  |  | 3,28E-09 | -1,519 |  |  |
| 201608_s_at | PWP1 | PWP1 homolog (S. cerevisiae) | other |  |  | 4,76E-09 | -1,713 |  |  |
| 201611_s_at | ICMT | isoprenylcysteine carboxyl methyltransferase | enzyme |  |  | 1,72E-08 | -1,700 |  |  |
| 201612_at | ALDH9A1 | aldehyde dehydrogenase 9 family, member A1 | enzyme |  |  | 1,02E-09 | -1,690 |  |  |
| 201617_x_at | CALD1 | caldesmon 1 | other | 7,80E-12 | -1,571 | 7,80E-12 | -1,757 | 7,80E-12 | -1,432 |
| 201618_x_at | GPAA1 | glycosylphosphatidylinositol anchor attachment protein 1 homolog (yeast) | enzyme |  |  | 1,60E-08 | -1,952 |  |  |
| 201624_at | DARS | aspartyl-tRNA synthetase | enzyme |  |  | 2,74E-09 | -1,635 |  |  |
| 201629_s_at | ACP1 | acid phosphatase 1, soluble | phosphatase |  |  | 1,43E-09 | -1,582 |  |  |
| 201630_s_at | ACP1 | acid phosphatase 1, soluble | phosphatase |  |  | 1,98E-11 | -1,775 |  |  |
| 201631_s_at | IER3 | immediate early response 3 | other |  |  |  |  | 1,83E-14 | 1,694 |
| 201634_s_at | CYB5B | cytochrome b5 type B (outer mitochondrial membrane) | enzyme |  |  | 1,57E-07 | -1,487 |  |  |
| 201636_at | FXR1 | fragile X mental retardation, autosomal homolog 1 | other |  |  | 2,36E-08 | -1,538 |  |  |
| 201641_at | BST2 | bone marrow stromal cell antigen 2 | other | 1,83E-14 | -1,759 | 1,83E-14 | -2,694 | 1,83E-14 | -1,720 |
| 201642_at | IFNGR2 | interferon gamma receptor 2 (interferon gamma transducer 1) | transmembrane receptor |  |  | 4,36E-08 | -1,679 |  |  |
| 201648_at | JAK1 | Janus kinase 1 (a protein tyrosine kinase) | kinase | 1,28E-10 | -1,584 | 1,28E-10 | -1,657 |  |  |
| 201652_at | COPS5 | COP9 constitutive photomorphogenic homolog subunit 5 (Arabidopsis) | transcription regulator |  |  | 1,35E-08 | -1,576 |  |  |
| 201653_at | CNIH | cornichon homolog (Drosophila) | other |  |  | 1,42E-10 | -1,579 |  |  |
| 201655_s_at | HSPG2 (includes EG:3339) | heparan sulfate proteoglycan 2 | other | 1,80E-10 | -1,798 | 1,80E-10 | -2,106 | 1,80E-10 | -1,676 |
| 201666_at | TIMP1 | TIMP metallopeptidase inhibitor 1 | other |  |  | 7,51E-08 | -1,643 |  |  |
| 201668_x_at | MARCKS (includes EG:4082) | myristoylated alanine-rich protein kinase C substrate | other |  |  |  |  | 3,57E-10 | 2,275 |
| 201669_s_at | MARCKS (includes EG:4082) | myristoylated alanine-rich protein kinase C substrate | other |  |  | 1,44E-12 | -1,698 | 1,44E-12 | -1,313 |
| 201672_s_at | USP14 | ubiquitin specific peptidase 14 (tRNA-guanine transglycosylase) | peptidase |  |  | 5,05E-09 | -1,452 |  |  |
| 201673_s_at | GYS1 | glycogen synthase 1 (muscle) | enzyme |  |  | 5,27E-08 | -2,066 |  |  |
| 201681_s_at | DLG5 | discs, large homolog 5 (Drosophila) | other |  |  | 9,15E-09 | -1,491 |  |  |
| 201688_s_at | TPD52 | tumor protein D52 | other |  |  | 2,10E-07 | -1,646 |  |  |
| 201689_s_at | TPD52 | tumor protein D52 | other |  |  | 2,88E-11 | -1,718 |  |  |
| 201692_at | OPRS1 | sigma non-opioid intracellular receptor 1 | G-protein coupled receptor |  |  | 4,15E-10 | -1,983 |  |  |
| 201694_s_at | EGR1 | early growth response 1 | transcription regulator |  |  |  |  | 1,30E-08 | 1,451 |
| 201695_s_at | NP | nucleoside phosphorylase | enzyme |  |  | 3,14E-08 | -1,751 |  |  |
| 201699_at | PSMC6 | proteasome (prosome, macropain) 26S subunit, ATPase, 6 | peptidase | 7,28E-12 | -1,476 | 7,28E-12 | -1,612 | 7,28E-12 | -1,304 |
| 201705_at | PSMD7 | proteasome (prosome, macropain) 26S subunit, non-ATPase, 7 | other | 1,83E-14 | -1,538 | 1,83E-14 | -1,913 | 1,83E-14 | -1,383 |
| 201710_at | MYBL2 | v-myb myeloblastosis viral oncogene homolog (avian)-like 2 | transcription regulator |  |  | 1,83E-14 | -2,112 | 1,83E-14 | -1,349 |
| 201713_s_at | RANBP2 | RAN binding protein 2 | enzyme |  |  | 9,02E-11 | -1,650 |  |  |
| 201714_at | TUBG1 | tubulin, gamma 1 | other |  |  | 1,97E-10 | -1,760 |  |  |
| 201716_at | SNX1 | sorting nexin 1 | transporter |  |  | 7,88E-10 | -1,806 |  |  |
| 201717_at | MRPL49 | mitochondrial ribosomal protein L49 | enzyme |  |  | 2,50E-09 | -1,689 |  |  |
| 201718_s_at | EPB41L2 | erythrocyte membrane protein band 4.1-like 2 | other |  |  | 2,73E-09 | -1,736 |  |  |
| 201722_s_at | GALNT1 | UDP-N-acetyl-alpha-D-galactosamine:polypeptide N-acetylgalactosaminyltransferase 1 (GalNAc-T1) | enzyme | 2,01E-10 | -1,539 | 2,01E-10 | -1,552 |  |  |
| 201724_s_at | GALNT1 | UDP-N-acetyl-alpha-D-galactosamine:polypeptide N-acetylgalactosaminyltransferase 1 (GalNAc-T1) | enzyme | 1,46E-10 | -1,438 | 1,46E-10 | -1,629 |  |  |
| 201737_s_at | MARCH6 | membrane-associated ring finger (C3HC4) 6 | other |  |  | 2,01E-08 | -1,567 |  |  |
| 201740_at | NDUFS3 | NADH dehydrogenase (ubiquinone) Fe-S protein 3, 30kDa (NADH-coenzyme Q reductase) | enzyme |  |  | 1,39E-07 | -1,501 |  |  |
| 201752_s_at | ADD3 | adducin 3 (gamma) | other |  |  | 3,95E-09 | -1,555 |  |  |
| 201756_at | RPA2 | replication protein A2, 32kDa | other | 3,37E-12 | -1,462 | 3,37E-12 | -1,696 | 3,37E-12 | -1,396 |
| 201758_at | TSG101 | tumor susceptibility gene 101 | transcription regulator | 7,60E-10 | -1,481 | 7,60E-10 | -1,697 |  |  |
| 201760_s_at | WSB2 | WD repeat and SOCS box-containing 2 | other |  |  | 3,44E-12 | -1,813 |  |  |
| 201762_s_at | PSME2 | proteasome (prosome, macropain) activator subunit 2 (PA28 beta) | peptidase |  |  | 6,84E-09 | -1,562 |  |  |
| 201765_s_at | HEXA | hexosaminidase A (alpha polypeptide) | enzyme | 1,83E-14 | -2,037 | 1,83E-14 | -2,367 | 1,83E-14 | -1,823 |
| 201770_at | SNRPA | small nuclear ribonucleoprotein polypeptide A | other |  |  | 1,17E-10 | -1,765 |  |  |
| 201773_at | ADNP | activity-dependent neuroprotector homeobox | transcription regulator |  |  | 8,88E-09 | -1,510 |  |  |
| 201781_s_at | AIP | aryl hydrocarbon receptor interacting protein | transcription regulator |  |  | 1,58E-08 | -1,958 |  |  |
| 201782_s_at | AIP | aryl hydrocarbon receptor interacting protein | transcription regulator |  |  | 2,47E-08 | -1,870 |  |  |
| 201790_s_at | DHCR7 | 7-dehydrocholesterol reductase | enzyme |  |  | 3,88E-10 | -1,933 |  |  |
| 201791_s_at | DHCR7 | 7-dehydrocholesterol reductase | enzyme | 1,83E-14 | -1,627 | 1,83E-14 | -2,112 | 1,83E-14 | -1,432 |
| 201797_s_at | VARS | valyl-tRNA synthetase | enzyme |  |  | 1,02E-12 | -2,232 |  |  |
| 201812_s_at | TOMM7 | translocase of outer mitochondrial membrane 7 homolog (yeast) | transporter |  |  | 1,69E-09 | -1,544 | 1,69E-09 | -1,313 |
| 201816_s_at | GBAS | glioblastoma amplified sequence | other | 6,26E-14 | -1,405 | 6,26E-14 | -1,727 | 6,26E-14 | -1,458 |
| 201817_at | UBE3C | ubiquitin protein ligase E3C | enzyme | 4,75E-11 | -1,396 | 4,75E-11 | -1,653 |  |  |
| 201840_at | NEDD8 | neural precursor cell expressed, developmentally down-regulated 8 | enzyme |  |  | 8,36E-09 | -1,649 |  |  |
| 201847_at | LIPA | lipase A, lysosomal acid, cholesterol esterase | enzyme | 4,58E-11 | -1,438 | 4,58E-11 | -1,611 | 4,58E-11 | -1,380 |
| 201862_s_at | LRRFIP1 | leucine rich repeat (in FLII) interacting protein 1 | transcription regulator |  |  | 1,37E-09 | -1,619 |  |  |
| 201863_at | FAM32A | family with sequence similarity 32, member A | other | 3,91E-10 | -1,473 | 3,91E-10 | -1,700 | 3,91E-10 | -1,345 |
| 201870_at | TOMM34 | translocase of outer mitochondrial membrane 34 | other | 3,44E-12 | -1,482 | 3,44E-12 | -1,854 | 3,44E-12 | -1,411 |
| 201874_at | MPZL1 | myelin protein zero-like 1 | other | 1,83E-14 | -1,690 | 1,83E-14 | -2,005 | 1,83E-14 | -1,436 |
| 201880_at | ARIH1 | ariadne homolog, ubiquitin-conjugating enzyme E2 binding protein, 1 (Drosophila) | enzyme |  |  | 1,25E-09 | -1,590 |  |  |
| 201883_s_at | B4GALT1 | UDP-Gal:betaGlcNAc beta 1,4- galactosyltransferase, polypeptide 1 | enzyme | 1,85E-13 | -1,558 | 1,85E-13 | -1,670 |  |  |
| 201890_at | RRM2 | ribonucleotide reductase M2 polypeptide | enzyme |  |  | 6,06E-08 | -1,403 |  |  |
| 201891_s_at | B2M | beta-2-microglobulin | transmembrane receptor |  |  | 4,41E-08 | -1,442 |  |  |
| 201896_s_at | PSRC1 | proline/serine-rich coiled-coil 1 | other | 1,27E-11 | -1,518 | 1,27E-11 | -2,040 | 1,27E-11 | -1,597 |
| 201897_s_at | CKS1B | CDC28 protein kinase regulatory subunit 1B | kinase |  |  | 5,78E-09 | -1,538 |  |  |
| 201899_s_at | UBE2A | ubiquitin-conjugating enzyme E2A (RAD6 homolog) | enzyme |  |  | 1,16E-09 | -1,530 |  |  |
| 201908_at | DVL3 | dishevelled, dsh homolog 3 (Drosophila) | other |  |  | 7,83E-12 | -1,904 |  |  |
| 201910_at | FARP1 | FERM, RhoGEF (ARHGEF) and pleckstrin domain protein 1 (chondrocyte-derived) | other | 7,56E-09 | -1,850 | 7,56E-09 | -2,065 |  |  |
| 201912_s_at | GSPT1 | G1 to S phase transition 1 | translation regulator |  |  | 4,16E-10 | -1,593 |  |  |
| 201913_s_at | COASY | Coenzyme A synthase | kinase |  |  | 2,84E-09 | -1,917 |  |  |
| 201916_s_at | SEC63 | SEC63 homolog (S. cerevisiae) | transporter | 1,83E-14 | -1,567 | 1,83E-14 | -1,785 |  |  |
| 201917_s_at | SLC25A36 | solute carrier family 25, member 36 | transporter |  |  |  |  | 3,03E-07 | 1,561 |
| 201922_at | TINP1 | TGF beta-inducible nuclear protein 1 | other |  |  | 2,77E-08 | -1,457 |  |  |
| 201923_at | PRDX4 | peroxiredoxin 4 | enzyme |  |  | 1,37E-08 | -1,496 |  |  |
| 201925_s_at | CD55 | CD55 molecule, decay accelerating factor for complement (Cromer blood group) | other | 2,13E-09 | -1,440 |  |  |  |  |
| 201926_s_at | CD55 | CD55 molecule, decay accelerating factor for complement (Cromer blood group) | other | 2,16E-13 | -1,628 | 2,16E-13 | -1,704 |  |  |
| 201928_at | PKP4 | plakophilin 4 | other | 2,39E-10 | -1,527 | 2,39E-10 | -1,458 |  |  |
| 201929_s_at | PKP4 | plakophilin 4 | other | 4,87E-14 | -1,587 | 4,87E-14 | -1,823 |  |  |
| 201930_at | MCM6 | minichromosome maintenance complex component 6 | enzyme |  |  | 1,10E-10 | -1,667 |  |  |
| 201931_at | ETFA | electron-transfer-flavoprotein, alpha polypeptide | transporter |  |  | 2,46E-10 | -1,564 |  |  |
| 201933_at | CHMP1A | chromatin modifying protein 1A | peptidase |  |  | 1,62E-08 | -1,730 |  |  |
| 201934_at | WDR82 | WD repeat domain 82 | other |  |  | 5,35E-09 | -1,610 |  |  |
| 201937_s_at | DNPEP | aspartyl aminopeptidase | peptidase |  |  | 1,09E-08 | -1,816 |  |  |
| 201940_at | CPD | carboxypeptidase D | peptidase |  |  | 5,20E-09 | -1,580 |  |  |
| 201949_x_at | CAPZB | capping protein (actin filament) muscle Z-line, beta | other |  |  | 1,38E-10 | -1,762 |  |  |
| 201950_x_at | CAPZB | capping protein (actin filament) muscle Z-line, beta | other |  |  | 1,02E-11 | -1,938 |  |  |
| 201953_at | CIB1 | calcium and integrin binding 1 (calmyrin) | other |  |  | 2,59E-08 | -1,635 |  |  |
| 201955_at | CCNC | cyclin C | other |  |  | 9,65E-09 | -1,446 |  |  |
| 201967_at | RBM6 | RNA binding motif protein 6 | other |  |  | 1,58E-09 | -1,878 |  |  |
| 201973_s_at | C7ORF28A | chromosome 7 open reading frame 28A | other |  |  | 2,21E-10 | -1,669 |  |  |
| 201983_s_at | EGFR | epidermal growth factor receptor (erythroblastic leukemia viral (v-erb-b) oncogene homolog, avian) | kinase |  |  |  |  | 3,56E-11 | 1,551 |
| 201996_s_at | SPEN | spen homolog, transcriptional regulator (Drosophila) | transcription regulator |  |  |  |  | 3,44E-14 | 1,953 |
| 201999_s_at | DYNLT1 | dynein, light chain, Tctex-type 1 | other |  |  | 2,03E-08 | -1,560 |  |  |
| 202000_at | NDUFA6 | NADH dehydrogenase (ubiquinone) 1 alpha subcomplex, 6, 14kDa | enzyme |  |  | 7,95E-08 | -1,700 |  |  |
| 202004_x_at | SDHC | succinate dehydrogenase complex, subunit C, integral membrane protein, 15kDa | enzyme | 1,83E-14 | -1,902 | 1,83E-14 | -2,743 | 1,83E-14 | -1,847 |
| 202005_at | ST14 | suppression of tumorigenicity 14 (colon carcinoma) | peptidase |  |  | 2,72E-08 | -1,642 |  |  |
| 202007_at | NID1 | nidogen 1 | other | 2,27E-10 | -1,471 | 2,27E-10 | -1,724 | 2,27E-10 | -1,327 |
| 202023_at | EFNA1 | ephrin-A1 | other |  |  | 2,96E-09 | -2,157 |  |  |
| 202024_at | ASNA1 | arsA arsenite transporter, ATP-binding, homolog 1 (bacterial) | transporter |  |  | 1,14E-12 | -2,167 |  |  |
| 202028_s_at | RPL38 (includes EG:6169) | ribosomal protein L38 | other |  |  | 1,63E-13 | 1,796 |  |  |
| 202031_s_at | WIPI2 | WD repeat domain, phosphoinositide interacting 2 | other | 4,69E-13 | -1,536 | 4,69E-13 | -1,907 | 4,69E-13 | -1,427 |
| 202042_at | HARS | histidyl-tRNA synthetase | enzyme |  |  | 3,97E-08 | -1,564 |  |  |
| 202043_s_at | SMS | spermine synthase | enzyme | 2,03E-12 | -1,523 | 2,03E-12 | -1,766 | 2,03E-12 | -1,429 |
| 202060_at | CTR9 | Ctr9, Paf1/RNA polymerase II complex component, homolog (S. cerevisiae) | other | 4,80E-12 | -1,354 | 4,80E-12 | -1,628 | 4,80E-12 | -1,275 |
| 202061_s_at | SEL1L | sel-1 suppressor of lin-12-like (C. elegans) | other | 4,12E-12 | -1,447 | 4,12E-12 | -1,649 |  |  |
| 202066_at | PPFIA1 | protein tyrosine phosphatase, receptor type, f polypeptide (PTPRF), interacting protein (liprin), alpha 1 | phosphatase | 1,80E-09 | -1,405 | 1,80E-09 | -1,579 |  |  |
| 202071_at | SDC4 | syndecan 4 | other | 1,83E-14 | -1,419 | 1,83E-14 | -1,535 |  |  |
| 202077_at | NDUFAB1 | NADH dehydrogenase (ubiquinone) 1, alpha/beta subcomplex, 1, 8kDa | enzyme |  |  | 5,37E-10 | -1,585 |  |  |
| 202087_s_at | CTSL1 | cathepsin L1 | peptidase |  |  | 6,12E-09 | -1,758 | 6,12E-09 | -1,419 |
| 202088_at | SLC39A6 | solute carrier family 39 (zinc transporter), member 6 | transporter |  |  | 5,55E-10 | -1,531 |  |  |
| 202090_s_at | UQCR | ubiquinol-cytochrome c reductase, 6.4kDa subunit | enzyme |  |  | 2,67E-13 | -1,733 | 2,67E-13 | -1,391 |
| 202094_at | BIRC5 | baculoviral IAP repeat-containing 5 | other |  |  | 4,82E-08 | -1,776 |  |  |
| 202095_s_at | BIRC5 | baculoviral IAP repeat-containing 5 | other | 5,89E-09 | -1,472 | 5,89E-09 | -1,616 |  |  |
| 202097_at | NUP153 | nucleoporin 153kDa | transporter | 6,72E-12 | -1,328 | 6,72E-12 | -1,582 |  |  |
| 202100_at | RALB | v-ral simian leukemia viral oncogene homolog B (ras related; GTP binding protein) | enzyme | 1,26E-13 | -1,575 | 1,26E-13 | -1,931 |  |  |
| 202101_s_at | RALB | v-ral simian leukemia viral oncogene homolog B (ras related; GTP binding protein) | enzyme |  |  |  |  | 2,33E-08 | -1,522 |
| 202104_s_at | SPG7 | spastic paraplegia 7 (pure and complicated autosomal recessive) | peptidase |  |  | 5,98E-09 | -1,662 |  |  |
| 202107_s_at | MCM2 | minichromosome maintenance complex component 2 | enzyme | 3,84E-10 | -1,348 | 3,84E-10 | -1,715 |  |  |
| 202109_at | ARFIP2 | ADP-ribosylation factor interacting protein 2 | other |  |  | 5,29E-08 | -2,107 |  |  |
| 202111_at | SLC4A2 | solute carrier family 4, anion exchanger, member 2 (erythrocyte membrane protein band 3-like 1) | transporter |  |  | 1,15E-09 | -1,972 |  |  |
| 202113_s_at | SNX2 | sorting nexin 2 | transporter |  |  | 2,40E-08 | -1,485 |  |  |
| 202116_at | DPF2 | D4, zinc and double PHD fingers family 2 | other |  |  | 2,57E-10 | -1,784 |  |  |
| 202117_at | ARHGAP1 | Rho GTPase activating protein 1 | other |  |  |  |  | 1,90E-07 | -1,374 |
| 202121_s_at | CHMP2A | chromatin modifying protein 2A | other | 8,31E-12 | -1,392 | 8,31E-12 | -1,724 | 8,31E-12 | -1,431 |
| 202133_at | WWTR1 | WW domain containing transcription regulator 1 | transcription regulator |  |  | 1,05E-07 | -1,524 |  |  |
| 202135_s_at | ACTR1B (includes EG:10120) | ARP1 actin-related protein 1 homolog B, centractin beta (yeast) | other |  |  | 1,93E-09 | -2,046 |  |  |
| 202139_at | AKR7A2 | aldo-keto reductase family 7, member A2 (aflatoxin aldehyde reductase) | enzyme | 3,72E-13 | -1,439 | 3,72E-13 | -1,804 | 3,72E-13 | -1,427 |
| 202143_s_at | COPS8 | COP9 constitutive photomorphogenic homolog subunit 8 (Arabidopsis) | other |  |  | 1,25E-07 | -1,653 |  |  |
| 202153_s_at | NUP62 | nucleoporin 62kDa | transporter | 4,01E-12 | -1,596 | 4,01E-12 | -2,010 |  |  |
| 202165_at | PPP1R2 | protein phosphatase 1, regulatory (inhibitor) subunit 2 | phosphatase | 3,19E-11 | -1,472 | 3,19E-11 | -1,744 | 3,19E-11 | -1,326 |
| 202166_s_at | PPP1R2 | protein phosphatase 1, regulatory (inhibitor) subunit 2 | phosphatase | 7,33E-11 | -1,294 | 7,33E-11 | -1,558 | 7,33E-11 | -1,348 |
| 202171_at | VEZF1 | vascular endothelial zinc finger 1 | transcription regulator |  |  | 2,91E-10 | -1,688 |  |  |
| 202173_s_at | VEZF1 | vascular endothelial zinc finger 1 | transcription regulator |  |  | 1,85E-09 | -1,665 |  |  |
| 202174_s_at | PCM1 | pericentriolar material 1 | other |  |  | 3,67E-12 | -1,801 |  |  |
| 202179_at | BLMH | bleomycin hydrolase | peptidase |  |  | 9,17E-09 | -2,061 |  |  |
| 202183_s_at | KIF22 | kinesin family member 22 | other | 2,67E-11 | -1,339 | 2,67E-11 | -1,860 |  |  |
| 202184_s_at | NUP133 | nucleoporin 133kDa | transporter |  |  | 1,72E-08 | -1,550 |  |  |
| 202193_at | LIMK2 | LIM domain kinase 2 | kinase |  |  | 2,33E-08 | -1,984 |  |  |
| 202201_at | BLVRB | biliverdin reductase B (flavin reductase (NADPH)) | enzyme |  |  | 2,04E-08 | -1,723 |  |  |
| 202207_at | ARL4C | ADP-ribosylation factor-like 4C | enzyme | 9,26E-12 | -1,479 | 9,26E-12 | -1,881 | 9,26E-12 | -1,368 |
| 202215_s_at | NFYC | nuclear transcription factor Y, gamma | transcription regulator |  |  | 7,31E-08 | -1,883 |  |  |
| 202227_s_at | BRD8 | bromodomain containing 8 | transcription regulator | 8,73E-11 | -1,451 | 8,73E-11 | -1,555 |  |  |
| 202233_s_at | UQCRH | ubiquinol-cytochrome c reductase hinge protein | enzyme |  |  | 4,73E-09 | -1,650 |  |  |
| 202241_at | TRIB1 | tribbles homolog 1 (Drosophila) | kinase |  |  |  |  | 1,83E-14 | 1,549 |
| 202245_at | LSS | lanosterol synthase (2,3-oxidosqualene-lanosterol cyclase) | enzyme |  |  | 5,39E-13 | -1,914 |  |  |
| 202246_s_at | CDK4 | cyclin-dependent kinase 4 | kinase |  |  | 3,41E-10 | -1,612 |  |  |
| 202252_at | RAB13 | RAB13, member RAS oncogene family | enzyme |  |  | 1,76E-10 | -1,755 |  |  |
| 202263_at | CYB5R1 | cytochrome b5 reductase 1 | enzyme | 8,39E-10 | -1,812 | 8,39E-10 | -1,982 |  |  |
| 202265_at | BMI1 | BMI1 polycomb ring finger oncogene | transcription regulator |  |  | 7,26E-10 | -1,642 |  |  |
| 202272_s_at | FBXO28 | F-box protein 28 | other |  |  | 2,34E-08 | -1,636 |  |  |
| 202279_at | C14ORF2 | chromosome 14 open reading frame 2 | other | 5,14E-10 | -1,435 | 5,14E-10 | -1,598 | 5,14E-10 | -1,403 |
| 202282_at | HSD17B10 | hydroxysteroid (17-beta) dehydrogenase 10 | enzyme |  |  | 2,78E-11 | -1,710 | 2,78E-11 | -1,309 |
| 202290_at | PDAP1 | PDGFA associated protein 1 | other | 1,83E-14 | -1,854 | 1,83E-14 | -2,541 | 1,83E-14 | -1,997 |
| 202295_s_at | CTSH | cathepsin H | peptidase |  |  | 3,12E-10 | -1,701 |  |  |
| 202299_s_at | HBXIP | hepatitis B virus x interacting protein | other | 1,83E-14 | -1,515 | 1,83E-14 | -1,841 | 1,83E-14 | -1,395 |
| 202300_at | HBXIP | hepatitis B virus x interacting protein | other |  |  | 8,69E-09 | -1,610 |  |  |
| 202314_at | CYP51A1 | cytochrome P450, family 51, subfamily A, polypeptide 1 | enzyme |  |  | 6,06E-09 | -1,559 |  |  |
| 202324_s_at | ACBD3 | acyl-Coenzyme A binding domain containing 3 | other |  |  | 4,42E-08 | -1,648 |  |  |
| 202325_s_at | ATP5J | ATP synthase, H+ transporting, mitochondrial F0 complex, subunit F6 | transporter | 3,60E-09 | -1,359 | 3,60E-09 | -1,474 | 3,60E-09 | -1,328 |
| 202333_s_at | UBE2B | ubiquitin-conjugating enzyme E2B (RAD6 homolog) | enzyme | 4,63E-11 | -1,364 | 4,63E-11 | -1,604 | 4,63E-11 | -1,281 |
| 202338_at | TK1 | thymidine kinase 1, soluble | kinase | 2,23E-09 | -1,455 | 2,23E-09 | -1,640 | 2,23E-09 | -1,381 |
| 202348_s_at | TOR1A | torsin family 1, member A (torsin A) | peptidase |  |  |  |  | 1,44E-07 | -1,416 |
| 202352_s_at | PSMD12 | proteasome (prosome, macropain) 26S subunit, non-ATPase, 12 | other |  |  | 1,25E-09 | -1,628 |  |  |
| 202360_at | MAML1 | mastermind-like 1 (Drosophila) | transcription regulator |  |  | 4,09E-11 | -1,805 |  |  |
| 202361_at | SEC24C (includes EG:9632) | SEC24 family, member C (S. cerevisiae) | transporter | 1,83E-14 | -1,796 | 1,83E-14 | -2,518 | 1,83E-14 | -1,676 |
| 202362_at | RAP1A | RAP1A, member of RAS oncogene family | enzyme | 4,87E-14 | -1,308 | 4,87E-14 | -1,857 | 4,87E-14 | -1,646 |
| 202365_at | UNC119B | unc-119 homolog B (C. elegans) | other | 1,24E-10 | -1,526 | 1,24E-10 | -1,617 | 1,24E-10 | -1,385 |
| 202368_s_at | TRAM2 (includes EG:9697) | translocation associated membrane protein 2 | other |  |  | 8,73E-09 | -1,762 |  |  |
| 202369_s_at | TRAM2 (includes EG:9697) | translocation associated membrane protein 2 | other | 7,08E-11 | -1,450 | 7,08E-11 | -1,610 | 7,08E-11 | -1,617 |
| 202382_s_at | GNPDA1 (includes EG:10007) | glucosamine-6-phosphate deaminase 1 | enzyme |  |  | 1,66E-08 | -1,683 |  |  |
| 202395_at | NSF | N-ethylmaleimide-sensitive factor | transporter | 8,26E-13 | -1,777 | 8,26E-13 | -1,649 | 8,26E-13 | -1,341 |
| 202396_at | TCERG1 | transcription elongation regulator 1 | transcription regulator |  |  | 6,79E-08 | -1,486 |  |  |
| 202399_s_at | AP3S2 | adaptor-related protein complex 3, sigma 2 subunit | transporter |  |  | 1,66E-07 | -1,676 |  |  |
| 202408_s_at | PRPF31 | PRP31 pre-mRNA processing factor 31 homolog (S. cerevisiae) | other |  |  | 6,79E-09 | -1,646 |  |  |
| 202409_at | IGF2 | insulin-like growth factor 2 (somatomedin A) | growth factor | 1,83E-14 | -1,518 | 1,83E-14 | -2,330 | 1,83E-14 | -1,952 |
| 202429_s_at | PPP3CA | protein phosphatase 3 (formerly 2B), catalytic subunit, alpha isoform | phosphatase |  |  | 2,61E-12 | -1,677 |  |  |
| 202430_s_at | PLSCR1 | phospholipid scramblase 1 | enzyme |  |  |  |  | 1,70E-07 | -1,443 |
| 202444_s_at | ERLIN1 | ER lipid raft associated 1 | other |  |  | 7,63E-08 | -1,756 |  |  |
| 202446_s_at | PLSCR1 | phospholipid scramblase 1 | enzyme |  |  | 9,84E-09 | -1,546 |  |  |
| 202447_at | DECR1 | 2,4-dienoyl CoA reductase 1, mitochondrial | enzyme | 1,82E-10 | -1,592 | 1,82E-10 | -1,892 |  |  |
| 202454_s_at | ERBB3 | v-erb-b2 erythroblastic leukemia viral oncogene homolog 3 (avian) | kinase |  |  | 1,26E-13 | -1,878 | 1,26E-13 | -1,699 |
| 202457_s_at | PPP3CA | protein phosphatase 3 (formerly 2B), catalytic subunit, alpha isoform | phosphatase |  |  | 3,10E-09 | -1,684 |  |  |
| 202459_s_at | LPIN2 | lipin 2 | other |  |  | 4,36E-08 | -1,691 |  |  |
| 202468_s_at | CTNNAL1 | catenin (cadherin-associated protein), alpha-like 1 | other |  |  |  |  | 9,57E-08 | -1,279 |
| 202469_s_at | CPSF6 | cleavage and polyadenylation specific factor 6, 68kDa | other | 1,83E-14 | -1,373 | 1,83E-14 | -1,937 | 1,83E-14 | -1,358 |
| 202471_s_at | IDH3G | isocitrate dehydrogenase 3 (NAD+) gamma | enzyme |  |  | 3,71E-10 | -1,831 |  |  |
| 202481_at | DHRS3 | dehydrogenase/reductase (SDR family) member 3 | enzyme | 2,38E-07 | -1,887 |  |  |  |  |
| 202487_s_at | H2AFV (includes EG:94239) | H2A histone family, member V | other | 1,66E-11 | -1,426 | 1,66E-11 | -1,783 | 1,66E-11 | -1,359 |
| 202502_at | ACADM | acyl-Coenzyme A dehydrogenase, C-4 to C-12 straight chain | enzyme |  |  | 2,55E-10 | -1,772 |  |  |
| 202505_at | SNRPB2 | small nuclear ribonucleoprotein polypeptide B'' | other |  |  | 6,02E-08 | -1,520 |  |  |
| 202516_s_at | DLG1 | discs, large homolog 1 (Drosophila) | kinase |  |  |  |  | 3,66E-08 | -1,443 |
| 202521_at | CTCF | CCCTC-binding factor (zinc finger protein) | transcription regulator | 9,05E-08 | -1,326 |  |  |  |  |
| 202529_at | PRPSAP1 | phosphoribosyl pyrophosphate synthetase-associated protein 1 | other |  |  | 7,96E-09 | -1,580 |  |  |
| 202532_s_at | DHFR | dihydrofolate reductase | enzyme |  |  | 1,59E-08 | -1,685 |  |  |
| 202534_x_at | DHFR | dihydrofolate reductase | enzyme | 1,83E-14 | -1,329 | 1,83E-14 | -1,893 | 1,83E-14 | -1,457 |
| 202538_s_at | CHMP2B | chromatin modifying protein 2B | other |  |  | 2,17E-09 | -1,601 |  |  |
| 202539_s_at | HMGCR | 3-hydroxy-3-methylglutaryl-Coenzyme A reductase | enzyme |  |  | 1,64E-08 | -1,487 |  |  |
| 202541_at | SCYE1 | small inducible cytokine subfamily E, member 1 (endothelial monocyte-activating) | cytokine |  |  | 1,50E-08 | -1,547 |  |  |
| 202542_s_at | SCYE1 | small inducible cytokine subfamily E, member 1 (endothelial monocyte-activating) | cytokine |  |  | 3,17E-12 | -1,715 |  |  |
| 202544_at | GMFB | glia maturation factor, beta | growth factor | 1,61E-12 | -1,500 | 1,61E-12 | -1,730 |  |  |
| 202546_at | VAMP8 | vesicle-associated membrane protein 8 (endobrevin) | other |  |  | 9,43E-10 | -1,640 |  |  |
| 202548_s_at | ARHGEF7 | Rho guanine nucleotide exchange factor (GEF) 7 | other |  |  | 4,43E-09 | -1,661 |  |  |
| 202550_s_at | VAPB | VAMP (vesicle-associated membrane protein)-associated protein B and C | other | 6,83E-12 | -1,374 | 6,83E-12 | -1,678 |  |  |
| 202553_s_at | SYF2 | SYF2 homolog, RNA splicing factor (S. cerevisiae) | other | 6,29E-10 | -1,786 | 6,29E-10 | -1,569 |  |  |
| 202554_s_at | GSTM3 (includes EG:2947) | glutathione S-transferase mu 3 (brain) | enzyme | 9,27E-10 | -1,377 | 9,27E-10 | -1,598 | 9,27E-10 | -1,435 |
| 202556_s_at | MCRS1 | microspherule protein 1 | other |  |  | 4,97E-09 | -1,910 |  |  |
| 202560_s_at | C1ORF77 | chromosome 1 open reading frame 77 | other | 2,52E-10 | -1,468 | 2,52E-10 | -1,720 |  |  |
| 202562_s_at | C14ORF1 | chromosome 14 open reading frame 1 | other | 7,33E-12 | -1,432 | 7,33E-12 | -1,700 | 7,33E-12 | -1,326 |
| 202565_s_at | SVIL | supervillin | other | 6,53E-10 | -1,566 | 6,53E-10 | -1,603 |  |  |
| 202578_s_at | DDX19A | DEAD (Asp-Glu-Ala-As) box polypeptide 19A | enzyme |  |  | 5,03E-10 | -1,809 |  |  |
| 202579_x_at | HMGN4 | high mobility group nucleosomal binding domain 4 | other | 2,63E-10 | -1,491 | 2,63E-10 | -1,548 |  |  |
| 202580_x_at | FOXM1 | forkhead box M1 | transcription regulator |  |  | 1,35E-09 | -1,895 |  |  |
| 202583_s_at | RANBP9 | RAN binding protein 9 | other | 1,06E-12 | -1,645 | 1,06E-12 | -1,783 |  |  |
| 202587_s_at | AK1 | adenylate kinase 1 | kinase |  |  | 4,28E-09 | -1,978 |  |  |
| 202589_at | TYMS | thymidylate synthetase | enzyme |  |  | 3,22E-07 | -1,423 |  |  |
| 202591_s_at | SSBP1 | single-stranded DNA binding protein 1 | other |  |  | 6,09E-10 | -1,572 |  |  |
| 202593_s_at | GDE1 | glycerophosphodiester phosphodiesterase 1 | enzyme |  |  | 1,29E-08 | -1,639 |  |  |
| 202595_s_at | LEPROTL1 | leptin receptor overlapping transcript-like 1 | other | 7,76E-11 | -1,316 | 7,76E-11 | -1,682 | 7,76E-11 | -1,479 |
| 202596_at | ENSA | endosulfine alpha | transporter |  |  | 1,01E-08 | -1,753 |  |  |
| 202598_at | S100A13 | S100 calcium binding protein A13 | other | 1,69E-11 | -1,459 | 1,69E-11 | -1,952 |  |  |
| 202602_s_at | HTATSF1 | HIV-1 Tat specific factor 1 | transcription regulator | 2,06E-13 | -1,379 | 2,06E-13 | -1,835 | 2,06E-13 | -1,660 |
| 202605_at | GUSB | glucuronidase, beta | enzyme | 1,27E-10 | -1,493 | 1,27E-10 | -1,645 |  |  |
| 202609_at | EPS8 | epidermal growth factor receptor pathway substrate 8 | peptidase |  |  | 3,73E-09 | -1,440 |  |  |
| 202613_at | CTPS | CTP synthase | enzyme |  |  | 2,72E-09 | -1,602 |  |  |
| 202619_s_at | PLOD2 | procollagen-lysine, 2-oxoglutarate 5-dioxygenase 2 | enzyme |  |  |  |  | 3,41E-06 | 1,381 |
| 202620_s_at | PLOD2 | procollagen-lysine, 2-oxoglutarate 5-dioxygenase 2 | enzyme | 7,55E-14 | -1,566 | 7,55E-14 | -1,672 |  |  |
| 202623_at | EAPP | E2F-associated phosphoprotein | other | 1,83E-14 | -1,455 | 1,83E-14 | -2,045 | 1,83E-14 | -1,379 |
| 202624_s_at | CABIN1 | calcineurin binding protein 1 | other |  |  | 7,21E-10 | -3,110 |  |  |
| 202629_at | APPBP2 | amyloid beta precursor protein (cytoplasmic tail) binding protein 2 | other |  |  | 1,37E-08 | -1,649 |  |  |
| 202630_at | APPBP2 | amyloid beta precursor protein (cytoplasmic tail) binding protein 2 | other | 8,85E-09 | -1,885 |  |  |  |  |
| 202632_at | DPH1 | DPH1 homolog (S. cerevisiae) | other |  |  | 5,58E-10 | -2,008 |  |  |
| 202635_s_at | POLR2K | polymerase (RNA) II (DNA directed) polypeptide K, 7.0kDa | enzyme |  |  | 1,64E-08 | -1,585 |  |  |
| 202641_at | ARL3 | ADP-ribosylation factor-like 3 | other |  |  | 3,06E-09 | -1,625 | 3,06E-09 | -1,322 |
| 202642_s_at | TRRAP | transformation/transcription domain-associated protein | transcription regulator |  |  | 9,81E-10 | -1,858 |  |  |
| 202645_s_at | MEN1 | multiple endocrine neoplasia I | transcription regulator |  |  | 1,49E-09 | -1,896 |  |  |
| 202646_s_at | CSDE1 | cold shock domain containing E1, RNA-binding | enzyme |  |  | 9,68E-11 | -1,702 |  |  |
| 202658_at | PEX11B | peroxisomal biogenesis factor 11 beta | other |  |  | 3,72E-08 | -1,684 |  |  |
| 202668_at | EFNB2 | ephrin-B2 | other |  |  |  |  | 1,83E-14 | 1,310 |
| 202669_s_at | EFNB2 | ephrin-B2 | other |  |  |  |  | 2,88E-11 | 2,223 |
| 202670_at | MAP2K1 | mitogen-activated protein kinase kinase 1 | kinase |  |  | 1,39E-07 | -1,689 |  |  |
| 202671_s_at | PDXK | pyridoxal (pyridoxine, vitamin B6) kinase | kinase |  |  | 1,83E-14 | -1,997 |  |  |
| 202683_s_at | RNMT | RNA (guanine-7-) methyltransferase | enzyme |  |  | 1,41E-08 | -1,717 |  |  |
| 202703_at | DUSP11 | dual specificity phosphatase 11 (RNA/RNP complex 1-interacting) | phosphatase |  |  | 4,06E-09 | -1,588 |  |  |
| 202715_at | CAD | carbamoyl-phosphate synthetase 2, aspartate transcarbamylase, and dihydroorotase | enzyme |  |  | 1,21E-08 | -1,751 |  |  |
| 202717_s_at | CDC16 | cell division cycle 16 homolog (S. cerevisiae) | other | 1,84E-11 | -1,439 | 1,84E-11 | -1,764 | 1,84E-11 | -1,435 |
| 202720_at | TES | testis derived transcript (3 LIM domains) | other | 1,17E-10 | -1,513 | 1,17E-10 | -1,564 |  |  |
| 202729_s_at | LTBP1 | latent transforming growth factor beta binding protein 1 | other | 3,83E-12 | -1,698 | 3,83E-12 | -1,823 |  |  |
| 202736_s_at | LSM4 | LSM4 homolog, U6 small nuclear RNA associated (S. cerevisiae) | other | 4,86E-12 | -1,443 | 4,86E-12 | -1,752 | 4,86E-12 | -1,306 |
| 202737_s_at | LSM4 | LSM4 homolog, U6 small nuclear RNA associated (S. cerevisiae) | other | 3,80E-12 | -1,435 | 3,80E-12 | -1,672 | 3,80E-12 | -1,283 |
| 202738_s_at | PHKB | phosphorylase kinase, beta | kinase |  |  | 6,45E-08 | -1,590 |  |  |
| 202739_s_at | PHKB | phosphorylase kinase, beta | kinase | 5,78E-10 | -1,778 | 5,78E-10 | -1,581 | 5,78E-10 | -1,596 |
| 202740_at | ACY1 | aminoacylase 1 | peptidase |  |  | 1,83E-14 | -2,188 |  |  |
| 202745_at | USP8 | ubiquitin specific peptidase 8 | peptidase |  |  | 7,41E-08 | -1,635 |  |  |
| 202749_at | WRB | tryptophan rich basic protein | other |  |  | 2,23E-08 | -1,560 |  |  |
| 202757_at | COBRA1 | cofactor of BRCA1 | other |  |  | 1,23E-09 | -1,774 |  |  |
| 202769_at | CCNG2 | cyclin G2 | other | 7,57E-11 | -1,654 | 7,57E-11 | -1,502 | 7,57E-11 | -1,483 |
| 202770_s_at | CCNG2 | cyclin G2 | other | 3,47E-13 | -1,788 | 3,47E-13 | -1,659 | 3,47E-13 | -1,846 |
| 202771_at | FAM38A | family with sequence similarity 38, member A | other |  |  | 2,08E-10 | -1,745 |  |  |
| 202776_at | DNTTIP2 | deoxynucleotidyltransferase, terminal, interacting protein 2 | other |  |  | 1,39E-13 | -1,880 |  |  |
| 202780_at | OXCT1 | 3-oxoacid CoA transferase 1 | enzyme |  |  | 4,89E-09 | -2,216 | 4,89E-09 | -1,603 |
| 202783_at | NNT | nicotinamide nucleotide transhydrogenase | enzyme | 4,75E-09 | -1,399 | 4,75E-09 | -1,597 | 4,75E-09 | -1,636 |
| 202802_at | DHPS | deoxyhypusine synthase | enzyme |  |  | 8,31E-08 | -2,065 |  |  |
| 202804_at | ABCC1 | ATP-binding cassette, sub-family C (CFTR/MRP), member 1 | transporter |  |  | 1,90E-09 | -1,903 |  |  |
| 202806_at | DBN1 | drebrin 1 | other |  |  | 3,39E-08 | -2,185 |  |  |
| 202809_s_at | INTS3 | integrator complex subunit 3 | other |  |  | 1,46E-09 | -1,980 |  |  |
| 202814_s_at | HEXIM1 | hexamethylene bis-acetamide inducible 1 | transcription regulator | 1,23E-09 | -1,432 | 1,23E-09 | -1,552 | 1,23E-09 | -1,456 |
| 202826_at | SPINT1 | serine peptidase inhibitor, Kunitz type 1 | other |  |  | 1,02E-12 | -1,960 |  |  |
| 202829_s_at | VAMP7 | vesicle-associated membrane protein 7 | transporter |  |  | 6,86E-09 | -1,471 |  |  |
| 202830_s_at | SLC37A4 | solute carrier family 37 (glucose-6-phosphate transporter), member 4 | transporter |  |  | 1,61E-08 | -1,764 | 1,61E-08 | -1,410 |
| 202832_at | GCC2 | GRIP and coiled-coil domain containing 2 | other |  |  | 2,01E-07 | -1,437 |  |  |
| 202833_s_at | SERPINA1 | serpin peptidase inhibitor, clade A (alpha-1 antiproteinase, antitrypsin), member 1 | other |  |  | 2,38E-11 | -1,685 |  |  |
| 202836_s_at | TXNL4A (includes EG:10907) | thioredoxin-like 4A | enzyme |  |  | 3,43E-09 | -1,600 |  |  |
| 202839_s_at | NDUFB7 | NADH dehydrogenase (ubiquinone) 1 beta subcomplex, 7, 18kDa | enzyme |  |  | 3,43E-08 | -1,691 |  |  |
| 202867_s_at | DNAJB12 | DnaJ (Hsp40) homolog, subfamily B, member 12 | other | 1,40E-10 | -1,560 | 1,40E-10 | -2,158 |  |  |
| 202870_s_at | CDC20 | cell division cycle 20 homolog (S. cerevisiae) | other |  |  | 5,46E-08 | -1,835 |  |  |
| 202894_at | EPHB4 | EPH receptor B4 | kinase |  |  | 2,61E-12 | -2,608 |  |  |
| 202900_s_at | NUP88 | nucleoporin 88kDa | transporter |  |  | 2,57E-09 | -1,621 |  |  |
| 202903_at | LSM5 | LSM5 homolog, U6 small nuclear RNA associated (S. cerevisiae) | other | 1,83E-14 | -2,222 | 1,83E-14 | -1,852 | 1,83E-14 | -1,930 |
| 202904_s_at | LSM5 | LSM5 homolog, U6 small nuclear RNA associated (S. cerevisiae) | other | 1,83E-14 | -1,433 | 1,83E-14 | -1,806 | 1,83E-14 | -1,588 |
| 202909_at | EPM2AIP1 | EPM2A (laforin) interacting protein 1 | other |  |  | 3,72E-12 | -1,933 |  |  |
| 202918_s_at | MOBKL3 | MOB1, Mps One Binder kinase activator-like 3 (yeast) | other |  |  | 1,90E-07 | -1,453 |  |  |
| 202922_at | GCLC | glutamate-cysteine ligase, catalytic subunit | enzyme |  |  |  |  | 1,15E-09 | 1,355 |
| 202925_s_at | PLAGL2 | pleiomorphic adenoma gene-like 2 | transcription regulator |  |  | 1,60E-10 | -1,785 |  |  |
| 202929_s_at | DDT | D-dopachrome tautomerase | enzyme |  |  | 4,75E-09 | -1,678 | 4,75E-09 | -1,527 |
| 202930_s_at | SUCLA2 | succinate-CoA ligase, ADP-forming, beta subunit | enzyme | 1,83E-14 | -1,298 | 1,83E-14 | -1,742 | 1,83E-14 | -1,494 |
| 202935_s_at | SOX9 | SRY (sex determining region Y)-box 9 | transcription regulator |  |  |  |  | 7,55E-14 | 1,726 |
| 202941_at | NDUFV2 | NADH dehydrogenase (ubiquinone) flavoprotein 2, 24kDa | enzyme |  |  | 7,70E-12 | -1,764 |  |  |
| 202943_s_at | NAGA | N-acetylgalactosaminidase, alpha- | enzyme |  |  | 4,32E-09 | -1,934 |  |  |
| 202946_s_at | BTBD3 | BTB (POZ) domain containing 3 | other |  |  | 1,17E-08 | -1,812 |  |  |
| 202951_at | STK38 | serine/threonine kinase 38 | kinase | 2,65E-09 | -1,512 | 2,65E-09 | -1,537 | 2,65E-09 | -1,312 |
| 202959_at | MUT | methylmalonyl Coenzyme A mutase | enzyme | 2,76E-09 | -1,644 | 2,76E-09 | -1,836 | 2,76E-09 | -1,514 |
| 202961_s_at | ATP5J2 | ATP synthase, H+ transporting, mitochondrial F0 complex, subunit F2 | transporter |  |  | 4,40E-12 | -1,836 |  |  |
| 202982_s_at | ACOT2 | acyl-CoA thioesterase 2 | enzyme | 1,83E-14 | -1,432 | 1,83E-14 | -2,383 | 1,83E-14 | -1,567 |
| 202985_s_at | BAG5 | BCL2-associated athanogene 5 | other |  |  | 5,61E-08 | -1,587 |  |  |
| 202990_at | PYGL | phosphorylase, glycogen, liver | enzyme |  |  | 8,32E-10 | -1,642 |  |  |
| 202993_at | ILVBL | ilvB (bacterial acetolactate synthase)-like | enzyme | 1,87E-09 | -1,675 | 1,87E-09 | -2,256 | 1,87E-09 | -1,867 |
| 203002_at | AMOTL2 | angiomotin like 2 | other | 2,47E-08 | -1,524 |  |  | 2,47E-08 | -1,385 |
| 203005_at | LTBR | lymphotoxin beta receptor (TNFR superfamily, member 3) | transmembrane receptor |  |  | 2,05E-10 | -2,381 |  |  |
| 203006_at | INPP5A | inositol polyphosphate-5-phosphatase, 40kDa | phosphatase |  |  | 9,89E-11 | -1,702 |  |  |
| 203022_at | RNASEH2A | ribonuclease H2, subunit A | enzyme |  |  | 2,62E-08 | -1,601 |  |  |
| 203025_at | ARD1A | ARD1 homolog A, N-acetyltransferase (S. cerevisiae) | enzyme |  |  | 3,73E-09 | -1,900 |  |  |
| 203028_s_at | CYBA | cytochrome b-245, alpha polypeptide | enzyme |  |  | 3,45E-09 | -1,825 |  |  |
| 203041_s_at | LAMP2 | lysosomal-associated membrane protein 2 | enzyme | 5,46E-10 | -1,491 | 5,46E-10 | -1,523 |  |  |
| 203043_at | ZBED1 | zinc finger, BED-type containing 1 | enzyme | 1,88E-10 | -1,717 | 1,88E-10 | -1,935 | 1,88E-10 | -1,542 |
| 203053_at | BCAS2 | breast carcinoma amplified sequence 2 | other | 1,70E-11 | -1,586 | 1,70E-11 | -1,658 |  |  |
| 203089_s_at | HTRA2 | HtrA serine peptidase 2 | peptidase |  |  | 5,54E-08 | -1,728 |  |  |
| 203093_s_at | TIMM44 | translocase of inner mitochondrial membrane 44 homolog (yeast) | transporter |  |  | 9,65E-09 | -2,437 |  |  |
| 203095_at | MTIF2 | mitochondrial translational initiation factor 2 | translation regulator |  |  | 1,87E-07 | -1,502 |  |  |
| 203097_s_at | RAPGEF2 | Rap guanine nucleotide exchange factor (GEF) 2 | other | 5,93E-11 | -1,489 | 5,93E-11 | -1,508 |  |  |
| 203109_at | UBE2M | ubiquitin-conjugating enzyme E2M (UBC12 homolog, yeast) | enzyme |  |  | 8,59E-08 | -1,753 |  |  |
| 203113_s_at | EEF1D | eukaryotic translation elongation factor 1 delta (guanine nucleotide exchange protein) | translation regulator | 1,83E-14 | -1,460 | 1,83E-14 | -1,839 | 1,83E-14 | -1,373 |
| 203120_at | TP53BP2 | tumor protein p53 binding protein, 2 | other | 6,55E-12 | -1,653 | 6,55E-12 | -2,056 | 6,55E-12 | -1,520 |
| 203136_at | RABAC1 | Rab acceptor 1 (prenylated) | other | 4,26E-09 | -1,449 | 4,26E-09 | -1,905 |  |  |
| 203139_at | DAPK1 | death-associated protein kinase 1 | kinase | 6,21E-11 | -1,671 | 6,21E-11 | -1,804 | 6,21E-11 | -1,644 |
| 203140_at | BCL6 | B-cell CLL/lymphoma 6 | transcription regulator | 1,85E-07 | -1,593 |  |  |  |  |
| 203152_at | MRPL40 | mitochondrial ribosomal protein L40 | other |  |  | 6,56E-09 | -1,684 |  |  |
| 203155_at | SETDB1 | SET domain, bifurcated 1 | enzyme |  |  | 7,60E-09 | -2,495 |  |  |
| 203164_at | SLC33A1 | solute carrier family 33 (acetyl-CoA transporter), member 1 | transporter | 3,65E-10 | -1,543 | 3,65E-10 | -1,810 |  |  |
| 203182_s_at | SRPK2 | SFRS protein kinase 2 | kinase |  |  | 6,83E-09 | -1,660 |  |  |
| 203187_at | DOCK1 | dedicator of cytokinesis 1 | other |  |  | 1,59E-08 | -1,639 | 1,59E-08 | -1,697 |
| 203188_at | B3GNT1 | UDP-GlcNAc:betaGal beta-1,3-N-acetylglucosaminyltransferase 1 | enzyme | 2,43E-13 | -1,593 | 2,43E-13 | -1,923 | 2,43E-13 | -1,707 |
| 203189_s_at | NDUFS8 | NADH dehydrogenase (ubiquinone) Fe-S protein 8, 23kDa (NADH-coenzyme Q reductase) | enzyme |  |  | 2,87E-08 | -1,704 |  |  |
| 203190_at | NDUFS8 | NADH dehydrogenase (ubiquinone) Fe-S protein 8, 23kDa (NADH-coenzyme Q reductase) | enzyme |  |  | 6,39E-11 | -1,872 |  |  |
| 203192_at | ABCB6 | ATP-binding cassette, sub-family B (MDR/TAP), member 6 | transporter |  |  | 1,83E-09 | -1,851 |  |  |
| 203200_s_at | MTRR | 5-methyltetrahydrofolate-homocysteine methyltransferase reductase | enzyme |  |  | 1,09E-08 | -1,529 |  |  |
| 203201_at | PMM2 | phosphomannomutase 2 | enzyme |  |  | 3,77E-09 | -2,004 |  |  |
| 203207_s_at | MTFR1 | mitochondrial fission regulator 1 | other |  |  | 4,61E-08 | -1,712 |  |  |
| 203209_at | RFC5 | replication factor C (activator 1) 5, 36.5kDa | enzyme | 5,42E-10 | -1,376 | 5,42E-10 | -1,534 |  |  |
| 203210_s_at | RFC5 | replication factor C (activator 1) 5, 36.5kDa | enzyme |  |  | 6,84E-09 | -1,723 |  |  |
| 203219_s_at | APRT | adenine phosphoribosyltransferase | enzyme |  |  | 3,25E-11 | -1,919 |  |  |
| 203228_at | PAFAH1B3 | platelet-activating factor acetylhydrolase, isoform Ib, gamma subunit 29kDa | enzyme |  |  | 1,05E-07 | -1,883 |  |  |
| 203241_at | UVRAG | UV radiation resistance associated gene | other |  |  | 4,48E-09 | -1,771 |  |  |
| 203245_s_at | NCRNA00094 | non-protein coding RNA 94 | other |  |  | 8,68E-10 | -2,237 |  |  |
| 203250_at | RBM16 | RNA binding motif protein 16 | other |  |  | 1,01E-12 | -1,766 |  |  |
| 203259_s_at | HDDC2 | HD domain containing 2 | other |  |  | 2,83E-10 | -1,655 |  |  |
| 203261_at | DCTN6 | dynactin 6 | enzyme |  |  | 3,56E-08 | -1,619 |  |  |
| 203272_s_at | TUSC2 | tumor suppressor candidate 2 | other | 2,31E-10 | -1,614 | 2,31E-10 | -2,033 |  |  |
| 203274_at | F8A1 | coagulation factor VIII-associated (intronic transcript) 1 | other |  |  | 9,15E-09 | -1,744 |  |  |
| 203275_at | IRF2 | interferon regulatory factor 2 | transcription regulator | 1,83E-14 | -2,151 | 1,83E-14 | -2,543 |  |  |
| 203284_s_at | HS2ST1 | heparan sulfate 2-O-sulfotransferase 1 | enzyme | 2,46E-10 | -1,391 | 2,46E-10 | -1,524 |  |  |
| 203302_at | DCK | deoxycytidine kinase | kinase |  |  | 2,75E-10 | -1,821 |  |  |
| 203304_at | BAMBI | BMP and activin membrane-bound inhibitor homolog (Xenopus laevis) | other | 2,73E-12 | -1,932 | 2,73E-12 | -1,753 | 2,73E-12 | -1,893 |
| 203314_at | GTPBP6 | GTP binding protein 6 (putative) | other |  |  | 1,20E-11 | -2,050 |  |  |
| 203316_s_at | SNRPE | small nuclear ribonucleoprotein polypeptide E | other |  |  | 1,53E-07 | -1,614 |  |  |
| 203322_at | ADNP2 | ADNP homeobox 2 | other | 1,94E-11 | -1,621 | 1,94E-11 | -1,983 | 1,94E-11 | -1,413 |
| 203327_at | IDE | insulin-degrading enzyme | peptidase |  |  | 3,68E-12 | -1,740 |  |  |
| 203328_x_at | IDE | insulin-degrading enzyme | peptidase | 2,16E-13 | -1,511 | 2,16E-13 | -1,933 | 2,16E-13 | -1,476 |
| 203336_s_at | ITGB1BP1 | integrin beta 1 binding protein 1 | other |  |  | 1,96E-08 | -1,676 |  |  |
| 203338_at | PPP2R5E | protein phosphatase 2, regulatory subunit B', epsilon isoform | phosphatase |  |  | 4,83E-10 | -1,640 |  |  |
| 203344_s_at | RBBP8 (includes EG:5932) | retinoblastoma binding protein 8 | other |  |  | 9,09E-12 | -1,737 |  |  |
| 203347_s_at | MTF2 | metal response element binding transcription factor 2 | transcription regulator |  |  | 1,06E-08 | -1,949 |  |  |
| 203350_at | AP1G1 | adaptor-related protein complex 1, gamma 1 subunit | transporter | 1,28E-10 | -1,580 | 1,28E-10 | -1,482 | 1,28E-10 | -1,450 |
| 203359_s_at | MYCBP | c-myc binding protein | transcription regulator | 3,66E-11 | -1,451 | 3,66E-11 | -1,635 |  |  |
| 203360_s_at | MYCBP | c-myc binding protein | transcription regulator |  |  | 8,91E-09 | -1,693 |  |  |
| 203363_s_at | KIAA0652 | KIAA0652 | other |  |  | 5,25E-09 | -2,105 | 5,25E-09 | -1,988 |
| 203364_s_at | KIAA0652 | KIAA0652 | other | 4,52E-10 | -1,636 | 4,52E-10 | -1,852 |  |  |
| 203372_s_at | SOCS2 | suppressor of cytokine signaling 2 | other |  |  | 4,78E-09 | -1,777 |  |  |
| 203373_at | SOCS2 | suppressor of cytokine signaling 2 | other | 1,01E-09 | -1,454 | 1,01E-09 | -1,532 |  |  |
| 203376_at | CDC40 | cell division cycle 40 homolog (S. cerevisiae) | other | 3,64E-13 | -1,591 | 3,64E-13 | -1,705 | 3,64E-13 | -1,434 |
| 203380_x_at | SFRS5 | splicing factor, arginine/serine-rich 5 | other |  |  | 2,89E-09 | -1,782 |  |  |
| 203381_s_at | APOE | apolipoprotein E | transporter |  |  | 1,44E-08 | -1,764 |  |  |
| 203382_s_at | APOE | apolipoprotein E | transporter |  |  | 3,29E-13 | -2,196 | 3,29E-13 | -1,668 |
| 203391_at | FKBP2 | FK506 binding protein 2, 13kDa | enzyme |  |  | 4,64E-08 | -2,404 |  |  |
| 203392_s_at | CTBP1 | C-terminal binding protein 1 | enzyme |  |  | 2,01E-12 | -1,838 |  |  |
| 203395_s_at | HES1 | hairy and enhancer of split 1, (Drosophila) | transcription regulator | 7,55E-14 | -1,994 |  |  |  |  |
| 203401_at | PRPS2 | phosphoribosyl pyrophosphate synthetase 2 | kinase | 1,67E-11 | -1,389 | 1,67E-11 | -1,683 | 1,67E-11 | -1,315 |
| 203403_s_at | RNF6 | ring finger protein (C3H2C3 type) 6 | transcription regulator |  |  | 1,96E-10 | -1,645 |  |  |
| 203405_at | PSMG1 | proteasome (prosome, macropain) assembly chaperone 1 | other |  |  |  |  | 5,26E-08 | -1,410 |
| 203406_at | MFAP1 | microfibrillar-associated protein 1 | other | 1,02E-09 | -1,421 | 1,02E-09 | -1,486 |  |  |
| 203407_at | PPL | periplakin | other |  |  | 6,16E-08 | -1,684 | 6,16E-08 | -1,412 |
| 203411_s_at | LMNA | lamin A/C | other |  |  | 7,40E-09 | -1,865 |  |  |
| 203418_at | CCNA2 | cyclin A2 | other |  |  | 9,08E-09 | -1,577 |  |  |
| 203427_at | ASF1A | ASF1 anti-silencing function 1 homolog A (S. cerevisiae) | other |  |  | 1,38E-08 | -1,677 |  |  |
| 203428_s_at | ASF1A | ASF1 anti-silencing function 1 homolog A (S. cerevisiae) | other |  |  | 1,27E-08 | -1,633 |  |  |
| 203430_at | HEBP2 | heme binding protein 2 | other |  |  | 1,69E-08 | -1,598 |  |  |
| 203432_at | TMPO | thymopoietin | other |  |  | 2,51E-08 | -1,506 |  |  |
| 203433_at | MTHFS | 5,10-methenyltetrahydrofolate synthetase (5-formyltetrahydrofolate cyclo-ligase) | enzyme |  |  | 1,09E-08 | -1,929 |  |  |
| 203437_at | TMEM11 | transmembrane protein 11 | G-protein coupled receptor |  |  | 3,58E-07 | -1,581 |  |  |
| 203447_at | PSMD5 | proteasome (prosome, macropain) 26S subunit, non-ATPase, 5 | other |  |  | 2,50E-08 | -1,697 |  |  |
| 203454_s_at | ATOX1 | ATX1 antioxidant protein 1 homolog (yeast) | transporter | 7,35E-13 | -1,400 | 7,35E-13 | -1,942 | 7,35E-13 | -1,507 |
| 203455_s_at | SAT1 | spermidine/spermine N1-acetyltransferase 1 | enzyme | 1,79E-08 | -1,396 | 1,79E-08 | -1,495 |  |  |
| 203458_at | SPR | sepiapterin reductase (7,8-dihydrobiopterin:NADP+ oxidoreductase) | enzyme |  |  | 8,10E-09 | -1,763 |  |  |
| 203462_x_at | EIF3B | eukaryotic translation initiation factor 3, subunit B | translation regulator |  |  | 7,38E-10 | -1,648 |  |  |
| 203473_at | SLCO2B1 | solute carrier organic anion transporter family, member 2B1 | transporter |  |  |  |  | 6,12E-08 | -1,807 |
| 203474_at | IQGAP2 | IQ motif containing GTPase activating protein 2 | other | 4,19E-12 | -1,522 | 4,19E-12 | -1,771 | 4,19E-12 | -1,653 |
| 203481_at | FAM178A | family with sequence similarity 178, member A | other | 9,61E-10 | -1,398 | 9,61E-10 | -1,633 | 9,61E-10 | -1,658 |
| 203489_at | SIVA1 | SIVA1, apoptosis-inducing factor | other | 3,53E-11 | -1,548 | 3,53E-11 | -1,807 | 3,53E-11 | -1,546 |
| 203493_s_at | CEP57 | centrosomal protein 57kDa | other | 9,58E-11 | -1,432 | 9,58E-11 | -1,616 | 9,58E-11 | -1,319 |
| 203494_s_at | CEP57 | centrosomal protein 57kDa | other | 4,87E-14 | -1,582 | 4,87E-14 | -1,781 | 4,87E-14 | -1,430 |
| 203513_at | SPG11 (includes EG:80208) | spastic paraplegia 11 (autosomal recessive) | other |  |  | 5,09E-10 | -1,710 |  |  |
| 203524_s_at | MPST | mercaptopyruvate sulfurtransferase | enzyme |  |  | 9,30E-08 | -1,913 |  |  |
| 203529_at | PPP6C | protein phosphatase 6, catalytic subunit | phosphatase |  |  | 9,45E-13 | -1,834 |  |  |
| 203537_at | PRPSAP2 | phosphoribosyl pyrophosphate synthetase-associated protein 2 | other |  |  | 2,36E-08 | -1,594 |  |  |
| 203538_at | CAMLG | calcium modulating ligand | other |  |  | 2,44E-07 | -1,525 |  |  |
| 203544_s_at | STAM | signal transducing adaptor molecule (SH3 domain and ITAM motif) 1 | other |  |  | 3,96E-09 | -1,595 |  |  |
| 203553_s_at | MAP4K5 | mitogen-activated protein kinase kinase kinase kinase 5 | kinase |  |  | 3,93E-07 | -1,591 |  |  |
| 203554_x_at | PTTG1 | pituitary tumor-transforming 1 | transcription regulator |  |  | 8,02E-09 | -1,525 |  |  |
| 203557_s_at | PCBD1 | pterin-4 alpha-carbinolamine dehydratase/dimerization cofactor of hepatocyte nuclear factor 1 alpha | transcription regulator |  |  | 2,98E-10 | -1,659 |  |  |
| 203560_at | GGH | gamma-glutamyl hydrolase (conjugase, folylpolygammaglutamyl hydrolase) | peptidase |  |  | 1,70E-08 | -1,509 |  |  |
| 203564_at | VCP | valosin-containing protein | enzyme | 7,91E-09 | -1,399 | 7,91E-09 | -1,725 |  |  |
| 203566_s_at | AGL | amylo-1, 6-glucosidase, 4-alpha-glucanotransferase | enzyme | 6,14E-13 | -1,506 | 6,14E-13 | -1,540 | 6,14E-13 | -1,465 |
| 203572_s_at | TAF6 | TAF6 RNA polymerase II, TATA box binding protein (TBP)-associated factor, 80kDa | transcription regulator |  |  | 3,46E-10 | -1,816 |  |  |
| 203575_at | CSNK2A2 | casein kinase 2, alpha prime polypeptide | kinase |  |  | 4,18E-08 | -1,742 |  |  |
| 203576_at | BCAT2 | branched chain aminotransferase 2, mitochondrial | enzyme |  |  | 1,07E-12 | -2,060 |  |  |
| 203578_s_at | TRPV6 | transient receptor potential cation channel, subfamily V, member 6 | ion channel |  |  | 8,60E-08 | -1,629 |  |  |
| 203580_s_at | TRPV6 | transient receptor potential cation channel, subfamily V, member 6 | ion channel | 2,60E-11 | -1,615 | 2,60E-11 | -1,581 |  |  |
| 203582_s_at | RAB4A | RAB4A, member RAS oncogene family | enzyme | 2,96E-12 | -1,409 | 2,96E-12 | -1,782 | 2,96E-12 | -1,408 |
| 203583_at | UNC50 | unc-50 homolog (C. elegans) | other | 2,05E-12 | -1,466 | 2,05E-12 | -1,748 | 2,05E-12 | -1,449 |
| 203588_s_at | TFDP2 | transcription factor Dp-2 (E2F dimerization partner 2) | transcription regulator | 1,65E-11 | -1,489 | 1,65E-11 | -2,157 |  |  |
| 203593_at | CD2AP | CD2-associated protein | other | 4,87E-14 | -1,581 | 4,87E-14 | -1,835 |  |  |
| 203608_at | ALDH5A1 | aldehyde dehydrogenase 5 family, member A1 | enzyme | 1,28E-12 | -1,551 | 1,28E-12 | -1,650 | 1,28E-12 | -1,380 |
| 203611_at | TERF2 | telomeric repeat binding factor 2 | other |  |  | 1,28E-08 | -1,672 |  |  |
| 203613_s_at | NDUFB6 | NADH dehydrogenase (ubiquinone) 1 beta subcomplex, 6, 17kDa | enzyme | 3,44E-14 | -1,562 | 3,44E-14 | -1,902 | 3,44E-14 | -1,543 |
| 203632_s_at | GPRC5B | G protein-coupled receptor, family C, group 5, member B | G-protein coupled receptor | 1,83E-14 | -1,618 | 1,83E-14 | -1,983 | 1,83E-14 | -1,795 |
| 203633_at | CPT1A | carnitine palmitoyltransferase 1A (liver) | enzyme | 1,50E-13 | 4,247 |  |  | 1,50E-13 | 3,677 |
| 203638_s_at | FGFR2 | fibroblast growth factor receptor 2 | kinase | 2,04E-10 | -2,551 | 2,04E-10 | -2,095 | 2,04E-10 | -2,996 |
| 203642_s_at | COBLL1 | COBL-like 1 | other | 1,50E-08 | -1,499 |  |  | 1,50E-08 | -1,387 |
| 203646_at | FDX1 | ferredoxin 1 | transporter |  |  | 3,46E-08 | -1,646 |  |  |
| 203647_s_at | FDX1 | ferredoxin 1 | transporter |  |  | 5,31E-08 | -1,601 |  |  |
| 203648_at | TATDN2 | TatD DNase domain containing 2 | other |  |  | 8,03E-08 | -1,823 |  |  |
| 203649_s_at | PLA2G2A | phospholipase A2, group IIA (platelets, synovial fluid) | enzyme |  |  | 4,17E-10 | -1,895 | 4,17E-10 | -1,668 |
| 203650_at | PROCR | protein C receptor, endothelial (EPCR) | other |  |  | 2,79E-09 | -1,661 |  |  |
| 203651_at | ZFYVE16 | zinc finger, FYVE domain containing 16 | transporter | 5,88E-13 | -1,569 | 5,88E-13 | -1,511 |  |  |
| 203659_s_at | TRIM13 | tripartite motif-containing 13 | other |  |  | 6,85E-09 | -1,678 |  |  |
| 203660_s_at | PCNT | pericentrin | other |  |  | 1,35E-08 | -1,787 |  |  |
| 203663_s_at | COX5A | cytochrome c oxidase subunit Va | enzyme |  |  | 1,98E-08 | -1,498 |  |  |
| 203664_s_at | POLR2D | polymerase (RNA) II (DNA directed) polypeptide D | enzyme |  |  | 7,95E-11 | -1,761 | 7,95E-11 | -1,330 |
| 203675_at | NUCB2 | nucleobindin 2 | other | 1,83E-14 | -1,814 | 1,83E-14 | -1,872 | 1,83E-14 | -1,431 |
| 203695_s_at | DFNA5 | deafness, autosomal dominant 5 | other | 2,50E-08 | -1,567 |  |  |  |  |
| 203706_s_at | FZD7 | frizzled homolog 7 (Drosophila) | G-protein coupled receptor |  |  | 1,72E-08 | -1,581 |  |  |
| 203717_at | DPP4 | dipeptidyl-peptidase 4 | peptidase |  |  | 7,36E-11 | -1,785 |  |  |
| 203721_s_at | UTP18 | UTP18, small subunit (SSU) processome component, homolog (yeast) | other |  |  | 9,43E-09 | -1,536 |  |  |
| 203737_s_at | PPRC1 | peroxisome proliferator-activated receptor gamma, coactivator-related 1 | other |  |  | 1,10E-07 | -1,655 |  |  |
| 203738_at | C5ORF22 | chromosome 5 open reading frame 22 | other |  |  | 4,47E-12 | -1,765 |  |  |
| 203746_s_at | HCCS | holocytochrome c synthase (cytochrome c heme-lyase) | enzyme |  |  | 6,48E-09 | -1,779 |  |  |
| 203752_s_at | JUND | jun D proto-oncogene | transcription regulator | 5,10E-10 | -1,485 | 5,10E-10 | -1,574 |  |  |
| 203753_at | TCF4 | transcription factor 4 | transcription regulator | 1,09E-10 | -1,661 | 1,09E-10 | -1,828 | 1,09E-10 | -2,095 |
| 203755_at | BUB1B | budding uninhibited by benzimidazoles 1 homolog beta (yeast) | kinase |  |  | 4,74E-09 | -1,677 |  |  |
| 203757_s_at | CEACAM6 (includes EG:4680) | carcinoembryonic antigen-related cell adhesion molecule 6 (non-specific cross reacting antigen) | other |  |  |  |  | 1,83E-14 | 1,603 |
| 203764_at | DLGAP5 | discs, large (Drosophila) homolog-associated protein 5 | phosphatase | 1,83E-14 | -1,372 | 1,83E-14 | -1,927 | 1,83E-14 | -1,302 |
| 203765_at | GCA | grancalcin, EF-hand calcium binding protein | other | 1,33E-08 | -1,501 | 1,33E-08 | -1,619 | 1,33E-08 | -1,388 |
| 203767_s_at | STS | steroid sulfatase (microsomal), isozyme S | enzyme |  |  | 3,00E-08 | -1,476 |  |  |
| 203775_at | SLC25A13 | solute carrier family 25, member 13 (citrin) | transporter |  |  | 8,25E-12 | -1,744 |  |  |
| 203786_s_at | TPD52L1 | tumor protein D52-like 1 | other | 3,07E-11 | -1,398 | 3,07E-11 | -1,705 | 3,07E-11 | -1,422 |
| 203790_s_at | HRSP12 | heat-responsive protein 12 | other | 3,09E-13 | -1,310 | 3,09E-13 | -1,913 |  |  |
| 203802_x_at | NSUN5 | NOL1/NOP2/Sun domain family, member 5 | other |  |  | 7,86E-10 | -1,649 |  |  |
| 203820_s_at | IGF2BP3 | insulin-like growth factor 2 mRNA binding protein 3 | other | 1,44E-11 | -1,369 | 1,44E-11 | -1,569 | 1,44E-11 | -1,313 |
| 203823_at | RGS3 | regulator of G-protein signaling 3 | other | 1,06E-08 | -1,778 |  |  | 1,06E-08 | -1,961 |
| 203824_at | TSPAN8 | tetraspanin 8 | other |  |  | 1,84E-08 | -1,522 |  |  |
| 203851_at | IGFBP6 | insulin-like growth factor binding protein 6 | other | 2,04E-09 | -1,616 | 2,04E-09 | -2,253 |  |  |
| 203852_s_at | SMN1 | survival of motor neuron 1, telomeric | other | 4,41E-13 | -1,524 | 4,41E-13 | -1,957 | 4,41E-13 | -1,399 |
| 203857_s_at | PDIA5 | protein disulfide isomerase family A, member 5 | enzyme |  |  | 1,28E-08 | -1,657 |  |  |
| 203869_at | USP46 | ubiquitin specific peptidase 46 | peptidase |  |  | 3,78E-09 | -1,800 |  |  |
| 203871_at | SENP3 | SUMO1/sentrin/SMT3 specific peptidase 3 | peptidase |  |  | 4,18E-11 | -2,204 |  |  |
| 203882_at | IRF9 | interferon regulatory factor 9 | transcription regulator | 5,54E-08 | -2,077 |  |  |  |  |
| 203883_s_at | RAB11FIP2 | RAB11 family interacting protein 2 (class I) | other | 6,22E-10 | -1,507 | 6,22E-10 | -1,609 |  |  |
| 203884_s_at | RAB11FIP2 | RAB11 family interacting protein 2 (class I) | other | 1,67E-10 | -1,564 | 1,67E-10 | -1,748 | 1,67E-10 | -1,375 |
| 203885_at | RAB21 | RAB21, member RAS oncogene family | enzyme |  |  | 1,92E-11 | -1,670 |  |  |
| 203897_at | LYRM1 | LYR motif containing 1 | other | 1,33E-11 | -1,453 | 1,33E-11 | -1,703 |  |  |
| 203903_s_at | HEPH | hephaestin | transporter |  |  | 4,37E-08 | -1,514 |  |  |
| 203909_at | SLC9A6 | solute carrier family 9 (sodium/hydrogen exchanger), member 6 | transporter |  |  | 2,65E-10 | -1,783 |  |  |
| 203910_at | ARHGAP29 | Rho GTPase activating protein 29 | other |  |  | 1,28E-11 | -1,598 |  |  |
| 203913_s_at | HPGD | hydroxyprostaglandin dehydrogenase 15-(NAD) | enzyme |  |  | 1,67E-12 | -1,392 | 1,67E-12 | -1,816 |
| 203914_x_at | HPGD | hydroxyprostaglandin dehydrogenase 15-(NAD) | enzyme | 1,62E-12 | -1,738 | 1,62E-12 | -1,565 | 1,62E-12 | -1,495 |
| 203924_at | GSTA1 | glutathione S-transferase alpha 1 | enzyme |  |  |  |  | 1,31E-10 | -2,049 |
| 203925_at | GCLM | glutamate-cysteine ligase, modifier subunit | enzyme |  |  | 1,83E-14 | -1,952 |  |  |
| 203943_at | KIF3B | kinesin family member 3B | transporter | 6,85E-12 | -1,385 | 6,85E-12 | -1,699 |  |  |
| 203946_s_at | ARG2 | arginase, type II | enzyme | 2,84E-12 | -1,780 | 2,84E-12 | -1,579 | 2,84E-12 | -1,541 |
| 203960_s_at | HSPB11 | heat shock protein family B (small), member 11 | other | 6,25E-11 | -1,499 | 6,25E-11 | -1,794 | 6,25E-11 | -1,518 |
| 203964_at | NMI | N-myc (and STAT) interactor | transcription regulator | 3,20E-09 | -1,496 |  |  |  |  |
| 203970_s_at | PEX3 | peroxisomal biogenesis factor 3 | other | 3,44E-14 | -1,628 | 3,44E-14 | -1,820 | 3,44E-14 | -1,471 |
| 203972_s_at | PEX3 | peroxisomal biogenesis factor 3 | other |  |  | 7,64E-08 | -1,610 |  |  |
| 203973_s_at | CEBPD | CCAAT/enhancer binding protein (C/EBP), delta | transcription regulator | 3,99E-09 | -1,464 | 3,99E-09 | -1,563 |  |  |
| 203974_at | HDHD1A | haloacid dehalogenase-like hydrolase domain containing 1A | enzyme |  |  | 2,14E-08 | -1,634 |  |  |
| 203978_at | NUBP1 | nucleotide binding protein 1 (MinD homolog, E. coli) | other |  |  | 6,39E-09 | -1,760 | 6,39E-09 | -1,448 |
| 203983_at | TSNAX | translin-associated factor X | transporter | 5,78E-13 | -1,491 | 5,78E-13 | -1,826 | 5,78E-13 | -1,317 |
| 203984_s_at | CASP9 | caspase 9, apoptosis-related cysteine peptidase | peptidase |  |  |  |  | 5,25E-08 | -1,646 |
| 204009_s_at | KRAS | v-Ki-ras2 Kirsten rat sarcoma viral oncogene homolog | enzyme | 2,64E-10 | -1,361 | 2,64E-10 | -1,519 |  |  |
| 204023_at | RFC4 | replication factor C (activator 1) 4, 37kDa | other |  |  | 2,11E-08 | -1,466 |  |  |
| 204031_s_at | PCBP2 | poly(rC) binding protein 2 | other | 5,20E-12 | -1,450 | 5,20E-12 | -1,775 | 5,20E-12 | -1,472 |
| 204034_at | ETHE1 | ethylmalonic encephalopathy 1 | other |  |  | 5,63E-09 | -1,768 |  |  |
| 204039_at | CEBPA | CCAAT/enhancer binding protein (C/EBP), alpha | transcription regulator |  |  | 3,64E-13 | -2,064 |  |  |
| 204044_at | QPRT | quinolinate phosphoribosyltransferase | enzyme |  |  | 4,39E-08 | -1,521 |  |  |
| 204045_at | TCEAL1 | transcription elongation factor A (SII)-like 1 | transcription regulator | 6,12E-09 | -1,447 | 6,12E-09 | -1,764 | 6,12E-09 | -1,563 |
| 204057_at | IRF8 | interferon regulatory factor 8 | transcription regulator |  |  | 3,47E-08 | -1,707 |  |  |
| 204058_at | ME1 | malic enzyme 1, NADP(+)-dependent, cytosolic | enzyme |  |  | 4,22E-08 | -1,519 |  |  |
| 204067_at | SUOX | sulfite oxidase | enzyme |  |  | 1,04E-09 | -2,099 |  |  |
| 204076_at | ENTPD4 | ectonucleoside triphosphate diphosphohydrolase 4 | enzyme | 2,16E-09 | -1,526 | 2,16E-09 | -1,449 | 2,16E-09 | -1,356 |
| 204092_s_at | AURKA | aurora kinase A | kinase |  |  | 1,76E-09 | -1,774 |  |  |
| 204093_at | CCNH | cyclin H | transcription regulator | 3,29E-13 | -1,459 | 3,29E-13 | -1,911 |  |  |
| 204094_s_at | TSC22D2 | TSC22 domain family, member 2 | other |  |  | 2,43E-08 | -1,484 |  |  |
| 204098_at | RBMX2 | RNA binding motif protein, X-linked 2 | other | 4,87E-14 | -1,963 | 4,87E-14 | -2,375 | 4,87E-14 | -1,775 |
| 204112_s_at | HNMT | histamine N-methyltransferase | enzyme | 6,10E-10 | -1,444 | 6,10E-10 | -1,534 | 6,10E-10 | -1,464 |
| 204119_s_at | ADK | adenosine kinase | kinase | 3,34E-10 | -1,462 | 3,34E-10 | -1,645 |  |  |
| 204123_at | LIG3 | ligase III, DNA, ATP-dependent | enzyme |  |  | 1,28E-07 | -2,514 |  |  |
| 204126_s_at | CDC45L | CDC45 cell division cycle 45-like (S. cerevisiae) | other |  |  | 1,09E-08 | -2,183 |  |  |
| 204127_at | RFC3 | replication factor C (activator 1) 3, 38kDa | enzyme |  |  | 1,22E-08 | -1,548 |  |  |
| 204135_at | FILIP1L | filamin A interacting protein 1-like | other | 1,83E-14 | -1,898 | 1,83E-14 | -1,785 | 1,83E-14 | -1,995 |
| 204143_s_at | ENOSF1 | enolase superfamily member 1 | other |  |  | 5,73E-10 | -1,698 | 5,73E-10 | -1,330 |
| 204146_at | RAD51AP1 | RAD51 associated protein 1 | other | 6,92E-10 | -1,425 | 6,92E-10 | -1,533 | 6,92E-10 | -1,361 |
| 204149_s_at | GSTM4 | glutathione S-transferase mu 4 | enzyme |  |  | 3,57E-10 | -1,831 | 3,57E-10 | -1,747 |
| 204151_x_at | AKR1C1 | aldo-keto reductase family 1, member C1 (dihydrodiol dehydrogenase 1; 20-alpha (3-alpha)-hydroxysteroid dehydrogenase) | enzyme |  |  | 4,90E-11 | -1,540 |  |  |
| 204156_at | KIAA0999 | KIAA0999 protein | kinase | 2,46E-10 | -1,648 | 2,46E-10 | -1,806 |  |  |
| 204159_at | CDKN2C | cyclin-dependent kinase inhibitor 2C (p18, inhibits CDK4) | transcription regulator |  |  | 6,68E-07 | -1,980 |  |  |
| 204162_at | NDC80 | NDC80 homolog, kinetochore complex component (S. cerevisiae) | other | 8,83E-09 | -1,458 | 8,83E-09 | -1,529 | 8,83E-09 | -1,360 |
| 204168_at | MGST2 | microsomal glutathione S-transferase 2 | enzyme |  |  | 1,39E-13 | -1,988 |  |  |
| 204173_at | MYL6B | myosin, light chain 6B, alkali, smooth muscle and non-muscle | other |  |  | 2,10E-10 | -2,043 |  |  |
| 204175_at | ZNF593 | zinc finger protein 593 | transcription regulator |  |  | 1,64E-08 | -1,830 |  |  |
| 204178_s_at | RBM14 | RNA binding motif protein 14 | transcription regulator |  |  | 5,90E-09 | -1,847 | 5,90E-09 | -1,515 |
| 204186_s_at | PPID | peptidylprolyl isomerase D | enzyme |  |  | 2,79E-09 | -1,522 |  |  |
| 204215_at | C7ORF23 | chromosome 7 open reading frame 23 | other |  |  | 2,68E-09 | -1,633 |  |  |
| 204218_at | C11ORF51 | chromosome 11 open reading frame 51 | other |  |  | 5,01E-08 | -1,794 |  |  |
| 204235_s_at | GULP1 | GULP, engulfment adaptor PTB domain containing 1 | other |  |  |  |  | 1,50E-13 | 1,364 |
| 204240_s_at | SMC2 | structural maintenance of chromosomes 2 | transporter | 8,20E-10 | -1,411 | 8,20E-10 | -1,597 | 8,20E-10 | -1,341 |
| 204244_s_at | DBF4 | DBF4 homolog (S. cerevisiae) | kinase |  |  | 5,27E-08 | -1,540 |  |  |
| 204246_s_at | DCTN3 | dynactin 3 (p22) | other | 9,60E-13 | -1,302 | 9,60E-13 | -1,779 | 9,60E-13 | -1,413 |
| 204247_s_at | CDK5 | cyclin-dependent kinase 5 | kinase |  |  | 1,46E-07 | -1,885 |  |  |
| 204252_at | CDK2 | cyclin-dependent kinase 2 | kinase | 1,83E-14 | -1,761 | 1,83E-14 | -1,861 | 1,83E-14 | -1,468 |
| 204254_s_at | VDR | vitamin D (1,25- dihydroxyvitamin D3) receptor | ligand-dependent nuclear receptor |  |  | 3,62E-08 | -1,995 |  |  |
| 204266_s_at | CHKA | choline kinase alpha | kinase |  |  | 4,59E-10 | -1,773 |  |  |
| 204275_at | SOLH | small optic lobes homolog (Drosophila) | peptidase |  |  | 6,25E-09 | -2,084 |  |  |
| 204283_at | FARS2 | phenylalanyl-tRNA synthetase 2, mitochondrial | enzyme |  |  | 2,90E-10 | -2,037 |  |  |
| 204290_s_at | ALDH6A1 | aldehyde dehydrogenase 6 family, member A1 | enzyme | 4,35E-09 | -1,512 |  |  | 4,35E-09 | -1,833 |
| 204299_at | FUSIP1 | FUS interacting protein (serine/arginine-rich) 1 | other | 2,07E-08 | -1,338 | 2,07E-08 | -1,531 |  |  |
| 204304_s_at | PROM1 | prominin 1 | other | 3,45E-11 | -1,537 | 3,45E-11 | -1,491 | 3,45E-11 | -1,330 |
| 204326_x_at | MT1X | metallothionein 1X | other | 1,83E-14 | 6,064 |  |  | 1,83E-14 | 4,047 |
| 204350_s_at | MED7 (includes EG:9443) | mediator complex subunit 7 | transcription regulator |  |  | 1,91E-08 | -1,570 |  |  |
| 204361_s_at | SKAP2 | src kinase associated phosphoprotein 2 | other |  |  | 5,44E-08 | -1,476 | 5,44E-08 | -1,546 |
| 204362_at | SKAP2 | src kinase associated phosphoprotein 2 | other | 1,83E-14 | -1,692 | 1,83E-14 | -1,867 | 1,83E-14 | -1,516 |
| 204367_at | SP2 | Sp2 transcription factor | transcription regulator | 4,22E-13 | -1,761 | 4,22E-13 | -1,614 | 4,22E-13 | -2,058 |
| 204372_s_at | KHSRP | KH-type splicing regulatory protein | enzyme |  |  | 2,14E-08 | -1,654 |  |  |
| 204379_s_at | FGFR3 | fibroblast growth factor receptor 3 | kinase |  |  | 3,90E-09 | -1,649 |  |  |
| 204386_s_at | MRP63 | mitochondrial ribosomal protein 63 | other |  |  | 4,77E-10 | -1,630 |  |  |
| 204404_at | SLC12A2 | solute carrier family 12 (sodium/potassium/chloride transporters), member 2 | transporter |  |  | 3,31E-07 | -1,433 |  |  |
| 204417_at | GALC | galactosylceramidase | enzyme | 5,62E-11 | -1,551 | 5,62E-11 | -1,723 | 5,62E-11 | -1,452 |
| 204420_at | FOSL1 | FOS-like antigen 1 | transcription regulator |  |  |  |  | 6,03E-10 | 1,844 |
| 204433_s_at | SPATA2 | spermatogenesis associated 2 | other |  |  | 1,08E-07 | -2,440 |  |  |
| 204437_s_at | FOLR1 | folate receptor 1 (adult) | transporter | 1,61E-11 | -1,706 | 1,61E-11 | -2,366 | 1,61E-11 | -1,763 |
| 204444_at | KIF11 | kinesin family member 11 | other | 6,50E-13 | -1,472 | 6,50E-13 | -1,609 | 6,50E-13 | -1,393 |
| 204449_at | PDCL | phosducin-like | enzyme | 1,83E-14 | -1,919 | 1,83E-14 | -2,231 |  |  |
| 204450_x_at | APOA1 | apolipoprotein A-I | transporter | 1,21E-10 | -1,364 | 1,21E-10 | -1,730 | 1,21E-10 | -1,518 |
| 204459_at | CSTF2 (includes EG:1478) | cleavage stimulation factor, 3' pre-RNA, subunit 2, 64kDa | other |  |  | 3,17E-11 | -1,970 |  |  |
| 204480_s_at | C9ORF16 | chromosome 9 open reading frame 16 | other |  |  | 2,13E-10 | -1,750 | 2,13E-10 | -1,762 |
| 204485_s_at | TOM1L1 | target of myb1 (chicken)-like 1 | other |  |  | 3,05E-08 | -1,612 |  |  |
| 204504_s_at | HIRIP3 | HIRA interacting protein 3 | other |  |  | 8,85E-09 | -1,909 | 8,85E-09 | -1,566 |
| 204517_at | PPIC | peptidylprolyl isomerase C (cyclophilin C) | enzyme |  |  | 3,91E-08 | -1,570 |  |  |
| 204519_s_at | PLLP | plasma membrane proteolipid (plasmolipin) | transporter |  |  | 5,80E-09 | -1,843 |  |  |
| 204521_at | C12ORF24 | chromosome 12 open reading frame 24 | other |  |  | 1,02E-09 | -1,870 |  |  |
| 204523_at | ZNF140 | zinc finger protein 140 | transcription regulator | 3,40E-09 | -1,968 | 3,40E-09 | -1,553 |  |  |
| 204559_s_at | LSM7 | LSM7 homolog, U6 small nuclear RNA associated (S. cerevisiae) | other |  |  | 4,07E-09 | -1,657 |  |  |
| 204565_at | THEM2 | thioesterase superfamily member 2 | enzyme |  |  | 3,19E-07 | -1,538 |  |  |
| 204571_x_at | PIN4 | protein (peptidylprolyl cis/trans isomerase) NIMA-interacting, 4 (parvulin) | enzyme |  |  | 1,83E-11 | -1,975 |  |  |
| 204573_at | CROT | carnitine O-octanoyltransferase | enzyme | 7,66E-08 | -1,527 |  |  |  |  |
| 204587_at | SLC25A14 | solute carrier family 25 (mitochondrial carrier, brain), member 14 | transporter |  |  | 5,89E-08 | -1,847 |  |  |
| 204599_s_at | MRPL28 | mitochondrial ribosomal protein L28 | other | 1,51E-10 | -1,467 | 1,51E-10 | -1,880 |  |  |
| 204607_at | HMGCS2 | 3-hydroxy-3-methylglutaryl-Coenzyme A synthase 2 (mitochondrial) | enzyme | 1,83E-14 | 3,707 |  |  | 1,83E-14 | 2,264 |
| 204608_at | ASL | argininosuccinate lyase | enzyme | 1,08E-11 | -1,380 | 1,08E-11 | -1,778 | 1,08E-11 | -1,641 |
| 204616_at | UCHL3 | ubiquitin carboxyl-terminal esterase L3 (ubiquitin thiolesterase) | peptidase |  |  | 8,42E-08 | -1,479 |  |  |
| 204617_s_at | ACD | adrenocortical dysplasia homolog (mouse) | other |  |  | 1,42E-08 | -1,784 |  |  |
| 204624_at | ATP7B | ATPase, Cu++ transporting, beta polypeptide | transporter | 1,83E-14 | -1,833 | 1,83E-14 | -2,330 | 1,83E-14 | -2,164 |
| 204632_at | RPS6KA4 | ribosomal protein S6 kinase, 90kDa, polypeptide 4 | kinase |  |  | 8,03E-09 | -2,325 |  |  |
| 204641_at | NEK2 | NIMA (never in mitosis gene a)-related kinase 2 | kinase |  |  | 5,24E-09 | -1,664 |  |  |
| 204667_at | FOXA1 | forkhead box A1 | transcription regulator | 2,21E-09 | -1,617 | 2,21E-09 | -1,873 | 2,21E-09 | -1,741 |
| 204688_at | SGCE | sarcoglycan, epsilon | other |  |  | 5,85E-08 | -1,638 |  |  |
| 204689_at | HHEX | hematopoietically expressed homeobox | transcription regulator |  |  | 9,08E-07 | -1,487 |  |  |
| 204702_s_at | NFE2L3 | nuclear factor (erythroid-derived 2)-like 3 | transcription regulator |  |  | 4,64E-12 | -1,553 |  |  |
| 204709_s_at | KIF23 | kinesin family member 23 | other |  |  | 5,01E-08 | -1,619 |  |  |
| 204733_at | KLK6 | kallikrein-related peptidase 6 | peptidase | 2,25E-13 | -1,423 | 2,25E-13 | -2,103 | 2,25E-13 | -1,650 |
| 204739_at | CENPC1 | centromere protein C 1 | other | 1,84E-08 | -1,534 | 1,84E-08 | -1,666 | 1,84E-08 | -1,416 |
| 204745_x_at | MT1G | metallothionein 1G | other | 1,83E-14 | 5,627 |  |  | 1,83E-14 | 3,651 |
| 204752_x_at | PARP2 | poly (ADP-ribose) polymerase 2 | enzyme | 1,22E-09 | -1,384 | 1,22E-09 | -1,880 |  |  |
| 204767_s_at | FEN1 | flap structure-specific endonuclease 1 | enzyme | 3,29E-13 | -1,653 | 3,29E-13 | -1,795 | 3,29E-13 | -1,498 |
| 204768_s_at | FEN1 | flap structure-specific endonuclease 1 | enzyme |  |  | 2,33E-08 | -1,591 |  |  |
| 204779_s_at | HOXB7 | homeobox B7 | transcription regulator |  |  | 9,63E-12 | -1,787 |  |  |
| 204805_s_at | H1FX | H1 histone family, member X | other |  |  | 7,95E-12 | -1,915 | 7,95E-12 | -1,509 |
| 204806_x_at | HLA-F | major histocompatibility complex, class I, F | transmembrane receptor | 1,26E-13 | -1,802 | 1,26E-13 | -2,658 | 1,26E-13 | -1,736 |
| 204812_at | ZW10 | ZW10, kinetochore associated, homolog (Drosophila) | other |  |  | 5,61E-10 | -1,672 | 5,61E-10 | -1,436 |
| 204822_at | TTK | TTK protein kinase | kinase |  |  | 1,46E-08 | -1,572 |  |  |
| 204823_at | NAV3 | neuron navigator 3 | other |  |  |  |  | 1,80E-12 | 2,500 |
| 204826_at | CCNF | cyclin F | other |  |  | 2,14E-10 | -2,150 |  |  |
| 204832_s_at | BMPR1A | bone morphogenetic protein receptor, type IA | kinase | 2,01E-12 | -1,594 | 2,01E-12 | -2,009 | 2,01E-12 | -1,474 |
| 204839_at | POP5 | processing of precursor 5, ribonuclease P/MRP subunit (S. cerevisiae) | enzyme |  |  | 2,12E-10 | -1,620 |  |  |
| 204849_at | TCFL5 | transcription factor-like 5 (basic helix-loop-helix) | transcription regulator |  |  | 1,83E-14 | -2,221 | 1,83E-14 | -1,761 |
| 204863_s_at | IL6ST | interleukin 6 signal transducer (gp130, oncostatin M receptor) | transmembrane receptor | 1,83E-14 | 2,656 | 1,83E-14 | 2,830 | 1,83E-14 | 4,150 |
| 204866_at | PHF16 | PHD finger protein 16 | other |  |  | 9,87E-09 | -1,746 |  |  |
| 204900_x_at | SAP30 | Sin3A-associated protein, 30kDa | transcription regulator | 1,31E-07 | -1,357 |  |  |  |  |
| 204933_s_at | TNFRSF11B | tumor necrosis factor receptor superfamily, member 11b | transmembrane receptor |  |  |  |  | 1,83E-14 | 1,702 |
| 204957_at | ORC5L | origin recognition complex, subunit 5-like (yeast) | other |  |  | 1,96E-10 | -1,699 |  |  |
| 204975_at | EMP2 | epithelial membrane protein 2 | other | 4,87E-14 | -1,684 | 4,87E-14 | -1,770 | 4,87E-14 | -1,608 |
| 204988_at | FGB | fibrinogen beta chain | other | 1,47E-10 | -2,295 | 1,47E-10 | -1,895 | 1,47E-10 | -3,477 |
| 205024_s_at | RAD51 | RAD51 homolog (RecA homolog, E. coli) (S. cerevisiae) | enzyme | 1,09E-10 | -1,654 | 1,09E-10 | -1,950 | 1,09E-10 | -1,445 |
| 205032_at | ITGA2 | integrin, alpha 2 (CD49B, alpha 2 subunit of VLA-2 receptor) | other |  |  |  |  | 4,87E-14 | 1,521 |
| 205042_at | GNE | glucosamine (UDP-N-acetyl)-2-epimerase/N-acetylmannosamine kinase | kinase |  |  | 3,62E-08 | -1,925 |  |  |
| 205053_at | PRIM1 | primase, DNA, polypeptide 1 (49kDa) | enzyme |  |  | 3,62E-09 | -1,641 |  |  |
| 205055_at | ITGAE | integrin, alpha E (antigen CD103, human mucosal lymphocyte antigen 1; alpha polypeptide) | other |  |  | 2,21E-10 | -1,729 |  |  |
| 205078_at | PIGF | phosphatidylinositol glycan anchor biosynthesis, class F | enzyme |  |  | 4,66E-08 | -1,681 |  |  |
| 205084_at | BCAP29 | B-cell receptor-associated protein 29 | transporter | 1,83E-14 | -1,832 | 1,83E-14 | -2,147 | 1,83E-14 | -1,593 |
| 205087_at | RWDD3 | RWD domain containing 3 | other | 4,97E-10 | -1,510 | 4,97E-10 | -1,762 |  |  |
| 205108_s_at | APOB | apolipoprotein B (including Ag(x) antigen) | transporter |  |  | 5,90E-09 | -1,640 | 5,90E-09 | -1,665 |
| 205129_at | NPM3 | nucleophosmin/nucleoplasmin, 3 | other |  |  | 5,73E-09 | -1,739 |  |  |
| 205158_at | RNASE4 | ribonuclease, RNase A family, 4 | enzyme | 6,53E-08 | -1,713 |  |  |  |  |
| 205167_s_at | CDC25C | cell division cycle 25 homolog C (S. pombe) | phosphatase | 1,93E-08 | -1,518 | 1,93E-08 | -1,622 |  |  |
| 205176_s_at | ITGB3BP | integrin beta 3 binding protein (beta3-endonexin) | other |  |  | 1,50E-09 | -1,619 |  |  |
| 205187_at | SMAD5 | SMAD family member 5 | transcription regulator |  |  | 8,80E-08 | 1,681 |  |  |
| 205194_at | PSPH | phosphoserine phosphatase | phosphatase | 1,08E-08 | -1,466 |  |  | 1,08E-08 | -1,462 |
| 205216_s_at | APOH | apolipoprotein H (beta-2-glycoprotein I) | transporter | 1,83E-14 | -1,777 | 1,83E-14 | -1,829 | 1,83E-14 | -1,767 |
| 205229_s_at | COCH | coagulation factor C homolog, cochlin (Limulus polyphemus) | other | 1,81E-11 | -1,426 | 1,81E-11 | -1,649 |  |  |
| 205235_s_at | KIF20B | kinesin family member 20B | enzyme |  |  | 9,15E-10 | -1,650 |  |  |
| 205260_s_at | ACYP1 | acylphosphatase 1, erythrocyte (common) type | enzyme | 9,78E-10 | -1,533 | 9,78E-10 | -1,824 |  |  |
| 205289_at | BMP2 | bone morphogenetic protein 2 | growth factor | 1,14E-09 | -1,704 | 1,14E-09 | -1,607 | 1,14E-09 | -1,555 |
| 205290_s_at | BMP2 | bone morphogenetic protein 2 | growth factor | 3,62E-09 | -1,621 | 3,62E-09 | -1,741 | 3,62E-09 | -1,440 |
| 205296_at | RBL1 | retinoblastoma-like 1 (p107) | other | 1,58E-06 | -1,431 |  |  |  |  |
| 205301_s_at | OGG1 | 8-oxoguanine DNA glycosylase | enzyme |  |  | 3,25E-10 | -2,533 | 3,25E-10 | -2,240 |
| 205315_s_at | SNTB2 | syntrophin, beta 2 (dystrophin-associated protein A1, 59kDa, basic component 2) | other |  |  | 3,43E-09 | -1,875 |  |  |
| 205361_s_at | PFDN4 | prefoldin subunit 4 | other |  |  | 7,63E-08 | -1,583 |  |  |
| 205370_x_at | DBT | dihydrolipoamide branched chain transacylase E2 | enzyme |  |  |  |  | 2,01E-08 | -1,704 |
| 205393_s_at | CHEK1 | CHK1 checkpoint homolog (S. pombe) | kinase |  |  | 1,49E-09 | -1,638 |  |  |
| 205394_at | CHEK1 | CHK1 checkpoint homolog (S. pombe) | kinase |  |  | 1,28E-08 | -1,529 |  |  |
| 205406_s_at | SPA17 | sperm autoantigenic protein 17 | other |  |  | 4,00E-08 | -1,688 |  |  |
| 205417_s_at | DAG1 | dystroglycan 1 (dystrophin-associated glycoprotein 1) | transmembrane receptor |  |  | 7,61E-12 | -1,958 |  |  |
| 205429_s_at | MPP6 | membrane protein, palmitoylated 6 (MAGUK p55 subfamily member 6) | kinase |  |  | 1,46E-12 | -1,658 |  |  |
| 205452_at | PIGB | phosphatidylinositol glycan anchor biosynthesis, class B | enzyme |  |  | 2,50E-08 | -1,665 | 2,50E-08 | -1,461 |
| 205463_s_at | PDGFA | platelet-derived growth factor alpha polypeptide | growth factor | 1,25E-09 | -1,792 | 1,25E-09 | -2,034 | 1,25E-09 | -1,512 |
| 205480_s_at | UGP2 | UDP-glucose pyrophosphorylase 2 | enzyme |  |  | 2,03E-10 | -1,562 |  |  |
| 205512_s_at | AIFM1 | apoptosis-inducing factor, mitochondrion-associated, 1 | enzyme |  |  | 2,44E-10 | -1,816 |  |  |
| 205541_s_at | GSPT2 | G1 to S phase transition 2 | translation regulator |  |  | 1,44E-09 | -1,692 |  |  |
| 205542_at | STEAP1 | six transmembrane epithelial antigen of the prostate 1 | transporter |  |  | 2,02E-09 | -1,547 |  |  |
| 205545_x_at | DNAJC8 | DnaJ (Hsp40) homolog, subfamily C, member 8 | other |  |  | 6,37E-09 | -1,772 |  |  |
| 205547_s_at | TAGLN | transgelin | other |  |  | 3,64E-13 | -1,725 |  |  |
| 205548_s_at | BTG3 | BTG family, member 3 | other |  |  | 1,62E-08 | -1,602 |  |  |
| 205552_s_at | OAS1 | 2',5'-oligoadenylate synthetase 1, 40/46kDa | enzyme |  |  | 1,15E-07 | -1,683 |  |  |
| 205555_s_at | MSX2 | msh homeobox 2 | transcription regulator | 1,25E-08 | -1,703 | 1,25E-08 | -1,863 | 1,25E-08 | -2,092 |
| 205632_s_at | PIP5K1B | phosphatidylinositol-4-phosphate 5-kinase, type I, beta | kinase | 1,79E-07 | -1,457 |  |  |  |  |
| 205633_s_at | ALAS1 | aminolevulinate, delta-, synthase 1 | enzyme |  |  | 7,31E-08 | -1,722 |  |  |
| 205644_s_at | SNRPG | small nuclear ribonucleoprotein polypeptide G | other |  |  | 1,57E-07 | -1,419 |  |  |
| 205650_s_at | FGA | fibrinogen alpha chain | other | 3,22E-10 | -3,426 |  |  | 3,22E-10 | -5,578 |
| 205664_at | KIN | KIN, antigenic determinant of recA protein homolog (mouse) | other |  |  | 1,35E-09 | -1,762 |  |  |
| 205690_s_at | BUD31 (includes EG:8896) | BUD31 homolog (S. cerevisiae) | transcription regulator | 2,18E-09 | -1,424 | 2,18E-09 | -1,606 |  |  |
| 205710_at | LRP2 | low density lipoprotein-related protein 2 | transporter | 1,43E-12 | -1,689 | 1,43E-12 | -1,623 | 1,43E-12 | -1,760 |
| 205711_x_at | ATP5C1 | ATP synthase, H+ transporting, mitochondrial F1 complex, gamma polypeptide 1 | transporter |  |  | 2,59E-08 | -1,479 |  |  |
| 205740_s_at | RBM42 | RNA binding motif protein 42 | other |  |  | 1,50E-08 | -1,974 |  |  |
| 205749_at | CYP1A1 | cytochrome P450, family 1, subfamily A, polypeptide 1 | enzyme | 1,83E-14 | 9,691 | 1,83E-14 | 5,329 | 1,83E-14 | 11,092 |
| 205774_at | F12 | coagulation factor XII (Hageman factor) | peptidase |  |  | 1,56E-08 | -2,159 |  |  |
| 205788_s_at | ZC3H11A | zinc finger CCCH-type containing 11A | other |  |  | 1,09E-08 | -1,495 |  |  |
| 205805_s_at | ROR1 | receptor tyrosine kinase-like orphan receptor 1 | kinase | 3,50E-07 | -1,582 |  |  |  |  |
| 205809_s_at | WASL (includes EG:8976) | Wiskott-Aldrich syndrome-like | other |  |  |  |  | 9,94E-10 | 1,627 |
| 205848_at | GAS2 | growth arrest-specific 2 | other | 6,17E-09 | -3,402 |  |  |  |  |
| 205849_s_at | UQCRB | ubiquinol-cytochrome c reductase binding protein | enzyme |  |  | 6,12E-10 | -1,619 |  |  |
| 205865_at | ARID3A | AT rich interactive domain 3A (BRIGHT-like) | transcription regulator |  |  | 3,60E-08 | -1,793 |  |  |
| 205882_x_at | ADD3 | adducin 3 (gamma) | other |  |  | 1,19E-08 | -1,568 |  |  |
| 205890_s_at | GABBR1 | gamma-aminobutyric acid (GABA) B receptor, 1 | G-protein coupled receptor | 7,26E-12 | -2,153 | 7,26E-12 | -1,607 | 7,26E-12 | -1,438 |
| 205892_s_at | FABP1 | fatty acid binding protein 1, liver | transporter | 1,83E-14 | 1,441 | 1,83E-14 | -2,118 |  |  |
| 205905_s_at | HLA-B | major histocompatibility complex, class I, B | transmembrane receptor |  |  | 1,64E-07 | -1,662 |  |  |
| 205961_s_at | PSIP1 | PC4 and SFRS1 interacting protein 1 | other |  |  | 4,34E-11 | -1,786 |  |  |
| 205963_s_at | DNAJA3 | DnaJ (Hsp40) homolog, subfamily A, member 3 | other |  |  | 2,03E-08 | -1,796 |  |  |
| 205977_s_at | EPHA1 | EPH receptor A1 | kinase |  |  | 6,13E-09 | -2,289 |  |  |
| 206028_s_at | MERTK | c-mer proto-oncogene tyrosine kinase | kinase |  |  | 2,68E-09 | -1,654 |  |  |
| 206042_x_at | SNRPN | small nuclear ribonucleoprotein polypeptide N | other | 5,18E-13 | -1,412 | 5,18E-13 | -1,897 | 5,18E-13 | -1,586 |
| 206052_s_at | SLBP | stem-loop binding protein | other | 2,53E-12 | -1,381 | 2,53E-12 | -1,674 | 2,53E-12 | -1,453 |
| 206055_s_at | SNRPA1 | small nuclear ribonucleoprotein polypeptide A' | other |  |  | 1,58E-10 | -1,505 |  |  |
| 206074_s_at | HMGA1 | high mobility group AT-hook 1 | transcription regulator |  |  | 5,15E-12 | -2,051 |  |  |
| 206138_s_at | PI4KB | phosphatidylinositol 4-kinase, catalytic, beta | kinase |  |  | 1,34E-09 | -2,018 |  |  |
| 206238_s_at | YAF2 | YY1 associated factor 2 | transcription regulator |  |  | 2,44E-10 | -2,198 |  |  |
| 206245_s_at | IVNS1ABP | influenza virus NS1A binding protein | other |  |  | 3,95E-09 | -1,608 |  |  |
| 206256_at | CPN1 | carboxypeptidase N, polypeptide 1 | peptidase |  |  | 1,04E-06 | -1,866 |  |  |
| 206284_x_at | CLTB | clathrin, light chain (Lcb) | other |  |  | 9,27E-10 | -1,956 |  |  |
| 206289_at | HOXA4 | homeobox A4 | transcription regulator | 5,78E-07 | -1,777 |  |  |  |  |
| 206385_s_at | ANK3 | ankyrin 3, node of Ranvier (ankyrin G) | other |  |  | 8,65E-09 | -1,623 |  |  |
| 206429_at | F2RL1 | coagulation factor II (thrombin) receptor-like 1 | G-protein coupled receptor |  |  | 1,10E-09 | -1,577 |  |  |
| 206445_s_at | PRMT1 | protein arginine methyltransferase 1 | enzyme | 1,83E-14 | -1,586 | 1,83E-14 | -2,471 | 1,83E-14 | -1,605 |
| 206461_x_at | MT1H | metallothionein 1H | other | 1,83E-14 | 4,919 |  |  | 1,83E-14 | 3,199 |
| 206463_s_at | DHRS2 (includes EG:10202) | dehydrogenase/reductase (SDR family) member 2 | enzyme | 2,62E-09 | -1,851 | 2,62E-09 | -2,321 | 2,62E-09 | -2,239 |
| 206468_s_at | KIAA0859 | KIAA0859 | enzyme |  |  | 1,26E-09 | -2,416 |  |  |
| 206491_s_at | NAPA | N-ethylmaleimide-sensitive factor attachment protein, alpha | transporter |  |  | 1,15E-09 | -2,087 |  |  |
| 206565_x_at | SMA4 | glucuronidase, beta pseudogene | other | 1,57E-10 | -1,836 | 1,57E-10 | -2,041 | 1,57E-10 | -1,505 |
| 206593_s_at | MED22 | mediator complex subunit 22 | other |  |  | 1,66E-08 | -1,874 |  |  |
| 206632_s_at | APOBEC3B | apolipoprotein B mRNA editing enzyme, catalytic polypeptide-like 3B | enzyme | 1,32E-10 | -1,453 | 1,32E-10 | -1,591 | 1,32E-10 | -1,365 |
| 206664_at | SI | sucrase-isomaltase (alpha-glucosidase) | enzyme | 3,44E-14 | -1,886 | 3,44E-14 | -1,641 | 3,44E-14 | -3,944 |
| 206665_s_at | BCL2L1 | BCL2-like 1 | other |  |  | 3,84E-09 | -1,928 |  |  |
| 206688_s_at | CPSF4 | cleavage and polyadenylation specific factor 4, 30kDa | other |  |  | 2,92E-09 | -1,821 |  |  |
| 206715_at | TFEC | transcription factor EC | transcription regulator | 1,83E-14 | -1,948 | 1,83E-14 | -2,211 | 1,83E-14 | -3,041 |
| 206790_s_at | NDUFB1 | NADH dehydrogenase (ubiquinone) 1 beta subcomplex, 1, 7kDa | enzyme |  |  | 1,55E-12 | -1,704 | 1,55E-12 | -1,439 |
| 206805_at | SEMA3A | sema domain, immunoglobulin domain (Ig), short basic domain, secreted, (semaphorin) 3A | other | 6,76E-10 | -2,033 | 6,76E-10 | -1,977 | 6,76E-10 | -1,631 |
| 206845_s_at | RNF40 | ring finger protein 40 | enzyme |  |  | 3,01E-09 | -2,304 |  |  |
| 206846_s_at | HDAC6 | histone deacetylase 6 | transcription regulator |  |  | 9,03E-08 | -1,930 |  |  |
| 206855_s_at | HYAL2 | hyaluronoglucosaminidase 2 | enzyme |  |  | 1,41E-11 | -2,214 |  |  |
| 206858_s_at | HOXC6 | homeobox C6 | transcription regulator |  |  | 3,40E-11 | -1,686 | 3,40E-11 | -1,656 |
| 206861_s_at | CGGBP1 | CGG triplet repeat binding protein 1 | other |  |  | 7,35E-08 | -1,551 |  |  |
| 206918_s_at | CPNE1 | copine I | transporter |  |  | 3,27E-08 | -1,595 |  |  |
| 206945_at | LCT | lactase | enzyme |  |  |  |  | 3,44E-14 |  |
| 206958_s_at | UPF3A | UPF3 regulator of nonsense transcripts homolog A (yeast) | transporter | 6,26E-14 | -1,535 | 6,26E-14 | -1,779 | 6,26E-14 | -1,446 |
| 206989_s_at | SFRS2IP | splicing factor, arginine/serine-rich 2, interacting protein | other |  |  |  |  | 3,90E-08 | -1,360 |
| 207023_x_at | KRT10 | keratin 10 | other |  |  | 3,55E-09 | -1,678 |  |  |
| 207029_at | KITLG | KIT ligand | growth factor |  |  | 1,87E-10 | -1,727 | 1,87E-10 | -1,579 |
| 207040_s_at | ST13 | suppression of tumorigenicity 13 (colon carcinoma) (Hsp70 interacting protein) | other |  |  | 5,25E-08 | -1,485 |  |  |
| 207076_s_at | ASS1 | argininosuccinate synthetase 1 | enzyme |  |  | 9,71E-09 | -1,866 |  |  |
| 207081_s_at | PI4KA | phosphatidylinositol 4-kinase, catalytic, alpha | kinase |  |  | 3,51E-10 | -2,074 | 3,51E-10 | -1,480 |
| 207098_s_at | MFN1 | mitofusin 1 | enzyme |  |  | 5,35E-09 | -1,541 |  |  |
| 207157_s_at | GNG5 | guanine nucleotide binding protein (G protein), gamma 5 | enzyme |  |  | 8,05E-12 | -1,722 | 8,05E-12 | -1,417 |
| 207163_s_at | AKT1 | v-akt murine thymoma viral oncogene homolog 1 | kinase |  |  | 4,50E-13 | -2,351 |  |  |
| 207169_x_at | DDR1 | discoidin domain receptor tyrosine kinase 1 | kinase | 1,83E-14 | -1,525 | 1,83E-14 | -2,220 | 1,83E-14 | -1,567 |
| 207170_s_at | LETMD1 | LETM1 domain containing 1 | other |  |  | 3,04E-08 | -1,644 |  |  |
| 207181_s_at | CASP7 | caspase 7, apoptosis-related cysteine peptidase | peptidase |  |  | 3,73E-07 | -1,773 |  |  |
| 207300_s_at | F7 | coagulation factor VII (serum prothrombin conversion accelerator) | peptidase | 3,32E-10 | -1,676 | 3,32E-10 | -2,413 | 3,32E-10 | -1,914 |
| 207335_x_at | ATP5I | ATP synthase, H+ transporting, mitochondrial F0 complex, subunit E | transporter |  |  | 1,61E-07 | -1,601 |  |  |
| 207405_s_at | RAD17 | RAD17 homolog (S. pombe) | other |  |  | 4,79E-11 | -1,731 |  |  |
| 207431_s_at | DEGS1 | degenerative spermatocyte homolog 1, lipid desaturase (Drosophila) | enzyme |  |  | 2,46E-07 | -1,666 |  |  |
| 207438_s_at | SNUPN | snurportin 1 | other |  |  | 2,35E-08 | -1,643 | 2,35E-08 | -1,418 |
| 207467_x_at | CAST | calpastatin | peptidase | 7,47E-12 | -1,409 | 7,47E-12 | -1,447 | 7,47E-12 | -1,657 |
| 207480_s_at | MEIS2 | Meis homeobox 2 | transcription regulator |  |  | 1,61E-08 | -1,651 |  |  |
| 207545_s_at | NUMB | numb homolog (Drosophila) | other | 1,83E-14 | -1,962 | 1,83E-14 | -2,357 | 1,83E-14 | -1,942 |
| 207556_s_at | DGKZ | diacylglycerol kinase, zeta 104kDa | kinase |  |  | 4,54E-09 | -1,927 |  |  |
| 207558_s_at | PITX2 | paired-like homeodomain 2 | transcription regulator |  |  | 6,02E-08 | -1,497 |  |  |
| 207614_s_at | CUL1 | cullin 1 | enzyme |  |  | 8,65E-11 | -1,808 |  |  |
| 207622_s_at | ABCF2 | ATP-binding cassette, sub-family F (GCN20), member 2 | transporter |  |  | 1,98E-09 | -1,852 |  |  |
| 207628_s_at | WBSCR22 | Williams Beuren syndrome chromosome region 22 | enzyme | 1,98E-13 | -1,409 | 1,98E-13 | -1,745 |  |  |
| 207714_s_at | SERPINH1 | serpin peptidase inhibitor, clade H (heat shock protein 47), member 1, (collagen binding protein 1) | other |  |  | 3,22E-10 | -1,769 |  |  |
| 207717_s_at | PKP2 | plakophilin 2 | other | 1,83E-14 | -1,460 | 1,83E-14 | -1,599 | 1,83E-14 | -1,577 |
| 207727_s_at | MUTYH | mutY homolog (E. coli) | enzyme |  |  | 1,52E-08 | -1,762 |  |  |
| 207761_s_at | METTL7A | methyltransferase like 7A | other |  |  | 2,89E-08 | -2,061 |  |  |
| 207782_s_at | PSEN1 | presenilin 1 | peptidase |  |  | 2,02E-08 | -1,729 |  |  |
| 207791_s_at | RAB1A | RAB1A, member RAS oncogene family | enzyme |  |  | 2,71E-08 | -1,445 |  |  |
| 207801_s_at | RNF10 | ring finger protein 10 | other |  |  | 6,35E-09 | -1,675 |  |  |
| 207805_s_at | PSMD9 | proteasome (prosome, macropain) 26S subunit, non-ATPase, 9 | transcription regulator |  |  | 4,67E-09 | -1,607 |  |  |
| 207809_s_at | ATP6AP1 | ATPase, H+ transporting, lysosomal accessory protein 1 | transporter |  |  | 6,00E-10 | -1,753 |  |  |
| 207843_x_at | CYB5A | cytochrome b5 type A (microsomal) | enzyme |  |  | 2,90E-09 | -1,634 |  |  |
| 207922_s_at | MAEA | macrophage erythroblast attacher | other |  |  | 2,39E-12 | -1,923 |  |  |
| 207941_s_at | RBM39 | RNA binding motif protein 39 | transcription regulator | 3,14E-10 | -1,383 | 3,14E-10 | -1,455 |  |  |
| 207945_s_at | CSNK1D | casein kinase 1, delta | kinase |  |  | 6,34E-08 | -1,768 |  |  |
| 207974_s_at | SKP1 | S-phase kinase-associated protein 1 | transcription regulator |  |  | 1,93E-07 | -1,414 |  |  |
| 208002_s_at | ACOT7 | acyl-CoA thioesterase 7 | enzyme | 6,26E-14 | -1,504 | 6,26E-14 | -1,981 | 6,26E-14 | -1,457 |
| 208003_s_at | NFAT5 | nuclear factor of activated T-cells 5, tonicity-responsive | transcription regulator | 1,83E-14 | 2,404 | 1,83E-14 | 3,048 | 1,83E-14 | 3,891 |
| 208024_s_at | DGCR6 | DiGeorge syndrome critical region gene 6 | other | 2,41E-09 | -1,618 | 2,41E-09 | -2,056 |  |  |
| 208029_s_at | LAPTM4B | lysosomal protein transmembrane 4 beta | other |  |  | 1,56E-11 | -1,658 |  |  |
| 208047_s_at | NAB1 | NGFI-A binding protein 1 (EGR1 binding protein 1) | transcription regulator | 6,28E-08 | -1,795 |  |  |  |  |
| 208073_x_at | TTC3 | tetratricopeptide repeat domain 3 | other | 8,88E-11 | -1,407 | 8,88E-11 | -1,600 |  |  |
| 208079_s_at | AURKA | aurora kinase A | kinase |  |  | 2,28E-10 | -1,634 | 2,28E-10 | -1,294 |
| 208091_s_at | ECOP | EGFR-coamplified and overexpressed protein | other |  |  | 2,10E-08 | -1,789 |  |  |
| 208093_s_at | NDEL1 | nudE nuclear distribution gene E homolog (A. nidulans)-like 1 | other |  |  | 3,57E-09 | -1,847 |  |  |
| 208107_s_at | LOC81691 | exonuclease NEF-sp | enzyme |  |  |  |  | 1,04E-06 | -1,676 |
| 208146_s_at | CPVL | carboxypeptidase, vitellogenic-like | peptidase |  |  | 4,78E-08 | -1,577 |  |  |
| 208149_x_at | DDX11 | DEAD/H (Asp-Glu-Ala-Asp/His) box polypeptide 11 (CHL1-like helicase homolog, S. cerevisiae) | enzyme |  |  | 5,22E-09 | -1,892 |  |  |
| 208161_s_at | ABCC3 | ATP-binding cassette, sub-family C (CFTR/MRP), member 3 | transporter |  |  | 1,04E-10 | -1,830 |  |  |
| 208190_s_at | LSR | lipolysis stimulated lipoprotein receptor | transcription regulator | 1,83E-14 | -1,750 | 1,83E-14 | -2,417 | 1,83E-14 | -1,662 |
| 208209_s_at | C4BPB | complement component 4 binding protein, beta | other | 7,91E-13 | -1,695 | 7,91E-13 | -1,678 | 7,91E-13 | -1,409 |
| 208216_at | DLX4 | distal-less homeobox 4 | transcription regulator |  |  | 1,74E-08 | -1,737 | 1,74E-08 | -1,594 |
| 208228_s_at | FGFR2 | fibroblast growth factor receptor 2 | kinase | 9,97E-10 | -1,808 |  |  | 9,97E-10 | -3,183 |
| 208249_s_at | TGDS | TDP-glucose 4,6-dehydratase | enzyme |  |  | 4,76E-10 | -1,758 |  |  |
| 208264_s_at | EIF3J | eukaryotic translation initiation factor 3, subunit J | translation regulator |  |  | 2,04E-08 | -1,636 |  |  |
| 208270_s_at | RNPEP | arginyl aminopeptidase (aminopeptidase B) | peptidase |  |  | 4,98E-11 | -1,702 |  |  |
| 208289_s_at | EI24 | etoposide induced 2.4 mRNA | other |  |  | 1,19E-07 | -1,554 |  |  |
| 208290_s_at | EIF5 | eukaryotic translation initiation factor 5 | translation regulator |  |  | 6,76E-11 | -1,707 |  |  |
| 208308_s_at | GPI | glucose phosphate isomerase | enzyme |  |  | 2,35E-11 | -1,804 |  |  |
| 208319_s_at | RBM3 | RNA binding motif (RNP1, RRM) protein 3 | other |  |  | 1,05E-12 | -1,827 |  |  |
| 208336_s_at | GPSN2 | glycoprotein, synaptic 2 | other |  |  | 6,58E-08 | -1,597 |  |  |
| 208398_s_at | TBPL1 | TBP-like 1 | transcription regulator | 2,53E-09 | -1,331 | 2,53E-09 | -1,631 |  |  |
| 208424_s_at | CIAPIN1 | cytokine induced apoptosis inhibitor 1 | other |  |  | 5,10E-11 | -1,684 |  |  |
| 208450_at | LGALS2 | lectin, galactoside-binding, soluble, 2 | other |  |  | 1,39E-13 | -1,975 | 1,39E-13 | -1,514 |
| 208453_s_at | XPNPEP1 | X-prolyl aminopeptidase (aminopeptidase P) 1, soluble | peptidase |  |  | 8,32E-10 | -1,770 |  |  |
| 208540_x_at | null |  | other |  |  | 1,83E-14 | -2,387 | 1,83E-14 | -1,716 |
| 208561_at | ABCC9 | ATP-binding cassette, sub-family C (CFTR/MRP), member 9 | ion channel | 2,25E-13 | -2,225 | 2,25E-13 | -2,073 | 2,25E-13 | -2,470 |
| 208581_x_at | MT1X | metallothionein 1X | other | 1,83E-14 | 5,359 |  |  | 1,83E-14 | 3,434 |
| 208596_s_at | UGT1A7 | UDP glucuronosyltransferase 1 family, polypeptide A7 | enzyme |  |  |  |  | 3,44E-14 | 3,031 |
| 208598_s_at | HUWE1 | HECT, UBA and WWE domain containing 1 | transcription regulator |  |  | 2,18E-08 | -1,583 |  |  |
| 208619_at | DDB1 | damage-specific DNA binding protein 1, 127kDa | other |  |  | 1,34E-08 | -1,647 |  |  |
| 208620_at | PCBP1 (includes EG:5093) | poly(rC) binding protein 1 | translation regulator |  |  | 7,46E-08 | -1,586 |  |  |
| 208622_s_at | EZR | ezrin | other | 1,44E-09 | -1,489 | 1,44E-09 | -1,658 | 1,44E-09 | -1,351 |
| 208623_s_at | EZR | ezrin | other | 3,44E-14 | -1,538 | 3,44E-14 | -2,013 |  |  |
| 208625_s_at | EIF4G1 | eukaryotic translation initiation factor 4 gamma, 1 | translation regulator |  |  | 2,08E-10 | -1,783 |  |  |
| 208631_s_at | HADHA | hydroxyacyl-Coenzyme A dehydrogenase/3-ketoacyl-Coenzyme A thiolase/enoyl-Coenzyme A hydratase (trifunctional protein), alpha subunit | enzyme |  |  | 1,40E-08 | -1,624 |  |  |
| 208634_s_at | MACF1 | microtubule-actin crosslinking factor 1 | other |  |  | 1,23E-08 | -1,633 |  |  |
| 208636_at | ACTN1 | actinin, alpha 1 | other | 5,90E-11 | -1,475 | 5,90E-11 | -1,701 | 5,90E-11 | -1,395 |
| 208639_x_at | PDIA6 | protein disulfide isomerase family A, member 6 | enzyme |  |  | 9,30E-08 | -1,480 |  |  |
| 208640_at | RAC1 | ras-related C3 botulinum toxin substrate 1 (rho family, small GTP binding protein Rac1) | enzyme |  |  | 1,10E-08 | -1,611 |  |  |
| 208643_s_at | XRCC5 | X-ray repair complementing defective repair in Chinese hamster cells 5 (double-strand-break rejoining) | enzyme |  |  | 6,26E-14 | -1,671 |  |  |
| 208653_s_at | CD164 | CD164 molecule, sialomucin | other |  |  | 8,11E-08 | -1,445 |  |  |
| 208654_s_at | CD164 | CD164 molecule, sialomucin | other | 1,28E-08 | -1,369 | 1,28E-08 | -1,403 |  |  |
| 208656_s_at | CCNI | cyclin I | other |  |  | 1,40E-07 | -1,546 |  |  |
| 208657_s_at | SEPT9 | septin 9 | enzyme | 1,26E-13 | -1,886 | 1,26E-13 | -3,409 | 1,26E-13 | -1,784 |
| 208658_at | PDIA4 | protein disulfide isomerase family A, member 4 | enzyme | 1,23E-11 | -1,723 | 1,23E-11 | -1,900 |  |  |
| 208660_at | CS | citrate synthase | enzyme |  |  | 1,00E-08 | -1,576 |  |  |
| 208666_s_at | ST13 | suppression of tumorigenicity 13 (colon carcinoma) (Hsp70 interacting protein) | other | 4,87E-14 | -1,479 | 4,87E-14 | -1,908 | 4,87E-14 | -1,437 |
| 208667_s_at | ST13 | suppression of tumorigenicity 13 (colon carcinoma) (Hsp70 interacting protein) | other |  |  | 1,96E-08 | -1,544 |  |  |
| 208669_s_at | EID1 | EP300 interacting inhibitor of differentiation 1 | transcription regulator | 5,31E-11 | -1,480 | 5,31E-11 | -1,891 | 5,31E-11 | -1,544 |
| 208671_at | SERINC1 | serine incorporator 1 | transporter |  |  | 2,00E-08 | -1,503 |  |  |
| 208675_s_at | DDOST | dolichyl-diphosphooligosaccharide-protein glycosyltransferase | enzyme |  |  | 2,09E-08 | -1,602 |  |  |
| 208682_s_at | MAGED2 | melanoma antigen family D, 2 | other | 1,83E-14 | -1,776 | 1,83E-14 | -2,339 | 1,83E-14 | -1,746 |
| 208683_at | CAPN2 | calpain 2, (m/II) large subunit | peptidase |  |  | 3,17E-08 | -1,447 |  |  |
| 208690_s_at | PDLIM1 | PDZ and LIM domain 1 | transcription regulator | 6,32E-13 | -1,365 | 6,32E-13 | -1,626 | 6,32E-13 | -1,424 |
| 208694_at | PRKDC | protein kinase, DNA-activated, catalytic polypeptide | kinase | 7,71E-11 | -1,415 | 7,71E-11 | -1,551 |  |  |
| 208699_x_at | TKT | transketolase | enzyme |  |  | 9,59E-12 | -1,827 |  |  |
| 208700_s_at | TKT | transketolase | enzyme |  |  | 2,73E-11 | -1,656 |  |  |
| 208710_s_at | AP3D1 | adaptor-related protein complex 3, delta 1 subunit | transporter | 1,83E-14 | 2,750 | 1,83E-14 | 3,634 | 1,83E-14 | 3,945 |
| 208713_at | HNRNPUL1 | heterogeneous nuclear ribonucleoprotein U-like 1 | other | 1,83E-14 | -1,377 | 1,83E-14 | -2,158 | 1,83E-14 | -1,712 |
| 208717_at | OXA1L | oxidase (cytochrome c) assembly 1-like | enzyme |  |  | 1,29E-07 | -1,662 |  |  |
| 208720_s_at | RBM39 | RNA binding motif protein 39 | transcription regulator | 8,30E-12 | -1,661 | 8,30E-12 | -1,456 |  |  |
| 208722_s_at | ANAPC5 | anaphase promoting complex subunit 5 | enzyme |  |  | 2,88E-08 | -1,566 |  |  |
| 208734_x_at | RAB2A | RAB2A, member RAS oncogene family | enzyme |  |  | 2,66E-07 | -1,567 |  |  |
| 208736_at | ARPC3 | actin related protein 2/3 complex, subunit 3, 21kDa | other | 1,83E-14 | -1,392 | 1,83E-14 | -1,840 | 1,83E-14 | -1,529 |
| 208749_x_at | FLOT1 | flotillin 1 | other |  |  | 2,29E-10 | -1,737 |  |  |
| 208750_s_at | ARF1 | ADP-ribosylation factor 1 | enzyme | 4,59E-13 | -1,533 | 4,59E-13 | -1,998 | 4,59E-13 | -1,501 |
| 208752_x_at | NAP1L1 | nucleosome assembly protein 1-like 1 | other |  |  | 2,69E-08 | -1,439 |  |  |
| 208753_s_at | NAP1L1 | nucleosome assembly protein 1-like 1 | other |  |  | 2,14E-08 | -1,518 |  |  |
| 208754_s_at | NAP1L1 | nucleosome assembly protein 1-like 1 | other | 5,62E-11 | -1,371 | 5,62E-11 | -1,572 | 5,62E-11 | -1,335 |
| 208756_at | EIF3I (includes EG:8668) | eukaryotic translation initiation factor 3, subunit I | translation regulator |  |  | 4,18E-09 | -1,592 |  |  |
| 208761_s_at | SUMO1 | SMT3 suppressor of mif two 3 homolog 1 (S. cerevisiae) | enzyme |  |  | 2,17E-08 | -1,529 |  |  |
| 208762_at | SUMO1 | SMT3 suppressor of mif two 3 homolog 1 (S. cerevisiae) | enzyme | 2,73E-12 | -1,552 | 2,73E-12 | -1,978 | 2,73E-12 | -1,720 |
| 208764_s_at | ATP5G2 | ATP synthase, H+ transporting, mitochondrial F0 complex, subunit C2 (subunit 9) | transporter | 1,39E-13 | -1,450 | 1,39E-13 | -1,837 | 1,39E-13 | -1,455 |
| 208766_s_at | HNRNPR | heterogeneous nuclear ribonucleoprotein R | other |  |  | 6,70E-09 | -1,498 |  |  |
| 208767_s_at | LAPTM4B | lysosomal protein transmembrane 4 beta | other |  |  | 7,57E-10 | -1,585 |  |  |
| 208774_at | CSNK1D | casein kinase 1, delta | kinase | 1,68E-12 | -1,686 | 1,68E-12 | -2,014 |  |  |
| 208779_x_at | DDR1 | discoidin domain receptor tyrosine kinase 1 | kinase |  |  | 7,49E-09 | -1,785 |  |  |
| 208781_x_at | SNX3 | sorting nexin 3 | transporter |  |  | 6,70E-09 | -1,618 | 6,70E-09 | -1,341 |
| 208784_s_at | KLHDC3 | kelch domain containing 3 | other | 6,14E-13 | -2,134 | 6,14E-13 | -2,512 | 6,14E-13 | -1,542 |
| 208785_s_at | MAP1LC3B | microtubule-associated protein 1 light chain 3 beta | other | 2,13E-09 | -1,316 | 2,13E-09 | -1,600 |  |  |
| 208786_s_at | MAP1LC3B | microtubule-associated protein 1 light chain 3 beta | other |  |  | 1,12E-09 | -1,681 |  |  |
| 208787_at | MRPL3 | mitochondrial ribosomal protein L3 | other |  |  | 5,26E-08 | -1,417 |  |  |
| 208792_s_at | CLU | clusterin | other | 4,87E-14 | -1,654 | 4,87E-14 | -1,710 |  |  |
| 208796_s_at | CCNG1 | cyclin G1 | other |  |  | 5,92E-09 | -1,516 |  |  |
| 208800_at | SRP72 | signal recognition particle 72kDa | kinase | 9,31E-11 | -1,502 | 9,31E-11 | -1,661 | 9,31E-11 | -1,422 |
| 208801_at | SRP72 | signal recognition particle 72kDa | kinase |  |  | 7,24E-08 | -1,515 |  |  |
| 208804_s_at | SFRS6 | splicing factor, arginine/serine-rich 6 | other | 3,80E-12 | -1,385 | 3,80E-12 | -1,554 |  |  |
| 208808_s_at | HMGB2 | high-mobility group box 2 | transcription regulator | 4,97E-11 | -1,395 | 4,97E-11 | -1,488 | 4,97E-11 | -1,326 |
| 208812_x_at | HLA-C | major histocompatibility complex, class I, C | transmembrane receptor | 3,09E-13 | -1,356 | 3,09E-13 | -1,907 | 3,09E-13 | -1,431 |
| 208814_at | HSPA4 | heat shock 70kDa protein 4 | other |  |  |  |  | 2,64E-08 | 2,217 |
| 208817_at | COMT | catechol-O-methyltransferase | enzyme |  |  | 2,55E-10 | -1,820 |  |  |
| 208818_s_at | COMT | catechol-O-methyltransferase | enzyme |  |  | 5,32E-10 | -1,780 |  |  |
| 208828_at | POLE3 | polymerase (DNA directed), epsilon 3 (p17 subunit) | enzyme | 4,66E-09 | -1,458 | 4,66E-09 | -1,533 | 4,66E-09 | -1,326 |
| 208831_x_at | SUPT6H | suppressor of Ty 6 homolog (S. cerevisiae) | transcription regulator |  |  | 2,58E-10 | -2,154 | 2,58E-10 | -1,503 |
| 208837_at | TMED3 | transmembrane emp24 protein transport domain containing 3 | transporter | 1,83E-14 | -1,615 | 1,83E-14 | -2,047 | 1,83E-14 | -1,508 |
| 208839_s_at | CAND1 | cullin-associated and neddylation-dissociated 1 | transcription regulator | 3,54E-11 | -1,509 | 3,54E-11 | -1,651 |  |  |
| 208841_s_at | G3BP2 | GTPase activating protein (SH3 domain) binding protein 2 | enzyme |  |  | 3,76E-09 | -1,533 |  |  |
| 208846_s_at | VDAC3 | voltage-dependent anion channel 3 | ion channel | 7,55E-14 | -1,482 | 7,55E-14 | -1,697 | 7,55E-14 | -1,432 |
| 208848_at | ADH5 (includes EG:128) | alcohol dehydrogenase 5 (class III), chi polypeptide | enzyme | 3,99E-09 | -1,415 | 3,99E-09 | -1,607 | 3,99E-09 | -1,460 |
| 208858_s_at | FAM62A | family with sequence similarity 62 (C2 domain containing), member A | other |  |  | 9,46E-11 | -2,119 |  |  |
| 208859_s_at | ATRX | alpha thalassemia/mental retardation syndrome X-linked (RAD54 homolog, S. cerevisiae) | transcription regulator |  |  |  |  | 7,35E-08 | 1,868 |
| 208861_s_at | ATRX | alpha thalassemia/mental retardation syndrome X-linked (RAD54 homolog, S. cerevisiae) | transcription regulator |  |  | 1,06E-08 | -1,679 |  |  |
| 208868_s_at | GABARAPL1 | GABA(A) receptor-associated protein like 1 | other | 4,40E-08 | -1,830 |  |  |  |  |
| 208869_s_at | GABARAPL1 | GABA(A) receptor-associated protein like 1 | other | 3,85E-11 | -1,527 | 3,85E-11 | -1,950 |  |  |
| 208870_x_at | ATP5C1 | ATP synthase, H+ transporting, mitochondrial F1 complex, gamma polypeptide 1 | transporter |  |  | 2,47E-07 | -1,455 |  |  |
| 208872_s_at | REEP5 | receptor accessory protein 5 | transporter |  |  | 1,73E-12 | -1,707 |  |  |
| 208873_s_at | REEP5 | receptor accessory protein 5 | transporter |  |  | 1,88E-09 | -1,548 |  |  |
| 208877_at | PAK2 | p21 protein (Cdc42/Rac)-activated kinase 2 | kinase |  |  | 8,88E-14 | -1,895 |  |  |
| 208880_s_at | PRPF6 | PRP6 pre-mRNA processing factor 6 homolog (S. cerevisiae) | transcription regulator |  |  | 8,11E-09 | -1,823 |  |  |
| 208890_s_at | PLXNB2 | plexin B2 | other |  |  | 3,94E-08 | -1,756 |  |  |
| 208891_at | DUSP6 | dual specificity phosphatase 6 | phosphatase |  |  |  |  | 1,83E-14 | 1,882 |
| 208892_s_at | DUSP6 | dual specificity phosphatase 6 | phosphatase | 1,83E-14 | 1,466 |  |  | 1,83E-14 | 1,850 |
| 208893_s_at | DUSP6 | dual specificity phosphatase 6 | phosphatase | 1,83E-14 | 1,987 |  |  | 1,83E-14 | 2,933 |
| 208896_at | DDX18 | DEAD (Asp-Glu-Ala-Asp) box polypeptide 18 | enzyme |  |  | 2,41E-08 | -1,568 |  |  |
| 208898_at | ATP6V1D | ATPase, H+ transporting, lysosomal 34kDa, V1 subunit D | transporter | 9,84E-11 | -1,425 | 9,84E-11 | -1,590 |  |  |
| 208900_s_at | TOP1 | topoisomerase (DNA) I | enzyme |  |  |  |  | 2,74E-11 | 1,616 |
| 208910_s_at | C1QBP | complement component 1, q subcomponent binding protein | other | 3,79E-11 | -1,263 | 3,79E-11 | -1,604 |  |  |
| 208912_s_at | CNP | 2',3'-cyclic nucleotide 3' phosphodiesterase | enzyme | 1,11E-08 | -1,445 | 1,11E-08 | -1,869 |  |  |
| 208916_at | SLC1A5 | solute carrier family 1 (neutral amino acid transporter), member 5 | transporter | 1,83E-14 | -1,832 | 1,83E-14 | -2,970 | 1,83E-14 | -1,672 |
| 208918_s_at | NADK (includes EG:65220) | NAD kinase | kinase |  |  | 1,17E-07 | -1,546 |  |  |
| 208919_s_at | NADK (includes EG:65220) | NAD kinase | kinase |  |  | 6,01E-12 | -2,008 |  |  |
| 208921_s_at | SRI | sorcin | transporter |  |  | 8,00E-12 | -1,780 |  |  |
| 208926_at | NEU1 | sialidase 1 (lysosomal sialidase) | enzyme |  |  | 3,71E-10 | -1,588 | 3,71E-10 | -1,315 |
| 208938_at | PRCC | papillary renal cell carcinoma (translocation-associated) | other |  |  | 1,36E-09 | -2,067 |  |  |
| 208944_at | TGFBR2 | transforming growth factor, beta receptor II (70/80kDa) | kinase |  |  | 1,16E-11 | -1,603 |  |  |
| 208946_s_at | BECN1 | beclin 1, autophagy related | other |  |  | 7,40E-09 | -1,681 |  |  |
| 208950_s_at | ALDH7A1 | aldehyde dehydrogenase 7 family, member A1 | enzyme | 1,85E-08 | -1,426 | 1,85E-08 | -1,503 |  |  |
| 208951_at | ALDH7A1 | aldehyde dehydrogenase 7 family, member A1 | enzyme | 2,35E-09 | -1,370 | 2,35E-09 | -1,600 |  |  |
| 208953_at | LARP5 | La ribonucleoprotein domain family, member 5 | other |  |  | 1,37E-08 | -1,634 |  |  |
| 208955_at | DUT (includes EG:1854) | deoxyuridine triphosphatase | enzyme | 3,25E-11 | -1,592 | 3,25E-11 | -1,933 | 3,25E-11 | -1,628 |
| 208961_s_at | KLF6 | Kruppel-like factor 6 | transcription regulator |  |  |  |  | 4,69E-13 | 1,602 |
| 208967_s_at | AK2 | adenylate kinase 2 | kinase |  |  | 5,29E-08 | -1,574 |  |  |
| 208968_s_at | CIAPIN1 | cytokine induced apoptosis inhibitor 1 | other |  |  | 9,53E-10 | -1,664 |  |  |
| 208972_s_at | ATP5G1 | ATP synthase, H+ transporting, mitochondrial F0 complex, subunit C1 (subunit 9) | transporter |  |  | 1,84E-08 | -1,661 |  |  |
| 208975_s_at | KPNB1 | karyopherin (importin) beta 1 | transporter |  |  | 3,40E-10 | -1,636 |  |  |
| 208979_at | NCOA6 | nuclear receptor coactivator 6 | transcription regulator |  |  | 1,74E-08 | -1,607 |  |  |
| 208990_s_at | HNRNPH3 | heterogeneous nuclear ribonucleoprotein H3 (2H9) | other | 1,76E-10 | -1,408 | 1,76E-10 | -1,448 |  |  |
| 208996_s_at | POLR2C | polymerase (RNA) II (DNA directed) polypeptide C, 33kDa | enzyme | 3,44E-14 | -1,561 | 3,44E-14 | -1,829 | 3,44E-14 | -1,289 |
| 208997_s_at | UCP2 | uncoupling protein 2 (mitochondrial, proton carrier) | transporter |  |  | 1,76E-09 | -2,151 | 1,76E-09 | -1,860 |
| 208998_at | UCP2 | uncoupling protein 2 (mitochondrial, proton carrier) | transporter | 1,83E-14 | -1,423 | 1,83E-14 | -2,133 | 1,83E-14 | -1,932 |
| 209001_s_at | ANAPC13 | anaphase promoting complex subunit 13 | other |  |  | 4,72E-09 | -1,499 |  |  |
| 209003_at | SLC25A11 | solute carrier family 25 (mitochondrial carrier; oxoglutarate carrier), member 11 | transporter |  |  | 4,78E-09 | -1,805 |  |  |
| 209014_at | MAGED1 | melanoma antigen family D, 1 | transcription regulator | 7,73E-09 | -1,344 | 7,73E-09 | -1,639 |  |  |
| 209015_s_at | DNAJB6 | DnaJ (Hsp40) homolog, subfamily B, member 6 | transcription regulator |  |  | 2,82E-09 | -1,662 |  |  |
| 209017_s_at | LONP1 | lon peptidase 1, mitochondrial | peptidase |  |  | 2,03E-09 | -1,822 |  |  |
| 209020_at | C20ORF111 | chromosome 20 open reading frame 111 | kinase |  |  | 1,43E-08 | -1,600 |  |  |
| 209022_at | STAG2 | stromal antigen 2 | other |  |  | 1,14E-10 | -1,646 |  |  |
| 209025_s_at | SYNCRIP | synaptotagmin binding, cytoplasmic RNA interacting protein | other |  |  | 1,96E-08 | -1,494 |  |  |
| 209027_s_at | ABI1 | abl-interactor 1 | other |  |  | 1,07E-08 | -1,530 |  |  |
| 209029_at | COPS7A | COP9 constitutive photomorphogenic homolog subunit 7A (Arabidopsis) | other | 4,23E-09 | -1,555 | 4,23E-09 | -1,724 | 4,23E-09 | -1,458 |
| 209031_at | CADM1 | cell adhesion molecule 1 | other |  |  | 7,98E-08 | -1,530 |  |  |
| 209032_s_at | CADM1 | cell adhesion molecule 1 | other |  |  | 2,56E-07 | -1,438 |  |  |
| 209035_at | MDK | midkine (neurite growth-promoting factor 2) | growth factor | 7,46E-11 | -1,404 | 7,46E-11 | -1,696 | 7,46E-11 | -1,435 |
| 209036_s_at | MDH2 | malate dehydrogenase 2, NAD (mitochondrial) | enzyme |  |  | 1,29E-07 | -1,487 |  |  |
| 209043_at | PAPSS1 (includes EG:9061) | 3'-phosphoadenosine 5'-phosphosulfate synthase 1 | enzyme |  |  | 6,39E-08 | -1,528 |  |  |
| 209048_s_at | ZMYND8 | zinc finger, MYND-type containing 8 | transcription regulator |  |  | 2,68E-12 | -1,788 |  |  |
| 209058_at | EDF1 | endothelial differentiation-related factor 1 | transcription regulator |  |  | 3,06E-07 | -1,520 |  |  |
| 209064_x_at | PAIP1 | poly(A) binding protein interacting protein 1 | translation regulator |  |  | 1,08E-08 | -1,530 |  |  |
| 209066_x_at | UQCRB | ubiquinol-cytochrome c reductase binding protein | enzyme |  |  | 4,15E-10 | -1,667 |  |  |
| 209077_at | TXN2 | thioredoxin 2 | enzyme | 1,35E-10 | -1,419 | 1,35E-10 | -1,989 | 1,35E-10 | -1,588 |
| 209080_x_at | GLRX3 | glutaredoxin 3 | enzyme |  |  | 2,10E-09 | -1,565 |  |  |
| 209085_x_at | RFC1 | replication factor C (activator 1) 1, 145kDa | transcription regulator |  |  | 2,23E-07 | -1,667 |  |  |
| 209089_at | RAB5A | RAB5A, member RAS oncogene family | enzyme |  |  | 4,20E-09 | -1,515 |  |  |
| 209091_s_at | SH3GLB1 | SH3-domain GRB2-like endophilin B1 | enzyme | 2,43E-13 | -1,467 | 2,43E-13 | -1,700 | 2,43E-13 | -1,263 |
| 209093_s_at | GBA (includes EG:2629) | glucosidase, beta; acid (includes glucosylceramidase) | enzyme | 6,98E-09 | -1,780 | 6,98E-09 | -2,298 |  |  |
| 209094_at | DDAH1 | dimethylarginine dimethylaminohydrolase 1 | enzyme |  |  | 3,85E-08 | -1,463 |  |  |
| 209096_at | UBE2V2 | ubiquitin-conjugating enzyme E2 variant 2 | enzyme | 3,72E-13 | -1,371 | 3,72E-13 | -1,767 |  |  |
| 209103_s_at | UFD1L | ubiquitin fusion degradation 1 like (yeast) | peptidase |  |  | 3,13E-10 | -1,622 |  |  |
| 209104_s_at | NHP2 | NHP2 ribonucleoprotein homolog (yeast) | other |  |  | 3,04E-11 | -1,790 |  |  |
| 209108_at | TSPAN6 | tetraspanin 6 | other |  |  | 9,75E-10 | -1,561 | 9,75E-10 | -1,315 |
| 209109_s_at | TSPAN6 | tetraspanin 6 | other |  |  | 1,22E-08 | -1,567 |  |  |
| 209111_at | RNF5 (includes EG:6048) | ring finger protein 5 | other | 1,83E-14 | -1,437 | 1,83E-14 | -1,976 | 1,83E-14 | -1,709 |
| 209112_at | CDKN1B | cyclin-dependent kinase inhibitor 1B (p27, Kip1) | other | 5,55E-09 | -1,274 | 5,55E-09 | -1,444 | 5,55E-09 | -1,366 |
| 209118_s_at | TUBA1A | tubulin, alpha 1a | other |  |  |  |  | 2,50E-12 | 1,426 |
| 209120_at | NR2F2 | nuclear receptor subfamily 2, group F, member 2 | ligand-dependent nuclear receptor | 1,20E-11 | -1,342 | 1,20E-11 | -1,634 | 1,20E-11 | -1,491 |
| 209122_at | ADFP | adipose differentiation-related protein | other | 1,83E-14 | 1,584 |  |  | 1,83E-14 | 1,864 |
| 209129_at | TRIP6 | thyroid hormone receptor interactor 6 | cytokine |  |  | 1,53E-07 | -1,969 |  |  |
| 209132_s_at | COMMD4 | COMM domain containing 4 | other | 1,83E-14 | -1,517 | 1,83E-14 | -1,992 | 1,83E-14 | -1,519 |
| 209137_s_at | USP10 | ubiquitin specific peptidase 10 | peptidase | 2,13E-10 | -1,587 | 2,13E-10 | -1,844 |  |  |
| 209139_s_at | PRKRA | protein kinase, interferon-inducible double stranded RNA dependent activator | other |  |  | 2,14E-08 | -1,517 |  |  |
| 209140_x_at | HLA-B | major histocompatibility complex, class I, B | transmembrane receptor |  |  | 2,41E-09 | -1,695 |  |  |
| 209142_s_at | UBE2G1 | ubiquitin-conjugating enzyme E2G 1 (UBC7 homolog, yeast) | enzyme |  |  | 4,56E-09 | -1,566 |  |  |
| 209146_at | SC4MOL | sterol-C4-methyl oxidase-like | enzyme |  |  | 1,80E-08 | -1,464 |  |  |
| 209149_s_at | TM9SF1 | transmembrane 9 superfamily member 1 | transporter |  |  | 6,89E-08 | -1,943 |  |  |
| 209150_s_at | TM9SF1 | transmembrane 9 superfamily member 1 | transporter | 2,09E-09 | -1,379 | 2,09E-09 | -1,794 |  |  |
| 209154_at | TAX1BP3 | Tax1 (human T-cell leukemia virus type I) binding protein 3 | transcription regulator | 1,91E-12 | -1,479 | 1,91E-12 | -1,648 | 1,91E-12 | -1,432 |
| 209160_at | AKR1C3 | aldo-keto reductase family 1, member C3 (3-alpha hydroxysteroid dehydrogenase, type II) | enzyme |  |  | 8,56E-08 | -1,510 |  |  |
| 209161_at | PRPF4 | PRP4 pre-mRNA processing factor 4 homolog (yeast) | other |  |  | 4,09E-11 | -1,874 |  |  |
| 209163_at | CYB561 | cytochrome b-561 | enzyme |  |  | 1,83E-14 | -2,441 | 1,83E-14 | -1,695 |
| 209165_at | AATF | apoptosis antagonizing transcription factor | transcription regulator |  |  | 2,16E-13 | -2,052 |  |  |
| 209171_at | ITPA | inosine triphosphatase (nucleoside triphosphate pyrophosphatase) | enzyme | 1,01E-12 | -1,590 | 1,01E-12 | -1,984 |  |  |
| 209174_s_at | QRICH1 | glutamine-rich 1 | other | 1,73E-10 | -1,590 | 1,73E-10 | -1,873 |  |  |
| 209175_at | SEC23IP | SEC23 interacting protein | other | 1,83E-14 | -1,473 | 1,83E-14 | -1,830 | 1,83E-14 | -1,598 |
| 209178_at | DHX38 | DEAH (Asp-Glu-Ala-His) box polypeptide 38 | enzyme |  |  | 3,10E-08 | -2,330 |  |  |
| 209190_s_at | DIAPH1 | diaphanous homolog 1 (Drosophila) | other |  |  | 1,35E-10 | -1,648 |  |  |
| 209194_at | CETN2 | centrin, EF-hand protein, 2 | enzyme |  |  | 6,71E-11 | -1,718 |  |  |
| 209208_at | MPDU1 | mannose-P-dolichol utilization defect 1 | other | 1,79E-12 | -1,666 | 1,79E-12 | -2,386 | 1,79E-12 | -1,654 |
| 209211_at | KLF5 | Kruppel-like factor 5 (intestinal) | transcription regulator |  |  |  |  | 1,07E-10 | 1,526 |
| 209218_at | SQLE | squalene epoxidase | enzyme | 3,33E-11 | -1,633 |  |  |  |  |
| 209222_s_at | OSBPL2 | oxysterol binding protein-like 2 | other |  |  | 2,68E-12 | -2,008 |  |  |
| 209229_s_at | SAPS1 | SAPS domain family, member 1 | other | 1,15E-09 | -1,430 | 1,15E-09 | -1,835 |  |  |
| 209233_at | EMG1 | EMG1 nucleolar protein homolog (S. cerevisiae) | other |  |  | 6,61E-09 | -1,581 |  |  |
| 209263_x_at | TSPAN4 | tetraspanin 4 | other |  |  | 6,57E-08 | -2,135 |  |  |
| 209270_at | LAMB3 | laminin, beta 3 | transporter | 1,83E-14 | -1,916 | 1,83E-14 | -2,668 | 1,83E-14 | -1,880 |
| 209271_at | BPTF | bromodomain PHD finger transcription factor | transcription regulator | 9,06E-09 | -1,727 | 9,06E-09 | -1,831 | 9,06E-09 | -1,465 |
| 209272_at | NAB1 | NGFI-A binding protein 1 (EGR1 binding protein 1) | transcription regulator |  |  | 2,79E-09 | -1,628 |  |  |
| 209288_s_at | CDC42EP3 | CDC42 effector protein (Rho GTPase binding) 3 | other | 1,33E-09 | -1,593 | 1,33E-09 | -1,720 |  |  |
| 209296_at | PPM1B | protein phosphatase 1B (formerly 2C), magnesium-dependent, beta isoform | phosphatase | 1,13E-09 | -1,412 | 1,13E-09 | -1,441 |  |  |
| 209297_at | ITSN1 | intersectin 1 (SH3 domain protein) | other |  |  |  |  | 2,15E-07 | -1,474 |
| 209298_s_at | ITSN1 | intersectin 1 (SH3 domain protein) | other |  |  | 6,41E-09 | -1,627 |  |  |
| 209307_at | SWAP70 | SWAP-70 protein | other |  |  | 2,78E-08 | -1,576 |  |  |
| 209313_at | GPN1 | GPN-loop GTPase 1 | transcription regulator |  |  | 4,33E-10 | -1,802 |  |  |
| 209326_at | SLC35A2 | solute carrier family 35 (UDP-galactose transporter), member A2 | transporter | 2,15E-11 | -1,543 | 2,15E-11 | -1,835 | 2,15E-11 | -1,346 |
| 209330_s_at | HNRNPD | heterogeneous nuclear ribonucleoprotein D (AU-rich element RNA binding protein 1, 37kDa) | transcription regulator | 1,83E-14 | -1,618 | 1,83E-14 | -1,922 | 1,83E-14 | -1,495 |
| 209345_s_at | PI4K2A | phosphatidylinositol 4-kinase type 2 alpha | kinase |  |  | 4,96E-09 | -1,917 |  |  |
| 209362_at | MED21 | mediator complex subunit 21 | transcription regulator | 3,29E-11 | -1,404 | 3,29E-11 | -1,681 |  |  |
| 209366_x_at | CYB5A | cytochrome b5 type A (microsomal) | enzyme |  |  | 2,73E-08 | -1,552 |  |  |
| 209369_at | ANXA3 | annexin A3 | enzyme | 2,60E-09 | -1,299 | 2,60E-09 | -1,545 |  |  |
| 209380_s_at | ABCC5 | ATP-binding cassette, sub-family C (CFTR/MRP), member 5 | transporter | 1,38E-10 | -1,796 |  |  |  |  |
| 209384_at | PROSC | proline synthetase co-transcribed homolog (bacterial) | enzyme |  |  | 1,55E-12 | -1,974 |  |  |
| 209385_s_at | PROSC | proline synthetase co-transcribed homolog (bacterial) | enzyme | 1,83E-14 | -1,522 | 1,83E-14 | -1,899 |  |  |
| 209386_at | TM4SF1 | transmembrane 4 L six family member 1 | other |  |  |  |  | 1,83E-14 | 1,340 |
| 209391_at | DPM2 | dolichyl-phosphate mannosyltransferase polypeptide 2, regulatory subunit | enzyme | 6,17E-12 | -1,744 | 6,17E-12 | -2,046 |  |  |
| 209406_at | BAG2 | BCL2-associated athanogene 2 | other |  |  | 1,90E-09 | -1,565 |  |  |
| 209412_at | TRAPPC10 | trafficking protein particle complex 10 | transporter |  |  | 1,34E-10 | -1,873 |  |  |
| 209418_s_at | THOC5 | THO complex 5 | other |  |  | 5,38E-10 | -2,096 |  |  |
| 209421_at | MSH2 | mutS homolog 2, colon cancer, nonpolyposis type 1 (E. coli) | enzyme |  |  | 8,89E-10 | -1,585 | 8,89E-10 | -1,357 |
| 209436_at | SPON1 | spondin 1, extracellular matrix protein | other |  |  | 1,81E-10 | -1,874 |  |  |
| 209445_x_at | C7ORF44 | chromosome 7 open reading frame 44 | other |  |  | 5,61E-08 | -1,616 |  |  |
| 209449_at | LSM2 | LSM2 homolog, U6 small nuclear RNA associated (S. cerevisiae) | other |  |  | 9,03E-08 | -1,588 |  |  |
| 209450_at | OSGEP | O-sialoglycoprotein endopeptidase | peptidase |  |  | 1,25E-08 | -1,624 | 1,25E-08 | -1,626 |
| 209451_at | TANK | TRAF family member-associated NFKB activator | other |  |  | 5,72E-10 | -1,768 |  |  |
| 209454_s_at | TEAD3 | TEA domain family member 3 | transcription regulator |  |  | 2,72E-09 | -2,099 |  |  |
| 209457_at | DUSP5 | dual specificity phosphatase 5 | phosphatase |  |  |  |  | 8,47E-11 | 1,820 |
| 209471_s_at | FNTA | farnesyltransferase, CAAX box, alpha | enzyme |  |  | 2,57E-10 | -1,715 |  |  |
| 209478_at | STRA13 | stimulated by retinoic acid 13 homolog (mouse) | other | 1,83E-14 | -1,637 | 1,83E-14 | -2,100 | 1,83E-14 | -1,514 |
| 209487_at | RBPMS | RNA binding protein with multiple splicing | other | 1,83E-14 | -1,446 | 1,83E-14 | -1,657 | 1,83E-14 | -1,907 |
| 209492_x_at | ATP5I | ATP synthase, H+ transporting, mitochondrial F0 complex, subunit E | transporter |  |  | 2,36E-09 | -1,710 |  |  |
| 209504_s_at | PLEKHB1 | pleckstrin homology domain containing, family B (evectins) member 1 | other |  |  | 2,45E-08 | -2,814 |  |  |
| 209507_at | RPA3 | replication protein A3, 14kDa | other |  |  | 3,32E-10 | -1,603 | 3,32E-10 | -1,280 |
| 209512_at | HSDL2 | hydroxysteroid dehydrogenase like 2 | transporter |  |  | 2,41E-08 | -1,713 |  |  |
| 209517_s_at | ASH2L | ash2 (absent, small, or homeotic)-like (Drosophila) | transcription regulator |  |  | 1,11E-08 | -1,700 | 1,11E-08 | -1,400 |
| 209518_at | SMARCD1 | SWI/SNF related, matrix associated, actin dependent regulator of chromatin, subfamily d, member 1 | transcription regulator |  |  | 9,45E-09 | -1,910 |  |  |
| 209520_s_at | NCBP1 (includes EG:4686) | nuclear cap binding protein subunit 1, 80kDa | other |  |  | 1,09E-09 | -1,659 |  |  |
| 209528_s_at | TELO2 | TEL2, telomere maintenance 2, homolog (S. cerevisiae) | other |  |  | 3,59E-09 | -2,699 | 3,59E-09 | -1,720 |
| 209529_at | PPAP2C | phosphatidic acid phosphatase type 2C | phosphatase |  |  | 2,48E-08 | -1,944 |  |  |
| 209549_s_at | DGUOK | deoxyguanosine kinase | kinase |  |  | 8,34E-09 | -1,546 |  |  |
| 209572_s_at | EED | embryonic ectoderm development | transcription regulator |  |  | 1,78E-08 | -1,578 |  |  |
| 209580_s_at | MBD4 | methyl-CpG binding domain protein 4 | enzyme |  |  | 3,92E-10 | -1,717 | 3,92E-10 | -1,363 |
| 209586_s_at | PRUNE | prune homolog (Drosophila) | enzyme | 9,19E-11 | -1,441 | 9,19E-11 | -1,982 |  |  |
| 209593_s_at | TOR1B | torsin family 1, member B (torsin B) | other |  |  | 2,27E-09 | -1,737 | 2,27E-09 | -1,371 |
| 209605_at | TST | thiosulfate sulfurtransferase (rhodanese) | enzyme | 2,89E-08 | -1,424 | 2,89E-08 | -1,690 |  |  |
| 209608_s_at | ACAT2 | acetyl-Coenzyme A acetyltransferase 2 | enzyme | 5,81E-12 | -1,380 | 5,81E-12 | -1,707 | 5,81E-12 | -1,324 |
| 209609_s_at | MRPL9 | mitochondrial ribosomal protein L9 | translation regulator |  |  | 3,08E-09 | -1,548 |  |  |
| 209623_at | MCCC2 | methylcrotonoyl-Coenzyme A carboxylase 2 (beta) | enzyme |  |  | 1,83E-14 | -1,843 | 1,83E-14 | -1,593 |
| 209624_s_at | MCCC2 | methylcrotonoyl-Coenzyme A carboxylase 2 (beta) | enzyme | 1,83E-14 | -1,693 | 1,83E-14 | -2,032 | 1,83E-14 | -1,712 |
| 209628_at | NXT2 | nuclear transport factor 2-like export factor 2 | transporter | 1,52E-07 | -1,426 |  |  |  |  |
| 209630_s_at | FBXW2 | F-box and WD repeat domain containing 2 | enzyme |  |  | 1,16E-07 | -1,567 |  |  |
| 209642_at | BUB1 | budding uninhibited by benzimidazoles 1 homolog (yeast) | kinase | 2,16E-13 | -1,451 | 2,16E-13 | -1,643 | 2,16E-13 | -1,348 |
| 209660_at | TTR | transthyretin | transporter | 1,83E-14 | -1,481 | 1,83E-14 | -2,090 | 1,83E-14 | -1,761 |
| 209662_at | CETN3 | centrin, EF-hand protein, 3 (CDC31 homolog, yeast) | other | 2,12E-10 | -1,365 | 2,12E-10 | -1,537 |  |  |
| 209666_s_at | CHUK | conserved helix-loop-helix ubiquitous kinase | kinase |  |  | 1,27E-09 | -1,656 |  |  |
| 209678_s_at | PRKCI | protein kinase C, iota | kinase |  |  |  |  | 1,28E-08 | 1,458 |
| 209679_s_at | LOC57228 | small trans-membrane and glycosylated protein | other | 1,83E-14 | -1,711 | 1,83E-14 | -2,151 | 1,83E-14 | -1,634 |
| 209691_s_at | DOK4 | docking protein 4 | other |  |  | 1,43E-08 | -1,734 |  |  |
| 209694_at | PTS | 6-pyruvoyltetrahydropterin synthase | enzyme |  |  | 3,32E-08 | -1,555 |  |  |
| 209709_s_at | HMMR | hyaluronan-mediated motility receptor (RHAMM) | other |  |  | 7,81E-11 | -1,633 | 7,81E-11 | -1,341 |
| 209714_s_at | CDKN3 | cyclin-dependent kinase inhibitor 3 | phosphatase | 1,83E-14 | -1,506 | 1,83E-14 | -1,999 | 1,83E-14 | -1,692 |
| 209735_at | ABCG2 | ATP-binding cassette, sub-family G (WHITE), member 2 | transporter |  |  |  |  | 1,83E-14 | 2,082 |
| 209757_s_at | MYCN | v-myc myelocytomatosis viral related oncogene, neuroblastoma derived (avian) | transcription regulator | 1,94E-11 | -1,824 | 1,94E-11 | -1,669 | 1,94E-11 | -1,920 |
| 209760_at | KIAA0922 | KIAA0922 | other | 7,13E-10 | -1,636 | 7,13E-10 | -1,927 | 7,13E-10 | -1,662 |
| 209786_at | HMGN4 | high mobility group nucleosomal binding domain 4 | other |  |  | 8,21E-09 | -1,641 |  |  |
| 209796_s_at | CNPY2 | canopy 2 homolog (zebrafish) | other |  |  | 1,55E-10 | -1,645 |  |  |
| 209806_at | HIST1H2BK | histone cluster 1, H2bk | other |  |  | 8,93E-09 | -1,511 |  |  |
| 209815_at | PTCH1 | patched homolog 1 (Drosophila) | transmembrane receptor |  |  |  |  | 9,43E-08 | -1,496 |
| 209852_x_at | PSME3 | proteasome (prosome, macropain) activator subunit 3 (PA28 gamma; Ki) | peptidase |  |  | 1,47E-08 | -1,721 |  |  |
| 209861_s_at | METAP2 (includes EG:10988) | methionyl aminopeptidase 2 | peptidase |  |  | 7,51E-10 | -1,613 |  |  |
| 209862_s_at | CEP57 | centrosomal protein 57kDa | other |  |  | 3,57E-09 | -1,668 |  |  |
| 209891_at | SPC25 | SPC25, NDC80 kinetochore complex component, homolog (S. cerevisiae) | other |  |  | 5,37E-08 | -1,624 |  |  |
| 209904_at | TNNC1 | troponin C type 1 (slow) | other |  |  | 1,65E-08 | -2,032 |  |  |
| 209916_at | DHTKD1 | dehydrogenase E1 and transketolase domain containing 1 | enzyme |  |  | 5,73E-08 | -1,732 |  |  |
| 209932_s_at | DUT (includes EG:1854) | deoxyuridine triphosphatase | enzyme |  |  | 1,35E-09 | -1,631 |  |  |
| 209980_s_at | SHMT1 | serine hydroxymethyltransferase 1 (soluble) | enzyme | 6,26E-14 | -1,504 | 6,26E-14 | -2,045 | 6,26E-14 | -1,748 |
| 210004_at | OLR1 | oxidized low density lipoprotein (lectin-like) receptor 1 | transmembrane receptor | 4,96E-08 | -1,583 |  |  |  |  |
| 210006_at | ABHD14A | abhydrolase domain containing 14A | enzyme | 5,16E-10 | -1,582 | 5,16E-10 | -2,234 | 5,16E-10 | -1,709 |
| 210010_s_at | SLC25A1 | solute carrier family 25 (mitochondrial carrier; citrate transporter), member 1 | transporter |  |  | 5,13E-11 | -2,018 |  |  |
| 210011_s_at | EWSR1 | Ewing sarcoma breakpoint region 1 | other | 1,07E-12 | -1,631 | 1,07E-12 | -2,127 | 1,07E-12 | -1,436 |
| 210014_x_at | IDH3B | isocitrate dehydrogenase 3 (NAD+) beta | enzyme |  |  | 1,52E-12 | -1,940 |  |  |
| 210024_s_at | UBE2E3 | ubiquitin-conjugating enzyme E2E 3 (UBC4/5 homolog, yeast) | enzyme |  |  | 1,73E-10 | -1,689 | 1,73E-10 | -1,320 |
| 210052_s_at | TPX2 | TPX2, microtubule-associated, homolog (Xenopus laevis) | other |  |  | 8,08E-09 | -1,613 |  |  |
| 210058_at | MAPK13 | mitogen-activated protein kinase 13 | kinase |  |  | 2,92E-09 | -1,765 |  |  |
| 210074_at | CTSL2 | cathepsin L2 | peptidase | 1,25E-08 | -1,428 | 1,25E-08 | -1,530 |  |  |
| 210085_s_at | ANXA9 | annexin A9 | transmembrane receptor | 1,05E-12 | -1,946 | 1,05E-12 | -2,492 | 1,05E-12 | -2,898 |
| 210092_at | MAGOH | mago-nashi homolog, proliferation-associated (Drosophila) | other | 1,83E-14 | -1,779 | 1,83E-14 | -2,750 | 1,83E-14 | -1,949 |
| 210093_s_at | MAGOH | mago-nashi homolog, proliferation-associated (Drosophila) | other | 1,83E-14 | -1,433 | 1,83E-14 | -2,573 | 1,83E-14 | -1,484 |
| 210097_s_at | NOL7 | nucleolar protein 7, 27kDa | other |  |  | 6,47E-08 | -1,440 |  |  |
| 210101_x_at | SH3GLB1 | SH3-domain GRB2-like endophilin B1 | enzyme |  |  | 9,76E-12 | -1,839 | 9,76E-12 | -1,371 |
| 210117_at | SPAG1 | sperm associated antigen 1 | other | 6,26E-14 | -1,633 | 6,26E-14 | -1,812 |  |  |
| 210125_s_at | BANF1 | barrier to autointegration factor 1 | other |  |  | 3,34E-10 | -1,851 |  |  |
| 210130_s_at | TM7SF2 | transmembrane 7 superfamily member 2 | enzyme |  |  | 3,81E-09 | -2,130 |  |  |
| 210131_x_at | SDHC | succinate dehydrogenase complex, subunit C, integral membrane protein, 15kDa | enzyme | 1,83E-14 | -2,595 | 1,83E-14 | -3,735 | 1,83E-14 | -2,287 |
| 210139_s_at | PMP22 | peripheral myelin protein 22 | other | 1,83E-14 | -2,105 | 1,83E-14 | -2,238 | 1,83E-14 | -2,498 |
| 210142_x_at | FLOT1 | flotillin 1 | other |  |  | 3,35E-09 | -1,712 |  |  |
| 210149_s_at | ATP5H (includes EG:10476) | ATP synthase, H+ transporting, mitochondrial F0 complex, subunit d | transporter |  |  | 5,61E-10 | -1,524 |  |  |
| 210153_s_at | ME2 | malic enzyme 2, NAD(+)-dependent, mitochondrial | enzyme |  |  | 3,29E-07 | -1,607 |  |  |
| 210156_s_at | PCMT1 | protein-L-isoaspartate (D-aspartate) O-methyltransferase | enzyme |  |  | 1,20E-12 | -1,852 |  |  |
| 210202_s_at | BIN1 | bridging integrator 1 | other |  |  | 1,42E-07 | -2,083 |  |  |
| 210208_x_at | BAT3 | HLA-B associated transcript 3 | enzyme |  |  | 1,37E-09 | -1,795 |  |  |
| 210220_at | FZD2 | frizzled homolog 2 (Drosophila) | G-protein coupled receptor | 1,83E-14 | -1,444 | 1,83E-14 | -1,853 | 1,83E-14 | -1,923 |
| 210235_s_at | PPFIA1 | protein tyrosine phosphatase, receptor type, f polypeptide (PTPRF), interacting protein (liprin), alpha 1 | phosphatase |  |  | 7,24E-08 | -1,852 |  |  |
| 210243_s_at | B4GALT3 | UDP-Gal:betaGlcNAc beta 1,4- galactosyltransferase, polypeptide 3 | enzyme |  |  | 4,99E-12 | -2,071 |  |  |
| 210250_x_at | ADSL | adenylosuccinate lyase | enzyme |  |  | 9,02E-09 | -1,638 |  |  |
| 210260_s_at | TNFAIP8 | tumor necrosis factor, alpha-induced protein 8 | other |  |  |  |  | 4,87E-14 | 1,522 |
| 210275_s_at | ZFAND5 | zinc finger, AN1-type domain 5 | other | 2,46E-10 | -1,343 | 2,46E-10 | -1,467 |  |  |
| 210296_s_at | PXMP3 | peroxisomal membrane protein 3, 35kDa | other | 1,39E-09 | -1,455 | 1,39E-09 | -1,707 | 1,39E-09 | -1,526 |
| 210319_x_at | MSX2 | msh homeobox 2 | transcription regulator | 4,67E-12 | -1,604 | 4,67E-12 | -2,196 | 4,67E-12 | -1,623 |
| 210320_s_at | DDX52 | DEAD (Asp-Glu-Ala-Asp) box polypeptide 52 | enzyme | 1,83E-14 | -1,745 | 1,83E-14 | -1,978 | 1,83E-14 | -1,411 |
| 210337_s_at | ACLY | ATP citrate lyase | enzyme |  |  | 1,09E-10 | -1,896 |  |  |
| 210372_s_at | TPD52L1 | tumor protein D52-like 1 | other | 4,00E-12 | -1,556 | 4,00E-12 | -1,740 | 4,00E-12 | -1,766 |
| 210378_s_at | SSNA1 | Sjogren syndrome nuclear autoantigen 1 | other | 1,11E-08 | -1,519 |  |  | 1,11E-08 | -1,757 |
| 210386_s_at | MTX1 (includes EG:4580) | metaxin 1 | transporter |  |  | 2,77E-08 | -1,681 |  |  |
| 210397_at | DEFB1 | defensin, beta 1 | other | 2,43E-13 | -2,048 | 2,43E-13 | -2,669 | 2,43E-13 | -3,137 |
| 210417_s_at | PI4KB | phosphatidylinositol 4-kinase, catalytic, beta | kinase |  |  | 1,47E-11 | -2,647 |  |  |
| 210418_s_at | IDH3B | isocitrate dehydrogenase 3 (NAD+) beta | enzyme |  |  | 1,08E-11 | -1,907 |  |  |
| 210428_s_at | HGS | hepatocyte growth factor-regulated tyrosine kinase substrate | other |  |  | 9,68E-11 | -1,950 |  |  |
| 210460_s_at | PSMD4 | proteasome (prosome, macropain) 26S subunit, non-ATPase, 4 | other |  |  | 2,68E-09 | -1,682 |  |  |
| 210502_s_at | PPIE | peptidylprolyl isomerase E (cyclophilin E) | enzyme |  |  | 6,45E-10 | -1,749 |  |  |
| 210514_x_at | HLA-G | major histocompatibility complex, class I, G | transmembrane receptor |  |  | 2,01E-10 | -2,084 |  |  |
| 210524_x_at | MT1F | metallothionein 1F | other | 1,83E-14 | 2,186 |  |  |  |  |
| 210538_s_at | BIRC3 | baculoviral IAP repeat-containing 3 | enzyme |  |  |  |  | 1,83E-14 | 2,075 |
| 210543_s_at | PRKDC | protein kinase, DNA-activated, catalytic polypeptide | kinase | 8,21E-12 | -1,338 | 8,21E-12 | -1,846 |  |  |
| 210559_s_at | CDC2 | cell division cycle 2, G1 to S and G2 to M | kinase |  |  | 9,72E-08 | -1,447 |  |  |
| 210574_s_at | NUDC | nuclear distribution gene C homolog (A. nidulans) | other |  |  | 9,75E-08 | -1,628 |  |  |
| 210580_x_at | SULT1A3 | sulfotransferase family, cytosolic, 1A, phenol-preferring, member 3 | enzyme |  |  | 7,24E-09 | -1,747 |  |  |
| 210589_s_at | GBA (includes EG:2629) | glucosidase, beta; acid (includes glucosylceramidase) | enzyme | 2,24E-08 | -2,165 |  |  |  |  |
| 210616_s_at | SEC31A | SEC31 homolog A (S. cerevisiae) | other |  |  | 1,97E-08 | -1,677 |  |  |
| 210621_s_at | RASA1 | RAS p21 protein activator (GTPase activating protein) 1 | transporter |  |  | 2,60E-08 | -1,532 |  |  |
| 210624_s_at | ILVBL | ilvB (bacterial acetolactate synthase)-like | enzyme |  |  | 5,66E-11 | -2,025 | 5,66E-11 | -1,696 |
| 210633_x_at | KRT10 | keratin 10 | other | 2,25E-13 | -1,585 | 2,25E-13 | -1,931 | 2,25E-13 | -1,733 |
| 210639_s_at | ATG5 | ATG5 autophagy related 5 homolog (S. cerevisiae) | other | 2,99E-11 | -1,442 | 2,99E-11 | -1,690 | 2,99E-11 | -1,518 |
| 210645_s_at | TTC3 | tetratricopeptide repeat domain 3 | other | 6,37E-10 | -1,409 | 6,37E-10 | -1,578 |  |  |
| 210749_x_at | DDR1 | discoidin domain receptor tyrosine kinase 1 | kinase | 1,83E-14 | -1,514 | 1,83E-14 | -2,163 | 1,83E-14 | -1,602 |
| 210751_s_at | RGN | regucalcin (senescence marker protein-30) | other |  |  | 1,34E-12 | -2,097 |  |  |
| 210757_x_at | DAB2 | disabled homolog 2, mitogen-responsive phosphoprotein (Drosophila) | other | 1,22E-09 | -1,463 | 1,22E-09 | -1,587 | 1,22E-09 | -1,560 |
| 210759_s_at | PSMA1 | proteasome (prosome, macropain) subunit, alpha type, 1 | peptidase |  |  | 6,23E-08 | -1,492 |  |  |
| 210792_x_at | SIVA1 | SIVA1, apoptosis-inducing factor | other |  |  | 3,03E-08 | -1,856 |  |  |
| 210793_s_at | NUP98 | nucleoporin 98kDa | transporter |  |  | 5,82E-09 | -1,565 |  |  |
| 210821_x_at | CENPA | centromere protein A | other |  |  | 2,57E-08 | -1,872 |  |  |
| 210825_s_at | PEBP1 | phosphatidylethanolamine binding protein 1 | other |  |  | 2,68E-11 | -1,707 |  |  |
| 210830_s_at | PON2 | paraoxonase 2 | enzyme |  |  | 3,14E-08 | -1,536 |  |  |
| 210845_s_at | PLAUR | plasminogen activator, urokinase receptor | transmembrane receptor |  |  |  |  | 1,83E-14 | 3,412 |
| 210904_s_at | IL13RA1 | interleukin 13 receptor, alpha 1 | transmembrane receptor |  |  | 3,09E-09 | -1,714 |  |  |
| 210908_s_at | PFDN5 | prefoldin subunit 5 | transcription regulator |  |  | 4,66E-08 | -1,513 |  |  |
| 210926_at | ACTBL3 | actin, beta-like 3 | other |  |  |  |  | 6,34E-09 | -1,626 |
| 210929_s_at | LOC100131613 | PRO1454 | other | 2,70E-07 | -1,594 |  |  |  |  |
| 210935_s_at | WDR1 | WD repeat domain 1 | other | 1,14E-13 | -1,461 | 1,14E-13 | -2,141 | 1,14E-13 | -1,691 |
| 210949_s_at | EIF3C | eukaryotic translation initiation factor 3, subunit C | translation regulator |  |  | 2,07E-11 | -1,634 |  |  |
| 210950_s_at | FDFT1 | farnesyl-diphosphate farnesyltransferase 1 | enzyme |  |  | 1,83E-14 | -1,928 |  |  |
| 210962_s_at | AKAP9 | A kinase (PRKA) anchor protein (yotiao) 9 | other | 8,92E-11 | -1,612 | 8,92E-11 | -1,562 | 8,92E-11 | -1,450 |
| 210966_x_at | LARP1 | La ribonucleoprotein domain family, member 1 | other |  |  | 2,88E-08 | -1,614 |  |  |
| 210976_s_at | PFKM | phosphofructokinase, muscle | kinase |  |  | 3,70E-08 | -1,572 |  |  |
| 210983_s_at | MCM7 | minichromosome maintenance complex component 7 | enzyme |  |  | 7,84E-11 | -1,851 |  |  |
| 210986_s_at | TPM1 | tropomyosin 1 (alpha) | other |  |  | 4,03E-09 | -1,477 |  |  |
| 211000_s_at | IL6ST | interleukin 6 signal transducer (gp130, oncostatin M receptor) | transmembrane receptor | 1,83E-14 | 2,173 | 1,83E-14 | 2,502 | 1,83E-14 | 2,731 |
| 211023_at | PDHB | pyruvate dehydrogenase (lipoamide) beta | enzyme |  |  | 2,35E-08 | -1,565 |  |  |
| 211025_x_at | COX5B | cytochrome c oxidase subunit Vb | enzyme |  |  | 5,18E-09 | -1,702 |  |  |
| 211043_s_at | CLTB | clathrin, light chain (Lcb) | other |  |  | 2,67E-08 | -1,776 |  |  |
| 211052_s_at | TBCD | tubulin folding cofactor D | other |  |  | 1,43E-08 | -2,424 |  |  |
| 211056_s_at | SRD5A1 | steroid-5-alpha-reductase, alpha polypeptide 1 (3-oxo-5 alpha-steroid delta 4-dehydrogenase alpha 1) | enzyme | 2,32E-09 | -1,831 | 2,32E-09 | -1,981 |  |  |
| 211061_s_at | MGAT2 | mannosyl (alpha-1,6-)-glycoprotein beta-1,2-N-acetylglucosaminyltransferase | enzyme | 7,07E-11 | -1,507 | 7,07E-11 | -1,742 | 7,07E-11 | -1,444 |
| 211068_x_at | FAM21C | family with sequence similarity 21, member C | other |  |  | 6,40E-08 | -1,580 |  |  |
| 211074_at | FOLR1 | folate receptor 1 (adult) | transporter | 1,47E-10 | 1,568 | 1,47E-10 | 1,680 |  |  |
| 211090_s_at | PRPF4B | PRP4 pre-mRNA processing factor 4 homolog B (yeast) | kinase |  |  | 1,70E-08 | -1,568 |  |  |
| 211124_s_at | KITLG | KIT ligand | growth factor |  |  | 1,85E-13 | -1,997 | 1,85E-13 | -1,999 |
| 211136_s_at | CLPTM1 | cleft lip and palate associated transmembrane protein 1 | other |  |  | 3,73E-12 | -2,114 |  |  |
| 211137_s_at | ATP2C1 | ATPase, Ca++ transporting, type 2C, member 1 | transporter |  |  | 2,41E-12 | -1,805 |  |  |
| 211150_s_at | DLAT | dihydrolipoamide S-acetyltransferase | enzyme |  |  | 2,67E-08 | -1,585 |  |  |
| 211159_s_at | PPP2R5D | protein phosphatase 2, regulatory subunit B', delta isoform | phosphatase |  |  | 6,04E-10 | -2,301 | 6,04E-10 | -1,522 |
| 211160_x_at | ACTN1 | actinin, alpha 1 | other |  |  | 8,54E-13 | -2,381 |  |  |
| 211212_s_at | ORC5L | origin recognition complex, subunit 5-like (yeast) | other | 2,31E-12 | -1,689 | 2,31E-12 | -2,006 | 2,31E-12 | -1,627 |
| 211284_s_at | GRN | granulin | growth factor | 4,08E-10 | -1,553 | 4,08E-10 | -1,877 | 4,08E-10 | -1,484 |
| 211297_s_at | CDK7 | cyclin-dependent kinase 7 | kinase |  |  | 3,02E-09 | -1,488 |  |  |
| 211299_s_at | FLOT2 | flotillin 2 | other |  |  | 6,14E-08 | -2,144 | 6,14E-08 | -1,606 |
| 211318_s_at | RAE1 | RAE1 RNA export 1 homolog (S. pombe) | other | 2,36E-10 | -1,417 | 2,36E-10 | -1,583 |  |  |
| 211375_s_at | ILF3 | interleukin enhancer binding factor 3, 90kDa | transcription regulator |  |  | 2,99E-13 | -1,829 | 2,99E-13 | -1,338 |
| 211376_s_at | NSMCE4A | non-SMC element 4 homolog A (S. cerevisiae) | other |  |  | 3,64E-13 | -1,967 | 3,64E-13 | -1,461 |
| 211432_s_at | TYRO3 | TYRO3 protein tyrosine kinase | kinase |  |  | 1,21E-10 | -2,269 |  |  |
| 211456_x_at | MT1P2 | metallothionein 1 pseudogene 2 | other | 1,83E-14 | 4,603 |  |  | 1,83E-14 | 2,777 |
| 211478_s_at | DPP4 | dipeptidyl-peptidase 4 | peptidase |  |  | 2,17E-11 | -1,767 |  |  |
| 211501_s_at | EIF3B | eukaryotic translation initiation factor 3, subunit B | translation regulator |  |  | 4,44E-08 | -1,632 |  |  |
| 211505_s_at | STAU1 | staufen, RNA binding protein, homolog 1 (Drosophila) | transporter |  |  | 4,70E-10 | -1,572 | 4,70E-10 | -1,478 |
| 211509_s_at | RTN4 | reticulon 4 | other |  |  | 8,09E-08 | -1,463 |  |  |
| 211518_s_at | BMP4 | bone morphogenetic protein 4 | growth factor |  |  |  |  | 3,32E-07 | -1,744 |
| 211519_s_at | KIF2C | kinesin family member 2C | other |  |  | 7,70E-11 | -1,866 |  |  |
| 211528_x_at | HLA-G | major histocompatibility complex, class I, G | transmembrane receptor | 1,75E-12 | -1,384 | 1,75E-12 | -1,901 |  |  |
| 211529_x_at | HLA-G | major histocompatibility complex, class I, G | transmembrane receptor | 1,12E-12 | -1,374 | 1,12E-12 | -1,839 | 1,12E-12 | -1,667 |
| 211530_x_at | HLA-G | major histocompatibility complex, class I, G | transmembrane receptor |  |  | 1,21E-10 | -2,057 |  |  |
| 211548_s_at | HPGD | hydroxyprostaglandin dehydrogenase 15-(NAD) | enzyme | 7,64E-13 | -1,700 | 7,64E-13 | -1,570 | 7,64E-13 | -1,493 |
| 211549_s_at | HPGD | hydroxyprostaglandin dehydrogenase 15-(NAD) | enzyme | 2,83E-12 | -1,680 | 2,83E-12 | -1,728 | 2,83E-12 | -1,720 |
| 211559_s_at | CCNG2 | cyclin G2 | other | 1,67E-10 | -1,597 | 1,67E-10 | -1,882 | 1,67E-10 | -2,030 |
| 211561_x_at | MAPK14 | mitogen-activated protein kinase 14 | kinase |  |  | 1,54E-10 | -1,934 |  |  |
| 211569_s_at | HADH | hydroxyacyl-Coenzyme A dehydrogenase | enzyme |  |  | 5,92E-10 | -1,804 |  |  |
| 211574_s_at | CD46 | CD46 molecule, complement regulatory protein | other |  |  | 2,02E-08 | -1,724 |  |  |
| 211575_s_at | UBE3A | ubiquitin protein ligase E3A | enzyme | 3,44E-12 | -1,553 | 3,44E-12 | -1,876 |  |  |
| 211609_x_at | PSMD4 | proteasome (prosome, macropain) 26S subunit, non-ATPase, 4 | other |  |  | 8,32E-08 | -1,561 |  |  |
| 211623_s_at | FBL | fibrillarin | other |  |  | 1,87E-08 | -1,631 |  |  |
| 211630_s_at | GSS | glutathione synthetase | enzyme | 1,83E-14 | -1,423 | 1,83E-14 | -2,074 | 1,83E-14 | -1,390 |
| 211651_s_at | LAMB1 | laminin, beta 1 | other |  |  | 8,26E-11 | -1,650 |  |  |
| 211658_at | PRDX2 | peroxiredoxin 2 | enzyme | 3,23E-11 | -1,374 | 3,23E-11 | -1,930 | 3,23E-11 | -1,514 |
| 211678_s_at | RNF114 | ring finger protein 114 | other |  |  | 3,38E-08 | -1,480 |  |  |
| 211712_s_at | ANXA9 | annexin A9 | transmembrane receptor | 1,83E-14 | -1,994 | 1,83E-14 | -2,409 | 1,83E-14 | -3,665 |
| 211725_s_at | BID | BH3 interacting domain death agonist | other |  |  | 1,78E-12 | -2,073 |  |  |
| 211730_s_at | POLR2L (includes EG:5441) | polymerase (RNA) II (DNA directed) polypeptide L, 7.6kDa | enzyme |  |  | 1,83E-14 | -1,859 | 1,83E-14 | -1,382 |
| 211733_x_at | SCP2 | sterol carrier protein 2 | transporter | 4,74E-09 | -1,403 | 4,74E-09 | -1,503 | 4,74E-09 | -1,332 |
| 211747_s_at | LSM5 | LSM5 homolog, U6 small nuclear RNA associated (S. cerevisiae) | other | 2,35E-12 | -1,282 | 2,35E-12 | -1,586 | 2,35E-12 | -1,354 |
| 211749_s_at | VAMP3 | vesicle-associated membrane protein 3 (cellubrevin) | other | 3,46E-11 | -1,316 | 3,46E-11 | -1,689 |  |  |
| 211755_s_at | ATP5F1 | ATP synthase, H+ transporting, mitochondrial F0 complex, subunit B1 | transporter |  |  | 2,70E-08 | -1,524 |  |  |
| 211758_x_at | TXNDC9 | thioredoxin domain containing 9 | other | 1,32E-12 | -1,512 | 1,32E-12 | -1,691 | 1,32E-12 | -1,335 |
| 211763_s_at | UBE2B | ubiquitin-conjugating enzyme E2B (RAD6 homolog) | enzyme |  |  | 4,96E-08 | -1,521 |  |  |
| 211779_x_at | AP2A2 | adaptor-related protein complex 2, alpha 2 subunit | transporter |  |  | 2,48E-08 | -1,639 |  |  |
| 211799_x_at | HLA-C | major histocompatibility complex, class I, C | transmembrane receptor |  |  | 4,93E-09 | -2,139 |  |  |
| 211804_s_at | CDK2 | cyclin-dependent kinase 2 | kinase | 3,64E-11 | -1,736 | 3,64E-11 | -1,918 | 3,64E-11 | -1,738 |
| 211911_x_at | HLA-B | major histocompatibility complex, class I, B | transmembrane receptor |  |  | 1,56E-12 | -1,960 |  |  |
| 211928_at | DYNC1H1 | dynein, cytoplasmic 1, heavy chain 1 | peptidase | 1,83E-14 | -1,584 | 1,83E-14 | -1,853 |  |  |
| 211931_s_at | HNRNPA3 | heterogeneous nuclear ribonucleoprotein A3 | other | 4,04E-12 | -1,478 | 4,04E-12 | -1,618 |  |  |
| 211934_x_at | GANAB | glucosidase, alpha; neutral AB | enzyme | 1,83E-14 | 3,196 | 1,83E-14 | 2,321 | 1,83E-14 | 3,693 |
| 211936_at | HSPA5 | heat shock 70kDa protein 5 (glucose-regulated protein, 78kDa) | other | 1,54E-10 | -1,265 | 1,54E-10 | -1,588 |  |  |
| 211953_s_at | IPO5 | importin 5 | transporter |  |  | 2,74E-09 | -1,583 |  |  |
| 211960_s_at | RAB7A | RAB7A, member RAS oncogene family | enzyme |  |  | 3,41E-09 | -1,678 |  |  |
| 211961_s_at | RAB7A | RAB7A, member RAS oncogene family | enzyme |  |  | 6,01E-11 | -1,672 |  |  |
| 211971_s_at | LRPPRC | leucine-rich PPR-motif containing | other |  |  | 3,36E-09 | -1,474 |  |  |
| 211975_at | ARFGAP2 | ADP-ribosylation factor GTPase activating protein 2 | other | 1,34E-09 | -1,395 | 1,34E-09 | -1,828 |  |  |
| 211979_at | GPR107 | G protein-coupled receptor 107 | G-protein coupled receptor | 1,83E-14 | -2,330 | 1,83E-14 | -2,853 | 1,83E-14 | -1,893 |
| 211985_s_at | CALM3 | calmodulin 3 (phosphorylase kinase, delta) | other |  |  | 5,66E-08 | -1,570 |  |  |
| 211998_at | H3F3A (includes EG:3020) | H3 histone, family 3A | other |  |  | 1,72E-07 | -1,813 |  |  |
| 212007_at | UBXN4 | UBX domain protein 4 | other |  |  |  |  | 2,92E-11 | 1,693 |
| 212008_at | UBXN4 | UBX domain protein 4 | other |  |  | 7,35E-11 | -1,755 |  |  |
| 212009_s_at | STIP1 | stress-induced-phosphoprotein 1 | other | 1,83E-14 | -2,594 | 1,83E-14 | -3,591 | 1,83E-14 | -2,734 |
| 212016_s_at | PTBP1 | polypyrimidine tract binding protein 1 | enzyme |  |  | 6,83E-12 | -2,343 |  |  |
| 212020_s_at | MKI67 | antigen identified by monoclonal antibody Ki-67 | other | 1,83E-14 | -1,733 | 1,83E-14 | -2,152 | 1,83E-14 | -1,600 |
| 212025_s_at | FLII | flightless I homolog (Drosophila) | other |  |  | 1,20E-10 | -2,006 |  |  |
| 212034_s_at | EXOC7 | exocyst complex component 7 | transporter |  |  | 1,76E-09 | -1,759 |  |  |
| 212047_s_at | RNF167 | ring finger protein 167 | other |  |  | 3,18E-09 | -1,996 |  |  |
| 212048_s_at | YARS | tyrosyl-tRNA synthetase | enzyme |  |  | 2,43E-13 | -1,973 |  |  |
| 212050_at | WIPF2 | WAS/WASL interacting protein family, member 2 | other |  |  | 4,39E-08 | -1,714 |  |  |
| 212055_at | C18ORF10 | chromosome 18 open reading frame 10 | other | 1,98E-10 | -1,461 | 1,98E-10 | -1,861 | 1,98E-10 | -1,512 |
| 212058_at | SR140 | U2-associated SR140 protein | other |  |  | 5,15E-10 | -1,495 |  |  |
| 212059_s_at | TRPC4AP | transient receptor potential cation channel, subfamily C, member 4 associated protein | transporter |  |  | 4,14E-09 | -1,901 |  |  |
| 212069_s_at | BAT2L | HLA-B associated transcript 2-like | other |  |  | 2,35E-10 | -2,207 |  |  |
| 212074_at | UNC84A | unc-84 homolog A (C. elegans) | other | 1,37E-09 | -1,588 | 1,37E-09 | -1,505 | 1,37E-09 | -1,392 |
| 212076_at | MLL | myeloid/lymphoid or mixed-lineage leukemia (trithorax homolog, Drosophila) | transcription regulator |  |  |  |  | 3,53E-08 | 1,594 |
| 212077_at | CALD1 | caldesmon 1 | other | 1,94E-09 | -1,416 | 1,94E-09 | -1,440 |  |  |
| 212080_at | MLL | myeloid/lymphoid or mixed-lineage leukemia (trithorax homolog, Drosophila) | transcription regulator |  |  | 4,47E-09 | -1,932 |  |  |
| 212083_at | TEX261 | testis expressed 261 | other | 3,57E-13 | -1,557 | 3,57E-13 | -1,974 | 3,57E-13 | -1,370 |
| 212085_at | SLC25A6 | solute carrier family 25 (mitochondrial carrier; adenine nucleotide translocator), member 6 | transporter |  |  | 2,39E-10 | -1,653 |  |  |
| 212088_at | PMPCA | peptidase (mitochondrial processing) alpha | peptidase |  |  | 1,34E-08 | -1,734 |  |  |
| 212094_at | PEG10 | paternally expressed 10 | other | 1,24E-09 | -1,328 | 1,24E-09 | -1,583 | 1,24E-09 | -1,403 |
| 212096_s_at | MTUS1 | mitochondrial tumor suppressor 1 | other |  |  | 2,79E-12 | -1,852 |  |  |
| 212099_at | RHOB | ras homolog gene family, member B | enzyme |  |  |  |  | 1,83E-14 | 1,768 |
| 212112_s_at | STX12 | syntaxin 12 | other | 4,18E-07 | -1,431 |  |  |  |  |
| 212114_at | LOC552889 | hypothetical protein LOC552889 | other |  |  | 7,89E-10 | -1,743 |  |  |
| 212121_at | TCTN3 | tectonic family member 3 | other |  |  | 8,22E-08 | -1,661 |  |  |
| 212124_at | ZMIZ1 | zinc finger, MIZ-type containing 1 | other | 9,68E-11 | -1,747 | 9,68E-11 | -2,160 | 9,68E-11 | -1,615 |
| 212127_at | RANGAP1 | Ran GTPase activating protein 1 | other | 4,99E-10 | -1,923 | 4,99E-10 | -2,476 | 4,99E-10 | -1,708 |
| 212131_at | LSM14A | LSM14A, SCD6 homolog A (S. cerevisiae) | other | 1,83E-14 | -1,509 | 1,83E-14 | -1,892 |  |  |
| 212137_at | LARP1 | La ribonucleoprotein domain family, member 1 | other |  |  | 4,59E-08 | -1,447 |  |  |
| 212138_at | PDS5A | PDS5, regulator of cohesion maintenance, homolog A (S. cerevisiae) | other |  |  | 6,06E-09 | -1,608 |  |  |
| 212140_at | PDS5A | PDS5, regulator of cohesion maintenance, homolog A (S. cerevisiae) | other | 1,61E-09 | -1,627 | 1,61E-09 | -1,883 |  |  |
| 212148_at | PBX1 | pre-B-cell leukemia homeobox 1 | transcription regulator | 2,92E-10 | -1,654 | 2,92E-10 | -1,889 | 2,92E-10 | -1,581 |
| 212166_at | XPO7 | exportin 7 | transporter | 2,17E-12 | -1,496 | 2,17E-12 | -1,948 |  |  |
| 212168_at | RBM12 | RNA binding motif protein 12 | other |  |  | 2,09E-09 | -1,501 |  |  |
| 212169_at | FKBP9 | FK506 binding protein 9, 63 kDa | enzyme |  |  | 1,48E-10 | -1,691 |  |  |
| 212175_s_at | AK2 | adenylate kinase 2 | kinase |  |  | 4,74E-09 | -1,889 |  |  |
| 212177_at | SFRS18 | splicing factor, arginine/serine-rich 18 | other |  |  |  |  | 4,14E-11 | 1,928 |
| 212180_at | CRKL | v-crk sarcoma virus CT10 oncogene homolog (avian)-like | kinase | 1,57E-12 | -1,566 | 1,57E-12 | -1,824 |  |  |
| 212183_at | NUDT4 | nudix (nucleoside diphosphate linked moiety X)-type motif 4 | phosphatase |  |  | 2,51E-09 | -1,571 |  |  |
| 212184_s_at | MAP3K7IP2 | mitogen-activated protein kinase kinase kinase 7 interacting protein 2 | other |  |  | 1,48E-09 | -1,582 |  |  |
| 212185_x_at | MT2A | metallothionein 2A | other | 1,83E-14 | 4,446 |  |  | 1,83E-14 | 3,010 |
| 212193_s_at | LARP1 | La ribonucleoprotein domain family, member 1 | other |  |  | 1,83E-14 | -1,959 |  |  |
| 212198_s_at | TM9SF4 | transmembrane 9 superfamily protein member 4 | transporter |  |  | 8,66E-11 | -2,213 |  |  |
| 212204_at | TMEM87A | transmembrane protein 87A | other |  |  | 5,73E-09 | -1,764 |  |  |
| 212205_at | H2AFV (includes EG:94239) | H2A histone family, member V | other | 3,19E-11 | -1,411 | 3,19E-11 | -1,558 | 3,19E-11 | -1,270 |
| 212206_s_at | H2AFV (includes EG:94239) | H2A histone family, member V | other |  |  | 8,86E-11 | -1,972 |  |  |
| 212218_s_at | FASN | fatty acid synthase | enzyme |  |  | 3,87E-11 | -1,860 |  |  |
| 212219_at | PSME4 | proteasome (prosome, macropain) activator subunit 4 | other |  |  | 4,48E-10 | -1,502 |  |  |
| 212230_at | PPAP2B | phosphatidic acid phosphatase type 2B | phosphatase | 1,83E-14 | -1,526 | 1,83E-14 | -2,362 | 1,83E-14 | -2,019 |
| 212231_at | FBXO21 | F-box protein 21 | enzyme | 1,83E-14 | -1,768 | 1,83E-14 | -1,994 | 1,83E-14 | -1,511 |
| 212242_at | TUBA4A | tubulin, alpha 4a | other |  |  | 8,73E-11 | -1,735 |  |  |
| 212250_at | MTDH | metadherin | other |  |  | 6,38E-11 | -1,660 | 6,38E-11 | -1,306 |
| 212251_at | MTDH | metadherin | other |  |  | 3,57E-13 | -1,844 |  |  |
| 212265_at | QKI | quaking homolog, KH domain RNA binding (mouse) | other |  |  | 2,81E-09 | -1,495 |  |  |
| 212266_s_at | SFRS5 | splicing factor, arginine/serine-rich 5 | other | 1,83E-14 | -1,641 | 1,83E-14 | -2,293 | 1,83E-14 | -1,359 |
| 212268_at | SERPINB1 | serpin peptidase inhibitor, clade B (ovalbumin), member 1 | other | 6,26E-14 | -1,566 | 6,26E-14 | -1,881 | 6,26E-14 | -1,488 |
| 212271_at | MAPK1 | mitogen-activated protein kinase 1 | kinase |  |  | 2,96E-08 | -1,607 |  |  |
| 212276_at | LPIN1 | lipin 1 | other |  |  | 1,31E-09 | -1,863 | 1,31E-09 | -1,473 |
| 212277_at | MTMR4 | myotubularin related protein 4 | phosphatase | 1,83E-14 | -1,903 | 1,83E-14 | -2,505 | 1,83E-14 | -1,780 |
| 212279_at | TMEM97 | transmembrane protein 97 | other | 1,83E-14 | -1,353 | 1,83E-14 | -2,372 | 1,83E-14 | -1,600 |
| 212280_x_at | ATG4B | ATG4 autophagy related 4 homolog B (S. cerevisiae) | peptidase |  |  | 7,76E-10 | -2,070 | 7,76E-10 | -1,502 |
| 212282_at | TMEM97 | transmembrane protein 97 | other |  |  | 1,30E-07 | -1,571 |  |  |
| 212287_at | SUZ12 | suppressor of zeste 12 homolog (Drosophila) | enzyme | 2,89E-10 | -1,460 | 2,89E-10 | -1,430 |  |  |
| 212290_at | SLC7A1 | solute carrier family 7 (cationic amino acid transporter, y+ system), member 1 | transporter |  |  |  |  | 3,72E-13 | 1,799 |
| 212297_at | ATP13A3 (includes EG:79572) | ATPase type 13A3 | transporter | 2,73E-10 | -1,590 |  |  |  |  |
| 212301_at | RTF1 | Rtf1, Paf1/RNA polymerase II complex component, homolog (S. cerevisiae) | other |  |  | 6,71E-09 | -1,646 |  |  |
| 212321_at | SGPL1 | sphingosine-1-phosphate lyase 1 | enzyme |  |  | 9,09E-08 | -1,676 |  |  |
| 212322_at | SGPL1 | sphingosine-1-phosphate lyase 1 | enzyme |  |  | 9,68E-09 | -1,573 |  |  |
| 212330_at | TFDP1 | transcription factor Dp-1 | transcription regulator | 6,20E-12 | -1,364 | 6,20E-12 | -1,666 | 6,20E-12 | -1,281 |
| 212334_at | GNS | glucosamine (N-acetyl)-6-sulfatase | enzyme | 1,27E-09 | -1,422 | 1,27E-09 | -1,473 | 1,27E-09 | -1,332 |
| 212338_at | MYO1D | myosin ID | other | 1,83E-14 | -1,538 | 1,83E-14 | -2,118 | 1,83E-14 | -1,383 |
| 212352_s_at | TMED10 | transmembrane emp24-like trafficking protein 10 (yeast) | transporter |  |  | 4,80E-11 | -1,738 |  |  |
| 212371_at | FAM152A | family with sequence similarity 152, member A | other | 1,83E-14 | -1,518 | 1,83E-14 | -1,923 |  |  |
| 212372_at | MYH10 | myosin, heavy chain 10, non-muscle | other |  |  | 1,43E-08 | -1,585 |  |  |
| 212378_at | GART | phosphoribosylglycinamide formyltransferase, phosphoribosylglycinamide synthetase, phosphoribosylaminoimidazole synthetase | enzyme | 3,63E-11 | -1,326 | 3,63E-11 | -1,474 |  |  |
| 212383_at | ATP6V0A1 | ATPase, H+ transporting, lysosomal V0 subunit a1 | transporter | 1,41E-10 | -1,856 | 1,41E-10 | -1,721 |  |  |
| 212386_at | TCF4 | transcription factor 4 | transcription regulator | 1,74E-13 | -1,668 | 1,74E-13 | -1,618 | 1,74E-13 | -1,817 |
| 212387_at | TCF4 | transcription factor 4 | transcription regulator | 2,83E-10 | -1,595 | 2,83E-10 | -1,992 | 2,83E-10 | -2,074 |
| 212398_at | RDX | radixin | other | 4,21E-11 | -1,410 | 4,21E-11 | -1,765 | 4,21E-11 | -1,594 |
| 212401_s_at | CDC2L2 | cell division cycle 2-like 2 (PITSLRE proteins) | kinase |  |  | 7,33E-11 | -1,695 |  |  |
| 212406_s_at | PCMTD2 | protein-L-isoaspartate (D-aspartate) O-methyltransferase domain containing 2 | enzyme | 1,23E-12 | -1,324 | 1,23E-12 | -1,813 | 1,23E-12 | -1,467 |
| 212408_at | TOR1AIP1 | torsin A interacting protein 1 | other |  |  | 3,59E-09 | -1,582 |  |  |
| 212411_at | IMP4 | IMP4, U3 small nucleolar ribonucleoprotein, homolog (yeast) | other |  |  | 1,55E-12 | -1,918 |  |  |
| 212428_at | KIAA0368 | KIAA0368 | other |  |  | 5,58E-10 | -1,628 |  |  |
| 212432_at | GRPEL1 | GrpE-like 1, mitochondrial (E. coli) | other |  |  | 4,79E-10 | -1,920 |  |  |
| 212453_at | KIAA1279 | KIAA1279 | enzyme |  |  | 6,21E-09 | -1,558 |  |  |
| 212457_at | TFE3 | transcription factor binding to IGHM enhancer 3 | transcription regulator | 1,83E-14 | -2,066 | 1,83E-14 | -2,398 | 1,83E-14 | -1,511 |
| 212459_x_at | SUCLG2 | succinate-CoA ligase, GDP-forming, beta subunit | enzyme |  |  | 2,23E-08 | -1,523 |  |  |
| 212460_at | C14ORF147 | chromosome 14 open reading frame 147 | other |  |  | 4,52E-10 | -1,700 |  |  |
| 212467_at | DNAJC13 | DnaJ (Hsp40) homolog, subfamily C, member 13 | other |  |  | 1,29E-07 | -1,609 |  |  |
| 212472_at | MICAL2 | microtubule associated monoxygenase, calponin and LIM domain containing 2 | other |  |  | 1,88E-11 | -1,728 |  |  |
| 212476_at | ACAP2 | ArfGAP with coiled-coil, ankyrin repeat and PH domains 2 | other |  |  | 1,40E-07 | -1,566 |  |  |
| 212485_at | GPATCH8 | G patch domain containing 8 | other |  |  | 8,85E-09 | -1,736 |  |  |
| 212509_s_at | MXRA7 | matrix-remodelling associated 7 | other |  |  | 1,28E-10 | -1,625 |  |  |
| 212511_at | PICALM | phosphatidylinositol binding clathrin assembly protein | other |  |  | 2,81E-08 | -1,422 | 2,81E-08 | -1,296 |
| 212514_x_at | DDX3X | DEAD (Asp-Glu-Ala-Asp) box polypeptide 3, X-linked | enzyme |  |  | 6,50E-11 | -1,736 | 6,50E-11 | -1,490 |
| 212525_s_at | H2AFX | H2A histone family, member X | other |  |  | 8,96E-10 | -2,353 |  |  |
| 212540_at | CDC34 (includes EG:997) | cell division cycle 34 homolog (S. cerevisiae) | enzyme |  |  | 1,07E-10 | -1,961 |  |  |
| 212544_at | ZNHIT3 | zinc finger, HIT type 3 | transcription regulator |  |  | 2,34E-10 | -1,675 |  |  |
| 212554_at | CAP2 | CAP, adenylate cyclase-associated protein, 2 (yeast) | other | 1,35E-08 | -1,441 |  |  | 1,35E-08 | -1,799 |
| 212563_at | BOP1 | block of proliferation 1 | other |  |  | 3,54E-10 | -2,326 |  |  |
| 212566_at | MAP4 | microtubule-associated protein 4 | other |  |  | 8,49E-08 | -1,741 |  |  |
| 212573_at | ENDOD1 | endonuclease domain containing 1 | enzyme |  |  | 2,32E-10 | -1,635 |  |  |
| 212584_at | AQR | aquarius homolog (mouse) | other |  |  | 2,11E-08 | -1,589 |  |  |
| 212585_at | OSBPL8 | oxysterol binding protein-like 8 | other |  |  | 2,14E-10 | -1,585 |  |  |
| 212586_at | CAST | calpastatin | peptidase | 1,05E-12 | -1,467 | 1,05E-12 | -1,696 | 1,05E-12 | -1,414 |
| 212604_at | MRPS31 | mitochondrial ribosomal protein S31 | other |  |  | 3,33E-08 | -1,654 |  |  |
| 212614_at | ARID5B | AT rich interactive domain 5B (MRF1-like) | transcription regulator |  |  | 1,96E-09 | -1,609 |  |  |
| 212615_at | CHD9 (includes EG:80205) | chromodomain helicase DNA binding protein 9 | other | 2,05E-10 | -1,711 | 2,05E-10 | -1,583 | 2,05E-10 | -1,488 |
| 212616_at | CHD9 (includes EG:80205) | chromodomain helicase DNA binding protein 9 | other |  |  | 3,05E-09 | -1,628 |  |  |
| 212626_x_at | HNRNPC | heterogeneous nuclear ribonucleoprotein C (C1/C2) | other |  |  | 9,54E-08 | -1,519 |  |  |
| 212629_s_at | PKN2 | protein kinase N2 | kinase |  |  |  |  | 1,83E-14 | 1,932 |
| 212633_at | KIAA0776 | KIAA0776 | other | 1,83E-14 | -1,720 | 1,83E-14 | -1,680 | 1,83E-14 | -1,625 |
| 212637_s_at | WWP1 | WW domain containing E3 ubiquitin protein ligase 1 | enzyme | 9,43E-12 | -1,673 | 9,43E-12 | -1,954 |  |  |
| 212640_at | PTPLB | protein tyrosine phosphatase-like (proline instead of catalytic arginine), member b | phosphatase | 5,87E-09 | -1,361 | 5,87E-09 | -1,475 |  |  |
| 212646_at | RFTN1 | raftlin, lipid raft linker 1 | other | 8,91E-09 | -1,604 |  |  |  |  |
| 212648_at | DHX29 | DEAH (Asp-Glu-Ala-His) box polypeptide 29 | enzyme | 1,89E-09 | -1,483 | 1,89E-09 | -1,549 | 1,89E-09 | -1,310 |
| 212660_at | PHF15 | PHD finger protein 15 | other |  |  | 2,09E-08 | -1,743 |  |  |
| 212665_at | TIPARP | TCDD-inducible poly(ADP-ribose) polymerase | other |  |  |  |  | 1,83E-14 | 2,476 |
| 212672_at | ATM | ataxia telangiectasia mutated | kinase |  |  | 4,72E-09 | -1,910 |  |  |
| 212694_s_at | PCCB | propionyl Coenzyme A carboxylase, beta polypeptide | enzyme |  |  | 1,47E-11 | -1,878 |  |  |
| 212696_s_at | RNF4 | ring finger protein 4 | transcription regulator |  |  | 3,46E-11 | -1,907 |  |  |
| 212708_at | MSL1 | male-specific lethal 1 homolog (Drosophila) | other |  |  | 1,14E-10 | -1,837 |  |  |
| 212709_at | NUP160 | nucleoporin 160kDa | transporter |  |  | 4,18E-12 | -1,851 |  |  |
| 212716_s_at | EIF3K | eukaryotic translation initiation factor 3, subunit K | translation regulator |  |  | 6,93E-09 | -1,600 |  |  |
| 212721_at | SFRS12 | splicing factor, arginine/serine-rich 12 | other |  |  | 7,60E-10 | -1,507 |  |  |
| 212725_s_at | TUG1 (includes EG:55000) | taurine upregulated 1 (non-protein coding) | other | 4,23E-09 | -1,356 | 4,23E-09 | -1,480 |  |  |
| 212737_at | GM2A | GM2 ganglioside activator | enzyme |  |  | 7,14E-10 | -1,783 |  |  |
| 212739_s_at | NME4 | non-metastatic cells 4, protein expressed in | kinase |  |  | 2,76E-10 | -1,883 |  |  |
| 212740_at | PIK3R4 | phosphoinositide-3-kinase, regulatory subunit 4 | kinase |  |  | 4,51E-10 | -1,551 |  |  |
| 212757_s_at | CAMK2G | calcium/calmodulin-dependent protein kinase II gamma | kinase |  |  | 1,31E-08 | -1,729 |  |  |
| 212761_at | TCF7L2 (includes EG:6934) | transcription factor 7-like 2 (T-cell specific, HMG-box) | transcription regulator |  |  | 7,67E-08 | -1,645 |  |  |
| 212762_s_at | TCF7L2 (includes EG:6934) | transcription factor 7-like 2 (T-cell specific, HMG-box) | transcription regulator |  |  | 1,92E-10 | -1,848 |  |  |
| 212773_s_at | TOMM20 | translocase of outer mitochondrial membrane 20 homolog (yeast) | transporter |  |  | 8,47E-11 | -1,624 |  |  |
| 212782_x_at | POLR2J | polymerase (RNA) II (DNA directed) polypeptide J, 13.3kDa | enzyme |  |  | 3,85E-08 | -1,614 |  |  |
| 212783_at | RBBP6 (includes EG:5930) | retinoblastoma binding protein 6 | other |  |  | 1,48E-08 | -1,680 |  |  |
| 212785_s_at | LARP7 (includes EG:51574) | La ribonucleoprotein domain family, member 7 | other | 1,01E-10 | -1,632 | 1,01E-10 | -1,775 |  |  |
| 212789_at | NCAPD3 | non-SMC condensin II complex, subunit D3 | other | 1,78E-12 | -1,446 | 1,78E-12 | -1,751 |  |  |
| 212799_at | STX6 | syntaxin 6 | transporter |  |  | 2,63E-09 | -1,732 |  |  |
| 212802_s_at | GAPVD1 | GTPase activating protein and VPS9 domains 1 | other |  |  | 5,19E-09 | -1,646 |  |  |
| 212812_at | SERINC5 | serine incorporator 5 | transporter |  |  | 4,21E-08 | -1,396 | 4,21E-08 | -1,374 |
| 212825_at | PAXIP1 | PAX interacting (with transcription-activation domain) protein 1 | other |  |  | 8,46E-08 | -1,512 |  |  |
| 212828_at | SYNJ2 | synaptojanin 2 | phosphatase |  |  | 2,08E-09 | -2,035 |  |  |
| 212838_at | DNMBP | dynamin binding protein | other | 3,14E-12 | -1,537 | 3,14E-12 | -2,012 | 3,14E-12 | -1,508 |
| 212846_at | RRP1B | ribosomal RNA processing 1 homolog B (S. cerevisiae) | other |  |  | 9,40E-08 | -1,471 |  |  |
| 212859_x_at | MT1E | metallothionein 1E | other | 1,83E-14 | 6,004 |  |  | 1,83E-14 | 3,527 |
| 212862_at | CDS2 | CDP-diacylglycerol synthase (phosphatidate cytidylyltransferase) 2 | enzyme | 1,83E-14 | -1,780 | 1,83E-14 | -2,272 | 1,83E-14 | -1,567 |
| 212863_x_at | CTBP1 | C-terminal binding protein 1 | enzyme |  |  | 1,40E-11 | -1,872 |  |  |
| 212864_at | CDS2 | CDP-diacylglycerol synthase (phosphatidate cytidylyltransferase) 2 | enzyme | 1,81E-10 | -1,717 | 1,81E-10 | -1,936 |  |  |
| 212888_at | DICER1 | dicer 1, ribonuclease type III | enzyme | 2,93E-10 | -1,456 | 2,93E-10 | -1,627 |  |  |
| 212898_at | KIAA0406 | KIAA0406 | other |  |  | 3,74E-11 | -1,849 |  |  |
| 212899_at | CDC2L6 | cell division cycle 2-like 6 (CDK8-like) | kinase | 1,61E-11 | -1,852 | 1,61E-11 | -1,678 | 1,61E-11 | -1,513 |
| 212902_at | SEC24A | SEC24 family, member A (S. cerevisiae) | transporter |  |  | 2,27E-08 | -1,657 |  |  |
| 212904_at | LRRC47 | leucine rich repeat containing 47 | other |  |  | 5,74E-12 | -1,788 | 5,74E-12 | -1,328 |
| 212907_at | SLC30A1 | solute carrier family 30 (zinc transporter), member 1 | transporter |  |  |  |  | 1,83E-14 | 1,992 |
| 212915_at | PDZRN3 | PDZ domain containing ring finger 3 | other | 4,87E-14 | -1,788 | 4,87E-14 | -2,084 | 4,87E-14 | -2,070 |
| 212916_at | PHF8 | PHD finger protein 8 | other | 1,46E-10 | -2,032 | 1,46E-10 | -2,607 | 1,46E-10 | -1,610 |
| 212917_x_at | RECQL | RecQ protein-like (DNA helicase Q1-like) | enzyme | 1,83E-14 | -1,656 | 1,83E-14 | -1,989 | 1,83E-14 | -1,532 |
| 212918_at | RECQL | RecQ protein-like (DNA helicase Q1-like) | enzyme |  |  | 3,57E-09 | -1,630 |  |  |
| 212919_at | DCP2 | DCP2 decapping enzyme homolog (S. cerevisiae) | other | 2,24E-08 | -1,428 |  |  |  |  |
| 212949_at | NCAPH | non-SMC condensin I complex, subunit H | other |  |  | 2,75E-08 | -1,648 |  |  |
| 212953_x_at | CALR | calreticulin | transcription regulator | 8,45E-08 | -1,704 |  |  |  |  |
| 212955_s_at | POLR2I | polymerase (RNA) II (DNA directed) polypeptide I, 14.5kDa | transcription regulator |  |  | 1,06E-08 | -1,598 |  |  |
| 212966_at | HIC2 | hypermethylated in cancer 2 | other |  |  | 1,67E-10 | -1,772 |  |  |
| 212967_x_at | NAP1L1 | nucleosome assembly protein 1-like 1 | other |  |  | 1,24E-09 | -1,471 |  |  |
| 212973_at | RPIA | ribose 5-phosphate isomerase A | enzyme |  |  | 1,23E-11 | -1,652 |  |  |
| 212981_s_at | FAM115A | family with sequence similarity 115, member A | other | 1,83E-14 | -1,844 | 1,83E-14 | -2,010 | 1,83E-14 | -2,245 |
| 212983_at | HRAS | v-Ha-ras Harvey rat sarcoma viral oncogene homolog | enzyme |  |  | 1,42E-10 | -2,133 | 1,42E-10 | -1,483 |
| 212987_at | FBXO9 | F-box protein 9 | enzyme | 3,50E-11 | -1,607 | 3,50E-11 | -1,482 |  |  |
| 212993_at | NACC2 | NACC family member 2, BEN and BTB (POZ) domain containing | other |  |  | 1,22E-08 | -1,647 |  |  |
| 213017_at | ABHD3 | abhydrolase domain containing 3 | enzyme |  |  | 6,58E-11 | -1,757 |  |  |
| 213018_at | GATAD1 | GATA zinc finger domain containing 1 | other |  |  | 2,91E-08 | -1,725 |  |  |
| 213025_at | THUMPD1 | THUMP domain containing 1 | other |  |  | 2,78E-11 | -1,989 |  |  |
| 213026_at | ATG12 | ATG12 autophagy related 12 homolog (S. cerevisiae) | other |  |  | 2,80E-09 | -1,527 |  |  |
| 213039_at | ARHGEF18 | rho/rac guanine nucleotide exchange factor (GEF) 18 | other |  |  | 2,42E-07 | -1,595 |  |  |
| 213041_s_at | ATP5D | ATP synthase, H+ transporting, mitochondrial F1 complex, delta subunit | transporter | 1,83E-14 | -1,544 | 1,83E-14 | -2,519 | 1,83E-14 | -1,500 |
| 213056_at | FRMD4B | FERM domain containing 4B | other |  |  | 1,99E-08 | -1,439 | 1,99E-08 | -1,597 |
| 213057_at | ATPAF2 | ATP synthase mitochondrial F1 complex assembly factor 2 | other |  |  | 5,46E-10 | -2,232 |  |  |
| 213065_at | ZFC3H1 | zinc finger, C3H1-type containing | other |  |  | 8,82E-09 | -1,574 |  |  |
| 213079_at | TSR2 | TSR2, 20S rRNA accumulation, homolog (S. cerevisiae) | other |  |  | 2,99E-09 | -2,014 |  |  |
| 213106_at | ATP8A1 (includes EG:10396) | ATPase, aminophospholipid transporter (APLT), class I, type 8A, member 1 | transporter |  |  |  |  | 1,82E-07 | -1,501 |
| 213123_at | MFAP3 | microfibrillar-associated protein 3 | other |  |  | 3,78E-08 | -1,709 |  |  |
| 213132_s_at | MCAT | malonyl CoA:ACP acyltransferase (mitochondrial) | enzyme |  |  | 5,62E-10 | -2,195 |  |  |
| 213133_s_at | GCSH | glycine cleavage system protein H (aminomethyl carrier) | enzyme |  |  | 9,75E-10 | -1,621 |  |  |
| 213134_x_at | BTG3 | BTG family, member 3 | other |  |  | 4,39E-08 | -1,541 |  |  |
| 213143_at | C2ORF72 | chromosome 2 open reading frame 72 | other | 5,73E-11 | -1,655 | 5,73E-11 | -1,872 | 5,73E-11 | -2,407 |
| 213145_at | FBXL14 | F-box and leucine-rich repeat protein 14 | other |  |  | 8,00E-13 | -2,006 |  |  |
| 213148_at | C2ORF72 | chromosome 2 open reading frame 72 | other | 7,81E-06 | -1,656 |  |  |  |  |
| 213168_at | SP3 | Sp3 transcription factor | transcription regulator |  |  | 5,77E-08 | -1,407 |  |  |
| 213179_at | RQCD1 | RCD1 required for cell differentiation1 homolog (S. pombe) | other |  |  | 1,04E-10 | -1,831 |  |  |
| 213203_at | SNAPC5 | small nuclear RNA activating complex, polypeptide 5, 19kDa | transcription regulator |  |  | 3,85E-08 | -1,581 |  |  |
| 213222_at | PLCB1 | phospholipase C, beta 1 (phosphoinositide-specific) | enzyme |  |  | 3,46E-08 | -1,445 |  |  |
| 213225_at | PPM1B | protein phosphatase 1B (formerly 2C), magnesium-dependent, beta isoform | phosphatase |  |  |  |  | 9,33E-07 | -1,338 |
| 213226_at | CCNA2 | cyclin A2 | other |  |  | 5,09E-08 | -1,452 |  |  |
| 213241_at | PLXNC1 | plexin C1 | other | 1,83E-14 | -2,004 | 1,83E-14 | -1,785 | 1,83E-14 | -2,599 |
| 213246_at | C14ORF109 | chromosome 14 open reading frame 109 | other | 1,34E-12 | -1,601 | 1,34E-12 | -1,860 | 1,34E-12 | -1,486 |
| 213258_at | TFPI | tissue factor pathway inhibitor (lipoprotein-associated coagulation inhibitor) | other | 2,17E-08 | -1,493 |  |  |  |  |
| 213263_s_at | PCBP2 | poly(rC) binding protein 2 | other |  |  | 8,19E-09 | -1,762 |  |  |
| 213264_at | PCBP2 | poly(rC) binding protein 2 | other |  |  | 3,20E-10 | -1,916 | 3,20E-10 | -1,412 |
| 213280_at | GARNL4 | GTPase activating Rap/RanGAP domain-like 4 | other |  |  | 3,29E-11 | -1,990 |  |  |
| 213292_s_at | SNX13 | sorting nexin 13 | transporter |  |  | 6,22E-08 | -1,866 |  |  |
| 213294_at | EIF2AK2 | eukaryotic translation initiation factor 2-alpha kinase 2 | kinase |  |  | 1,70E-07 | -1,523 |  |  |
| 213310_at | EIF2C2 | eukaryotic translation initiation factor 2C, 2 | translation regulator |  |  | 2,88E-09 | -1,779 |  |  |
| 213313_at | RABGAP1 | RAB GTPase activating protein 1 | other |  |  | 8,88E-14 | -2,151 |  |  |
| 213320_at | PRMT3 | protein arginine methyltransferase 3 | enzyme |  |  | 3,66E-10 | -1,676 |  |  |
| 213322_at | C6ORF130 | chromosome 6 open reading frame 130 | other | 1,57E-11 | -1,642 | 1,57E-11 | -1,714 | 1,57E-11 | -1,486 |
| 213330_s_at | STIP1 | stress-induced-phosphoprotein 1 | other | 1,83E-14 | -2,676 | 1,83E-14 | -3,699 | 1,83E-14 | -2,171 |
| 213360_s_at | POM121 | POM121 membrane glycoprotein (rat) | other |  |  | 1,67E-09 | -1,684 |  |  |
| 213361_at | TDRD7 | tudor domain containing 7 | other |  |  | 4,99E-08 | -1,594 |  |  |
| 213366_x_at | ATP5C1 | ATP synthase, H+ transporting, mitochondrial F1 complex, gamma polypeptide 1 | transporter |  |  | 1,78E-09 | -1,518 |  |  |
| 213374_x_at | HIBCH | 3-hydroxyisobutyryl-Coenzyme A hydrolase | enzyme | 7,46E-11 | -1,408 | 7,46E-11 | -1,620 |  |  |
| 213378_s_at | DDX11 | DEAD/H (Asp-Glu-Ala-Asp/His) box polypeptide 11 (CHL1-like helicase homolog, S. cerevisiae) | enzyme |  |  | 2,08E-11 | -2,566 |  |  |
| 213379_at | COQ2 | coenzyme Q2 homolog, prenyltransferase (yeast) | enzyme | 2,06E-13 | -1,536 | 2,06E-13 | -2,060 | 2,06E-13 | -1,364 |
| 213397_x_at | RNASE4 | ribonuclease, RNase A family, 4 | enzyme | 5,93E-12 | -2,382 |  |  | 5,93E-12 | -2,483 |
| 213398_s_at | SDR39U1 | short chain dehydrogenase/reductase family 39U, member 1 | other |  |  | 2,04E-08 | -1,637 | 2,04E-08 | -1,400 |
| 213446_s_at | IQGAP1 | IQ motif containing GTPase activating protein 1 | other | 1,83E-14 | 1,907 |  |  | 1,83E-14 | 2,324 |
| 213465_s_at | PPP1R7 | protein phosphatase 1, regulatory (inhibitor) subunit 7 | phosphatase | 1,07E-09 | -1,503 | 1,07E-09 | -1,681 |  |  |
| 213504_at | COPS6 | COP9 constitutive photomorphogenic homolog subunit 6 (Arabidopsis) | other | 9,14E-12 | -1,400 | 9,14E-12 | -1,938 |  |  |
| 213507_s_at | KPNB1 | karyopherin (importin) beta 1 | transporter |  |  | 9,87E-09 | -1,561 |  |  |
| 213511_s_at | MTMR1 | myotubularin related protein 1 | phosphatase |  |  | 8,28E-12 | -1,648 |  |  |
| 213521_at | PTPN18 | protein tyrosine phosphatase, non-receptor type 18 (brain-derived) | phosphatase | 1,83E-14 | -2,030 | 1,83E-14 | -2,684 | 1,83E-14 | -2,279 |
| 213523_at | CCNE1 | cyclin E1 | transcription regulator | 2,28E-12 | -1,641 | 2,28E-12 | -2,107 |  |  |
| 213524_s_at | G0S2 | G0/G1switch 2 | other | 1,83E-14 | -2,009 | 1,83E-14 | -2,009 | 1,83E-14 | -3,506 |
| 213532_at | ADAM17 | ADAM metallopeptidase domain 17 | peptidase |  |  | 2,96E-12 | -1,921 |  |  |
| 213535_s_at | UBE2I | ubiquitin-conjugating enzyme E2I (UBC9 homolog, yeast) | enzyme |  |  | 1,56E-09 | -1,637 |  |  |
| 213548_s_at | CDV3 | CDV3 homolog (mouse) | other | 7,55E-14 | -1,660 | 7,55E-14 | -2,309 | 7,55E-14 | -2,186 |
| 213571_s_at | EIF4E2 | eukaryotic translation initiation factor 4E family member 2 | translation regulator |  |  | 1,31E-08 | -1,544 |  |  |
| 213572_s_at | SERPINB1 | serpin peptidase inhibitor, clade B (ovalbumin), member 1 | other |  |  |  |  | 9,04E-08 | -1,362 |
| 213587_s_at | ATP6V0E2 | ATPase, H+ transporting V0 subunit e2 | enzyme | 2,40E-10 | -1,368 | 2,40E-10 | -1,787 | 2,40E-10 | -1,515 |
| 213594_x_at | FUSIP1 | FUS interacting protein (serine/arginine-rich) 1 | other |  |  | 3,87E-08 | -1,549 |  |  |
| 213599_at | OIP5 | Opa interacting protein 5 | other | 7,01E-11 | -1,450 | 7,01E-11 | -1,618 | 7,01E-11 | -1,362 |
| 213600_at | SIPA1L3 (includes EG:23094) | signal-induced proliferation-associated 1 like 3 | other | 6,26E-14 | -2,132 | 6,26E-14 | -2,440 | 6,26E-14 | -1,671 |
| 213605_s_at | LOC100134282 | hypothetical protein LOC100134282 | other | 6,03E-09 | -1,784 | 6,03E-09 | -1,617 |  |  |
| 213617_s_at | C18ORF10 | chromosome 18 open reading frame 10 | other | 2,92E-11 | -1,402 | 2,92E-11 | -1,961 |  |  |
| 213623_at | KIF3A | kinesin family member 3A | other |  |  |  |  | 4,28E-12 | 1,971 |
| 213624_at | SMPDL3A | sphingomyelin phosphodiesterase, acid-like 3A | enzyme | 2,32E-09 | -1,602 | 2,32E-09 | -1,641 | 2,32E-09 | -1,384 |
| 213629_x_at | MT1F | metallothionein 1F | other | 1,83E-14 | 3,725 |  |  | 1,83E-14 | 2,410 |
| 213647_at | DNA2 | DNA replication helicase 2 homolog (yeast) | other |  |  | 1,23E-11 | -1,800 |  |  |
| 213687_s_at | RPL35A | ribosomal protein L35a | other |  |  | 2,09E-07 | -1,485 |  |  |
| 213735_s_at | COX5B | cytochrome c oxidase subunit Vb | enzyme |  |  |  |  | 7,93E-08 | -1,374 |
| 213746_s_at | FLNA | filamin A, alpha (actin binding protein 280) | other |  |  | 4,40E-12 | -1,996 |  |  |
| 213754_s_at | PAIP1 | poly(A) binding protein interacting protein 1 | translation regulator |  |  | 2,86E-09 | -1,611 | 2,86E-09 | -1,321 |
| 213775_x_at | ZNF638 | zinc finger protein 638 | other | 1,62E-12 | -1,584 | 1,62E-12 | -1,763 | 1,62E-12 | -1,363 |
| 213787_s_at | EBP | emopamil binding protein (sterol isomerase) | enzyme |  |  | 6,14E-11 | -1,864 |  |  |
| 213846_at | COX7C (includes EG:1350) | cytochrome c oxidase subunit VIIc | enzyme |  |  | 6,94E-08 | -1,642 |  |  |
| 213853_at | DNAJC24 | DnaJ (Hsp40) homolog, subfamily C, member 24 | other |  |  |  |  | 3,07E-08 | 1,419 |
| 213857_s_at | CD47 | CD47 molecule | other | 2,97E-09 | -1,647 | 2,97E-09 | -1,749 |  |  |
| 213864_s_at | NAP1L1 | nucleosome assembly protein 1-like 1 | other |  |  | 3,99E-09 | -1,474 |  |  |
| 213887_s_at | POLR2E | polymerase (RNA) II (DNA directed) polypeptide E, 25kDa | enzyme |  |  | 3,81E-11 | -1,782 |  |  |
| 213891_s_at | TCF4 | transcription factor 4 | transcription regulator | 1,83E-14 | -2,242 | 1,83E-14 | -2,419 | 1,83E-14 | -2,867 |
| 213892_s_at | APRT | adenine phosphoribosyltransferase | enzyme |  |  | 3,91E-08 | -1,608 |  |  |
| 213893_x_at | PMS2L5 | postmeiotic segregation increased 2-like 5 | other |  |  | 5,45E-09 | -1,969 |  |  |
| 213923_at | RAP2B | RAP2B, member of RAS oncogene family | enzyme |  |  | 3,04E-08 | -1,620 |  |  |
| 213944_x_at | GNA11 | guanine nucleotide binding protein (G protein), alpha 11 (Gq class) | enzyme |  |  | 1,77E-08 | -1,580 | 1,77E-08 | -1,580 |
| 213947_s_at | NUP210 | nucleoporin 210kDa | transporter |  |  | 5,72E-10 | -2,213 |  |  |
| 213953_at | KRT20 | keratin 20 | other | 1,83E-14 | 2,054 |  |  | 1,83E-14 | 2,119 |
| 213980_s_at | CTBP1 | C-terminal binding protein 1 | enzyme | 1,74E-13 | -1,539 | 1,74E-13 | -2,070 | 1,74E-13 | -1,357 |
| 213989_x_at | SETD4 | SET domain containing 4 | other |  |  | 2,96E-09 | -2,050 |  |  |
| 213995_at | ATP5S | ATP synthase, H+ transporting, mitochondrial F0 complex, subunit s (factor B) | transporter |  |  | 1,79E-09 | -1,747 | 1,79E-09 | -1,521 |
| 214004_s_at | VGLL4 | vestigial like 4 (Drosophila) | other | 2,92E-08 | -1,502 | 2,92E-08 | -1,841 |  |  |
| 214007_s_at | TWF1 | twinfilin, actin-binding protein, homolog 1 (Drosophila) | kinase |  |  |  |  | 5,08E-10 | -1,578 |
| 214008_at | TWF1 | twinfilin, actin-binding protein, homolog 1 (Drosophila) | kinase |  |  | 1,81E-07 | 2,175 |  |  |
| 214033_at | ABCC6 | ATP-binding cassette, sub-family C (CFTR/MRP), member 6 | transporter |  |  | 2,79E-11 | -1,755 | 2,79E-11 | -1,676 |
| 214039_s_at | LAPTM4B | lysosomal protein transmembrane 4 beta | other |  |  | 1,16E-08 | -1,431 |  |  |
| 214042_s_at | RPL22 | ribosomal protein L22 | other | 2,75E-12 | -1,606 | 2,75E-12 | -1,662 | 2,75E-12 | -1,378 |
| 214048_at | MBD4 | methyl-CpG binding domain protein 4 | enzyme |  |  |  |  | 3,63E-08 | -1,444 |
| 214051_at | TMSL8 | thymosin-like 8 | other |  |  | 2,41E-10 | -2,027 | 2,41E-10 | -1,449 |
| 214075_at | NENF | neuron derived neurotrophic factor | growth factor |  |  | 2,86E-09 | -1,805 | 2,86E-09 | -1,579 |
| 214079_at | DHRS2 (includes EG:10202) | dehydrogenase/reductase (SDR family) member 2 | enzyme | 1,83E-14 | -1,804 | 1,83E-14 | -1,853 | 1,83E-14 | -2,131 |
| 214080_x_at | PRKCSH | protein kinase C substrate 80K-H | enzyme |  |  | 8,58E-10 | -1,970 |  |  |
| 214091_s_at | GPX3 | glutathione peroxidase 3 (plasma) | enzyme | 4,96E-12 | -1,460 | 4,96E-12 | -1,745 |  |  |
| 214093_s_at | FUBP1 | far upstream element (FUSE) binding protein 1 | transcription regulator | 1,20E-11 | -1,736 | 1,20E-11 | -1,531 | 1,20E-11 | -1,586 |
| 214104_at | GPR161 | G protein-coupled receptor 161 | G-protein coupled receptor | 1,11E-06 | -1,490 |  |  |  |  |
| 214239_x_at | PCGF2 | polycomb group ring finger 2 | transcription regulator |  |  | 9,46E-09 | -1,803 |  |  |
| 214240_at | GAL | galanin prepropeptide | other |  |  | 4,87E-14 | -1,990 |  |  |
| 214247_s_at | DKK3 | dickkopf homolog 3 (Xenopus laevis) | other | 8,18E-10 | -1,719 | 8,18E-10 | -1,594 | 8,18E-10 | -1,412 |
| 214264_s_at | C14ORF143 | chromosome 14 open reading frame 143 | other | 2,84E-09 | -1,420 | 2,84E-09 | -1,502 | 2,84E-09 | -1,445 |
| 214273_x_at | C16ORF35 | chromosome 16 open reading frame 35 | other | 1,74E-10 | -1,620 | 1,74E-10 | -2,233 | 1,74E-10 | -1,739 |
| 214283_at | TMEM97 | transmembrane protein 97 | other | 1,07E-09 | -1,352 | 1,07E-09 | -1,604 | 1,07E-09 | -1,493 |
| 214308_s_at | HGD | homogentisate 1,2-dioxygenase (homogentisate oxidase) | enzyme |  |  | 6,26E-09 | -1,897 | 6,26E-09 | -1,459 |
| 214315_x_at | CALR | calreticulin | transcription regulator | 1,83E-14 | -1,848 | 1,83E-14 | -1,628 | 1,83E-14 | -1,656 |
| 214336_s_at | COPA | coatomer protein complex, subunit alpha | transporter |  |  |  |  | 1,51E-08 | -2,099 |
| 214394_x_at | EEF1D | eukaryotic translation elongation factor 1 delta (guanine nucleotide exchange protein) | translation regulator |  |  | 1,26E-07 | -1,596 |  |  |
| 214430_at | GLA | galactosidase, alpha | enzyme |  |  | 5,36E-09 | -1,511 |  |  |
| 214439_x_at | BIN1 | bridging integrator 1 | other |  |  | 8,24E-09 | -1,879 |  |  |
| 214440_at | NAT1 | N-acetyltransferase 1 (arylamine N-acetyltransferase) | enzyme | 2,44E-08 | -1,374 | 2,44E-08 | -1,532 |  |  |
| 214459_x_at | HLA-C | major histocompatibility complex, class I, C | transmembrane receptor |  |  | 1,69E-07 | -1,587 |  |  |
| 214464_at | CDC42BPA | CDC42 binding protein kinase alpha (DMPK-like) | kinase |  |  |  |  | 8,66E-12 | 1,921 |
| 214484_s_at | OPRS1 | sigma non-opioid intracellular receptor 1 | G-protein coupled receptor |  |  | 9,46E-12 | -2,268 |  |  |
| 214526_x_at | PMS2L1 | postmeiotic segregation increased 2-like 1 pseudogene | other | 3,46E-11 | -1,744 | 3,46E-11 | -2,128 | 3,46E-11 | -1,780 |
| 214527_s_at | PQBP1 | polyglutamine binding protein 1 | transcription regulator |  |  | 2,53E-10 | -1,698 |  |  |
| 214531_s_at | SNX1 | sorting nexin 1 | transporter |  |  | 8,72E-11 | -1,867 |  |  |
| 214544_s_at | SNAP23 | synaptosomal-associated protein, 23kDa | transporter | 3,12E-09 | -1,463 | 3,12E-09 | -1,504 | 3,12E-09 | -1,599 |
| 214545_s_at | PROSC | proline synthetase co-transcribed homolog (bacterial) | enzyme |  |  | 7,55E-14 | -2,462 |  |  |
| 214581_x_at | TNFRSF21 | tumor necrosis factor receptor superfamily, member 21 | other |  |  | 1,83E-14 | -1,618 |  |  |
| 214626_s_at | GANAB | glucosidase, alpha; neutral AB | enzyme | 1,83E-14 | 3,159 | 1,83E-14 | 2,390 | 1,83E-14 | 3,282 |
| 214639_s_at | HOXA1 | homeobox A1 | transcription regulator |  |  | 8,46E-09 | -1,676 | 8,46E-09 | -1,672 |
| 214672_at | TTLL5 | tubulin tyrosine ligase-like family, member 5 | enzyme |  |  | 1,92E-07 | -1,819 |  |  |
| 214686_at | ZNF266 | zinc finger protein 266 | other |  |  | 1,87E-08 | -1,675 |  |  |
| 214710_s_at | CCNB1 | cyclin B1 | other | 3,74E-11 | -1,383 | 3,74E-11 | -1,534 | 3,74E-11 | -1,357 |
| 214736_s_at | ADD1 | adducin 1 (alpha) | other | 1,83E-14 | -1,825 | 1,83E-14 | -2,070 |  |  |
| 214737_x_at | HNRNPC | heterogeneous nuclear ribonucleoprotein C (C1/C2) | other |  |  | 1,38E-07 | -1,493 |  |  |
| 214749_s_at | ARMCX6 | armadillo repeat containing, X-linked 6 | other |  |  | 1,76E-09 | -1,680 |  |  |
| 214752_x_at | FLNA | filamin A, alpha (actin binding protein 280) | other |  |  | 9,78E-10 | -2,029 |  |  |
| 214756_x_at | PMS2L1 | postmeiotic segregation increased 2-like 1 pseudogene | other |  |  | 1,09E-12 | -2,301 | 1,09E-12 | -1,824 |
| 214784_x_at | XPO6 | exportin 6 | transporter | 1,97E-10 | -1,633 | 1,97E-10 | -1,950 | 1,97E-10 | -1,546 |
| 214789_x_at | SFRS2B | splicing factor, arginine/serine-rich 2B | other |  |  | 4,47E-08 | -1,509 |  |  |
| 214794_at | PA2G4 | proliferation-associated 2G4, 38kDa | transcription regulator |  |  | 2,05E-10 | -1,885 |  |  |
| 214835_s_at | SUCLG2 | succinate-CoA ligase, GDP-forming, beta subunit | enzyme | 3,07E-11 | -1,395 | 3,07E-11 | -1,647 | 3,07E-11 | -1,390 |
| 214853_s_at | SHC1 | SHC (Src homology 2 domain containing) transforming protein 1 | other |  |  | 1,23E-08 | -1,644 |  |  |
| 214909_s_at | DDAH2 | dimethylarginine dimethylaminohydrolase 2 | enzyme | 7,13E-11 | -1,750 | 7,13E-11 | -2,297 | 7,13E-11 | -1,732 |
| 214911_s_at | BRD2 | bromodomain containing 2 | kinase |  |  |  |  | 2,45E-10 | 1,471 |
| 215000_s_at | FEZ2 | fasciculation and elongation protein zeta 2 (zygin II) | other |  |  | 5,76E-12 | -1,792 |  |  |
| 215001_s_at | GLUL | glutamate-ammonia ligase (glutamine synthetase) | enzyme | 2,02E-10 | -1,385 | 2,02E-10 | -1,512 | 2,02E-10 | -1,360 |
| 215016_x_at | DST | dystonin | other |  |  | 1,12E-07 | -1,553 |  |  |
| 215034_s_at | TM4SF1 | transmembrane 4 L six family member 1 | other |  |  |  |  | 4,59E-13 | 1,373 |
| 215037_s_at | BCL2L1 | BCL2-like 1 | other |  |  | 2,02E-08 | -2,157 |  |  |
| 215073_s_at | NR2F2 | nuclear receptor subfamily 2, group F, member 2 | ligand-dependent nuclear receptor | 1,83E-14 | -1,636 | 1,83E-14 | -2,113 | 1,83E-14 | -1,849 |
| 215088_s_at | SDHC | succinate dehydrogenase complex, subunit C, integral membrane protein, 15kDa | enzyme | 1,14E-13 | -1,581 | 1,14E-13 | -2,016 | 1,14E-13 | -1,421 |
| 215089_s_at | RBM10 | RNA binding motif protein 10 | other |  |  | 4,98E-10 | -2,099 |  |  |
| 215091_s_at | GTF3A | general transcription factor IIIA | transcription regulator |  |  | 1,98E-10 | -1,646 |  |  |
| 215113_s_at | SENP3 | SUMO1/sentrin/SMT3 specific peptidase 3 | peptidase |  |  | 6,69E-08 | -2,223 |  |  |
| 215136_s_at | EXOSC8 | exosome component 8 | enzyme |  |  | 1,67E-10 | -1,624 |  |  |
| 215210_s_at | DLST | dihydrolipoamide S-succinyltransferase (E2 component of 2-oxo-glutarate complex) | enzyme | 1,83E-14 | 2,485 | 1,83E-14 | 2,839 | 1,83E-14 | 2,069 |
| 215236_s_at | PICALM | phosphatidylinositol binding clathrin assembly protein | other |  |  | 2,42E-11 | -1,794 | 2,42E-11 | -1,503 |
| 215299_x_at | SULT1A1 | sulfotransferase family, cytosolic, 1A, phenol-preferring, member 1 | enzyme | 7,39E-12 | -1,663 | 7,39E-12 | -2,139 | 7,39E-12 | -1,806 |
| 215333_x_at | GSTM1 | glutathione S-transferase mu 1 | enzyme |  |  | 6,47E-10 | -1,874 | 6,47E-10 | -1,810 |
| 215380_s_at | GGCT | gamma-glutamyl cyclotransferase | other |  |  | 2,45E-08 | -1,470 |  |  |
| 215399_s_at | OS9 | amplified in osteosarcoma | other | 7,05E-12 | -1,494 | 7,05E-12 | -1,941 |  |  |
| 215416_s_at | STOML2 | stomatin (EPB72)-like 2 | other |  |  | 1,08E-08 | -1,639 |  |  |
| 215424_s_at | SNW1 | SNW domain containing 1 | transcription regulator | 5,50E-12 | -1,491 | 5,50E-12 | -1,779 |  |  |
| 215425_at | BTG3 | BTG family, member 3 | other |  |  | 2,41E-10 | -1,963 |  |  |
| 215464_s_at | TAX1BP3 | Tax1 (human T-cell leukemia virus type I) binding protein 3 | transcription regulator |  |  |  |  | 3,31E-06 | -1,402 |
| 215535_s_at | AGPAT1 | 1-acylglycerol-3-phosphate O-acyltransferase 1 (lysophosphatidic acid acyltransferase, alpha) | enzyme |  |  | 9,66E-09 | -1,896 |  |  |
| 215606_s_at | ERC1 | ELKS/RAB6-interacting/CAST family member 1 | other |  |  | 7,16E-08 | -1,990 |  |  |
| 215631_s_at | BRMS1 | breast cancer metastasis suppressor 1 | other |  |  | 1,16E-09 | -1,907 |  |  |
| 215691_x_at | HSPB11 | heat shock protein family B (small), member 11 | other | 2,51E-10 | -1,348 | 2,51E-10 | -1,664 | 2,51E-10 | -1,424 |
| 215695_s_at | GYG2 | glycogenin 2 | enzyme | 6,13E-10 | -1,487 | 6,13E-10 | -2,021 | 6,13E-10 | -1,642 |
| 215696_s_at | SEC16A | SEC16 homolog A (S. cerevisiae) | phosphatase |  |  | 2,05E-09 | -1,619 | 2,05E-09 | -1,310 |
| 215714_s_at | SMARCA4 | SWI/SNF related, matrix associated, actin dependent regulator of chromatin, subfamily a, member 4 | transcription regulator |  |  | 1,84E-09 | -1,830 |  |  |
| 215718_s_at | PHF3 | PHD finger protein 3 | other | 1,63E-13 | -1,852 | 1,63E-13 | -2,208 | 1,63E-13 | -1,780 |
| 215726_s_at | CYB5A | cytochrome b5 type A (microsomal) | enzyme | 1,83E-14 | -1,449 | 1,83E-14 | -1,812 | 1,83E-14 | -1,612 |
| 215729_s_at | VGLL1 | vestigial like 1 (Drosophila) | transcription regulator |  |  | 1,50E-07 | -1,970 |  |  |
| 215772_x_at | SUCLG2 | succinate-CoA ligase, GDP-forming, beta subunit | enzyme |  |  | 3,01E-09 | -1,585 |  |  |
| 215773_x_at | PARP2 | poly (ADP-ribose) polymerase 2 | enzyme |  |  | 7,13E-11 | -1,927 |  |  |
| 215794_x_at | GLUD2 | glutamate dehydrogenase 2 | enzyme | 7,90E-10 | -1,357 | 7,90E-10 | -1,587 | 7,90E-10 | -1,635 |
| 215933_s_at | HHEX | hematopoietically expressed homeobox | transcription regulator | 7,99E-11 | -1,507 | 7,99E-11 | -1,894 | 7,99E-11 | -1,956 |
| 215947_s_at | FAM136A | family with sequence similarity 136, member A | other |  |  | 4,59E-08 | -1,511 |  |  |
| 216032_s_at | ERGIC3 | ERGIC and golgi 3 | other | 1,83E-14 | -1,431 | 1,83E-14 | -2,060 | 1,83E-14 | -1,459 |
| 216035_x_at | TCF7L2 (includes EG:6934) | transcription factor 7-like 2 (T-cell specific, HMG-box) | transcription regulator |  |  | 2,51E-08 | -1,584 |  |  |
| 216048_s_at | RHOBTB3 | Rho-related BTB domain containing 3 | enzyme |  |  | 2,83E-12 | -1,935 |  |  |
| 216064_s_at | AGA | aspartylglucosaminidase | enzyme | 1,36E-09 | -1,395 | 1,36E-09 | -1,870 |  |  |
| 216194_s_at | TBCB | tubulin folding cofactor B | other |  |  | 3,44E-10 | -1,834 |  |  |
| 216202_s_at | SPTLC2 | serine palmitoyltransferase, long chain base subunit 2 | enzyme |  |  | 2,14E-09 | -1,863 |  |  |
| 216215_s_at | RBM9 | RNA binding motif protein 9 | transcription regulator | 1,83E-14 | -1,922 | 1,83E-14 | -2,222 | 1,83E-14 | -1,948 |
| 216222_s_at | MYO10 | myosin X | other |  |  |  |  | 1,83E-08 |  |
| 216232_s_at | GCN1L1 | GCN1 general control of amino-acid synthesis 1-like 1 (yeast) | translation regulator |  |  | 1,24E-08 | -1,803 |  |  |
| 216237_s_at | MCM5 | minichromosome maintenance complex component 5 | enzyme |  |  | 3,85E-08 | -1,615 |  |  |
| 216238_s_at | FGB | fibrinogen beta chain | other | 5,58E-13 | -1,915 | 5,58E-13 | -1,748 | 5,58E-13 | -3,174 |
| 216251_s_at | TTLL12 | tubulin tyrosine ligase-like family, member 12 | other |  |  | 4,41E-08 | -1,797 |  |  |
| 216295_s_at | CLTA | clathrin, light chain (Lca) | other |  |  | 2,02E-08 | -1,524 |  |  |
| 216309_x_at | JRK | jerky homolog (mouse) | other |  |  | 4,35E-08 | -2,454 |  |  |
| 216326_s_at | HDAC3 | histone deacetylase 3 | transcription regulator |  |  | 8,01E-09 | -1,731 |  |  |
| 216336_x_at | MT1M | metallothionein 1M | other | 1,83E-14 | 2,799 |  |  | 1,83E-14 | 2,318 |
| 216338_s_at | YIPF3 | Yip1 domain family, member 3 | other | 1,83E-14 | -2,378 | 1,83E-14 | -2,782 | 1,83E-14 | -2,145 |
| 216348_at | RPS17L4 | ribosomal protein S17-like 4 | other |  |  | 1,58E-08 | -1,688 |  |  |
| 216380_x_at | LOC728453 | similar to 40S ribosomal protein S28 | other | 3,64E-13 | -1,367 | 3,64E-13 | -1,928 | 3,64E-13 | -1,712 |
| 216396_s_at | EI24 | etoposide induced 2.4 mRNA | other |  |  | 1,04E-09 | -1,541 |  |  |
| 216438_s_at | TMSB4X | thymosin beta 4, X-linked | other | 1,24E-10 | -1,530 | 1,24E-10 | -1,466 | 1,24E-10 | -1,364 |
| 216450_x_at | HSP90B1 | heat shock protein 90kDa beta (Grp94), member 1 | other |  |  | 1,57E-10 | 2,107 |  |  |
| 216457_s_at | SF3A1 | splicing factor 3a, subunit 1, 120kDa | other |  |  | 1,28E-07 | -1,583 |  |  |
| 216470_x_at | PRSS1 (includes EG:5644) | protease, serine, 1 (trypsin 1) | peptidase |  |  | 1,44E-10 | -2,223 |  |  |
| 216483_s_at | C19ORF10 | chromosome 19 open reading frame 10 | cytokine | 1,83E-14 | -1,445 | 1,83E-14 | -1,962 | 1,83E-14 | -1,409 |
| 216490_x_at | LOC442175 | similar to hCG1811681 | other | 1,83E-14 | -2,540 | 1,83E-14 | -3,256 | 1,83E-14 | -2,847 |
| 216511_s_at | TCF7L2 (includes EG:6934) | transcription factor 7-like 2 (T-cell specific, HMG-box) | transcription regulator |  |  | 1,68E-08 | -1,659 |  |  |
| 216526_x_at | HLA-B | major histocompatibility complex, class I, B | transmembrane receptor | 1,83E-14 | -1,349 | 1,83E-14 | -1,981 | 1,83E-14 | -1,515 |
| 216563_at | ANKRD12 | ankyrin repeat domain 12 | other |  |  |  |  | 3,86E-11 | 1,975 |
| 216574_s_at | RPE | ribulose-5-phosphate-3-epimerase | enzyme |  |  | 3,12E-08 | -1,621 |  |  |
| 216591_s_at | SDHC | succinate dehydrogenase complex, subunit C, integral membrane protein, 15kDa | enzyme | 1,83E-14 | -3,390 | 1,83E-14 | -5,238 | 1,83E-14 | -3,389 |
| 216602_s_at | FARSA | phenylalanyl-tRNA synthetase, alpha subunit | enzyme |  |  | 2,35E-08 | -2,124 |  |  |
| 216609_at | TXN | thioredoxin | enzyme |  |  | 1,76E-09 | 1,875 |  |  |
| 216640_s_at | PDIA6 | protein disulfide isomerase family A, member 6 | enzyme |  |  | 2,42E-09 | -1,512 |  |  |
| 216641_s_at | LAD1 | ladinin 1 | other |  |  | 1,08E-08 | -3,134 |  |  |
| 216693_x_at | HDGFRP3 | hepatoma-derived growth factor, related protein 3 | other |  |  | 3,60E-08 | -1,695 |  |  |
| 216836_s_at | ERBB2 | v-erb-b2 erythroblastic leukemia viral oncogene homolog 2, neuro/glioblastoma derived oncogene homolog (avian) | kinase |  |  | 7,26E-09 | -2,175 |  |  |
| 216862_s_at | MTCP1 | mature T-cell proliferation 1 | other |  |  | 2,15E-08 | -1,787 |  |  |
| 216899_s_at | SKAP2 | src kinase associated phosphoprotein 2 | other | 1,06E-08 | -1,571 | 1,06E-08 | -1,663 | 1,06E-08 | -1,466 |
| 216903_s_at | CBARA1 | calcium binding atopy-related autoantigen 1 | other |  |  | 3,87E-08 | -1,774 |  |  |
| 216905_s_at | ST14 | suppression of tumorigenicity 14 (colon carcinoma) | peptidase |  |  | 2,25E-11 | -1,905 |  |  |
| 216973_s_at | HOXB7 | homeobox B7 | transcription regulator |  |  | 1,52E-10 | -1,786 |  |  |
| 216977_x_at | SNRPA1 | small nuclear ribonucleoprotein polypeptide A' | other |  |  | 2,36E-13 | -1,827 |  |  |
| 217047_s_at | FAM13A1 | family with sequence similarity 13, member A1 | other |  |  | 1,12E-08 | -2,008 | 1,12E-08 | -1,764 |
| 217073_x_at | APOA1 | apolipoprotein A-I | transporter |  |  | 3,53E-08 | -1,723 |  |  |
| 217122_s_at | SLC35E2 | solute carrier family 35, member E2 | other |  |  | 7,13E-08 | -1,594 |  |  |
| 217144_at | UBC | ubiquitin C | other | 1,59E-09 | -1,399 | 1,59E-09 | -1,638 | 1,59E-09 | -1,737 |
| 217165_x_at | MT1F | metallothionein 1F | other | 1,83E-14 | 6,113 |  |  | 1,83E-14 | 3,770 |
| 217168_s_at | HERPUD1 | homocysteine-inducible, endoplasmic reticulum stress-inducible, ubiquitin-like domain member 1 | other |  |  | 1,09E-07 | -1,540 |  |  |
| 217202_s_at | GLUL | glutamate-ammonia ligase (glutamine synthetase) | enzyme | 4,99E-10 | -1,404 | 4,99E-10 | -1,790 | 4,99E-10 | -1,650 |
| 217289_s_at | SLC37A4 | solute carrier family 37 (glucose-6-phosphate transporter), member 4 | transporter | 1,83E-14 | -1,461 | 1,83E-14 | -2,665 | 1,83E-14 | -1,699 |
| 217294_s_at | ENO1 | enolase 1, (alpha) | transcription regulator |  |  | 2,19E-09 | -1,602 |  |  |
| 217313_at | HECW1 | HECT, C2 and WW domain containing E3 ubiquitin protein ligase 1 | enzyme |  |  | 3,82E-12 | -2,635 |  |  |
| 217336_at | RPS10 | ribosomal protein S10 | other |  |  | 1,01E-13 | -2,538 | 1,01E-13 | -2,446 |
| 217346_at | PPIA (includes EG:5478) | peptidylprolyl isomerase A (cyclophilin A) | enzyme | 7,55E-14 | -1,431 | 7,55E-14 | -1,798 | 7,55E-14 | -2,309 |
| 217379_at | RPL10 | ribosomal protein L10 | other |  |  |  |  | 9,17E-08 | -1,686 |
| 217408_at | MRPS18B | mitochondrial ribosomal protein S18B | other | 3,44E-14 | -1,430 | 3,44E-14 | -2,065 | 3,44E-14 | -1,563 |
| 217485_x_at | PMS2L1 | postmeiotic segregation increased 2-like 1 pseudogene | other | 4,08E-10 | -1,645 | 4,08E-10 | -2,011 | 4,08E-10 | -1,782 |
| 217496_s_at | IDE | insulin-degrading enzyme | peptidase |  |  | 4,95E-08 | -1,614 |  |  |
| 217501_at | CIAO1 | cytosolic iron-sulfur protein assembly 1 homolog (S. cerevisiae) | transcription regulator | 9,42E-11 | -1,485 | 9,42E-11 | -1,919 |  |  |
| 217526_at | NFATC2IP | nuclear factor of activated T-cells, cytoplasmic, calcineurin-dependent 2 interacting protein | other | 3,19E-11 | -1,346 | 3,19E-11 | -1,737 | 3,19E-11 | -1,565 |
| 217527_s_at | NFATC2IP | nuclear factor of activated T-cells, cytoplasmic, calcineurin-dependent 2 interacting protein | other | 2,79E-09 | -1,412 | 2,79E-09 | -1,649 | 2,79E-09 | -1,481 |
| 217551_at | LOC441453 | similar to olfactory receptor, family 7, subfamily A, member 17 | other |  |  | 3,40E-09 | -2,417 | 3,40E-09 | -2,148 |
| 217640_x_at | C18ORF24 | chromosome 18 open reading frame 24 | other | 1,83E-14 | -1,801 | 1,83E-14 | -2,042 | 1,83E-14 | -1,701 |
| 217678_at | SLC7A11 | solute carrier family 7, (cationic amino acid transporter, y+ system) member 11 | transporter |  |  |  |  | 1,83E-14 | 1,633 |
| 217716_s_at | SEC61A1 | Sec61 alpha 1 subunit (S. cerevisiae) | transporter |  |  | 3,44E-14 | -2,044 |  |  |
| 217720_at | CHCHD2 | coiled-coil-helix-coiled-coil-helix domain containing 2 | other |  |  | 1,41E-07 | -1,475 |  |  |
| 217722_s_at | NGRN | neugrin, neurite outgrowth associated | other |  |  | 6,13E-11 | -1,587 | 6,13E-11 | -1,286 |
| 217724_at | SERBP1 | SERPINE1 mRNA binding protein 1 | other |  |  | 3,72E-09 | -1,523 |  |  |
| 217725_x_at | SERBP1 | SERPINE1 mRNA binding protein 1 | other |  |  | 3,69E-10 | -1,650 |  |  |
| 217726_at | COPZ1 | coatomer protein complex, subunit zeta 1 | transporter | 1,08E-12 | -1,432 | 1,08E-12 | -1,765 | 1,08E-12 | -1,318 |
| 217728_at | S100A6 | S100 calcium binding protein A6 | transporter |  |  | 5,00E-13 | -1,978 |  |  |
| 217730_at | TMBIM1 | transmembrane BAX inhibitor motif containing 1 | other | 2,04E-12 | -1,425 | 2,04E-12 | -1,906 | 2,04E-12 | -1,593 |
| 217732_s_at | ITM2B | integral membrane protein 2B | other |  |  | 1,28E-09 | -1,527 | 1,28E-09 | -1,336 |
| 217736_s_at | EIF2AK1 | eukaryotic translation initiation factor 2-alpha kinase 1 | kinase |  |  | 3,02E-09 | -1,608 |  |  |
| 217738_at | NAMPT | nicotinamide phosphoribosyltransferase | cytokine |  |  | 1,50E-07 | -1,569 |  |  |
| 217742_s_at | WAC | WW domain containing adaptor with coiled-coil | other |  |  | 2,10E-11 | -1,661 |  |  |
| 217743_s_at | TMEM30A | transmembrane protein 30A | other | 1,40E-08 | -1,331 | 1,40E-08 | -1,464 |  |  |
| 217744_s_at | PERP | PERP, TP53 apoptosis effector | other |  |  | 4,65E-10 | -1,576 |  |  |
| 217748_at | ADIPOR1 | adiponectin receptor 1 | transmembrane receptor |  |  | 1,31E-07 | -1,688 |  |  |
| 217761_at | ADI1 | acireductone dioxygenase 1 | enzyme | 3,93E-13 | -1,680 | 3,93E-13 | -1,477 | 3,93E-13 | -1,351 |
| 217768_at | C14ORF166 | chromosome 14 open reading frame 166 | other | 2,06E-08 | -1,390 |  |  | 2,06E-08 | -1,344 |
| 217771_at | GOLM1 | golgi membrane protein 1 | other |  |  | 3,21E-08 | -1,638 |  |  |
| 217778_at | SLC39A1 | solute carrier family 39 (zinc transporter), member 1 | transporter |  |  | 1,14E-10 | -1,994 | 1,14E-10 | -1,592 |
| 217782_s_at | GPS1 (includes EG:2873) | G protein pathway suppressor 1 | other |  |  | 2,49E-09 | -2,013 |  |  |
| 217783_s_at | YPEL5 | yippee-like 5 (Drosophila) | other |  |  | 1,60E-09 | -1,724 |  |  |
| 217786_at | PRMT5 | protein arginine methyltransferase 5 | enzyme |  |  | 5,10E-12 | -2,009 |  |  |
| 217787_s_at | GALNT2 | UDP-N-acetyl-alpha-D-galactosamine:polypeptide N-acetylgalactosaminyltransferase 2 (GalNAc-T2) | enzyme |  |  | 1,80E-12 | -2,519 |  |  |
| 217788_s_at | GALNT2 | UDP-N-acetyl-alpha-D-galactosamine:polypeptide N-acetylgalactosaminyltransferase 2 (GalNAc-T2) | enzyme |  |  | 7,49E-09 | -1,746 |  |  |
| 217795_s_at | TMEM43 | transmembrane protein 43 | other |  |  | 4,27E-10 | -1,894 |  |  |
| 217798_at | CNOT2 | CCR4-NOT transcription complex, subunit 2 | transcription regulator |  |  | 2,41E-08 | -1,620 |  |  |
| 217800_s_at | NDFIP1 | Nedd4 family interacting protein 1 | other |  |  | 1,74E-13 | -1,907 |  |  |
| 217806_s_at | POLDIP2 | polymerase (DNA-directed), delta interacting protein 2 | other |  |  | 8,30E-09 | -1,625 |  |  |
| 217816_s_at | PCNP | PEST proteolytic signal containing nuclear protein | other | 3,44E-14 | -1,547 | 3,44E-14 | -1,768 |  |  |
| 217821_s_at | WBP11 | WW domain binding protein 11 | phosphatase |  |  | 5,54E-11 | -1,800 | 5,54E-11 | -1,329 |
| 217822_at | WBP11 | WW domain binding protein 11 | phosphatase | 2,78E-10 | -1,369 | 2,78E-10 | -1,581 | 2,78E-10 | -1,309 |
| 217823_s_at | UBE2J1 | ubiquitin-conjugating enzyme E2, J1 (UBC6 homolog, yeast) | enzyme | 2,12E-11 | -1,647 | 2,12E-11 | -1,751 | 2,12E-11 | -1,485 |
| 217829_s_at | USP39 | ubiquitin specific peptidase 39 | peptidase | 1,74E-13 | -1,675 | 1,74E-13 | -1,957 | 1,74E-13 | -1,361 |
| 217831_s_at | NSFL1C | NSFL1 (p97) cofactor (p47) | other |  |  | 3,64E-13 | -2,139 |  |  |
| 217832_at | SYNCRIP | synaptotagmin binding, cytoplasmic RNA interacting protein | other |  |  | 4,68E-10 | -1,554 |  |  |
| 217836_s_at | YY1AP1 | YY1 associated protein 1 | other |  |  | 3,80E-08 | -1,546 |  |  |
| 217837_s_at | VPS24 | vacuolar protein sorting 24 homolog (S. cerevisiae) | other | 1,83E-14 | -1,494 | 1,83E-14 | -1,770 | 1,83E-14 | -1,596 |
| 217840_at | DDX41 | DEAD (Asp-Glu-Ala-Asp) box polypeptide 41 | enzyme |  |  | 1,13E-10 | -1,827 |  |  |
| 217841_s_at | PPME1 | protein phosphatase methylesterase 1 | enzyme | 3,07E-11 | -1,549 | 3,07E-11 | -1,863 | 3,07E-11 | -1,432 |
| 217843_s_at | MED4 | mediator complex subunit 4 | transcription regulator |  |  | 1,85E-10 | -1,927 |  |  |
| 217852_s_at | ARL8B | ADP-ribosylation factor-like 8B | enzyme |  |  | 5,56E-09 | -1,512 |  |  |
| 217855_x_at | SDF4 | stromal cell derived factor 4 | other | 3,71E-10 | -1,447 | 3,71E-10 | -1,794 |  |  |
| 217860_at | NDUFA10 (includes EG:4705) | NADH dehydrogenase (ubiquinone) 1 alpha subcomplex, 10, 42kDa | enzyme | 2,25E-13 | -1,464 | 2,25E-13 | -1,956 | 2,25E-13 | -1,385 |
| 217861_s_at | PREB | prolactin regulatory element binding | transcription regulator |  |  | 2,01E-08 | -1,824 |  |  |
| 217864_s_at | PIAS1 | protein inhibitor of activated STAT, 1 | transcription regulator |  |  | 1,20E-07 | -1,740 |  |  |
| 217873_at | CAB39 | calcium binding protein 39 | other |  |  | 1,39E-12 | -1,674 |  |  |
| 217879_at | CDC27 | cell division cycle 27 homolog (S. cerevisiae) | other |  |  | 6,52E-10 | -1,723 |  |  |
| 217882_at | TMEM111 | transmembrane protein 111 | other |  |  | 1,34E-08 | -1,557 |  |  |
| 217892_s_at | LIMA1 | LIM domain and actin binding 1 | other |  |  | 3,28E-10 | -1,623 |  |  |
| 217900_at | IARS2 | isoleucyl-tRNA synthetase 2, mitochondrial | enzyme |  |  | 4,21E-11 | -1,708 |  |  |
| 217906_at | KLHDC2 | kelch domain containing 2 | other | 1,89E-11 | -1,475 | 1,89E-11 | -1,653 | 1,89E-11 | -1,459 |
| 217908_s_at | IQWD1 | IQ motif and WD repeats 1 | other | 1,83E-14 | -1,427 | 1,83E-14 | -1,929 | 1,83E-14 | -1,448 |
| 217915_s_at | C15ORF15 | chromosome 15 open reading frame 15 | other |  |  | 2,66E-08 | -1,493 |  |  |
| 217917_s_at | DYNLRB1 | dynein, light chain, roadblock-type 1 | other |  |  | 8,42E-10 | -1,648 |  |  |
| 217926_at | C19ORF53 | chromosome 19 open reading frame 53 | other |  |  | 9,07E-08 | -1,682 |  |  |
| 217927_at | SPCS1 (includes EG:28972) | signal peptidase complex subunit 1 homolog (S. cerevisiae) | peptidase | 1,41E-11 | -1,273 | 1,41E-11 | -1,619 | 1,41E-11 | -1,372 |
| 217928_s_at | SAPS3 | SAPS domain family, member 3 | other | 2,25E-13 | -1,772 | 2,25E-13 | -1,843 |  |  |
| 217930_s_at | TOLLIP | toll interacting protein | other |  |  | 5,40E-08 | -2,076 |  |  |
| 217932_at | MRPS7 | mitochondrial ribosomal protein S7 | other |  |  | 1,66E-10 | -1,653 |  |  |
| 217933_s_at | LAP3 | leucine aminopeptidase 3 | peptidase |  |  | 2,77E-08 | -1,517 |  |  |
| 217945_at | BTBD1 | BTB (POZ) domain containing 1 | other |  |  | 7,22E-10 | -1,693 |  |  |
| 217949_s_at | VKORC1 | vitamin K epoxide reductase complex, subunit 1 | enzyme |  |  | 2,30E-08 | -1,715 |  |  |
| 217950_at | NOSIP | nitric oxide synthase interacting protein | other |  |  | 1,04E-08 | -1,782 |  |  |
| 217955_at | BCL2L13 | BCL2-like 13 (apoptosis facilitator) | other |  |  | 2,92E-09 | -1,876 |  |  |
| 217970_s_at | CNOT6 | CCR4-NOT transcription complex, subunit 6 | other | 2,10E-09 | -1,546 | 2,10E-09 | -1,590 | 2,10E-09 | -1,375 |
| 217971_at | MAPKSP1 | MAPK scaffold protein 1 | other |  |  | 1,40E-07 | -1,510 |  |  |
| 217973_at | DCXR | dicarbonyl/L-xylulose reductase | enzyme |  |  | 1,05E-07 | -1,545 |  |  |
| 217978_s_at | UBE2Q1 | ubiquitin-conjugating enzyme E2Q family member 1 | enzyme |  |  | 3,92E-09 | -1,764 |  |  |
| 217983_s_at | RNASET2 | ribonuclease T2 | enzyme |  |  | 1,58E-09 | -1,748 |  |  |
| 217984_at | RNASET2 | ribonuclease T2 | enzyme |  |  | 3,48E-09 | -1,735 |  |  |
| 217990_at | GMPR2 | guanosine monophosphate reductase 2 | enzyme |  |  | 6,01E-09 | -1,835 | 6,01E-09 | -1,407 |
| 217994_x_at | CPSF3L | cleavage and polyadenylation specific factor 3-like | other |  |  | 1,31E-08 | -1,873 |  |  |
| 217996_at | PHLDA1 | pleckstrin homology-like domain, family A, member 1 | other | 1,83E-14 | -1,429 | 1,83E-14 | -1,869 |  |  |
| 218001_at | MRPS2 | mitochondrial ribosomal protein S2 | other |  |  | 1,83E-14 | -2,158 | 1,83E-14 | -1,362 |
| 218003_s_at | FKBP3 | FK506 binding protein 3, 25kDa | enzyme | 1,52E-11 | -1,548 | 1,52E-11 | -1,497 |  |  |
| 218007_s_at | RPS27L (includes EG:51065) | ribosomal protein S27-like | other |  |  | 3,27E-09 | -1,589 |  |  |
| 218009_s_at | PRC1 | protein regulator of cytokinesis 1 | other | 3,48E-11 | -1,426 | 3,48E-11 | -1,578 |  |  |
| 218014_at | NUP85 | nucleoporin 85kDa | other |  |  | 1,68E-09 | -1,591 |  |  |
| 218016_s_at | POLR3E | polymerase (RNA) III (DNA directed) polypeptide E (80kD) | transcription regulator |  |  | 1,98E-10 | -1,644 |  |  |
| 218023_s_at | FAM53C | family with sequence similarity 53, member C | other |  |  | 5,27E-08 | -1,694 |  |  |
| 218025_s_at | PECI | peroxisomal D3,D2-enoyl-CoA isomerase | enzyme |  |  | 1,23E-07 | -1,643 |  |  |
| 218026_at | CCDC56 | coiled-coil domain containing 56 | other |  |  | 1,72E-09 | -1,567 |  |  |
| 218032_at | SNN | stannin | other | 1,65E-08 | -1,618 |  |  | 1,65E-08 | -1,581 |
| 218034_at | FIS1 | fission 1 (mitochondrial outer membrane) homolog (S. cerevisiae) | other | 2,43E-13 | -1,357 | 2,43E-13 | -1,988 | 2,43E-13 | -1,500 |
| 218035_s_at | RBM47 | RNA binding motif protein 47 | other |  |  | 1,53E-09 | -1,652 |  |  |
| 218039_at | NUSAP1 | nucleolar and spindle associated protein 1 | other | 2,02E-10 | -1,542 | 2,02E-10 | -1,477 | 2,02E-10 | -1,346 |
| 218041_x_at | SLC38A2 | solute carrier family 38, member 2 | transporter |  |  | 2,93E-11 | -1,558 |  |  |
| 218042_at | COPS4 | COP9 constitutive photomorphogenic homolog subunit 4 (Arabidopsis) | other |  |  | 1,00E-09 | -1,563 |  |  |
| 218043_s_at | AZI2 | 5-azacytidine induced 2 | other | 1,83E-14 | -1,942 | 1,83E-14 | -2,219 | 1,83E-14 | -1,914 |
| 218047_at | OSBPL9 | oxysterol binding protein-like 9 | other |  |  | 7,19E-08 | -1,515 |  |  |
| 218049_s_at | MRPL13 | mitochondrial ribosomal protein L13 | other |  |  | 3,71E-10 | -1,611 |  |  |
| 218051_s_at | NT5DC2 | 5'-nucleotidase domain containing 2 | other | 1,83E-14 | -1,417 | 1,83E-14 | -2,208 | 1,83E-14 | -1,411 |
| 218059_at | ZNF706 | zinc finger protein 706 | other |  |  | 2,17E-12 | -1,840 |  |  |
| 218061_at | MEA1 | male-enhanced antigen 1 | other |  |  | 5,85E-09 | -1,647 |  |  |
| 218062_x_at | CDC42EP4 | CDC42 effector protein (Rho GTPase binding) 4 | other |  |  | 1,68E-07 | -2,206 |  |  |
| 218079_s_at | GGNBP2 | gametogenetin binding protein 2 | other | 2,92E-11 | -1,486 | 2,92E-11 | -1,743 |  |  |
| 218082_s_at | UBP1 | upstream binding protein 1 (LBP-1a) | transcription regulator |  |  | 1,67E-09 | -1,591 |  |  |
| 218084_x_at | FXYD5 | FXYD domain containing ion transport regulator 5 | ion channel |  |  | 1,01E-09 | -1,930 |  |  |
| 218089_at | C20ORF4 | chromosome 20 open reading frame 4 | other |  |  | 1,43E-10 | -1,805 |  |  |
| 218094_s_at | DBNDD2 | dysbindin (dystrobrevin binding protein 1) domain containing 2 | other |  |  | 2,22E-08 | -1,853 |  |  |
| 218097_s_at | CUEDC2 | CUE domain containing 2 | other |  |  | 1,87E-10 | -1,867 |  |  |
| 218098_at | ARFGEF2 | ADP-ribosylation factor guanine nucleotide-exchange factor 2 (brefeldin A-inhibited) | other | 2,04E-09 | -1,535 |  |  |  |  |
| 218102_at | DERA | 2-deoxyribose-5-phosphate aldolase homolog (C. elegans) | enzyme |  |  | 2,53E-07 | -1,451 |  |  |
| 218105_s_at | MRPL4 | mitochondrial ribosomal protein L4 | other |  |  | 1,66E-11 | -2,025 |  |  |
| 218106_s_at | MRPS10 | mitochondrial ribosomal protein S10 | other | 6,85E-13 | -1,380 | 6,85E-13 | -1,780 |  |  |
| 218107_at | WDR26 | WD repeat domain 26 | other |  |  | 1,68E-09 | -1,660 |  |  |
| 218111_s_at | CMAS | cytidine monophosphate N-acetylneuraminic acid synthetase | enzyme | 5,46E-08 | -1,445 |  |  |  |  |
| 218114_at | GGA1 | golgi associated, gamma adaptin ear containing, ARF binding protein 1 | transporter |  |  | 2,39E-10 | -2,887 |  |  |
| 218115_at | ASF1B | ASF1 anti-silencing function 1 homolog B (S. cerevisiae) | other | 2,62E-10 | -1,492 | 2,62E-10 | -1,711 | 2,62E-10 | -1,497 |
| 218117_at | RBX1 (includes EG:9978) | ring-box 1 | enzyme |  |  | 5,85E-10 | -1,582 |  |  |
| 218118_s_at | TIMM23 | translocase of inner mitochondrial membrane 23 homolog (yeast) | transporter |  |  | 4,19E-09 | -1,612 |  |  |
| 218120_s_at | HMOX2 | heme oxygenase (decycling) 2 | enzyme | 2,06E-13 | -1,606 | 2,06E-13 | -1,998 | 2,06E-13 | -1,463 |
| 218127_at | NFYB | nuclear transcription factor Y, beta | transcription regulator | 3,40E-08 | -1,503 |  |  |  |  |
| 218131_s_at | GATAD2A | GATA zinc finger domain containing 2A | transcription regulator |  |  | 5,62E-10 | -1,841 |  |  |
| 218134_s_at | RBM22 | RNA binding motif protein 22 | other | 5,06E-10 | -1,514 | 5,06E-10 | -1,527 | 5,06E-10 | -1,411 |
| 218147_s_at | GLT8D1 | glycosyltransferase 8 domain containing 1 | enzyme |  |  | 3,96E-08 | -1,686 |  |  |
| 218155_x_at | TSR1 | TSR1, 20S rRNA accumulation, homolog (S. cerevisiae) | other |  |  | 2,99E-13 | -2,412 |  |  |
| 218162_at | OLFML3 | olfactomedin-like 3 | other | 1,97E-06 | -1,908 |  |  |  |  |
| 218163_at | MCTS1 | malignant T cell amplified sequence 1 | other | 1,32E-10 | -1,351 | 1,32E-10 | -1,623 | 1,32E-10 | -1,381 |
| 218164_at | SPATA20 | spermatogenesis associated 20 | other | 1,83E-14 | -1,504 | 1,83E-14 | -2,152 | 1,83E-14 | -1,518 |
| 218166_s_at | RSF1 | remodeling and spacing factor 1 | transcription regulator | 1,03E-08 | -1,642 | 1,03E-08 | -1,597 | 1,03E-08 | -1,383 |
| 218167_at | AMZ2 | archaelysin family metallopeptidase 2 | other | 1,92E-10 | -1,348 | 1,92E-10 | -1,686 |  |  |
| 218168_s_at | CABC1 | chaperone, ABC1 activity of bc1 complex homolog (S. pombe) | kinase |  |  | 1,33E-09 | -1,873 |  |  |
| 218170_at | ISOC1 | isochorismatase domain containing 1 | enzyme |  |  | 3,95E-09 | -1,480 | 3,95E-09 | -1,261 |
| 218174_s_at | C10ORF57 | chromosome 10 open reading frame 57 | other | 6,75E-09 | -2,362 | 6,75E-09 | -1,953 |  |  |
| 218180_s_at | EPS8L2 | EPS8-like 2 | other | 2,36E-10 | -1,535 | 2,36E-10 | -1,942 | 2,36E-10 | -1,661 |
| 218186_at | RAB25 | RAB25, member RAS oncogene family | enzyme |  |  | 3,54E-12 | -1,818 |  |  |
| 218187_s_at | C8ORF33 | chromosome 8 open reading frame 33 | other |  |  | 9,17E-10 | -1,869 |  |  |
| 218193_s_at | GOLT1B | golgi transport 1 homolog B (S. cerevisiae) | other |  |  |  |  | 8,75E-08 | -1,504 |
| 218194_at | REXO2 | REX2, RNA exonuclease 2 homolog (S. cerevisiae) | enzyme |  |  | 7,52E-08 | -1,501 |  |  |
| 218195_at | C6ORF211 | chromosome 6 open reading frame 211 | other | 3,41E-12 | -1,510 | 3,41E-12 | -1,595 | 3,41E-12 | -1,387 |
| 218200_s_at | NDUFB2 | NADH dehydrogenase (ubiquinone) 1 beta subcomplex, 2, 8kDa | enzyme |  |  | 1,69E-09 | -1,610 |  |  |
| 218201_at | NDUFB2 | NADH dehydrogenase (ubiquinone) 1 beta subcomplex, 2, 8kDa | enzyme | 5,94E-12 | -1,362 | 5,94E-12 | -1,901 |  |  |
| 218203_at | ALG5 | asparagine-linked glycosylation 5, dolichyl-phosphate beta-glucosyltransferase homolog (S. cerevisiae) | enzyme |  |  | 8,92E-08 | -1,526 |  |  |
| 218205_s_at | MKNK2 | MAP kinase interacting serine/threonine kinase 2 | kinase |  |  | 2,76E-09 | -1,629 |  |  |
| 218206_x_at | SCAND1 | SCAN domain containing 1 | transcription regulator |  |  | 3,18E-08 | -1,690 |  |  |
| 218213_s_at | C11ORF10 | chromosome 11 open reading frame 10 | other |  |  | 4,39E-12 | -1,663 |  |  |
| 218217_at | SCPEP1 | serine carboxypeptidase 1 | peptidase | 4,96E-08 | -1,450 |  |  |  |  |
| 218218_at | APPL2 | adaptor protein, phosphotyrosine interaction, PH domain and leucine zipper containing 2 | other | 9,71E-10 | -1,572 | 9,71E-10 | -1,634 | 9,71E-10 | -1,349 |
| 218220_at | C12ORF10 | chromosome 12 open reading frame 10 | other |  |  | 2,50E-11 | -1,936 |  |  |
| 218227_at | NUBP2 | nucleotide binding protein 2 (MinD homolog, E. coli) | other | 4,33E-08 | -1,714 | 4,33E-08 | -2,225 |  |  |
| 218236_s_at | PRKD3 | protein kinase D3 | kinase |  |  | 9,74E-11 | -1,587 |  |  |
| 218237_s_at | SLC38A1 | solute carrier family 38, member 1 | transporter |  |  | 1,65E-10 | -1,526 |  |  |
| 218241_at | GOLGA5 | golgi autoantigen, golgin subfamily a, 5 | kinase |  |  | 4,59E-08 | -1,537 |  |  |
| 218248_at | FAM111A | family with sequence similarity 111, member A | other | 7,65E-10 | -1,632 | 7,65E-10 | -1,637 |  |  |
| 218251_at | MID1IP1 | MID1 interacting protein 1 (gastrulation specific G12 homolog (zebrafish)) | other |  |  | 1,29E-08 | -2,030 |  |  |
| 218252_at | CKAP2 | cytoskeleton associated protein 2 | other | 6,42E-13 | -1,738 | 6,42E-13 | -1,506 | 6,42E-13 | -1,291 |
| 218254_s_at | SAR1B | SAR1 homolog B (S. cerevisiae) | enzyme |  |  | 1,68E-09 | -1,454 | 1,68E-09 | -1,553 |
| 218259_at | MKL2 | MKL/myocardin-like 2 | transcription regulator | 2,37E-09 | -1,487 | 2,37E-09 | -1,616 |  |  |
| 218262_at | RMND5B | required for meiotic nuclear division 5 homolog B (S. cerevisiae) | other |  |  | 5,53E-09 | -1,910 |  |  |
| 218269_at | RNASEN | ribonuclease type III, nuclear | enzyme | 2,79E-12 | -1,482 | 2,79E-12 | -1,692 |  |  |
| 218271_s_at | PARL | presenilin associated, rhomboid-like | other |  |  | 5,09E-11 | -1,613 |  |  |
| 218277_s_at | DHX40 | DEAH (Asp-Glu-Ala-His) box polypeptide 40 | enzyme | 2,16E-09 | -1,383 | 2,16E-09 | -1,531 | 2,16E-09 | -1,335 |
| 218281_at | MRPL48 | mitochondrial ribosomal protein L48 | other |  |  | 1,95E-09 | -1,663 |  |  |
| 218282_at | EDEM2 | ER degradation enhancer, mannosidase alpha-like 2 | enzyme | 1,01E-13 | -1,692 | 1,01E-13 | -1,811 | 1,01E-13 | -1,365 |
| 218283_at | SS18L2 | synovial sarcoma translocation gene on chromosome 18-like 2 | other |  |  | 1,42E-08 | -1,598 |  |  |
| 218286_s_at | RNF7 | ring finger protein 7 | other | 1,10E-12 | -1,320 | 1,10E-12 | -1,775 |  |  |
| 218288_s_at | CCDC90B | coiled-coil domain containing 90B | other |  |  | 2,01E-08 | -1,494 |  |  |
| 218289_s_at | UBA5 | ubiquitin-like modifier activating enzyme 5 | enzyme |  |  | 1,39E-11 | -1,892 |  |  |
| 218290_at | PLEKHJ1 | pleckstrin homology domain containing, family J member 1 | other |  |  | 6,40E-08 | -1,828 |  |  |
| 218303_x_at | KRCC1 | lysine-rich coiled-coil 1 | other |  |  | 8,16E-08 | -1,812 |  |  |
| 218304_s_at | OSBPL11 | oxysterol binding protein-like 11 | other |  |  | 8,29E-08 | -1,533 |  |  |
| 218308_at | TACC3 | transforming, acidic coiled-coil containing protein 3 | other |  |  | 2,99E-09 | -2,038 |  |  |
| 218309_at | CAMK2N1 | calcium/calmodulin-dependent protein kinase II inhibitor 1 | kinase | 1,83E-14 | -1,678 | 1,83E-14 | -2,217 | 1,83E-14 | -1,461 |
| 218311_at | MAP4K3 | mitogen-activated protein kinase kinase kinase kinase 3 | kinase | 5,84E-09 | -1,449 | 5,84E-09 | -1,573 |  |  |
| 218319_at | PELI1 | pellino homolog 1 (Drosophila) | other | 1,83E-14 | -1,782 | 1,83E-14 | -1,720 | 1,83E-14 | -1,337 |
| 218320_s_at | NDUFB11 | NADH dehydrogenase (ubiquinone) 1 beta subcomplex, 11, 17.3kDa | enzyme |  |  | 1,32E-08 | -1,711 |  |  |
| 218322_s_at | ACSL5 | acyl-CoA synthetase long-chain family member 5 | enzyme | 1,83E-14 | 1,900 |  |  | 1,83E-14 | 2,521 |
| 218326_s_at | LGR4 | leucine-rich repeat-containing G protein-coupled receptor 4 | G-protein coupled receptor |  |  | 3,06E-08 | -1,593 | 3,06E-08 | -1,431 |
| 218330_s_at | NAV2 (includes EG:89797) | neuron navigator 2 | other | 2,78E-11 | -1,771 | 2,78E-11 | -1,993 | 2,78E-11 | -1,834 |
| 218333_at | DERL2 | Der1-like domain family, member 2 | other | 1,87E-09 | -1,666 | 1,87E-09 | -1,519 |  |  |
| 218334_at | THOC7 | THO complex 7 homolog (Drosophila) | other |  |  | 1,83E-14 | -1,925 |  |  |
| 218339_at | MRPL22 | mitochondrial ribosomal protein L22 | other |  |  | 2,19E-07 | -1,522 |  |  |
| 218343_s_at | GTF3C3 | general transcription factor IIIC, polypeptide 3, 102kDa | transcription regulator | 3,44E-14 | -1,491 | 3,44E-14 | -1,818 |  |  |
| 218351_at | COMMD8 | COMM domain containing 8 | other |  |  | 3,15E-08 | -1,495 |  |  |
| 218354_at | TRAPPC2L | trafficking protein particle complex 2-like | other |  |  | 2,36E-08 | -1,673 |  |  |
| 218355_at | KIF4A | kinesin family member 4A | other | 2,06E-13 | -1,527 | 2,06E-13 | -1,764 | 2,06E-13 | -1,298 |
| 218356_at | FTSJ2 | FtsJ homolog 2 (E. coli) | enzyme | 2,96E-09 | -1,334 | 2,96E-09 | -1,533 |  |  |
| 218357_s_at | TIMM8B | translocase of inner mitochondrial membrane 8 homolog B (yeast) | transporter |  |  | 2,00E-07 | -1,568 |  |  |
| 218358_at | CRELD2 | cysteine-rich with EGF-like domains 2 | other |  |  | 3,23E-11 | -1,924 |  |  |
| 218360_at | RAB22A | RAB22A, member RAS oncogene family | enzyme |  |  | 5,19E-09 | -1,599 |  |  |
| 218378_s_at | PRKRIP1 | PRKR interacting protein 1 (IL11 inducible) | other |  |  | 1,66E-09 | -2,052 | 1,66E-09 | -1,466 |
| 218381_s_at | U2AF2 (includes EG:11338) | U2 small nuclear RNA auxiliary factor 2 | other | 1,89E-12 | -1,443 | 1,89E-12 | -1,910 | 1,89E-12 | -1,639 |
| 218383_at | C14ORF94 | chromosome 14 open reading frame 94 | other |  |  | 5,36E-10 | -1,799 | 5,36E-10 | -1,423 |
| 218385_at | MRPS18A | mitochondrial ribosomal protein S18A | other |  |  | 8,20E-10 | -1,813 |  |  |
| 218388_at | PGLS | 6-phosphogluconolactonase | enzyme |  |  | 3,22E-08 | -1,762 |  |  |
| 218391_at | SNF8 | SNF8, ESCRT-II complex subunit, homolog (S. cerevisiae) | enzyme |  |  | 1,41E-08 | -1,520 |  |  |
| 218398_at | MRPS30 | mitochondrial ribosomal protein S30 | enzyme |  |  | 4,27E-08 | -1,619 |  |  |
| 218399_s_at | CDCA4 | cell division cycle associated 4 | other | 1,83E-14 | -1,489 | 1,83E-14 | -2,294 |  |  |
| 218407_x_at | NENF | neuron derived neurotrophic factor | growth factor |  |  | 3,41E-10 | -2,003 |  |  |
| 218408_at | TIMM10 | translocase of inner mitochondrial membrane 10 homolog (yeast) | transporter |  |  | 3,93E-10 | -1,836 |  |  |
| 218411_s_at | MBIP | MAP3K12 binding inhibitory protein 1 | other |  |  |  |  | 5,16E-08 | -1,412 |
| 218418_s_at | KANK2 | KN motif and ankyrin repeat domains 2 | transcription regulator | 1,83E-14 | -1,485 | 1,83E-14 | -2,179 | 1,83E-14 | -1,858 |
| 218425_at | RNF216 | ring finger protein 216 | other |  |  | 3,89E-08 | -3,017 |  |  |
| 218427_at | SDCCAG3 (includes EG:10807) | serologically defined colon cancer antigen 3 | other |  |  |  |  | 7,23E-08 | -1,474 |
| 218435_at | DNAJC15 | DnaJ (Hsp40) homolog, subfamily C, member 15 | other | 4,18E-11 | -1,392 | 4,18E-11 | -1,688 | 4,18E-11 | -1,558 |
| 218462_at | BXDC5 | brix domain containing 5 | other | 5,05E-12 | -1,556 | 5,05E-12 | -1,815 |  |  |
| 218467_at | PSMG2 | proteasome (prosome, macropain) assembly chaperone 2 | other |  |  | 4,21E-09 | -1,631 |  |  |
| 218468_s_at | GREM1 | gremlin 1, cysteine knot superfamily, homolog (Xenopus laevis) | other |  |  |  |  | 5,33E-12 | 2,012 |
| 218469_at | GREM1 | gremlin 1, cysteine knot superfamily, homolog (Xenopus laevis) | other |  |  |  |  | 1,83E-14 | 2,334 |
| 218473_s_at | GLT25D1 | glycosyltransferase 25 domain containing 1 | other |  |  | 3,22E-10 | -1,867 | 3,22E-10 | -1,477 |
| 218474_s_at | KCTD5 | potassium channel tetramerisation domain containing 5 | ion channel |  |  | 8,84E-08 | -1,657 |  |  |
| 218477_at | TMEM14A | transmembrane protein 14A | other | 8,45E-13 | -1,367 | 8,45E-13 | -1,676 | 8,45E-13 | -1,459 |
| 218478_s_at | ZCCHC8 | zinc finger, CCHC domain containing 8 | other | 1,83E-14 | -1,606 | 1,83E-14 | -1,727 |  |  |
| 218482_at | ENY2 | enhancer of yellow 2 homolog (Drosophila) | other |  |  | 1,01E-12 | -1,889 |  |  |
| 218490_s_at | ZNF302 | zinc finger protein 302 | other |  |  | 2,67E-08 | -1,579 | 2,67E-08 | -1,371 |
| 218491_s_at | THYN1 | thymocyte nuclear protein 1 | other |  |  | 5,92E-09 | -1,553 |  |  |
| 218492_s_at | THAP7 | THAP domain containing 7 | other |  |  | 1,74E-08 | -2,339 |  |  |
| 218493_at | SNRNP25 | small nuclear ribonucleoprotein 25kDa (U11/U12) | other | 1,37E-09 | -1,343 | 1,37E-09 | -1,672 |  |  |
| 218494_s_at | SLC2A4RG | SLC2A4 regulator | transcription regulator | 5,00E-13 | -1,385 | 5,00E-13 | -1,865 | 5,00E-13 | -1,857 |
| 218495_at | UXT | ubiquitously-expressed transcript | other | 9,27E-11 | -1,506 | 9,27E-11 | -1,741 | 9,27E-11 | -1,629 |
| 218515_at | C21ORF66 | chromosome 21 open reading frame 66 | other |  |  | 9,21E-10 | -1,716 |  |  |
| 218520_at | TBK1 | TANK-binding kinase 1 | kinase |  |  | 5,83E-09 | -1,640 |  |  |
| 218531_at | TMEM134 | transmembrane protein 134 | kinase | 1,83E-14 | -1,730 | 1,83E-14 | -2,240 | 1,83E-14 | -1,822 |
| 218535_s_at | RIOK2 | RIO kinase 2 (yeast) | kinase |  |  | 4,10E-08 | -1,646 |  |  |
| 218538_s_at | MRS2 | MRS2 magnesium homeostasis factor homolog (S. cerevisiae) | transporter |  |  | 1,63E-10 | -1,596 |  |  |
| 218549_s_at | FAM82B | family with sequence similarity 82, member B | other | 3,59E-12 | -1,351 | 3,59E-12 | -1,800 |  |  |
| 218556_at | ORMDL2 | ORM1-like 2 (S. cerevisiae) | other |  |  | 2,08E-10 | -1,672 | 2,08E-10 | -1,548 |
| 218558_s_at | MRPL39 | mitochondrial ribosomal protein L39 | other |  |  | 3,10E-09 | -1,614 |  |  |
| 218570_at | KBTBD4 | kelch repeat and BTB (POZ) domain containing 4 | other | 2,76E-08 | -1,521 |  |  | 2,76E-08 | -1,501 |
| 218571_s_at | CHMP4A | chromatin modifying protein 4A | other |  |  | 6,42E-09 | -1,772 |  |  |
| 218572_at | CHMP4A | chromatin modifying protein 4A | other |  |  | 9,03E-09 | -1,574 |  |  |
| 218574_s_at | LMCD1 | LIM and cysteine-rich domains 1 | transcription regulator |  |  | 2,06E-13 | -1,782 |  |  |
| 218575_at | ANAPC1 | anaphase promoting complex subunit 1 | other |  |  | 1,05E-09 | -1,859 |  |  |
| 218577_at | LRRC40 | leucine rich repeat containing 40 | other |  |  | 9,72E-10 | -1,609 |  |  |
| 218580_x_at | AURKAIP1 | aurora kinase A interacting protein 1 | enzyme |  |  | 9,05E-09 | -1,623 |  |  |
| 218581_at | ABHD4 | abhydrolase domain containing 4 | peptidase |  |  | 2,82E-10 | -2,368 | 2,82E-10 | -2,149 |
| 218582_at | MARCH5 | membrane-associated ring finger (C3HC4) 5 | other |  |  | 1,03E-08 | -1,640 |  |  |
| 218583_s_at | DCUN1D1 | DCN1, defective in cullin neddylation 1, domain containing 1 (S. cerevisiae) | other |  |  | 3,81E-08 | -1,531 |  |  |
| 218586_at | C20ORF20 | chromosome 20 open reading frame 20 | other |  |  | 1,28E-09 | -1,743 |  |  |
| 218604_at | LEMD3 | LEM domain containing 3 | other |  |  | 3,16E-08 | -1,620 |  |  |
| 218606_at | ZDHHC7 | zinc finger, DHHC-type containing 7 | enzyme |  |  | 1,66E-07 | -1,541 |  |  |
| 218610_s_at | FLJ11151 | hypothetical protein FLJ11151 | enzyme | 5,62E-10 | -1,917 | 5,62E-10 | -2,270 |  |  |
| 218640_s_at | PLEKHF2 (includes EG:79666) | pleckstrin homology domain containing, family F (with FYVE domain) member 2 | other |  |  | 1,95E-09 | -1,707 |  |  |
| 218641_at | MGC3032 | hypothetical protein MGC3032 | other |  |  | 1,85E-13 | -2,219 |  |  |
| 218646_at | C4ORF27 | chromosome 4 open reading frame 27 | other | 6,73E-10 | -1,448 | 6,73E-10 | -1,594 | 6,73E-10 | -1,531 |
| 218648_at | CRTC3 | CREB regulated transcription coactivator 3 | other |  |  | 1,99E-08 | -1,653 |  |  |
| 218654_s_at | MRPS33 | mitochondrial ribosomal protein S33 | other |  |  | 5,25E-09 | -1,683 |  |  |
| 218661_at | NAT15 | N-acetyltransferase 15 (GCN5-related, putative) | enzyme |  |  | 6,44E-08 | -2,194 |  |  |
| 218676_s_at | PCTP | phosphatidylcholine transfer protein | transporter | 1,06E-08 | -1,422 | 1,06E-08 | -1,725 |  |  |
| 218682_s_at | SLC4A1AP | solute carrier family 4 (anion exchanger), member 1, adaptor protein | transporter |  |  | 6,46E-10 | -2,006 |  |  |
| 218684_at | LRRC8D | leucine rich repeat containing 8 family, member D | G-protein coupled receptor |  |  | 6,31E-08 | -1,869 |  |  |
| 218685_s_at | SMUG1 | single-strand-selective monofunctional uracil-DNA glycosylase 1 | enzyme |  |  | 3,41E-08 | -1,772 |  |  |
| 218692_at | GOLSYN | Golgi-localized protein | other | 1,31E-10 | -1,538 | 1,31E-10 | -1,673 | 1,31E-10 | -1,734 |
| 218693_at | TSPAN15 | tetraspanin 15 | other |  |  | 4,41E-13 | -2,228 |  |  |
| 218701_at | LACTB2 | lactamase, beta 2 | other | 1,19E-12 | -1,438 | 1,19E-12 | -1,383 | 1,19E-12 | -1,812 |
| 218702_at | SARS2 | seryl-tRNA synthetase 2, mitochondrial | enzyme |  |  | 5,91E-08 | -2,496 |  |  |
| 218703_at | SEC22A | SEC22 vesicle trafficking protein homolog A (S. cerevisiae) | transporter |  |  | 6,05E-10 | -1,964 |  |  |
| 218705_s_at | SNX24 | sorting nexin 24 | transporter | 2,92E-10 | -1,410 | 2,92E-10 | -1,905 |  |  |
| 218709_s_at | IFT52 | intraflagellar transport 52 homolog (Chlamydomonas) | other |  |  | 9,77E-10 | -1,618 |  |  |
| 218716_x_at | MTO1 | mitochondrial translation optimization 1 homolog (S. cerevisiae) | enzyme |  |  | 7,04E-08 | -1,714 |  |  |
| 218718_at | PDGFC | platelet derived growth factor C | growth factor | 1,06E-09 | -1,588 | 1,06E-09 | -1,576 | 1,06E-09 | -1,374 |
| 218722_s_at | CCDC51 | coiled-coil domain containing 51 | other |  |  | 3,35E-07 | -1,696 |  |  |
| 218723_s_at | C13ORF15 | chromosome 13 open reading frame 15 | other | 5,18E-13 | -1,601 | 5,18E-13 | -1,899 | 5,18E-13 | -1,384 |
| 218728_s_at | CNIH4 | cornichon homolog 4 (Drosophila) | other | 8,51E-10 | -1,577 | 8,51E-10 | -1,553 |  |  |
| 218740_s_at | CDK5RAP3 (includes EG:80279) | CDK5 regulatory subunit associated protein 3 | other |  |  | 2,95E-09 | -1,681 | 2,95E-09 | -1,359 |
| 218756_s_at | DHRS11 | dehydrogenase/reductase (SDR family) member 11 | enzyme |  |  | 2,08E-08 | -1,874 |  |  |
| 218757_s_at | UPF3B | UPF3 regulator of nonsense transcripts homolog B (yeast) | transporter | 1,99E-11 | -1,455 | 1,99E-11 | -1,794 | 1,99E-11 | -1,373 |
| 218767_at | REXO4 | REX4, RNA exonuclease 4 homolog (S. cerevisiae) | transcription regulator |  |  | 8,41E-12 | -2,088 |  |  |
| 218768_at | NUP107 | nucleoporin 107kDa | transporter |  |  | 2,96E-09 | -1,539 |  |  |
| 218771_at | PANK4 | pantothenate kinase 4 | kinase |  |  | 8,77E-11 | -2,270 |  |  |
| 218772_x_at | TMEM38B | transmembrane protein 38B | other |  |  | 6,97E-08 | -1,583 |  |  |
| 218773_s_at | MSRB2 | methionine sulfoxide reductase B2 | transcription regulator |  |  | 1,94E-08 | -1,673 |  |  |
| 218777_at | REEP4 | receptor accessory protein 4 | other |  |  | 1,15E-08 | -2,134 |  |  |
| 218782_s_at | ATAD2 | ATPase family, AAA domain containing 2 | other | 7,14E-11 | -1,548 | 7,14E-11 | -1,721 | 7,14E-11 | -1,380 |
| 218794_s_at | TXNL4B | thioredoxin-like 4B | enzyme | 4,47E-10 | -1,656 | 4,47E-10 | -1,922 | 4,47E-10 | -1,549 |
| 218795_at | ACP6 | acid phosphatase 6, lysophosphatidic | phosphatase |  |  | 2,08E-07 | -1,652 |  |  |
| 218797_s_at | SIRT7 | sirtuin (silent mating type information regulation 2 homolog) 7 (S. cerevisiae) | enzyme |  |  | 1,28E-08 | -1,894 |  |  |
| 218823_s_at | KCTD9 | potassium channel tetramerisation domain containing 9 | other |  |  | 1,31E-10 | -1,621 |  |  |
| 218830_at | RPL26L1 | ribosomal protein L26-like 1 | other |  |  | 5,54E-08 | -1,555 |  |  |
| 218840_s_at | NADSYN1 | NAD synthetase 1 | enzyme | 2,23E-09 | -1,563 | 2,23E-09 | -1,805 | 2,23E-09 | -1,609 |
| 218846_at | MED23 | mediator complex subunit 23 | transcription regulator | 7,17E-12 | -1,628 | 7,17E-12 | -1,712 |  |  |
| 218852_at | PPP2R3C | protein phosphatase 2 (formerly 2A), regulatory subunit B'', gamma | other |  |  | 5,25E-09 | -1,633 |  |  |
| 218856_at | TNFRSF21 | tumor necrosis factor receptor superfamily, member 21 | other |  |  | 2,30E-12 | -1,491 |  |  |
| 218859_s_at | ESF1 | ESF1, nucleolar pre-rRNA processing protein, homolog (S. cerevisiae) | other |  |  |  |  | 3,99E-09 | 1,551 |
| 218866_s_at | POLR3K | polymerase (RNA) III (DNA directed) polypeptide K, 12.3 kDa | transcription regulator |  |  | 2,44E-08 | -1,600 |  |  |
| 218875_s_at | FBXO5 | F-box protein 5 | enzyme |  |  | 1,25E-07 | -1,559 |  |  |
| 218878_s_at | SIRT1 | sirtuin (silent mating type information regulation 2 homolog) 1 (S. cerevisiae) | transcription regulator | 1,83E-14 | -1,749 | 1,83E-14 | -1,865 | 1,83E-14 | -1,597 |
| 218890_x_at | MRPL35 | mitochondrial ribosomal protein L35 | other |  |  | 5,17E-12 | -1,760 |  |  |
| 218893_at | ISOC2 | isochorismatase domain containing 2 | enzyme |  |  | 6,06E-09 | -2,420 |  |  |
| 218903_s_at | OBFC2B | oligonucleotide/oligosaccharide-binding fold containing 2B | other |  |  | 4,08E-08 | -1,973 |  |  |
| 218906_x_at | KLC2 | kinesin light chain 2 | other |  |  | 2,41E-09 | -3,657 |  |  |
| 218915_at | NF2 | neurofibromin 2 (merlin) | other | 3,39E-10 | -1,929 | 3,39E-10 | -1,972 | 3,39E-10 | -1,550 |
| 218920_at | FLJ10404 | hypothetical protein FLJ10404 | other | 4,02E-10 | -1,735 | 4,02E-10 | -2,290 |  |  |
| 218945_at | C16ORF68 | chromosome 16 open reading frame 68 | enzyme |  |  | 3,81E-09 | -2,152 |  |  |
| 218946_at | NFU1 | NFU1 iron-sulfur cluster scaffold homolog (S. cerevisiae) | other |  |  | 4,08E-10 | -1,555 |  |  |
| 218957_s_at | PAAF1 | proteasomal ATPase-associated factor 1 | other | 9,21E-09 | -1,487 | 9,21E-09 | -1,542 | 9,21E-09 | -1,350 |
| 218982_s_at | MRPS17 | mitochondrial ribosomal protein S17 | other |  |  | 4,98E-08 | -1,545 |  |  |
| 218983_at | C1RL | complement component 1, r subcomponent-like | peptidase |  |  | 9,59E-10 | -2,139 |  |  |
| 218997_at | POLR1E | polymerase (RNA) I polypeptide E, 53kDa | enzyme | 3,28E-09 | -1,657 | 3,28E-09 | -1,984 | 3,28E-09 | -1,525 |
| 219006_at | C6ORF66 | chromosome 6 open reading frame 66 | other |  |  | 7,29E-10 | -1,592 |  |  |
| 219015_s_at | ALG13 (includes EG:79868) | asparagine-linked glycosylation 13 homolog (S. cerevisiae) | other | 4,22E-09 | -1,393 | 4,22E-09 | -1,683 |  |  |
| 219030_at | TPRKB | TP53RK binding protein | other |  |  | 1,26E-13 | -1,767 | 1,26E-13 | -1,463 |
| 219038_at | MORC4 | MORC family CW-type zinc finger 4 | other |  |  | 6,32E-13 | -1,786 |  |  |
| 219041_s_at | REPIN1 | replication initiator 1 | other |  |  | 1,82E-09 | -1,694 |  |  |
| 219045_at | RHOF | ras homolog gene family, member F (in filopodia) | enzyme |  |  | 3,84E-10 | -2,205 |  |  |
| 219061_s_at | LAGE3 | L antigen family, member 3 | other |  |  | 1,68E-09 | -1,829 |  |  |
| 219067_s_at | NSMCE4A | non-SMC element 4 homolog A (S. cerevisiae) | other |  |  | 4,41E-09 | -1,644 |  |  |
| 219072_at | BCL7C | B-cell CLL/lymphoma 7C | other | 3,44E-14 | -1,849 | 3,44E-14 | -2,416 | 3,44E-14 | -1,920 |
| 219099_at | C12ORF5 | chromosome 12 open reading frame 5 | enzyme |  |  | 2,67E-08 | -1,434 |  |  |
| 219109_at | SPAG16 | sperm associated antigen 16 | other | 4,95E-10 | -1,560 | 4,95E-10 | -1,596 | 4,95E-10 | -1,468 |
| 219117_s_at | FKBP11 | FK506 binding protein 11, 19 kDa | enzyme |  |  | 6,51E-09 | -1,455 |  |  |
| 219118_at | FKBP11 | FK506 binding protein 11, 19 kDa | enzyme |  |  | 1,83E-14 | -1,815 |  |  |
| 219119_at | LSM8 | LSM8 homolog, U6 small nuclear RNA associated (S. cerevisiae) | other | 1,48E-11 | -1,336 | 1,48E-11 | -1,684 | 1,48E-11 | -1,459 |
| 219121_s_at | RBM35A | RNA binding motif protein 35A | other |  |  | 6,40E-08 | -1,604 |  |  |
| 219126_at | PHF10 | PHD finger protein 10 | other |  |  | 1,10E-07 | -1,517 |  |  |
| 219140_s_at | RBP4 | retinol binding protein 4, plasma | transporter |  |  | 6,04E-09 | -1,735 |  |  |
| 219148_at | PBK | PDZ binding kinase | kinase | 1,83E-14 | -1,543 | 1,83E-14 | -1,817 |  |  |
| 219149_x_at | DBR1 | debranching enzyme homolog 1 (S. cerevisiae) | enzyme | 3,36E-12 | -1,939 | 3,36E-12 | -2,055 |  |  |
| 219181_at | LIPG | lipase, endothelial | enzyme |  |  | 3,08E-09 | -1,613 |  |  |
| 219200_at | FASTKD3 | FAST kinase domains 3 | other |  |  | 1,02E-08 | -1,575 |  |  |
| 219217_at | NARS2 | asparaginyl-tRNA synthetase 2, mitochondrial (putative) | other |  |  | 9,15E-09 | -1,580 |  |  |
| 219231_at | TGS1 | trimethylguanosine synthase homolog (S. cerevisiae) | transcription regulator | 2,70E-10 | -1,565 | 2,70E-10 | -1,837 | 2,70E-10 | -1,439 |
| 219241_x_at | SSH3 | slingshot homolog 3 (Drosophila) | phosphatase | 1,83E-14 | -2,758 | 1,83E-14 | -2,547 | 1,83E-14 | -1,540 |
| 219248_at | THUMPD2 | THUMP domain containing 2 | enzyme |  |  | 9,34E-08 | -1,685 |  |  |
| 219250_s_at | FLRT3 | fibronectin leucine rich transmembrane protein 3 | other | 5,20E-08 | -1,666 |  |  |  |  |
| 219260_s_at | C17ORF81 | chromosome 17 open reading frame 81 | other |  |  | 5,93E-12 | -1,757 | 5,93E-12 | -1,370 |
| 219275_at | PDCD5 | programmed cell death 5 | other |  |  | 4,02E-09 | -1,744 |  |  |
| 219283_at | C1GALT1C1 | C1GALT1-specific chaperone 1 | enzyme | 1,16E-10 | -1,580 | 1,16E-10 | -1,575 |  |  |
| 219290_x_at | DAPP1 | dual adaptor of phosphotyrosine and 3-phosphoinositides | other |  |  |  |  | 1,29E-08 | -1,970 |
| 219294_at | CENPQ | centromere protein Q | other |  |  | 9,35E-09 | -1,707 | 9,35E-09 | -1,390 |
| 219298_at | ECHDC3 | enoyl Coenzyme A hydratase domain containing 3 | enzyme |  |  | 2,35E-10 | -1,944 |  |  |
| 219305_x_at | FBXO2 | F-box protein 2 | enzyme |  |  | 1,83E-14 | -2,406 | 1,83E-14 | -1,749 |
| 219306_at | KIF15 | kinesin family member 15 | other | 1,63E-11 | -1,574 | 1,63E-11 | -1,770 | 1,63E-11 | -1,366 |
| 219320_at | MYO19 | myosin XIX | other |  |  | 1,79E-09 | -2,503 |  |  |
| 219321_at | MPP5 | membrane protein, palmitoylated 5 (MAGUK p55 subfamily member 5) | kinase |  |  | 2,84E-08 | -1,575 | 2,84E-08 | -1,533 |
| 219329_s_at | C2ORF28 | chromosome 2 open reading frame 28 | other | 2,40E-12 | -1,499 | 2,40E-12 | -1,791 | 2,40E-12 | -1,416 |
| 219357_at | GTPBP1 | GTP binding protein 1 | enzyme |  |  | 1,25E-07 | -2,399 |  |  |
| 219388_at | GRHL2 | grainyhead-like 2 (Drosophila) | transcription regulator |  |  | 1,17E-07 | -1,657 |  |  |
| 219401_at | XYLT2 | xylosyltransferase II | enzyme |  |  | 1,59E-07 | -1,950 |  |  |
| 219416_at | SCARA3 | scavenger receptor class A, member 3 | transmembrane receptor | 6,80E-10 | -2,093 | 6,80E-10 | -2,656 | 6,80E-10 | -1,891 |
| 219428_s_at | PXMP4 | peroxisomal membrane protein 4, 24kDa | other |  |  | 1,72E-11 | -1,850 | 1,72E-11 | -1,637 |
| 219465_at | APOA2 | apolipoprotein A-II | transporter |  |  | 7,12E-08 | -1,475 |  |  |
| 219470_x_at | CCNJ | cyclin J | other |  |  | 2,78E-08 | -1,969 |  |  |
| 219474_at | C3ORF52 | chromosome 3 open reading frame 52 | other | 2,04E-08 | -1,549 |  |  |  |  |
| 219485_s_at | PSMD10 | proteasome (prosome, macropain) 26S subunit, non-ATPase, 10 | transcription regulator | 1,01E-13 | -1,522 | 1,01E-13 | -1,687 | 1,01E-13 | -1,336 |
| 219489_s_at | NXN | nucleoredoxin | enzyme |  |  | 3,36E-08 | -1,522 | 3,36E-08 | -1,355 |
| 219492_at | CHIC2 | cysteine-rich hydrophobic domain 2 | other |  |  | 1,47E-10 | -1,781 | 1,47E-10 | -1,438 |
| 219512_at | DSN1 | DSN1, MIND kinetochore complex component, homolog (S. cerevisiae) | other | 3,39E-10 | -1,396 | 3,39E-10 | -1,678 | 3,39E-10 | -1,452 |
| 219522_at | FJX1 | four jointed box 1 (Drosophila) | other |  |  |  |  | 1,07E-12 | 2,309 |
| 219543_at | PBLD | phenazine biosynthesis-like protein domain containing | enzyme | 4,24E-10 | -1,566 | 4,24E-10 | -1,600 | 4,24E-10 | -1,638 |
| 219547_at | COX15 | COX15 homolog, cytochrome c oxidase assembly protein (yeast) | enzyme | 7,55E-14 | -1,704 | 7,55E-14 | -1,808 | 7,55E-14 | -1,457 |
| 219555_s_at | CENPN | centromere protein N | other |  |  | 1,72E-07 | -1,573 |  |  |
| 219588_s_at | NCAPG2 | non-SMC condensin II complex, subunit G2 | other | 7,55E-14 | -1,635 | 7,55E-14 | -2,176 | 7,55E-14 | -1,477 |
| 219598_s_at | RWDD1 (includes EG:51389) | RWD domain containing 1 | other |  |  | 1,23E-12 | -1,755 |  |  |
| 219612_s_at | FGG | fibrinogen gamma chain | other | 1,83E-14 | -2,021 | 1,83E-14 | -1,951 | 1,83E-14 | -3,733 |
| 219639_x_at | PARP6 | poly (ADP-ribose) polymerase family, member 6 | other |  |  | 1,00E-08 | -1,857 |  |  |
| 219648_at | MREG | melanoregulin | other | 1,83E-14 | -1,624 | 1,83E-14 | -2,132 |  |  |
| 219654_at | PTPLA | protein tyrosine phosphatase-like (proline instead of catalytic arginine), member A | phosphatase |  |  | 8,89E-10 | -1,801 |  |  |
| 219675_s_at | UXS1 | UDP-glucuronate decarboxylase 1 | enzyme |  |  | 5,40E-09 | -1,513 |  |  |
| 219703_at | MNS1 | meiosis-specific nuclear structural 1 | other |  |  | 1,92E-08 | -1,690 |  |  |
| 219762_s_at | RPL36 (includes EG:25873) | ribosomal protein L36 | other |  |  | 2,35E-09 | -1,570 |  |  |
| 219787_s_at | ECT2 | epithelial cell transforming sequence 2 oncogene | other | 3,01E-09 | -1,392 | 3,01E-09 | -1,533 |  |  |
| 219798_s_at | MEPCE | methylphosphate capping enzyme | enzyme |  |  | 3,02E-09 | -1,821 |  |  |
| 219806_s_at | C11ORF75 | chromosome 11 open reading frame 75 | other | 1,34E-10 | -1,534 | 1,34E-10 | -1,639 | 1,34E-10 | -1,331 |
| 219816_s_at | RBM23 | RNA binding motif protein 23 | other |  |  | 9,69E-08 | -1,687 |  |  |
| 219819_s_at | MRPS28 | mitochondrial ribosomal protein S28 | other |  |  | 4,19E-09 | -1,605 |  |  |
| 219911_s_at | SLCO4A1 | solute carrier organic anion transporter family, member 4A1 | transporter |  |  | 2,96E-09 | -1,650 |  |  |
| 219918_s_at | ASPM | asp (abnormal spindle) homolog, microcephaly associated (Drosophila) | other | 6,55E-08 | -1,379 |  |  |  |  |
| 219929_s_at | ZFYVE21 | zinc finger, FYVE domain containing 21 | other |  |  | 3,67E-10 | -2,021 |  |  |
| 219948_x_at | UGT2A3 | UDP glucuronosyltransferase 2 family, polypeptide A3 | enzyme |  |  |  |  | 1,39E-08 | -2,897 |
| 219979_s_at | C11ORF73 | chromosome 11 open reading frame 73 | other | 1,83E-14 | -1,328 | 1,83E-14 | -2,128 |  |  |
| 220155_s_at | BRD9 | bromodomain containing 9 | other |  |  | 2,54E-11 | -1,782 |  |  |
| 220189_s_at | MGAT4B | mannosyl (alpha-1,3-)-glycoprotein beta-1,4-N-acetylglucosaminyltransferase, isozyme B | enzyme |  |  | 1,83E-14 | -1,892 |  |  |
| 220199_s_at | AIDA | axin interactor, dorsalization associated | other | 8,86E-11 | -1,408 | 8,86E-11 | -1,657 | 8,86E-11 | -1,375 |
| 220235_s_at | C1ORF103 | chromosome 1 open reading frame 103 | other |  |  | 7,87E-12 | -1,706 | 7,87E-12 | -1,293 |
| 220238_s_at | KLHL7 | kelch-like 7 (Drosophila) | other | 1,62E-12 | -1,536 | 1,62E-12 | -1,959 | 1,62E-12 | -1,499 |
| 220239_at | KLHL7 | kelch-like 7 (Drosophila) | other | 2,25E-13 | -1,372 | 2,25E-13 | -1,809 | 2,25E-13 | -1,428 |
| 220248_x_at | NSFL1C | NSFL1 (p97) cofactor (p47) | other |  |  | 7,12E-10 | -1,611 |  |  |
| 220261_s_at | ZDHHC4 | zinc finger, DHHC-type containing 4 | other | 8,21E-12 | -1,594 | 8,21E-12 | -2,053 | 8,21E-12 | -1,387 |
| 220366_at | ELSPBP1 | epididymal sperm binding protein 1 | other | 5,71E-09 | -2,506 | 5,71E-09 | -3,671 |  |  |
| 220386_s_at | EML4 | echinoderm microtubule associated protein like 4 | other |  |  | 2,53E-09 | -1,625 |  |  |
| 220468_at | ARL14 | ADP-ribosylation factor-like 14 | other |  |  |  |  | 1,49E-08 | 1,527 |
| 220587_s_at | GBL | G protein beta subunit-like | other |  |  | 6,07E-12 | -2,299 |  |  |
| 220607_x_at | TH1L | TH1-like (Drosophila) | other |  |  | 1,82E-09 | -1,633 |  |  |
| 220643_s_at | FAIM | Fas apoptotic inhibitory molecule | other | 1,83E-14 | -1,636 | 1,83E-14 | -2,061 | 1,83E-14 | -1,556 |
| 220668_s_at | DNMT3B | DNA (cytosine-5-)-methyltransferase 3 beta | enzyme |  |  | 7,60E-09 | -1,651 | 7,60E-09 | -1,339 |
| 220755_s_at | C6ORF48 | chromosome 6 open reading frame 48 | other |  |  | 1,06E-07 | -1,589 |  |  |
| 220839_at | METTL5 | methyltransferase like 5 | enzyme |  |  |  |  | 1,31E-12 | 3,073 |
| 220864_s_at | NDUFA13 | NADH dehydrogenase (ubiquinone) 1 alpha subcomplex, 13 | enzyme |  |  | 1,65E-10 | -1,740 |  |  |
| 220890_s_at | DDX47 | DEAD (Asp-Glu-Ala-Asp) box polypeptide 47 | enzyme | 2,06E-13 | -1,466 | 2,06E-13 | -1,662 |  |  |
| 220924_s_at | SLC38A2 | solute carrier family 38, member 2 | transporter |  |  | 3,18E-10 | -1,499 |  |  |
| 220934_s_at | MGC3196 | hypothetical protein MGC3196 | other |  |  | 5,91E-10 | -1,655 |  |  |
| 220945_x_at | MANSC1 | MANSC domain containing 1 | other |  |  | 9,15E-07 | -1,453 |  |  |
| 220948_s_at | ATP1A1 | ATPase, Na+/K+ transporting, alpha 1 polypeptide | transporter |  |  | 2,30E-08 | -1,604 |  |  |
| 220951_s_at | A1CF | APOBEC1 complementation factor | enzyme | 9,09E-09 | -1,469 | 9,09E-09 | -1,785 |  |  |
| 221004_s_at | ITM2C | integral membrane protein 2C | other | 2,63E-12 | -1,614 | 2,63E-12 | -2,027 | 2,63E-12 | -1,651 |
| 221012_s_at | TRIM8 | tripartite motif-containing 8 | other |  |  | 8,02E-11 | -2,010 |  |  |
| 221020_s_at | SLC25A32 | solute carrier family 25, member 32 | transporter |  |  | 1,22E-08 | -1,581 |  |  |
| 221039_s_at | ASAP1 | ArfGAP with SH3 domain, ankyrin repeat and PH domain 1 | other |  |  | 1,48E-10 | -1,728 |  |  |
| 221058_s_at | CKLF | chemokine-like factor | cytokine | 3,31E-11 | -1,649 | 3,31E-11 | -2,022 |  |  |
| 221059_s_at | COTL1 | coactosin-like 1 (Dictyostelium) | other | 4,66E-11 | -1,378 | 4,66E-11 | -1,634 | 4,66E-11 | -1,393 |
| 221156_x_at | CCPG1 | cell cycle progression 1 | other | 1,96E-11 | -1,852 | 1,96E-11 | -2,111 |  |  |
| 221158_at | C21ORF66 | chromosome 21 open reading frame 66 | other |  |  | 2,69E-08 | -1,642 |  |  |
| 221196_x_at | BRCC3 | BRCA1/BRCA2-containing complex, subunit 3 | other | 1,83E-14 | -1,786 | 1,83E-14 | -1,991 | 1,83E-14 | -1,691 |
| 221218_s_at | TPK1 | thiamin pyrophosphokinase 1 | kinase | 1,83E-14 | -1,838 | 1,83E-14 | -1,817 | 1,83E-14 | -1,851 |
| 221263_s_at | SF3B5 | splicing factor 3b, subunit 5, 10kDa | other |  |  | 1,27E-07 | -1,593 |  |  |
| 221267_s_at | FAM108A1 | family with sequence similarity 108, member A1 | enzyme |  |  | 1,41E-10 | -2,012 |  |  |
| 221269_s_at | SH3BGRL3 | SH3 domain binding glutamic acid-rich protein like 3 | other |  |  | 3,22E-10 | -2,050 |  |  |
| 221311_x_at | LYRM2 | LYR motif containing 2 | other |  |  | 7,31E-08 | -1,533 |  |  |
| 221437_s_at | MRPS15 | mitochondrial ribosomal protein S15 | other | 6,14E-13 | -1,355 | 6,14E-13 | -1,904 | 6,14E-13 | -1,518 |
| 221452_s_at | TMEM14B | transmembrane protein 14B | other | 6,76E-13 | -1,354 | 6,76E-13 | -1,641 | 6,76E-13 | -1,423 |
| 221471_at | SERINC3 | serine incorporator 3 | transporter |  |  | 3,50E-10 | -1,668 |  |  |
| 221478_at | BNIP3L | BCL2/adenovirus E1B 19kDa interacting protein 3-like | other | 1,58E-12 | -1,614 | 1,58E-12 | -1,803 | 1,58E-12 | -1,512 |
| 221482_s_at | ARPP-19 | cyclic AMP phosphoprotein, 19 kD | transporter | 1,38E-10 | -1,387 | 1,38E-10 | -1,565 | 1,38E-10 | -1,364 |
| 221492_s_at | ATG3 | ATG3 autophagy related 3 homolog (S. cerevisiae) | enzyme |  |  | 2,64E-08 | -1,438 |  |  |
| 221493_at | TSPYL1 | TSPY-like 1 | other |  |  | 1,78E-08 | -1,517 | 1,78E-08 | -1,314 |
| 221500_s_at | STX16 | syntaxin 16 | transporter |  |  | 4,62E-09 | -1,637 |  |  |
| 221502_at | KPNA3 | karyopherin alpha 3 (importin alpha 4) | transporter | 2,04E-12 | -1,433 | 2,04E-12 | -1,679 |  |  |
| 221509_at | DENR (includes EG:8562) | density-regulated protein | other |  |  | 4,04E-10 | -1,660 |  |  |
| 221515_s_at | LCMT1 | leucine carboxyl methyltransferase 1 | enzyme |  |  | 4,21E-08 | -1,814 |  |  |
| 221519_at | FBXW4 | F-box and WD repeat domain containing 4 | other | 2,46E-10 | -1,711 | 2,46E-10 | -2,271 | 2,46E-10 | -1,655 |
| 221520_s_at | CDCA8 | cell division cycle associated 8 | other | 1,83E-14 | -1,581 | 1,83E-14 | -2,113 | 1,83E-14 | -1,777 |
| 221521_s_at | GINS2 | GINS complex subunit 2 (Psf2 homolog) | other |  |  | 8,09E-08 | -1,520 |  |  |
| 221531_at | WDR61 | WD repeat domain 61 | other |  |  | 8,48E-08 | -1,619 |  |  |
| 221532_s_at | WDR61 | WD repeat domain 61 | other | 1,07E-12 | -1,432 | 1,07E-12 | -1,676 |  |  |
| 221535_at | LSG1 | large subunit GTPase 1 homolog (S. cerevisiae) | other | 1,83E-14 | -1,583 | 1,83E-14 | -2,064 |  |  |
| 221536_s_at | LSG1 | large subunit GTPase 1 homolog (S. cerevisiae) | other | 2,43E-12 | -1,560 | 2,43E-12 | -1,968 |  |  |
| 221539_at | EIF4EBP1 | eukaryotic translation initiation factor 4E binding protein 1 | translation regulator |  |  | 3,18E-08 | -1,810 |  |  |
| 221543_s_at | ERLIN2 | ER lipid raft associated 2 | other | 5,49E-13 | -1,649 | 5,49E-13 | -1,701 | 5,49E-13 | -1,474 |
| 221550_at | COX15 | COX15 homolog, cytochrome c oxidase assembly protein (yeast) | enzyme |  |  |  |  | 1,71E-07 | -1,419 |
| 221555_x_at | CDC14C | CDC14 cell division cycle 14 homolog C (S. cerevisiae) | phosphatase |  |  |  |  | 4,55E-09 | 1,658 |
| 221570_s_at | METTL5 | methyltransferase like 5 | enzyme |  |  | 6,43E-09 | -1,504 |  |  |
| 221580_s_at | TAF1D | TATA box binding protein (TBP)-associated factor, RNA polymerase I, D, 41kDa | other |  |  | 6,93E-09 | -1,448 |  |  |
| 221589_s_at | ALDH6A1 | aldehyde dehydrogenase 6 family, member A1 | enzyme | 3,66E-10 | -1,603 | 3,66E-10 | -1,472 | 3,66E-10 | -1,722 |
| 221593_s_at | RPL31 | ribosomal protein L31 | other |  |  | 3,32E-08 | -1,511 |  |  |
| 221622_s_at | TMEM126B | transmembrane protein 126B | other |  |  | 2,35E-08 | -1,474 |  |  |
| 221665_s_at | EPS8L1 | EPS8-like 1 | other | 1,03E-06 | -1,929 |  |  |  |  |
| 221667_s_at | HSPB8 | heat shock 22kDa protein 8 | kinase | 6,86E-11 | -1,571 | 6,86E-11 | -1,713 | 6,86E-11 | -1,573 |
| 221669_s_at | ACAD8 | acyl-Coenzyme A dehydrogenase family, member 8 | enzyme | 1,01E-09 | -1,412 | 1,01E-09 | -1,604 |  |  |
| 221677_s_at | DONSON | downstream neighbor of SON | other | 4,43E-10 | -1,394 | 4,43E-10 | -1,509 | 4,43E-10 | -1,385 |
| 221685_s_at | CCDC99 | coiled-coil domain containing 99 | other | 2,15E-11 | -1,473 | 2,15E-11 | -1,603 | 2,15E-11 | -1,274 |
| 221692_s_at | MRPL34 | mitochondrial ribosomal protein L34 | other |  |  | 2,42E-08 | -1,748 |  |  |
| 221702_s_at | TM2D3 | TM2 domain containing 3 | other |  |  | 3,73E-09 | -1,627 |  |  |
| 221711_s_at | C19ORF62 | chromosome 19 open reading frame 62 | other | 1,29E-11 | -1,503 | 1,29E-11 | -1,917 |  |  |
| 221712_s_at | WDR74 | WD repeat domain 74 | other |  |  | 1,73E-12 | -2,063 |  |  |
| 221726_at | RPL22 | ribosomal protein L22 | other |  |  | 6,05E-08 | -1,516 |  |  |
| 221729_at | COL5A2 | collagen, type V, alpha 2 | other | 1,56E-12 | -1,579 | 1,56E-12 | -1,538 | 1,56E-12 | -1,620 |
| 221730_at | COL5A2 | collagen, type V, alpha 2 | other | 2,86E-08 | -1,576 |  |  |  |  |
| 221736_at | KIAA1219 | KIAA1219 | other | 2,90E-10 | -1,457 | 2,90E-10 | -1,688 |  |  |
| 221739_at | C19ORF10 | chromosome 19 open reading frame 10 | cytokine |  |  | 2,22E-10 | -1,849 |  |  |
| 221740_x_at | LRRC37A2 | leucine rich repeat containing 37, member A2 | other |  |  |  |  | 9,90E-09 | -1,647 |
| 221749_at | YTHDF3 | YTH domain family, member 3 | other |  |  | 5,94E-08 | -1,409 |  |  |
| 221759_at | G6PC3 | glucose 6 phosphatase, catalytic, 3 | phosphatase |  |  | 1,85E-09 | -2,653 |  |  |
| 221765_at | UGCG | UDP-glucose ceramide glucosyltransferase | enzyme |  |  |  |  | 6,13E-11 | 1,849 |
| 221776_s_at | BRD7 | bromodomain containing 7 | other | 1,83E-10 | -1,393 | 1,83E-10 | -1,610 |  |  |
| 221803_s_at | NRBF2 | nuclear receptor binding factor 2 | transcription regulator |  |  | 1,09E-09 | -1,736 |  |  |
| 221810_at | RAB15 | RAB15, member RAS onocogene family | enzyme | 2,32E-08 | -2,389 |  |  |  |  |
| 221817_at | DOLPP1 | dolichyl pyrophosphate phosphatase 1 | enzyme | 5,97E-13 | -1,693 | 5,97E-13 | -1,949 | 5,97E-13 | -1,469 |
| 221821_s_at | C12ORF41 | chromosome 12 open reading frame 41 | other | 1,73E-10 | -1,688 |  |  |  |  |
| 221841_s_at | KLF4 | Kruppel-like factor 4 (gut) | transcription regulator | 1,83E-14 | 1,920 |  |  | 1,83E-14 | 2,201 |
| 221847_at | LOC100129361 | hypothetical protein LOC100129361 | other |  |  | 1,63E-09 | -1,559 |  |  |
| 221873_at | ZNF143 | zinc finger protein 143 | transcription regulator |  |  |  |  | 8,54E-06 | -1,415 |
| 221875_x_at | HLA-F | major histocompatibility complex, class I, F | transmembrane receptor |  |  | 1,74E-13 | -2,390 |  |  |
| 221882_s_at | TMEM8 | transmembrane protein 8 (five membrane-spanning domains) | other | 1,83E-14 | -1,363 | 1,83E-14 | -2,002 | 1,83E-14 | -1,570 |
| 221919_at | HNRNPA1 | heterogeneous nuclear ribonucleoprotein A1 | other | 1,83E-14 | -2,925 | 1,83E-14 | -4,084 | 1,83E-14 | -3,848 |
| 221943_x_at | RPL38 (includes EG:6169) | ribosomal protein L38 | other |  |  | 7,26E-09 | 1,637 |  |  |
| 221952_x_at | TRMT5 | TRM5 tRNA methyltransferase 5 homolog (S. cerevisiae) | other | 1,83E-14 | -1,604 | 1,83E-14 | -1,610 | 1,83E-14 | -1,477 |
| 221965_at | MPHOSPH9 | M-phase phosphoprotein 9 | other |  |  | 3,06E-08 | -1,825 |  |  |
| 221972_s_at | SDF4 | stromal cell derived factor 4 | other |  |  | 2,72E-11 | -1,994 | 2,72E-11 | -1,387 |
| 221983_at | FAM134A | family with sequence similarity 134, member A | other | 2,53E-10 | -1,406 | 2,53E-10 | -1,610 | 2,53E-10 | -1,642 |
| 221984_s_at | FAM134A | family with sequence similarity 134, member A | other |  |  | 1,33E-09 | -1,581 | 1,33E-09 | -1,308 |
| 222024_s_at | AKAP13 | A kinase (PRKA) anchor protein 13 | other |  |  |  |  | 1,91E-08 | 2,051 |
| 222028_at | ZNF45 | zinc finger protein 45 | transcription regulator | 2,62E-10 | -1,641 | 2,62E-10 | -1,803 |  |  |
| 222047_s_at | ARS2 | arsenate resistance protein 2 | other |  |  | 1,57E-07 | -1,567 |  |  |
| 222056_s_at | FAHD2A | fumarylacetoacetate hydrolase domain containing 2A | enzyme | 1,00E-11 | -1,788 | 1,00E-11 | -2,247 |  |  |
| 222065_s_at | FLII | flightless I homolog (Drosophila) | other |  |  | 1,83E-14 | -2,097 |  |  |
| 222126_at | AGFG2 | ArfGAP with FG repeats 2 | other |  |  | 1,14E-09 | -2,173 | 1,14E-09 | -2,526 |
| 222146_s_at | TCF4 | transcription factor 4 | transcription regulator | 9,15E-09 | -1,753 |  |  | 9,15E-09 | -2,351 |
| 222154_s_at | LOC26010 | viral DNA polymerase-transactivated protein 6 | other |  |  | 1,08E-08 | -1,505 |  |  |
| 222155_s_at | GPR172A | G protein-coupled receptor 172A | other |  |  | 2,85E-11 | -2,403 |  |  |
| 222161_at | NAALAD2 | N-acetylated alpha-linked acidic dipeptidase 2 | peptidase | 1,83E-14 | -1,613 | 1,83E-14 | -1,584 | 1,83E-14 | -2,042 |
| 222190_s_at | C16ORF58 | chromosome 16 open reading frame 58 | other | 2,24E-12 | -2,190 | 2,24E-12 | -3,121 | 2,24E-12 | -2,620 |
| 222192_s_at | C2ORF43 | chromosome 2 open reading frame 43 | enzyme |  |  | 1,35E-07 | -1,862 |  |  |
| 222201_s_at | CASP8AP2 | caspase 8 associated protein 2 | transcription regulator |  |  | 2,68E-11 | -1,879 |  |  |
| 222203_s_at | RDH14 | retinol dehydrogenase 14 (all-trans/9-cis/11-cis) | enzyme | 7,21E-10 | -1,516 | 7,21E-10 | -1,630 | 7,21E-10 | -1,445 |
| 222209_s_at | TMEM135 | transmembrane protein 135 | other |  |  | 4,50E-13 | -1,555 |  |  |
| 222212_s_at | LASS2 | LAG1 homolog, ceramide synthase 2 | transcription regulator | 1,83E-14 | -1,535 | 1,83E-14 | -1,871 | 1,83E-14 | -1,423 |
| 222231_s_at | LRRC59 | leucine rich repeat containing 59 | other |  |  | 2,44E-08 | -1,495 |  |  |
| 222369_at | NAT11 | N-acetyltransferase 11 (GCN5-related, putative) | other |  |  | 1,49E-08 | -1,651 |  |  |
| 222385_x_at | SEC61A1 | Sec61 alpha 1 subunit (S. cerevisiae) | transporter |  |  | 9,79E-08 | -1,938 |  |  |
| 222386_s_at | COPZ1 | coatomer protein complex, subunit zeta 1 | transporter | 2,08E-10 | -1,404 | 2,08E-10 | -1,639 |  |  |
| 222388_s_at | VPS35 | vacuolar protein sorting 35 homolog (S. cerevisiae) | transporter |  |  | 1,33E-08 | -1,555 |  |  |
| 222389_s_at | WAC | WW domain containing adaptor with coiled-coil | other |  |  | 1,26E-08 | -1,544 |  |  |
| 222392_x_at | PERP | PERP, TP53 apoptosis effector | other |  |  | 5,00E-08 | -1,488 |  |  |
| 222395_s_at | UBE2Z | ubiquitin-conjugating enzyme E2Z | other |  |  |  |  | 1,09E-11 | 1,401 |
| 222396_at | HN1 | hematological and neurological expressed 1 | other | 1,36E-11 | -1,601 | 1,36E-11 | -1,894 | 1,36E-11 | -1,463 |
| 222398_s_at | EFTUD2 | elongation factor Tu GTP binding domain containing 2 | enzyme |  |  | 5,30E-12 | -1,908 |  |  |
| 222403_at | MTCH2 | mitochondrial carrier homolog 2 (C. elegans) | other |  |  | 1,46E-08 | -1,550 | 1,46E-08 | -1,385 |
| 222410_s_at | SNX6 | sorting nexin 6 | transporter |  |  | 1,35E-07 | -1,426 |  |  |
| 222417_s_at | SNX5 | sorting nexin 5 | transporter |  |  | 1,79E-07 | -1,501 |  |  |
| 222423_at | NDFIP1 | Nedd4 family interacting protein 1 | other |  |  | 2,79E-09 | -1,562 |  |  |
| 222425_s_at | POLDIP2 | polymerase (DNA-directed), delta interacting protein 2 | other |  |  | 4,85E-11 | -1,819 |  |  |
| 222438_at | MED4 | mediator complex subunit 4 | transcription regulator | 1,98E-13 | -1,906 | 1,98E-13 | -2,139 |  |  |
| 222439_s_at | THRAP3 | thyroid hormone receptor associated protein 3 | transcription regulator | 1,83E-14 | 2,104 | 1,83E-14 | 2,360 | 1,83E-14 | 2,806 |
| 222442_s_at | ARL8B | ADP-ribosylation factor-like 8B | enzyme |  |  | 1,46E-09 | -1,624 | 1,46E-09 | -1,372 |
| 222443_s_at | RBM8A | RNA binding motif protein 8A | other |  |  | 6,03E-10 | -1,752 |  |  |
| 222447_at | METTL9 | methyltransferase like 9 | other | 1,13E-11 | -1,681 | 1,13E-11 | -2,109 | 1,13E-11 | -1,566 |
| 222448_s_at | CMPK1 | cytidine monophosphate (UMP-CMP) kinase 1, cytosolic | kinase |  |  | 5,62E-10 | -1,808 | 5,62E-10 | -1,426 |
| 222449_at | PMEPA1 | prostate transmembrane protein, androgen induced 1 | other |  |  |  |  | 2,86E-08 | 1,860 |
| 222464_s_at | C10ORF119 | chromosome 10 open reading frame 119 | other |  |  | 1,04E-10 | -1,618 |  |  |
| 222465_at | C15ORF15 | chromosome 15 open reading frame 15 | other |  |  | 4,29E-09 | -1,642 |  |  |
| 222467_s_at | SAPS3 | SAPS domain family, member 3 | other | 8,73E-13 | -1,473 | 8,73E-13 | -1,615 |  |  |
| 222477_s_at | TM7SF3 | transmembrane 7 superfamily member 3 | other |  |  | 1,56E-08 | -1,621 |  |  |
| 222488_s_at | DCTN4 | dynactin 4 (p62) | other | 1,61E-08 | -1,421 | 1,61E-08 | -1,508 |  |  |
| 222495_at | TMEM167B | transmembrane protein 167B | other |  |  | 4,64E-08 | -1,730 |  |  |
| 222496_s_at | RBM47 | RNA binding motif protein 47 | other |  |  | 1,36E-08 | -1,708 |  |  |
| 222499_at | MRPS16 | mitochondrial ribosomal protein S16 | other | 2,24E-12 | -1,464 | 2,24E-12 | -2,004 | 2,24E-12 | -1,680 |
| 222504_s_at | COX4NB | COX4 neighbor | other |  |  | 2,99E-13 | -2,373 |  |  |
| 222514_at | RRAGC | Ras-related GTP binding C | enzyme |  |  | 2,00E-08 | -1,949 |  |  |
| 222516_at | AP3M1 | adaptor-related protein complex 3, mu 1 subunit | transporter |  |  | 3,75E-08 | -1,592 |  |  |
| 222527_s_at | RBM22 | RNA binding motif protein 22 | other |  |  | 1,66E-08 | -1,710 |  |  |
| 222533_at | CRBN | cereblon | enzyme |  |  | 1,29E-09 | -1,701 |  |  |
| 222549_at | CLDN1 | claudin 1 | other | 3,52E-11 | -1,860 |  |  |  |  |
| 222553_x_at | OXR1 | oxidation resistance 1 | other | 3,81E-10 | -1,635 | 3,81E-10 | -1,809 |  |  |
| 222557_at | STMN3 | stathmin-like 3 | other | 4,17E-12 | -1,598 | 4,17E-12 | -1,932 | 4,17E-12 | -1,496 |
| 222592_s_at | ACSL5 | acyl-CoA synthetase long-chain family member 5 | enzyme | 1,83E-14 | 1,775 |  |  | 1,83E-14 | 2,319 |
| 222605_at | RCOR3 (includes EG:55758) | REST corepressor 3 | other | 6,45E-10 | -1,582 | 6,45E-10 | -1,697 |  |  |
| 222607_s_at | DIS3 | DIS3 mitotic control homolog (S. cerevisiae) | enzyme |  |  | 6,93E-09 | -1,567 |  |  |
| 222608_s_at | ANLN | anillin, actin binding protein | other | 8,89E-10 | -1,438 | 8,89E-10 | -1,478 |  |  |
| 222609_s_at | EXOSC1 | exosome component 1 | enzyme |  |  | 1,47E-07 | -1,615 |  |  |
| 222634_s_at | TBL1XR1 | transducin (beta)-like 1 X-linked receptor 1 | transcription regulator |  |  |  |  | 2,49E-09 | 2,114 |
| 222637_at | COMMD10 | COMM domain containing 10 | other | 3,31E-09 | -1,389 | 3,31E-09 | -1,517 | 3,31E-09 | -1,341 |
| 222642_s_at | TMEM33 | transmembrane protein 33 | other | 2,90E-12 | -1,494 | 2,90E-12 | -1,832 |  |  |
| 222645_s_at | KCTD5 | potassium channel tetramerisation domain containing 5 | ion channel |  |  | 2,14E-08 | -2,341 |  |  |
| 222650_s_at | SLC2A4RG | SLC2A4 regulator | transcription regulator | 1,98E-08 | -1,940 |  |  | 1,98E-08 | -2,017 |
| 222653_at | PNPO | pyridoxamine 5'-phosphate oxidase | enzyme |  |  | 5,05E-09 | -1,795 |  |  |
| 222691_at | SLC35B3 | solute carrier family 35, member B3 | other | 1,48E-09 | -1,622 | 1,48E-09 | -1,493 | 1,48E-09 | -1,418 |
| 222696_at | AXIN2 | axin 2 | other | 1,83E-14 | -1,547 | 1,83E-14 | -1,852 | 1,83E-14 | -1,870 |
| 222714_s_at | LACTB2 | lactamase, beta 2 | other | 1,83E-14 | -1,428 | 1,83E-14 | -1,789 | 1,83E-14 | -1,556 |
| 222741_s_at | C6ORF64 | chromosome 6 open reading frame 64 | other | 1,14E-09 | -1,591 | 1,14E-09 | -1,823 | 1,14E-09 | -1,500 |
| 222756_s_at | ARRB1 | arrestin, beta 1 | other |  |  | 2,29E-08 | -2,556 |  |  |
| 222759_at | SUV420H1 | suppressor of variegation 4-20 homolog 1 (Drosophila) | enzyme |  |  | 1,34E-07 | -1,582 |  |  |
| 222770_s_at | GUF1 | GUF1 GTPase homolog (S. cerevisiae) | other |  |  | 2,79E-09 | -1,715 |  |  |
| 222774_s_at | NETO2 | neuropilin (NRP) and tolloid (TLL)-like 2 | other |  |  | 3,52E-08 | -1,730 |  |  |
| 222787_s_at | TMEM106B | transmembrane protein 106B | other | 2,46E-10 | 1,638 |  |  | 2,46E-10 | 1,801 |
| 222792_s_at | CCDC59 | coiled-coil domain containing 59 | other |  |  | 8,32E-09 | -1,570 |  |  |
| 222808_at | ALG13 (includes EG:79868) | asparagine-linked glycosylation 13 homolog (S. cerevisiae) | other |  |  |  |  | 8,97E-08 | -1,413 |
| 222821_s_at | GEMIN7 | gem (nuclear organelle) associated protein 7 | other |  |  | 2,01E-08 | -1,853 |  |  |
| 222826_at | PLDN | pallidin homolog (mouse) | other | 1,83E-14 | -1,776 | 1,83E-14 | -1,894 | 1,83E-14 | -1,491 |
| 222832_s_at | MFF | mitochondrial fission factor | other | 1,29E-09 | -1,381 | 1,29E-09 | -1,560 | 1,29E-09 | -1,287 |
| 222843_at | FIGNL1 | fidgetin-like 1 | other | 2,15E-08 | -1,363 | 2,15E-08 | -1,450 |  |  |
| 222849_s_at | SCRN3 | secernin 3 | other | 6,25E-11 | -1,436 | 6,25E-11 | -1,642 | 6,25E-11 | -1,379 |
| 222875_at | DHX33 | DEAH (Asp-Glu-Ala-His) box polypeptide 33 | enzyme | 4,22E-13 | -1,552 | 4,22E-13 | -1,973 | 4,22E-13 | -1,346 |
| 222894_x_at | C20ORF7 | chromosome 20 open reading frame 7 | other | 1,04E-09 | -1,676 | 1,04E-09 | -1,917 |  |  |
| 222912_at | ARRB1 | arrestin, beta 1 | other |  |  |  |  | 1,17E-07 | -1,549 |
| 222925_at | DCDC2 | doublecortin domain containing 2 | other | 4,96E-08 | -1,382 |  |  |  |  |
| 222983_s_at | PAIP2 | poly(A) binding protein interacting protein 2 | translation regulator |  |  | 2,16E-09 | -1,605 | 2,16E-09 | -1,318 |
| 222986_s_at | SHISA5 | shisa homolog 5 (Xenopus laevis) | other |  |  | 1,21E-10 | -1,759 | 1,21E-10 | -1,371 |
| 222987_s_at | TMEM9 | transmembrane protein 9 | other | 1,83E-14 | -1,715 | 1,83E-14 | -2,193 | 1,83E-14 | -1,662 |
| 222988_s_at | TMEM9 | transmembrane protein 9 | other |  |  | 1,83E-14 | -2,061 | 1,83E-14 | -1,329 |
| 222990_at | UBQLN1 | ubiquilin 1 | other |  |  | 8,47E-08 | -1,564 |  |  |
| 222993_at | MRPL37 | mitochondrial ribosomal protein L37 | enzyme |  |  | 2,96E-11 | -1,871 |  |  |
| 222994_at | PRDX5 | peroxiredoxin 5 | enzyme | 1,83E-14 | -1,549 | 1,83E-14 | -1,984 | 1,83E-14 | -1,527 |
| 222997_s_at | C1ORF51 | chromosome 1 open reading frame 51 | other |  |  | 1,30E-08 | -1,746 |  |  |
| 223001_at | DC2 | oligosaccharyltransferase complex subunit | enzyme |  |  | 6,72E-08 | -1,538 |  |  |
| 223003_at | C19ORF43 | chromosome 19 open reading frame 43 | other |  |  | 1,34E-12 | -1,778 |  |  |
| 223007_s_at | C9ORF5 | chromosome 9 open reading frame 5 | other | 6,48E-11 | -1,290 | 6,48E-11 | -1,671 |  |  |
| 223008_s_at | C9ORF5 | chromosome 9 open reading frame 5 | other | 7,36E-11 | -1,429 | 7,36E-11 | -1,604 |  |  |
| 223009_at | C11ORF59 | chromosome 11 open reading frame 59 | other |  |  | 1,63E-13 | -1,936 |  |  |
| 223011_s_at | OCIAD1 | OCIA domain containing 1 | other | 3,38E-13 | -1,592 | 3,38E-13 | -1,818 | 3,38E-13 | -1,406 |
| 223012_at | UBXN6 | UBX domain protein 6 | other |  |  | 1,15E-08 | -2,159 |  |  |
| 223014_at | UBE2R2 (includes EG:54926) | ubiquitin-conjugating enzyme E2R 2 | enzyme |  |  | 6,88E-11 | -1,816 |  |  |
| 223021_x_at | VTA1 | Vps20-associated 1 homolog (S. cerevisiae) | other | 9,18E-10 | -1,375 | 9,18E-10 | -1,540 | 9,18E-10 | -1,281 |
| 223022_s_at | VTA1 | Vps20-associated 1 homolog (S. cerevisiae) | other |  |  | 1,82E-09 | -1,661 |  |  |
| 223023_at | BET1L | blocked early in transport 1 homolog (S. cerevisiae)-like | transporter | 2,90E-08 | -1,735 |  |  |  |  |
| 223024_at | AP1M1 | adaptor-related protein complex 1, mu 1 subunit | transporter |  |  | 1,62E-10 | -1,909 | 1,62E-10 | -1,384 |
| 223026_s_at | VPS29 | vacuolar protein sorting 29 homolog (S. cerevisiae) | transporter |  |  | 9,07E-08 | -1,479 |  |  |
| 223028_s_at | SNX9 (includes EG:51429) | sorting nexin 9 | transporter |  |  | 1,95E-10 | -1,801 |  |  |
| 223032_x_at | PRELID1 | PRELI domain containing 1 | other |  |  | 8,57E-11 | -1,634 |  |  |
| 223037_at | PDZD11 | PDZ domain containing 11 | other |  |  | 3,93E-09 | -1,581 | 3,93E-09 | -1,306 |
| 223040_at | NAT5 | N-acetyltransferase 5 (GCN5-related, putative) | enzyme |  |  | 2,58E-08 | -1,525 |  |  |
| 223042_s_at | FUNDC2 (includes EG:65991) | FUN14 domain containing 2 | other | 1,83E-14 | -1,497 | 1,83E-14 | -1,783 | 1,83E-14 | -1,397 |
| 223043_at | TMEM85 | transmembrane protein 85 | other |  |  | 4,26E-09 | -1,685 |  |  |
| 223044_at | SLC40A1 | solute carrier family 40 (iron-regulated transporter), member 1 | transporter | 1,23E-12 | -1,346 | 1,23E-12 | -1,453 | 1,23E-12 | -1,679 |
| 223046_at | EGLN1 | egl nine homolog 1 (C. elegans) | enzyme | 6,26E-14 | -1,592 | 6,26E-14 | -1,651 | 6,26E-14 | -1,772 |
| 223047_at | CMTM6 | CKLF-like MARVEL transmembrane domain containing 6 | cytokine | 2,34E-10 | -1,536 | 2,34E-10 | -1,611 |  |  |
| 223055_s_at | XPO5 | exportin 5 | transporter | 1,87E-10 | -1,929 | 1,87E-10 | -2,209 | 1,87E-10 | -1,623 |
| 223062_s_at | PSAT1 | phosphoserine aminotransferase 1 | enzyme |  |  | 7,97E-10 | -1,554 |  |  |
| 223064_at | RNF181 | ring finger protein 181 | other |  |  | 2,09E-09 | -1,604 |  |  |
| 223065_s_at | STARD3NL | STARD3 N-terminal like | other | 2,78E-13 | -1,420 | 2,78E-13 | -1,775 |  |  |
| 223070_at | SELK | selenoprotein K | other |  |  | 1,58E-09 | -1,633 |  |  |
| 223071_at | IER3IP1 | immediate early response 3 interacting protein 1 | other |  |  | 1,89E-08 | -1,519 |  |  |
| 223073_at | HIATL1 | hippocampus abundant transcript-like 1 | other |  |  | 1,20E-10 | -1,678 |  |  |
| 223077_at | TMOD3 | tropomodulin 3 (ubiquitous) | other |  |  | 3,50E-08 | -1,542 |  |  |
| 223082_at | SH3KBP1 | SH3-domain kinase binding protein 1 | other | 1,53E-11 | -1,571 | 1,53E-11 | -1,865 |  |  |
| 223084_s_at | CCNDBP1 | cyclin D-type binding-protein 1 | other | 2,84E-08 | -1,495 |  |  |  |  |
| 223085_at | RNF19A | ring finger protein 19A | other | 1,42E-11 | -1,932 | 1,42E-11 | -1,669 | 1,42E-11 | -1,466 |
| 223103_at | STARD10 | StAR-related lipid transfer (START) domain containing 10 | other | 8,88E-14 | -1,439 | 8,88E-14 | -2,104 | 8,88E-14 | -1,483 |
| 223104_at | JAGN1 | jagunal homolog 1 (Drosophila) | other |  |  | 3,05E-11 | -1,785 |  |  |
| 223105_s_at | TMEM14C | transmembrane protein 14C | other |  |  | 4,97E-09 | -1,490 |  |  |
| 223106_at | TMEM14C | transmembrane protein 14C | other | 2,81E-10 | -1,308 | 2,81E-10 | -1,575 |  |  |
| 223107_s_at | ZCCHC17 | zinc finger, CCHC domain containing 17 | other | 4,87E-14 | -1,607 | 4,87E-14 | -2,253 | 4,87E-14 | -1,496 |
| 223112_s_at | NDUFB10 (includes EG:4716) | NADH dehydrogenase (ubiquinone) 1 beta subcomplex, 10, 22kDa | enzyme | 2,06E-13 | -1,332 | 2,06E-13 | -1,890 |  |  |
| 223113_at | TMEM138 | transmembrane protein 138 | other |  |  | 1,58E-10 | -1,819 |  |  |
| 223117_s_at | USP47 | ubiquitin specific peptidase 47 | peptidase |  |  | 4,30E-08 | -1,606 |  |  |
| 223128_at | FOXRED1 | FAD-dependent oxidoreductase domain containing 1 | other |  |  | 2,60E-08 | -1,892 |  |  |
| 223133_at | TMEM14B | transmembrane protein 14B | other |  |  | 8,84E-09 | -1,640 |  |  |
| 223136_at | AIG1 | androgen-induced 1 | other |  |  | 1,10E-08 | -1,752 |  |  |
| 223146_at | WDR33 | WD repeat domain 33 | other |  |  | 6,69E-09 | -3,869 |  |  |
| 223151_at | DCUN1D5 | DCN1, defective in cullin neddylation 1, domain containing 5 (S. cerevisiae) | other |  |  | 3,90E-12 | -1,622 |  |  |
| 223158_s_at | NEK6 | NIMA (never in mitosis gene a)-related kinase 6 | kinase |  |  | 8,20E-09 | -1,765 | 8,20E-09 | -1,386 |
| 223170_at | TMEM98 | transmembrane protein 98 | other | 1,51E-12 | -1,445 | 1,51E-12 | -1,770 | 1,51E-12 | -1,500 |
| 223171_at | DYM | dymeclin | other |  |  | 1,89E-08 | -1,880 |  |  |
| 223177_at | NT5DC1 | 5'-nucleotidase domain containing 1 | other |  |  | 2,01E-08 | -1,645 |  |  |
| 223186_at | UBE2V1 | ubiquitin-conjugating enzyme E2 variant 1 | transcription regulator |  |  | 1,83E-14 | -2,246 |  |  |
| 223194_s_at | SLC22A23 | solute carrier family 22, member 23 | transporter | 2,12E-07 | -1,613 |  |  |  |  |
| 223200_s_at | LSG1 | large subunit GTPase 1 homolog (S. cerevisiae) | other |  |  |  |  | 6,99E-08 | 1,694 |
| 223201_s_at | TMEM164 | transmembrane protein 164 | other |  |  | 3,23E-08 | -1,837 |  |  |
| 223206_s_at | NMRAL1 | NmrA-like family domain containing 1 | other |  |  | 5,18E-09 | -1,779 |  |  |
| 223207_x_at | PHPT1 | phosphohistidine phosphatase 1 | phosphatase |  |  | 1,22E-11 | -1,868 |  |  |
| 223209_s_at | SELS | selenoprotein S | other |  |  | 1,23E-10 | -1,523 |  |  |
| 223212_at | ZDHHC16 | zinc finger, DHHC-type containing 16 | other |  |  | 8,87E-10 | -1,629 | 8,87E-10 | -1,357 |
| 223218_s_at | NFKBIZ | nuclear factor of kappa light polypeptide gene enhancer in B-cells inhibitor, zeta | transcription regulator | 4,62E-12 | -1,350 | 4,62E-12 | -1,668 | 4,62E-12 | -1,530 |
| 223221_at | SCO1 | SCO cytochrome oxidase deficient homolog 1 (yeast) | other |  |  | 1,68E-10 | -2,054 |  |  |
| 223229_at | UBE2T | ubiquitin-conjugating enzyme E2T (putative) | enzyme |  |  | 5,73E-10 | -1,665 |  |  |
| 223231_at | TATDN1 (includes EG:83940) | TatD DNase domain containing 1 | other |  |  | 1,55E-07 | -1,538 |  |  |
| 223233_s_at | CGN | cingulin | other | 6,50E-11 | -1,617 | 6,50E-11 | -1,860 | 6,50E-11 | -1,459 |
| 223238_s_at | PBRM1 | polybromo 1 | other |  |  | 7,06E-08 | -1,567 |  |  |
| 223242_s_at | MFSD11 | major facilitator superfamily domain containing 11 | other | 4,88E-12 | -1,480 | 4,88E-12 | -1,854 | 4,88E-12 | -1,351 |
| 223255_at | KIAA1333 | KIAA1333 | other | 7,91E-13 | -1,859 | 7,91E-13 | -2,044 |  |  |
| 223259_at | ORMDL3 | ORM1-like 3 (S. cerevisiae) | other | 7,64E-13 | -1,881 | 7,64E-13 | -2,735 |  |  |
| 223264_at | MESDC1 | mesoderm development candidate 1 | other |  |  | 3,00E-11 | -1,949 |  |  |
| 223269_at | POLR3GL | polymerase (RNA) III (DNA directed) polypeptide G (32kD)-like | other |  |  |  |  | 1,67E-08 | -1,615 |
| 223271_s_at | CTDSPL2 | CTD (carboxy-terminal domain, RNA polymerase II, polypeptide A) small phosphatase like 2 | other |  |  | 6,28E-09 | -1,651 |  |  |
| 223272_s_at | C1ORF57 | chromosome 1 open reading frame 57 | other | 1,90E-09 | -1,714 | 1,90E-09 | -1,603 |  |  |
| 223273_at | C14ORF142 | chromosome 14 open reading frame 142 | other | 8,97E-13 | -1,383 | 8,97E-13 | -1,778 | 8,97E-13 | -1,830 |
| 223274_at | TCF19 | transcription factor 19 (SC1) | transcription regulator | 2,41E-09 | -1,673 | 2,41E-09 | -1,718 | 2,41E-09 | -1,617 |
| 223277_at | TMEM103 | chromosome 3 open reading frame 75 | other |  |  | 1,88E-09 | -2,149 |  |  |
| 223287_s_at | FOXP1 | forkhead box P1 | transcription regulator |  |  | 1,77E-07 | -1,434 |  |  |
| 223289_s_at | USP38 | ubiquitin specific peptidase 38 | peptidase |  |  | 1,59E-10 | -1,720 |  |  |
| 223299_at | SEC11C | SEC11 homolog C (S. cerevisiae) | peptidase |  |  | 4,57E-08 | -1,809 |  |  |
| 223308_s_at | WDR5 | WD repeat domain 5 | other |  |  | 1,53E-11 | -2,176 |  |  |
| 223310_x_at | PNPLA8 | patatin-like phospholipase domain containing 8 | enzyme |  |  |  |  | 6,60E-08 | -1,350 |
| 223315_at | NTN4 | netrin 4 | other | 1,83E-14 | -2,143 | 1,83E-14 | -1,748 | 1,83E-14 | -2,048 |
| 223318_s_at | ALKBH7 | alkB, alkylation repair homolog 7 (E. coli) | other |  |  | 5,31E-09 | -1,942 |  |  |
| 223331_s_at | DDX20 | DEAD (Asp-Glu-Ala-Asp) box polypeptide 20 | transcription regulator |  |  | 2,32E-07 | -1,634 |  |  |
| 223337_at | SDCCAG10 | serologically defined colon cancer antigen 10 | enzyme |  |  | 1,84E-08 | -1,559 |  |  |
| 223339_at | ATPIF1 | ATPase inhibitory factor 1 | other | 1,84E-09 | -1,555 | 1,84E-09 | -1,841 | 1,84E-09 | -1,648 |
| 223347_at | MUM1 | melanoma associated antigen (mutated) 1 | other |  |  | 9,02E-11 | -2,620 |  |  |
| 223351_at | C17ORF80 | chromosome 17 open reading frame 80 | other |  |  | 9,80E-11 | -1,935 |  |  |
| 223356_s_at | MTIF3 | mitochondrial translational initiation factor 3 | translation regulator | 3,20E-13 | -1,682 | 3,20E-13 | -2,045 |  |  |
| 223363_at | PSMG3 | proteasome (prosome, macropain) assembly chaperone 3 | other |  |  | 1,83E-08 | -1,823 |  |  |
| 223376_s_at | BRI3 | brain protein I3 | other | 1,83E-14 | -1,459 | 1,83E-14 | -1,959 | 1,83E-14 | -1,444 |
| 223378_at | GLIS2 | GLIS family zinc finger 2 | transcription regulator |  |  |  |  | 4,06E-08 | -1,828 |
| 223381_at | NUF2 | NUF2, NDC80 kinetochore complex component, homolog (S. cerevisiae) | other | 1,46E-12 | -1,553 | 1,46E-12 | -1,691 | 1,46E-12 | -1,618 |
| 223382_s_at | ZNRF1 | zinc and ring finger 1 | other |  |  | 1,15E-08 | -1,904 |  |  |
| 223397_s_at | NIP7 | nuclear import 7 homolog (S. cerevisiae) | other |  |  | 3,30E-07 | -1,740 |  |  |
| 223411_at | MIF4GD | MIF4G domain containing | other |  |  | 2,65E-09 | -1,757 |  |  |
| 223414_s_at | LYAR | Ly1 antibody reactive homolog (mouse) | other |  |  | 6,05E-10 | -1,661 |  |  |
| 223423_at | GPR160 | G protein-coupled receptor 160 | G-protein coupled receptor | 1,83E-14 | -1,376 | 1,83E-14 | -1,698 | 1,83E-14 | -1,657 |
| 223440_at | C16ORF70 | chromosome 16 open reading frame 70 | other |  |  | 3,49E-08 | -1,739 |  |  |
| 223441_at | SLC17A5 | solute carrier family 17 (anion/sugar transporter), member 5 | transporter |  |  | 1,01E-13 | -1,970 |  |  |
| 223443_s_at | FLJ32065 | hypothetical protein FLJ32065 | other | 3,83E-08 | -1,491 |  |  |  |  |
| 223447_at | REG4 | regenerating islet-derived family, member 4 | other |  |  | 1,88E-07 | -2,152 |  |  |
| 223450_s_at | COG3 | component of oligomeric golgi complex 3 | other |  |  | 7,96E-06 | -1,436 |  |  |
| 223452_s_at | ATL3 | atlastin GTPase 3 | other |  |  | 3,31E-09 | -1,753 |  |  |
| 223454_at | CXCL16 | chemokine (C-X-C motif) ligand 16 | cytokine | 6,86E-10 | -1,522 | 6,86E-10 | -1,612 | 6,86E-10 | -1,432 |
| 223479_s_at | CHCHD5 | coiled-coil-helix-coiled-coil-helix domain containing 5 | other |  |  | 2,50E-09 | -1,826 | 2,50E-09 | -1,459 |
| 223482_at | TMEM120A | transmembrane protein 120A | other |  |  | 6,67E-10 | -1,901 |  |  |
| 223484_at | C15ORF48 | chromosome 15 open reading frame 48 | other | 3,81E-08 | -1,427 | 3,81E-08 | -1,650 |  |  |
| 223491_at | COMMD2 | COMM domain containing 2 | other |  |  | 2,74E-08 | -1,679 |  |  |
| 223506_at | ZC3H8 | zinc finger CCCH-type containing 8 | other | 2,55E-09 | -1,561 | 2,55E-09 | -1,763 |  |  |
| 223507_at | CLPX | ClpX caseinolytic peptidase X homolog (E. coli) | enzyme |  |  | 1,53E-07 | -1,735 |  |  |
| 223509_at | CLDN2 | claudin 2 | other | 1,83E-14 | 1,781 |  |  | 1,83E-14 | 2,394 |
| 223513_at | CENPJ | centromere protein J | transcription regulator |  |  | 1,29E-09 | -1,737 |  |  |
| 223515_s_at | COQ3 | coenzyme Q3 homolog, methyltransferase (S. cerevisiae) | enzyme |  |  | 3,81E-08 | -1,799 |  |  |
| 223530_at | TDRKH | tudor and KH domain containing | other |  |  | 9,90E-11 | -2,060 | 9,90E-11 | -1,546 |
| 223532_at | ANKRD39 | ankyrin repeat domain 39 | other |  |  | 1,07E-07 | -1,739 |  |  |
| 223556_at | HELLS | helicase, lymphoid-specific | enzyme | 1,50E-08 | -1,384 | 1,50E-08 | -1,475 |  |  |
| 223559_s_at | C9ORF80 | chromosome 9 open reading frame 80 | other |  |  | 1,41E-08 | -1,541 |  |  |
| 223577_x_at | MALAT1 | metastasis associated lung adenocarcinoma transcript 1 (non-protein coding) | other | 1,83E-14 | -1,357 | 1,83E-14 | -2,277 | 1,83E-14 | -2,884 |
| 223580_at | SPSB2 | splA/ryanodine receptor domain and SOCS box containing 2 | other | 1,08E-12 | -2,068 | 1,08E-12 | -2,683 | 1,08E-12 | -2,009 |
| 223598_at | RAD23B | RAD23 homolog B (S. cerevisiae) | other |  |  | 5,14E-09 | -1,620 |  |  |
| 223608_at | EFCAB2 | EF-hand calcium binding domain 2 | other |  |  | 7,60E-09 | -1,793 |  |  |
| 223611_s_at | LNX1 | ligand of numb-protein X 1 | enzyme |  |  | 3,41E-08 | -1,546 | 3,41E-08 | -1,694 |
| 223631_s_at | C19ORF33 | chromosome 19 open reading frame 33 | other |  |  | 6,17E-10 | -1,758 |  |  |
| 223639_s_at | ZNRD1 | zinc ribbon domain containing 1 | transcription regulator |  |  | 1,75E-10 | -1,915 |  |  |
| 223649_s_at | SLC25A39 | solute carrier family 25, member 39 | other |  |  | 8,38E-11 | -2,029 |  |  |
| 223650_s_at | NRBF2 | nuclear receptor binding factor 2 | transcription regulator | 1,60E-12 | -1,488 | 1,60E-12 | -1,859 |  |  |
| 223677_at | ATG10 | ATG10 autophagy related 10 homolog (S. cerevisiae) | other |  |  | 2,43E-07 | -2,169 |  |  |
| 223686_at | TPK1 | thiamin pyrophosphokinase 1 | kinase | 1,83E-14 | -1,752 | 1,83E-14 | -1,998 | 1,83E-14 | -1,764 |
| 223700_at | MND1 | meiotic nuclear divisions 1 homolog (S. cerevisiae) | other | 2,95E-10 | -1,427 | 2,95E-10 | -1,673 |  |  |
| 223705_s_at | GPBP1 | GC-rich promoter binding protein 1 | transcription regulator | 1,83E-14 | -1,494 | 1,83E-14 | -1,618 |  |  |
| 223716_s_at | ZRANB2 | zinc finger, RAN-binding domain containing 2 | transcription regulator |  |  | 1,60E-08 | -1,444 |  |  |
| 223773_s_at | SNHG12 | small nucleolar RNA host gene 12 (non-protein coding) | other | 4,03E-12 | -1,514 | 4,03E-12 | -2,022 |  |  |
| 223785_at | FANCI | Fanconi anemia, complementation group I | other |  |  | 3,25E-10 | -1,889 | 3,25E-10 | -1,470 |
| 223804_s_at | THUMPD3 | THUMP domain containing 3 | other |  |  | 1,49E-09 | -1,857 |  |  |
| 223811_s_at | C7ORF20 | chromosome 7 open reading frame 20 | other |  |  | 6,11E-08 | -1,925 |  |  |
| 223819_x_at | COMMD5 | COMM domain containing 5 | other |  |  | 1,41E-09 | -2,416 |  |  |
| 223894_s_at | AKTIP | AKT interacting protein | other | 2,84E-09 | -1,526 | 2,84E-09 | -1,802 |  |  |
| 223940_x_at | MALAT1 | metastasis associated lung adenocarcinoma transcript 1 (non-protein coding) | other |  |  |  |  | 2,00E-06 | 1,574 |
| 223991_s_at | LOC100132910 | PRO1477 | other |  |  | 4,56E-09 | -1,873 |  |  |
| 223993_s_at | CNIH4 | cornichon homolog 4 (Drosophila) | other |  |  | 5,75E-08 | -1,494 |  |  |
| 223996_s_at | MRPL30 | mitochondrial ribosomal protein L30 | other |  |  | 5,65E-08 | -1,562 |  |  |
| 224068_x_at | RBM22 | RNA binding motif protein 22 | other | 1,83E-14 | -1,322 | 1,83E-14 | -2,011 | 1,83E-14 | -1,499 |
| 224129_s_at | DPY30 | dpy-30 homolog (C. elegans) | other | 2,81E-08 | -1,417 | 2,81E-08 | -1,577 |  |  |
| 224130_s_at | SRA1 | steroid receptor RNA activator 1 | transcription regulator |  |  | 2,47E-07 | -1,688 |  |  |
| 224150_s_at | CEP70 | centrosomal protein 70kDa | other | 9,52E-11 | -1,665 | 9,52E-11 | -1,709 | 9,52E-11 | -1,614 |
| 224173_s_at | MRPL30 | mitochondrial ribosomal protein L30 | other | 1,83E-14 | -1,690 | 1,83E-14 | -2,729 | 1,83E-14 | -2,435 |
| 224206_x_at | MYNN | myoneurin | transcription regulator | 1,44E-12 | -1,548 | 1,44E-12 | -1,923 | 1,44E-12 | -1,390 |
| 224232_s_at | PRELID1 | PRELI domain containing 1 | other |  |  | 4,98E-11 | -1,654 |  |  |
| 224252_s_at | FXYD5 | FXYD domain containing ion transport regulator 5 | ion channel |  |  | 4,07E-12 | -2,333 |  |  |
| 224302_s_at | MRPS36 | mitochondrial ribosomal protein S36 | other |  |  | 2,04E-11 | -1,640 | 2,04E-11 | -1,370 |
| 224312_x_at | CPSF3L | cleavage and polyadenylation specific factor 3-like | other |  |  | 6,73E-08 | -2,092 |  |  |
| 224326_s_at | PCGF6 | polycomb group ring finger 6 | transcription regulator |  |  | 5,40E-09 | -1,757 |  |  |
| 224332_s_at | MRPL43 | mitochondrial ribosomal protein L43 | translation regulator |  |  | 3,09E-09 | -1,703 |  |  |
| 224333_s_at | MRPS5 | mitochondrial ribosomal protein S5 | other |  |  | 3,75E-10 | -1,680 |  |  |
| 224366_s_at | REPS1 | RALBP1 associated Eps domain containing 1 | other |  |  | 3,73E-09 | -1,667 |  |  |
| 224391_s_at | SIAE | sialic acid acetylesterase | enzyme |  |  | 6,34E-08 | -1,652 |  |  |
| 224395_s_at | RNF7 | ring finger protein 7 | other | 1,16E-12 | -1,476 | 1,16E-12 | -1,909 | 1,16E-12 | -1,382 |
| 224413_s_at | TM2D2 | TM2 domain containing 2 | other | 1,83E-14 | -1,657 | 1,83E-14 | -1,839 | 1,83E-14 | -1,345 |
| 224415_s_at | HINT2 | histidine triad nucleotide binding protein 2 | other |  |  | 8,89E-13 | -1,917 | 8,89E-13 | -1,459 |
| 224436_s_at | NIPSNAP3A | nipsnap homolog 3A (C. elegans) | other | 1,83E-14 | -1,589 | 1,83E-14 | -1,591 | 1,83E-14 | -1,736 |
| 224439_x_at | RNF7 | ring finger protein 7 | other |  |  | 1,22E-07 | -1,559 |  |  |
| 224445_s_at | ZFYVE21 | zinc finger, FYVE domain containing 21 | other |  |  | 7,52E-11 | -2,000 |  |  |
| 224447_s_at | C17ORF37 | chromosome 17 open reading frame 37 | other |  |  | 7,32E-10 | -1,916 |  |  |
| 224448_s_at | C6ORF125 | chromosome 6 open reading frame 125 | other |  |  | 8,88E-14 | -2,045 |  |  |
| 224468_s_at | C19ORF48 | chromosome 19 open reading frame 48 | other |  |  | 1,01E-07 | -1,796 |  |  |
| 224472_x_at | SDF4 | stromal cell derived factor 4 | other |  |  | 7,56E-08 | -1,729 |  |  |
| 224474_x_at | SMEK2 | SMEK homolog 2, suppressor of mek1 (Dictyostelium) | other | 1,83E-14 | -1,625 | 1,83E-14 | -2,535 | 1,83E-14 | -1,937 |
| 224478_s_at | C7ORF50 | chromosome 7 open reading frame 50 | other | 1,74E-13 | -1,596 | 1,74E-13 | -2,192 | 1,74E-13 | -1,753 |
| 224480_s_at | AGPAT9 | 1-acylglycerol-3-phosphate O-acyltransferase 9 | enzyme |  |  |  |  | 2,45E-10 | 1,877 |
| 224502_s_at | KIAA1191 | KIAA1191 | other |  |  | 5,95E-12 | -1,641 | 5,95E-12 | -1,257 |
| 224504_s_at | BUD13 | BUD13 homolog (S. cerevisiae) | other | 2,56E-10 | -1,526 | 2,56E-10 | -1,770 |  |  |
| 224512_s_at | LSMD1 | LSM domain containing 1 | other | 6,68E-10 | -1,367 | 6,68E-10 | -1,818 |  |  |
| 224516_s_at | CXXC5 | CXXC finger 5 | other |  |  | 1,11E-08 | -1,764 |  |  |
| 224560_at | TIMP2 | TIMP metallopeptidase inhibitor 2 | other |  |  | 4,40E-09 | -1,800 |  |  |
| 224564_s_at | RTN3 | reticulon 3 | other | 3,82E-13 | -1,626 | 3,82E-13 | -1,806 | 3,82E-13 | -1,476 |
| 224565_at | NCRNA00084 | non-protein coding RNA 84 | other | 8,72E-09 | -1,339 | 8,72E-09 | -1,615 |  |  |
| 224566_at | NCRNA00084 | non-protein coding RNA 84 | other |  |  | 1,23E-10 | -2,068 |  |  |
| 224567_x_at | MALAT1 | metastasis associated lung adenocarcinoma transcript 1 (non-protein coding) | other |  |  |  |  | 8,94E-09 | 1,477 |
| 224570_s_at | IRF2BP2 | interferon regulatory factor 2 binding protein 2 | transcription regulator |  |  | 3,87E-10 | -1,805 |  |  |
| 224573_at | RNASEK | ribonuclease, RNase K | peptidase |  |  | 7,76E-09 | -1,660 |  |  |
| 224575_at | C3ORF10 | chromosome 3 open reading frame 10 | other | 3,03E-11 | -1,616 | 3,03E-11 | -1,809 |  |  |
| 224576_at | ERGIC1 | endoplasmic reticulum-golgi intermediate compartment (ERGIC) 1 | other |  |  | 8,06E-12 | -1,689 |  |  |
| 224577_at | ERGIC1 | endoplasmic reticulum-golgi intermediate compartment (ERGIC) 1 | other |  |  | 4,42E-11 | -1,947 |  |  |
| 224579_at | SLC38A1 | solute carrier family 38, member 1 | transporter |  |  | 2,11E-12 | -1,649 |  |  |
| 224582_s_at | NUCKS1 | nuclear casein kinase and cyclin-dependent kinase substrate 1 | kinase | 1,83E-14 | -1,848 | 1,83E-14 | -2,829 | 1,83E-14 | -2,095 |
| 224583_at | COTL1 | coactosin-like 1 (Dictyostelium) | other | 1,08E-10 | -1,449 | 1,08E-10 | -1,605 |  |  |
| 224587_at | SUB1 | SUB1 homolog (S. cerevisiae) | transcription regulator | 2,86E-09 | -1,409 | 2,86E-09 | -1,501 | 2,86E-09 | -1,303 |
| 224591_at | HP1BP3 | heterochromatin protein 1, binding protein 3 | other | 1,36E-09 | -1,460 | 1,36E-09 | -1,543 | 1,36E-09 | -1,308 |
| 224593_at | ZNF664 | zinc finger protein 664 | other |  |  | 1,50E-13 | -1,856 | 1,50E-13 | -1,441 |
| 224596_at | SLC44A1 | solute carrier family 44, member 1 | transporter |  |  | 8,27E-11 | -1,612 |  |  |
| 224598_at | MGAT4B | mannosyl (alpha-1,3-)-glycoprotein beta-1,4-N-acetylglucosaminyltransferase, isozyme B | enzyme |  |  | 2,68E-09 | -1,529 |  |  |
| 224600_at | CGGBP1 | CGG triplet repeat binding protein 1 | other |  |  | 4,64E-08 | -1,492 |  |  |
| 224602_at | LOC401152 | chromosome 4 open reading frame 3 | other | 3,18E-09 | -1,318 | 3,18E-09 | -1,633 |  |  |
| 224604_at | LOC401152 | chromosome 4 open reading frame 3 | other | 3,76E-09 | -1,374 | 3,76E-09 | -1,590 | 3,76E-09 | -1,425 |
| 224606_at | KLF6 | Kruppel-like factor 6 | transcription regulator |  |  |  |  | 1,83E-14 | 1,653 |
| 224607_s_at | SRP68 | signal recognition particle 68kDa | other |  |  | 9,79E-08 | -1,642 |  |  |
| 224608_s_at | VPS25 | vacuolar protein sorting 25 homolog (S. cerevisiae) | other |  |  | 1,10E-09 | -1,690 |  |  |
| 224609_at | SLC44A2 | solute carrier family 44, member 2 | transporter | 1,83E-14 | -1,573 | 1,83E-14 | -2,218 | 1,83E-14 | -1,796 |
| 224615_x_at | HM13 | histocompatibility (minor) 13 | peptidase |  |  | 1,37E-12 | -2,269 |  |  |
| 224616_at | DYNC1LI2 | dynein, cytoplasmic 1, light intermediate chain 2 | other | 1,06E-10 | -1,571 |  |  |  |  |
| 224619_at | CASC4 | cancer susceptibility candidate 4 | other |  |  | 1,87E-08 | -1,532 |  |  |
| 224626_at | SLC35A4 | solute carrier family 35, member A4 | transporter |  |  | 7,34E-11 | -1,928 |  |  |
| 224628_at | C2ORF30 | chromosome 2 open reading frame 30 | other | 7,40E-12 | -1,426 | 7,40E-12 | -1,609 |  |  |
| 224630_at | C2ORF30 | chromosome 2 open reading frame 30 | other |  |  | 2,73E-09 | -1,594 |  |  |
| 224639_at | UNQ1887 | signal peptide peptidase 3 | peptidase | 4,45E-11 | -1,421 | 4,45E-11 | -1,738 | 4,45E-11 | -1,395 |
| 224643_at | PRRC1 | proline-rich coiled-coil 1 | other |  |  | 2,79E-08 | -1,509 |  |  |
| 224650_at | MAL2 | mal, T-cell differentiation protein 2 | transporter |  |  | 4,47E-12 | -1,703 |  |  |
| 224651_at | CCNY | cyclin Y | other |  |  | 3,47E-10 | -1,738 |  |  |
| 224655_at | AK3 | adenylate kinase 3 | kinase |  |  | 9,81E-11 | -1,652 |  |  |
| 224657_at | ERRFI1 | ERBB receptor feedback inhibitor 1 | other | 4,51E-08 | -1,564 |  |  |  |  |
| 224660_at | PIGY (includes EG:84992) | phosphatidylinositol glycan anchor biosynthesis, class Y | other |  |  | 5,58E-13 | -1,844 |  |  |
| 224662_at | KIF5B | kinesin family member 5B | other | 1,83E-14 | -1,494 | 1,83E-14 | -1,671 |  |  |
| 224663_s_at | CFL2 | cofilin 2 (muscle) | other | 3,44E-14 | -1,487 | 3,44E-14 | -2,034 | 3,44E-14 | -1,370 |
| 224664_at | C10ORF104 | chromosome 10 open reading frame 104 | other |  |  | 1,43E-08 | -1,492 |  |  |
| 224669_at | SYS1 (includes EG:90196) | SYS1 Golgi-localized integral membrane protein homolog (S. cerevisiae) | other | 3,57E-08 | -1,463 | 3,57E-08 | -1,714 |  |  |
| 224671_at | MRPL10 | mitochondrial ribosomal protein L10 | other |  |  | 1,84E-09 | -1,771 |  |  |
| 224675_at | MESDC2 | mesoderm development candidate 2 | other |  |  | 1,44E-08 | -1,561 |  |  |
| 224680_at | TMED4 | transmembrane emp24 protein transport domain containing 4 | transporter |  |  | 1,81E-10 | -1,712 | 1,81E-10 | -1,308 |
| 224684_at | SNX12 | sorting nexin 12 | transporter | 2,84E-12 | -1,530 | 2,84E-12 | -1,849 | 2,84E-12 | -1,487 |
| 224689_at | MANBAL | mannosidase, beta A, lysosomal-like | other |  |  | 8,47E-09 | -1,667 |  |  |
| 224690_at | C20ORF108 | chromosome 20 open reading frame 108 | other |  |  | 6,53E-08 | -1,589 |  |  |
| 224700_at | STT3B | STT3, subunit of the oligosaccharyltransferase complex, homolog B (S. cerevisiae) | enzyme |  |  | 1,59E-11 | -1,666 |  |  |
| 224705_s_at | TNRC6A | trinucleotide repeat containing 6A | other |  |  | 1,54E-08 | -1,634 |  |  |
| 224706_at | KIAA2013 | KIAA2013 | other |  |  | 7,32E-12 | -2,273 |  |  |
| 224715_at | WDR34 | WD repeat domain 34 | other | 1,83E-14 | -1,587 | 1,83E-14 | -2,229 | 1,83E-14 | -1,572 |
| 224717_s_at | C19ORF42 | chromosome 19 open reading frame 42 | other |  |  | 5,01E-09 | -1,664 |  |  |
| 224721_at | WDR75 | WD repeat domain 75 | other |  |  |  |  | 1,41E-10 | 1,476 |
| 224725_at | MIB1 | mindbomb homolog 1 (Drosophila) | other | 4,40E-10 | -1,678 | 4,40E-10 | -1,553 |  |  |
| 224726_at | MIB1 | mindbomb homolog 1 (Drosophila) | other |  |  |  |  | 1,88E-11 | 1,800 |
| 224731_at | HMGB1 (includes EG:3146) | high-mobility group box 1 | other |  |  | 2,47E-08 | -1,464 |  |  |
| 224732_at | CTF8 | chromosome transmission fidelity factor 8 homolog (S. cerevisiae) | other |  |  | 2,82E-08 | -1,855 |  |  |
| 224747_at | UBE2Q2 | ubiquitin-conjugating enzyme E2Q family member 2 | enzyme | 1,83E-14 | -1,512 | 1,83E-14 | -1,835 | 1,83E-14 | -1,492 |
| 224749_at | ITFG3 | integrin alpha FG-GAP repeat containing 3 | other |  |  | 1,73E-11 | -2,253 |  |  |
| 224751_at | SLC13A4 | solute carrier family 13 (sodium/sulfate symporters), member 4 | transporter | 4,02E-13 | -1,463 | 4,02E-13 | -1,783 | 4,02E-13 | -1,424 |
| 224752_at | SLC13A4 | solute carrier family 13 (sodium/sulfate symporters), member 4 | transporter | 1,14E-12 | -1,334 | 1,14E-12 | -1,817 | 1,14E-12 | -1,422 |
| 224759_s_at | C12ORF23 | chromosome 12 open reading frame 23 | other |  |  | 1,51E-08 | -1,570 |  |  |
| 224779_s_at | FAM96A | family with sequence similarity 96, member A | other |  |  | 3,24E-07 | -1,451 |  |  |
| 224780_at | RBM17 | RNA binding motif protein 17 | other |  |  | 2,53E-09 | -2,145 |  |  |
| 224782_at | ZMAT2 | zinc finger, matrin type 2 | other | 2,83E-12 | -1,464 | 2,83E-12 | -1,729 |  |  |
| 224786_at | SCOC | short coiled-coil protein | other | 2,86E-09 | -1,369 | 2,86E-09 | -1,481 | 2,86E-09 | -1,362 |
| 224791_at | ASAP1 | ArfGAP with SH3 domain, ankyrin repeat and PH domain 1 | other |  |  | 1,40E-11 | -1,699 |  |  |
| 224793_s_at | TGFBR1 | transforming growth factor, beta receptor 1 | kinase | 1,94E-10 | -1,442 | 1,94E-10 | -1,674 |  |  |
| 224797_at | ARRDC3 | arrestin domain containing 3 | other | 6,70E-13 | -2,194 |  |  | 6,70E-13 | -1,435 |
| 224806_at | TRIM25 | tripartite motif-containing 25 | transcription regulator |  |  | 1,77E-08 | -1,708 |  |  |
| 224810_s_at | ANKRD13A | ankyrin repeat domain 13A | other |  |  | 1,33E-08 | -1,584 |  |  |
| 224812_at | HIBADH | 3-hydroxyisobutyrate dehydrogenase | enzyme | 6,97E-09 | -1,437 | 6,97E-09 | -1,575 | 6,97E-09 | -1,355 |
| 224814_at | DPP7 | dipeptidyl-peptidase 7 | peptidase |  |  | 1,96E-08 | -2,480 |  |  |
| 224815_at | COMMD7 | COMM domain containing 7 | other |  |  | 9,39E-09 | -1,603 |  |  |
| 224820_at | FAM36A | family with sequence similarity 36, member A | other |  |  | 9,09E-09 | -1,691 |  |  |
| 224821_at | ABHD14B | abhydrolase domain containing 14B | enzyme |  |  | 2,48E-10 | -2,015 |  |  |
| 224823_at | MYLK | myosin light chain kinase | kinase |  |  | 1,28E-08 | -1,590 |  |  |
| 224824_at | FAM36A | family with sequence similarity 36, member A | other |  |  | 3,24E-10 | -1,726 |  |  |
| 224825_at | DNTTIP1 | deoxynucleotidyltransferase, terminal, interacting protein 1 | ligand-dependent nuclear receptor | 1,03E-10 | -1,588 | 1,03E-10 | -1,983 | 1,03E-10 | -1,521 |
| 224832_at | DUSP16 | dual specificity phosphatase 16 | phosphatase |  |  | 2,66E-09 | -1,758 |  |  |
| 224833_at | ETS1 | v-ets erythroblastosis virus E26 oncogene homolog 1 (avian) | transcription regulator |  |  |  |  | 1,83E-14 | 1,804 |
| 224838_at | FOXP1 | forkhead box P1 | transcription regulator |  |  | 1,49E-10 | -1,782 | 1,49E-10 | -1,345 |
| 224839_s_at | GPT2 | glutamic pyruvate transaminase (alanine aminotransferase) 2 | enzyme | 1,19E-08 | -1,510 | 1,19E-08 | -1,846 |  |  |
| 224850_at | ATAD1 | ATPase family, AAA domain containing 1 | other | 2,93E-08 | -1,391 |  |  |  |  |
| 224858_at | ZDHHC5 | zinc finger, DHHC-type containing 5 | other |  |  | 2,89E-10 | -1,846 |  |  |
| 224864_at | SRA1 | steroid receptor RNA activator 1 | transcription regulator |  |  | 2,07E-09 | -1,697 |  |  |
| 224867_at | C1ORF151 | chromosome 1 open reading frame 151 | other |  |  | 5,36E-09 | -1,629 |  |  |
| 224880_at | RALA | v-ral simian leukemia viral oncogene homolog A (ras related) | enzyme | 4,68E-12 | -1,448 | 4,68E-12 | -1,645 |  |  |
| 224885_s_at | KRTCAP2 | keratinocyte associated protein 2 | enzyme |  |  | 3,28E-11 | -1,622 |  |  |
| 224890_s_at | C7ORF59 | chromosome 7 open reading frame 59 | other |  |  | 4,00E-09 | -1,736 |  |  |
| 224892_at | PLDN | pallidin homolog (mouse) | other | 3,27E-10 | -1,482 | 3,27E-10 | -1,555 | 3,27E-10 | -1,288 |
| 224894_at | YAP1 | Yes-associated protein 1, 65kDa | transcription regulator | 1,83E-14 | -1,289 | 1,83E-14 | -1,737 | 1,83E-14 | -1,343 |
| 224896_s_at | TTL | tubulin tyrosine ligase | enzyme |  |  | 2,59E-12 | -1,808 |  |  |
| 224899_s_at | MAGT1 | magnesium transporter 1 | enzyme |  |  | 1,97E-10 | -1,655 |  |  |
| 224903_at | CIRH1A | cirrhosis, autosomal recessive 1A (cirhin) | other | 1,83E-14 | -1,475 | 1,83E-14 | -1,837 |  |  |
| 224904_at | PDPR | pyruvate dehydrogenase phosphatase regulatory subunit | enzyme |  |  | 5,97E-08 | -1,658 |  |  |
| 224910_at | CARHSP1 | calcium regulated heat stable protein 1, 24kDa | other | 5,92E-09 | -1,624 | 5,92E-09 | -1,776 | 5,92E-09 | -1,437 |
| 224917_at | MIRN21 (includes EG:406991) | microRNA 21 | other |  |  |  |  | 1,83E-14 | 1,625 |
| 224926_at | EXOC4 | exocyst complex component 4 | transporter |  |  | 2,39E-11 | -2,030 | 2,39E-11 | -1,409 |
| 224936_at | EIF2S3 | eukaryotic translation initiation factor 2, subunit 3 gamma, 52kDa | translation regulator |  |  | 1,35E-08 | -1,507 |  |  |
| 224944_at | TMPO | thymopoietin | other |  |  | 7,45E-08 | -1,560 |  |  |
| 224946_s_at | CCDC115 | coiled-coil domain containing 115 | other | 1,15E-08 | -1,387 | 1,15E-08 | -1,578 |  |  |
| 224947_at | RNF26 | ring finger protein 26 | other |  |  | 2,72E-08 | -1,757 |  |  |
| 224948_at | MRPS24 | mitochondrial ribosomal protein S24 | other |  |  | 8,69E-10 | -1,595 |  |  |
| 224949_at | YIPF5 | Yip1 domain family, member 5 | other | 1,54E-12 | -1,743 | 1,54E-12 | -1,866 |  |  |
| 224951_at | LASS5 | LAG1 homolog, ceramide synthase 5 | transcription regulator | 8,88E-14 | -1,591 | 8,88E-14 | -2,078 | 8,88E-14 | -1,447 |
| 224960_at | SCYL2 | SCY1-like 2 (S. cerevisiae) | other | 1,08E-10 | -1,302 | 1,08E-10 | -1,588 |  |  |
| 224961_at | SCYL2 | SCY1-like 2 (S. cerevisiae) | other |  |  | 3,70E-12 | -1,821 |  |  |
| 224974_at | SUDS3 | suppressor of defective silencing 3 homolog (S. cerevisiae) | other | 3,27E-12 | -1,641 | 3,27E-12 | -1,630 |  |  |
| 224981_at | TMEM219 | transmembrane protein 219 | other |  |  | 5,89E-08 | -1,826 |  |  |
| 224983_at | SCARB2 | scavenger receptor class B, member 2 | other |  |  | 4,41E-09 | -1,658 |  |  |
| 224984_at | NFAT5 | nuclear factor of activated T-cells 5, tonicity-responsive | transcription regulator |  |  | 3,50E-08 | -1,489 |  |  |
| 224998_at | CMTM4 | CKLF-like MARVEL transmembrane domain containing 4 | cytokine |  |  | 4,21E-11 | -1,742 |  |  |
| 225001_at | RAB3D | RAB3D, member RAS oncogene family | enzyme |  |  | 1,20E-10 | -2,491 | 1,20E-10 | -2,269 |
| 225003_at | TMEM205 | transmembrane protein 205 | other |  |  | 1,18E-08 | -1,848 |  |  |
| 225005_at | PHF13 | PHD finger protein 13 | other |  |  | 8,40E-09 | -1,690 |  |  |
| 225006_x_at | TH1L | TH1-like (Drosophila) | other |  |  | 4,40E-08 | -1,589 |  |  |
| 225011_at | PRKAR2A | protein kinase, cAMP-dependent, regulatory, type II, alpha | kinase |  |  | 4,65E-09 | -1,514 |  |  |
| 225014_at | LOC389203 | hypothetical gene supported by BC032431 | other |  |  | 9,82E-08 | -1,688 |  |  |
| 225022_at | GOPC | golgi associated PDZ and coiled-coil motif containing | transporter | 4,38E-08 | -1,478 |  |  | 4,38E-08 | -1,445 |
| 225029_at | LOC550643 | hypothetical LOC550643 | other |  |  | 2,56E-09 | -1,731 |  |  |
| 225030_at | FAM44B | family with sequence similarity 44, member B | other |  |  | 4,51E-09 | -1,572 |  |  |
| 225035_x_at | WASH1 | WAS protein family homolog 1 | other |  |  | 6,81E-08 | -1,851 |  |  |
| 225038_s_at | SURF6 | surfeit 6 | other |  |  | 6,62E-09 | -2,098 |  |  |
| 225039_at | RPE | ribulose-5-phosphate-3-epimerase | enzyme |  |  | 6,15E-08 | -1,529 |  |  |
| 225046_at | LOC100132181 | hypothetical protein LOC100132181 | other |  |  | 7,23E-09 | -1,525 |  |  |
| 225052_at | TMEM203 | transmembrane protein 203 | other |  |  | 1,47E-08 | -1,678 |  |  |
| 225053_at | CNOT7 | CCR4-NOT transcription complex, subunit 7 | transcription regulator |  |  | 1,36E-09 | -1,538 |  |  |
| 225057_at | SLC15A4 | solute carrier family 15, member 4 | transporter |  |  | 1,83E-09 | -2,036 |  |  |
| 225068_at | KLHL12 | kelch-like 12 (Drosophila) | other | 1,50E-13 | -1,572 | 1,50E-13 | -1,820 |  |  |
| 225078_at | EMP2 | epithelial membrane protein 2 | other | 1,83E-14 | -2,176 | 1,83E-14 | -2,191 | 1,83E-14 | -1,698 |
| 225079_at | EMP2 | epithelial membrane protein 2 | other | 7,75E-11 | -1,657 | 7,75E-11 | -1,962 | 7,75E-11 | -1,707 |
| 225080_at | MYO1C | myosin IC | other |  |  | 1,34E-07 | -1,806 |  |  |
| 225082_at | CPSF3 | cleavage and polyadenylation specific factor 3, 73kDa | other |  |  | 1,49E-10 | -1,606 |  |  |
| 225086_at | FAM98B | family with sequence similarity 98, member B | other |  |  | 7,21E-10 | -1,634 |  |  |
| 225093_at | UTRN | utrophin | transmembrane receptor | 1,09E-09 | -1,613 | 1,09E-09 | -1,367 | 1,09E-09 | -1,410 |
| 225099_at | FBXO45 | F-box protein 45 | other | 2,99E-10 | -1,525 |  |  |  |  |
| 225100_at | FBXO45 | F-box protein 45 | other |  |  |  |  | 1,52E-09 | 1,560 |
| 225103_at | MRPL38 | mitochondrial ribosomal protein L38 | other |  |  | 3,23E-12 | -1,898 |  |  |
| 225105_at | LOC387882 | overexpressed in colon carcinoma-1 | other |  |  | 1,71E-09 | -1,530 | 1,71E-09 | -1,271 |
| 225107_at | HNRNPA2B1 | heterogeneous nuclear ribonucleoprotein A2/B1 | other |  |  | 1,59E-10 | -1,489 |  |  |
| 225114_at | AGPS | alkylglycerone phosphate synthase | enzyme | 1,05E-11 | -1,358 | 1,05E-11 | -1,641 | 1,05E-11 | -1,333 |
| 225131_at | ZRANB1 | zinc finger, RAN-binding domain containing 1 | other |  |  | 7,56E-09 | -1,823 |  |  |
| 225132_at | FBXL3 | F-box and leucine-rich repeat protein 3 | enzyme |  |  | 2,28E-10 | -1,772 |  |  |
| 225143_at | SFXN4 | sideroflexin 4 | transporter |  |  | 1,78E-11 | -1,783 |  |  |
| 225147_at | CYTH3 | cytohesin 3 | other |  |  |  |  | 1,53E-08 | -1,518 |
| 225148_at | RPS19BP1 | ribosomal protein S19 binding protein 1 | other | 1,37E-12 | -1,461 | 1,37E-12 | -1,840 |  |  |
| 225153_at | GFM1 | G elongation factor, mitochondrial 1 | translation regulator |  |  | 3,09E-09 | -1,597 |  |  |
| 225157_at | MLXIP | MLX interacting protein | other |  |  | 9,45E-09 | -1,743 |  |  |
| 225161_at | GFM1 | G elongation factor, mitochondrial 1 | translation regulator |  |  | 1,57E-10 | -1,653 |  |  |
| 225164_s_at | EIF2AK4 | eukaryotic translation initiation factor 2 alpha kinase 4 | kinase | 1,64E-11 | -1,404 | 1,64E-11 | -2,036 |  |  |
| 225170_at | WDR5 | WD repeat domain 5 | other |  |  | 3,67E-10 | -2,052 |  |  |
| 225173_at | ARHGAP18 | Rho GTPase activating protein 18 | other | 1,83E-14 | 1,758 | 1,83E-14 | 1,597 | 1,83E-14 | 2,154 |
| 225177_at | RAB11FIP1 | RAB11 family interacting protein 1 (class I) | other |  |  | 7,69E-12 | -1,953 |  |  |
| 225179_at | UBE2K | ubiquitin-conjugating enzyme E2K (UBC1 homolog, yeast) | transcription regulator | 1,83E-14 | -1,529 | 1,83E-14 | -2,465 | 1,83E-14 | -1,522 |
| 225189_s_at | RAPH1 | Ras association (RalGDS/AF-6) and pleckstrin homology domains 1 | other |  |  | 3,01E-08 | -1,553 |  |  |
| 225195_at | DPH3 | DPH3, KTI11 homolog (S. cerevisiae) | other |  |  | 1,84E-09 | -1,563 |  |  |
| 225201_s_at | MRPL14 | mitochondrial ribosomal protein L14 | other |  |  | 1,50E-08 | -1,657 |  |  |
| 225202_at | RHOBTB3 | Rho-related BTB domain containing 3 | enzyme |  |  | 4,11E-10 | -1,626 |  |  |
| 225205_at | KIF3B | kinesin family member 3B | transporter |  |  | 3,18E-12 | -1,856 |  |  |
| 225206_s_at | MTRF1L | mitochondrial translational release factor 1-like | translation regulator |  |  | 4,45E-11 | -2,329 |  |  |
| 225209_s_at | UBE2J2 | ubiquitin-conjugating enzyme E2, J2 (UBC6 homolog, yeast) | enzyme |  |  | 5,41E-08 | -1,650 |  |  |
| 225210_s_at | FAM103A1 | family with sequence similarity 103, member A1 | other |  |  | 2,12E-08 | -1,511 |  |  |
| 225216_at | CXORF39 | chromosome X open reading frame 39 | other |  |  | 6,12E-09 | -1,690 |  |  |
| 225217_s_at | BRPF3 | bromodomain and PHD finger containing, 3 | other |  |  | 6,39E-08 | -1,502 |  |  |
| 225230_at | TMEM77 | transmembrane protein 77 | other |  |  | 2,29E-09 | -1,740 |  |  |
| 225231_at | CBL | Cas-Br-M (murine) ecotropic retroviral transforming sequence | transcription regulator |  |  | 2,76E-11 | -1,644 |  |  |
| 225232_at | MTMR12 | myotubularin related protein 12 | other |  |  | 1,30E-09 | -1,538 |  |  |
| 225261_x_at | TH1L | TH1-like (Drosophila) | other |  |  | 3,64E-09 | -1,662 |  |  |
| 225262_at | FOSL2 | FOS-like antigen 2 | transcription regulator | 1,24E-10 | -1,610 | 1,24E-10 | -1,779 | 1,24E-10 | -1,445 |
| 225265_at | RBMS1 | RNA binding motif, single stranded interacting protein 1 | other |  |  | 1,53E-09 | -1,679 |  |  |
| 225267_at | KPNA4 | karyopherin alpha 4 (importin alpha 3) | transporter |  |  | 9,15E-09 | -1,602 |  |  |
| 225268_at | KPNA4 | karyopherin alpha 4 (importin alpha 3) | transporter |  |  | 7,37E-08 | -1,469 |  |  |
| 225272_at | SAT2 | spermidine/spermine N1-acetyltransferase family member 2 | enzyme |  |  | 1,58E-10 | -2,096 |  |  |
| 225283_at | ARRDC4 | arrestin domain containing 4 | other | 2,30E-12 | -1,839 |  |  | 2,30E-12 | -2,068 |
| 225285_at | BCAT1 | branched chain aminotransferase 1, cytosolic | enzyme | 1,83E-14 | -1,718 | 1,83E-14 | -1,867 | 1,83E-14 | -1,721 |
| 225287_s_at | TMEM55B | transmembrane protein 55B | other | 1,83E-14 | -1,723 | 1,83E-14 | -2,422 | 1,83E-14 | -1,813 |
| 225295_at | SLC39A10 | solute carrier family 39 (zinc transporter), member 10 | transporter | 1,83E-14 | -1,687 | 1,83E-14 | -1,787 | 1,83E-14 | -1,704 |
| 225297_at | CCDC5 | coiled-coil domain containing 5 (spindle associated) | other |  |  | 1,14E-10 | -1,779 |  |  |
| 225298_at | PNKD (includes EG:25953) | paroxysmal nonkinesigenic dyskinesia | other | 6,76E-13 | -1,421 | 6,76E-13 | -2,025 | 6,76E-13 | -1,567 |
| 225300_at | C15ORF23 | chromosome 15 open reading frame 23 | other |  |  | 2,94E-09 | -1,638 |  |  |
| 225303_at | KIRREL | kin of IRRE like (Drosophila) | other | 2,09E-11 | -1,735 | 2,09E-11 | -1,818 |  |  |
| 225304_s_at | NDUFA11 | NADH dehydrogenase (ubiquinone) 1 alpha subcomplex, 11, 14.7kDa | enzyme |  |  | 7,64E-12 | -1,734 |  |  |
| 225307_at | ZNF511 | zinc finger protein 511 | other | 2,53E-10 | -1,312 | 2,53E-10 | -1,709 |  |  |
| 225309_at | PHF5A | PHD finger protein 5A | transcription regulator |  |  | 9,03E-08 | -1,539 |  |  |
| 225310_at | RBMX | RNA binding motif protein, X-linked | other | 1,01E-13 | -1,557 | 1,01E-13 | -1,788 | 1,01E-13 | -1,339 |
| 225311_at | IVD | isovaleryl Coenzyme A dehydrogenase | enzyme | 2,73E-10 | -1,583 | 2,73E-10 | -1,867 | 2,73E-10 | -1,446 |
| 225313_at | C20ORF177 | chromosome 20 open reading frame 177 | other | 4,87E-14 | -1,400 | 4,87E-14 | -1,708 | 4,87E-14 | -1,409 |
| 225320_at | CCDC109A | coiled-coil domain containing 109A | other |  |  | 2,41E-10 | -1,873 |  |  |
| 225324_at | CRLS1 | cardiolipin synthase 1 | enzyme |  |  | 7,91E-09 | -1,715 |  |  |
| 225326_at | RBM27 | RNA binding motif protein 27 | other |  |  | 7,87E-12 | -1,764 |  |  |
| 225331_at | CCDC50 | coiled-coil domain containing 50 | other |  |  | 4,41E-10 | -1,699 |  |  |
| 225334_at | C10ORF32 | chromosome 10 open reading frame 32 | other | 6,26E-14 | -1,554 | 6,26E-14 | -1,754 | 6,26E-14 | -1,557 |
| 225336_at | SFRS2IP | splicing factor, arginine/serine-rich 2, interacting protein | other | 1,83E-14 | -1,789 | 1,83E-14 | -1,707 | 1,83E-14 | -1,512 |
| 225338_at | ZYG11B | zyg-11 homolog B (C. elegans) | other |  |  | 9,70E-08 | -1,548 |  |  |
| 225340_s_at | CAPRIN1 | cell cycle associated protein 1 | other |  |  | 1,65E-08 | -1,525 |  |  |
| 225344_at | NCOA7 | nuclear receptor coactivator 7 | other |  |  |  |  | 1,83E-14 | 1,813 |
| 225351_at | FAM45A | family with sequence similarity 45, member A | other |  |  | 3,29E-11 | -1,741 |  |  |
| 225355_at | LOC54492 | neuralized-2 | other |  |  | 2,03E-07 | -1,861 |  |  |
| 225357_s_at | INO80 | INO80 homolog (S. cerevisiae) | other |  |  | 6,71E-10 | -2,358 |  |  |
| 225358_at | DNAJC19 | DnaJ (Hsp40) homolog, subfamily C, member 19 | other |  |  | 7,67E-09 | -1,660 |  |  |
| 225359_at | DNAJC19 | DnaJ (Hsp40) homolog, subfamily C, member 19 | other |  |  | 4,37E-12 | -1,961 |  |  |
| 225373_at | C10ORF54 | chromosome 10 open reading frame 54 | other | 7,84E-09 | -1,702 | 7,84E-09 | -1,648 |  |  |
| 225374_at | TMEM199 | transmembrane protein 199 | other |  |  | 6,23E-10 | -1,854 |  |  |
| 225389_at | BTBD6 | BTB (POZ) domain containing 6 | other | 2,35E-09 | -1,671 | 2,35E-09 | -1,506 |  |  |
| 225391_at | LOC93622 | hypothetical LOC93622 | other |  |  | 3,65E-10 | -1,820 |  |  |
| 225392_at | GFM2 | G elongation factor, mitochondrial 2 | translation regulator |  |  | 2,97E-08 | -1,536 |  |  |
| 225394_s_at | ZCRB1 | zinc finger CCHC-type and RNA binding motif 1 | other |  |  | 2,53E-08 | -1,552 |  |  |
| 225395_s_at | FAM120AOS | family with sequence similarity 120A opposite strand | other | 9,94E-11 | -1,451 | 9,94E-11 | -1,864 |  |  |
| 225400_at | TSEN15 | tRNA splicing endonuclease 15 homolog (S. cerevisiae) | other |  |  | 1,70E-07 | -1,927 |  |  |
| 225401_at | C1ORF85 | chromosome 1 open reading frame 85 | other | 1,35E-09 | -1,463 | 1,35E-09 | -1,837 |  |  |
| 225406_at | TWSG1 | twisted gastrulation homolog 1 (Drosophila) | other | 1,83E-14 | -1,624 | 1,83E-14 | -1,778 |  |  |
| 225417_at | EPC1 | enhancer of polycomb homolog 1 (Drosophila) | transcription regulator | 4,24E-10 | -1,746 | 4,24E-10 | -1,629 |  |  |
| 225419_at | C7ORF11 | chromosome 7 open reading frame 11 | other |  |  | 4,24E-08 | -1,608 |  |  |
| 225421_at | PM20D2 | peptidase M20 domain containing 2 | other | 1,83E-14 | -1,631 | 1,83E-14 | -2,047 | 1,83E-14 | -1,620 |
| 225422_at | CDC26 | cell division cycle 26 homolog (S. cerevisiae) | other |  |  | 4,67E-08 | -1,567 |  |  |
| 225424_at | GPAM | glycerol-3-phosphate acyltransferase, mitochondrial | enzyme |  |  | 1,65E-07 | -1,709 |  |  |
| 225427_s_at | APOA1BP | apolipoprotein A-I binding protein | other |  |  | 1,62E-10 | -1,798 |  |  |
| 225432_s_at | CSRP2BP | CSRP2 binding protein | other | 1,89E-10 | -2,018 | 1,89E-10 | -1,769 | 1,89E-10 | -2,078 |
| 225436_at | FAM108C1 | family with sequence similarity 108, member C1 | enzyme |  |  | 1,28E-09 | -1,678 |  |  |
| 225441_x_at | LSMD1 | LSM domain containing 1 | other |  |  | 2,09E-08 | -1,883 |  |  |
| 225452_at | MED1 | mediator complex subunit 1 | transcription regulator |  |  |  |  | 8,62E-11 | 1,663 |
| 225472_at | BAT4 | HLA-B associated transcript 4 | transcription regulator |  |  | 1,58E-10 | -1,756 |  |  |
| 225474_at | MAGI1 | membrane associated guanylate kinase, WW and PDZ domain containing 1 | kinase | 2,20E-10 | -1,671 | 2,20E-10 | -1,772 |  |  |
| 225475_at | MIER1 | mesoderm induction early response 1 homolog (Xenopus laevis) | other | 2,99E-11 | -1,688 | 2,99E-11 | -1,969 | 2,99E-11 | -1,477 |
| 225479_at | LRRC58 | leucine rich repeat containing 58 | other |  |  | 5,94E-10 | -1,696 |  |  |
| 225480_at | C1ORF122 | chromosome 1 open reading frame 122 | other |  |  | 2,04E-11 | -2,142 |  |  |
| 225498_at | CHMP4B | chromatin modifying protein 4B | other |  |  | 1,73E-10 | -1,686 |  |  |
| 225512_at | ZBTB38 | zinc finger and BTB domain containing 38 | transcription regulator | 3,17E-08 | -1,440 |  |  |  |  |
| 225514_at | C14ORF21 | chromosome 14 open reading frame 21 | other |  |  | 1,53E-07 | -2,379 |  |  |
| 225515_s_at | RPL7L1 | ribosomal protein L7-like 1 | transcription regulator |  |  | 3,11E-10 | -1,675 |  |  |
| 225517_at | ZNF770 | zinc finger protein 770 | other |  |  |  |  | 8,82E-13 | 1,349 |
| 225519_at | PPP4R2 | protein phosphatase 4, regulatory subunit 2 | other |  |  | 3,83E-08 | -1,533 |  |  |
| 225528_at | IPO8 | importin 8 | transporter | 7,55E-14 | -1,616 | 7,55E-14 | -1,840 | 7,55E-14 | -1,338 |
| 225535_s_at | TIMM23 | translocase of inner mitochondrial membrane 23 homolog (yeast) | transporter |  |  | 6,40E-09 | -1,523 |  |  |
| 225536_at | TMEM54 | transmembrane protein 54 | other |  |  | 2,04E-09 | -1,910 |  |  |
| 225538_at | ZCCHC9 | zinc finger, CCHC domain containing 9 | other |  |  | 1,95E-10 | -1,710 | 1,95E-10 | -1,331 |
| 225548_at | SHROOM3 | shroom family member 3 | other | 1,00E-08 | -1,640 |  |  | 1,00E-08 | -1,741 |
| 225552_x_at | AURKAIP1 | aurora kinase A interacting protein 1 | enzyme |  |  | 6,90E-09 | -1,600 |  |  |
| 225556_at | LOC203547 | hypothetical protein LOC203547 | other |  |  | 5,98E-09 | -1,690 |  |  |
| 225569_at | EIF2C2 | eukaryotic translation initiation factor 2C, 2 | translation regulator |  |  | 2,10E-12 | -2,045 |  |  |
| 225580_at | MRPL50 | mitochondrial ribosomal protein L50 | other |  |  | 6,80E-10 | -1,638 |  |  |
| 225590_at | SH3RF1 | SH3 domain containing ring finger 1 | other |  |  |  |  | 1,19E-08 | 2,510 |
| 225592_at | NRM | nurim (nuclear envelope membrane protein) | other |  |  | 2,56E-08 | -1,900 |  |  |
| 225618_at | ARHGAP27 | Rho GTPase activating protein 27 | other |  |  | 3,97E-12 | -2,425 |  |  |
| 225623_at | KIAA1737 | KIAA1737 | other |  |  | 2,14E-09 | -1,910 |  |  |
| 225627_s_at | CACHD1 | cache domain containing 1 | other | 5,25E-10 | -1,913 | 5,25E-10 | -1,603 | 5,25E-10 | -1,528 |
| 225639_at | SKAP2 | src kinase associated phosphoprotein 2 | other | 3,85E-12 | -1,738 | 3,85E-12 | -1,658 | 3,85E-12 | -1,472 |
| 225647_s_at | CTSC | cathepsin C | peptidase |  |  | 5,44E-12 | -1,692 | 5,44E-12 | -1,454 |
| 225648_at | STK35 | serine/threonine kinase 35 | kinase |  |  | 7,74E-10 | -1,835 |  |  |
| 225651_at | UBE2E2 | ubiquitin-conjugating enzyme E2E 2 (UBC4/5 homolog, yeast) | enzyme |  |  | 1,69E-08 | -1,963 |  |  |
| 225664_at | COL12A1 | collagen, type XII, alpha 1 | other |  |  | 2,92E-09 | -1,618 |  |  |
| 225665_at | ZAK | sterile alpha motif and leucine zipper containing kinase AZK | kinase |  |  | 6,01E-09 | -1,592 |  |  |
| 225673_at | MYADM | myeloid-associated differentiation marker | other | 1,83E-14 | -1,684 | 1,83E-14 | -1,845 |  |  |
| 225684_at | FAM33A | family with sequence similarity 33, member A | other | 1,83E-14 | -1,365 | 1,83E-14 | -1,809 | 1,83E-14 | -1,329 |
| 225687_at | FAM83D | family with sequence similarity 83, member D | other |  |  | 4,47E-08 | -1,468 |  |  |
| 225688_s_at | PHLDB2 | pleckstrin homology-like domain, family B, member 2 | other | 2,92E-09 | -1,436 | 2,92E-09 | -1,428 |  |  |
| 225692_at | CAMTA1 (includes EG:23261) | calmodulin binding transcription activator 1 | other |  |  | 8,76E-08 | -1,625 |  |  |
| 225693_s_at | CAMTA1 (includes EG:23261) | calmodulin binding transcription activator 1 | other |  |  | 3,95E-09 | -1,753 |  |  |
| 225698_at | C5ORF26 | chromosome 5 open reading frame 26 | other |  |  | 1,18E-08 | -1,539 | 1,18E-08 | -1,426 |
| 225717_at | KIAA1715 | KIAA1715 | other |  |  |  |  | 5,90E-08 | -1,658 |
| 225724_at | FLJ31306 | hypothetical protein FLJ31306 | other | 9,94E-11 | -1,727 | 9,94E-11 | -1,821 | 9,94E-11 | -1,583 |
| 225728_at | SORBS2 | sorbin and SH3 domain containing 2 | other | 7,34E-10 | -2,572 | 7,34E-10 | -2,117 | 7,34E-10 | -2,779 |
| 225737_s_at | FBXO22 | F-box protein 22 | enzyme | 1,10E-11 | -1,647 | 1,10E-11 | -1,837 | 1,10E-11 | -1,506 |
| 225761_at | PAPD4 | PAP associated domain containing 4 | other |  |  | 2,22E-09 | -1,675 | 2,22E-09 | -1,361 |
| 225766_s_at | TNPO1 | transportin 1 | transporter | 3,68E-09 | -1,417 | 3,68E-09 | -1,557 |  |  |
| 225772_s_at | C12ORF62 | chromosome 12 open reading frame 62 | other |  |  | 2,90E-08 | -1,704 |  |  |
| 225773_at | RSPRY1 | ring finger and SPRY domain containing 1 | other |  |  | 5,37E-10 | -1,717 |  |  |
| 225777_at | C9ORF140 | chromosome 9 open reading frame 140 | other |  |  | 2,35E-08 | -1,721 |  |  |
| 225787_at | UBE2F | ubiquitin-conjugating enzyme E2F (putative) | enzyme |  |  | 2,94E-10 | -1,933 |  |  |
| 225788_at | C6ORF153 | chromosome 6 open reading frame 153 | other |  |  | 1,45E-07 | -1,612 |  |  |
| 225792_at | HOOK1 | hook homolog 1 (Drosophila) | other | 4,46E-10 | -1,427 | 4,46E-10 | -1,613 |  |  |
| 225794_s_at | C22ORF32 | chromosome 22 open reading frame 32 | other | 2,35E-10 | -1,579 | 2,35E-10 | -1,814 | 2,35E-10 | -1,524 |
| 225795_at | C22ORF32 | chromosome 22 open reading frame 32 | other | 3,44E-14 | -1,459 | 3,44E-14 | -2,314 | 3,44E-14 | -1,726 |
| 225799_at | NCRNA00152 | non-protein coding RNA 152 | other |  |  | 7,73E-10 | -1,710 |  |  |
| 225807_at | JUB | jub, ajuba homolog (Xenopus laevis) | other | 8,78E-08 | -1,470 |  |  |  |  |
| 225808_at | C17ORF95 | chromosome 17 open reading frame 95 | other |  |  | 3,29E-13 | -1,797 | 3,29E-13 | -1,413 |
| 225813_at | RC3H2 | ring finger and CCCH-type zinc finger domains 2 | other | 2,36E-12 | -1,559 | 2,36E-12 | -1,934 |  |  |
| 225819_at | TBRG1 (includes EG:84897) | transforming growth factor beta regulator 1 | other |  |  | 6,17E-09 | -1,887 |  |  |
| 225823_at | C19ORF70 | chromosome 19 open reading frame 70 | other |  |  | 8,39E-09 | -1,720 |  |  |
| 225824_at | CCNK | cyclin K | other |  |  | 4,41E-11 | -1,777 |  |  |
| 225830_at | PDZD8 | PDZ domain containing 8 | other |  |  | 1,99E-10 | -2,033 |  |  |
| 225836_s_at | C12ORF32 | chromosome 12 open reading frame 32 | other |  |  | 3,10E-11 | -1,513 | 3,10E-11 | -1,878 |
| 225837_at | C12ORF32 | chromosome 12 open reading frame 32 | other | 2,49E-12 | -1,481 | 2,49E-12 | -1,999 | 2,49E-12 | -1,558 |
| 225841_at | C1ORF59 | chromosome 1 open reading frame 59 | other |  |  | 9,26E-09 | -1,614 |  |  |
| 225844_at | POLE4 | polymerase (DNA-directed), epsilon 4 (p12 subunit) | enzyme |  |  | 8,80E-11 | -1,759 |  |  |
| 225845_at | ZBTB44 | zinc finger and BTB domain containing 44 | other | 3,18E-09 | -1,421 | 3,18E-09 | -1,573 | 3,18E-09 | -1,311 |
| 225849_s_at | SFT2D1 | SFT2 domain containing 1 | other |  |  | 1,54E-10 | -1,555 |  |  |
| 225855_at | EPB41L5 | erythrocyte membrane protein band 4.1 like 5 | other | 3,38E-13 | -1,776 | 3,38E-13 | -1,896 | 3,38E-13 | -1,700 |
| 225860_at | LOC729580 | hypothetical LOC729580 | other |  |  | 4,30E-09 | -2,324 |  |  |
| 225865_x_at | TH1L | TH1-like (Drosophila) | other |  |  | 1,35E-08 | -1,608 |  |  |
| 225872_at | SLC35F5 | solute carrier family 35, member F5 | other |  |  | 5,07E-08 | -1,457 |  |  |
| 225885_at | EEA1 | early endosome antigen 1 | other | 4,06E-09 | -1,468 | 4,06E-09 | -1,510 |  |  |
| 225887_at | C13ORF23 | chromosome 13 open reading frame 23 | other |  |  | 1,55E-08 | -1,559 |  |  |
| 225890_at | C20ORF72 | chromosome 20 open reading frame 72 | other |  |  | 2,35E-08 | -1,489 |  |  |
| 225892_at | IREB2 | iron-responsive element binding protein 2 | translation regulator |  |  | 1,36E-09 | -1,625 |  |  |
| 225893_at | RC3H1 | ring finger and CCCH-type zinc finger domains 1 | other | 4,62E-09 | -1,382 |  |  |  |  |
| 225899_x_at | TCAG7.907 | hypothetical LOC402483 | other |  |  |  |  | 1,21E-07 | -1,532 |
| 225901_at | PTPMT1 | protein tyrosine phosphatase, mitochondrial 1 | phosphatase |  |  | 3,05E-08 | -1,687 |  |  |
| 225921_at | NIN | ninein (GSK3B interacting protein) | other |  |  | 9,30E-09 | -1,833 |  |  |
| 225927_at | MAP3K1 | mitogen-activated protein kinase kinase kinase 1 | kinase | 7,51E-08 | -1,366 |  |  |  |  |
| 225951_s_at | CHD2 | chromodomain helicase DNA binding protein 2 | enzyme | 1,89E-12 | -1,399 | 1,89E-12 | -1,725 |  |  |
| 225954_s_at | MIDN | midnolin | other |  |  | 7,92E-12 | -2,211 |  |  |
| 225956_at | C5ORF41 | chromosome 5 open reading frame 41 | other | 5,04E-08 | -1,506 | 5,04E-08 | -1,748 |  |  |
| 225959_s_at | ZNRF1 | zinc and ring finger 1 | other |  |  | 2,71E-09 | -2,025 |  |  |
| 225974_at | TMEM64 | transmembrane protein 64 | other | 1,52E-12 | -1,561 | 1,52E-12 | -1,843 | 1,52E-12 | -1,405 |
| 225986_x_at | CPSF2 | cleavage and polyadenylation specific factor 2, 100kDa | other |  |  | 9,88E-09 | -2,006 |  |  |
| 226001_at | KLHL5 | kelch-like 5 (Drosophila) | other | 1,32E-09 | -1,449 | 1,32E-09 | -1,569 |  |  |
| 226004_at | CABLES2 | Cdk5 and Abl enzyme substrate 2 | other | 4,30E-10 | -1,528 | 4,30E-10 | -1,600 | 4,30E-10 | -1,636 |
| 226006_at | LOC100131801 | similar to hCG2036585 | other |  |  | 5,13E-08 | -1,729 |  |  |
| 226007_at | ISCA2 | iron-sulfur cluster assembly 2 homolog (S. cerevisiae) | other |  |  | 1,28E-11 | -1,775 |  |  |
| 226015_at | ZNF12 | zinc finger protein 12 | other |  |  | 7,33E-09 | -1,695 |  |  |
| 226021_at | RDH10 | retinol dehydrogenase 10 (all-trans) | enzyme |  |  | 1,17E-11 | -2,010 |  |  |
| 226024_at | COMMD1 (includes EG:150684) | copper metabolism (Murr1) domain containing 1 | transporter |  |  | 2,05E-07 | -1,606 |  |  |
| 226026_at | DIRC2 | disrupted in renal carcinoma 2 | other |  |  | 3,73E-11 | -1,750 | 3,73E-11 | -1,350 |
| 226027_at | C9ORF119 | chromosome 9 open reading frame 119 | other | 7,18E-08 | -1,387 |  |  |  |  |
| 226035_at | USP31 | ubiquitin specific peptidase 31 | peptidase |  |  |  |  | 6,80E-10 | 1,694 |
| 226037_s_at | TAF9B | TAF9B RNA polymerase II, TATA box binding protein (TBP)-associated factor, 31kDa | transcription regulator |  |  | 1,32E-07 | -1,521 |  |  |
| 226052_at | BRD4 | bromodomain containing 4 | kinase |  |  | 1,83E-14 | -2,235 | 1,83E-14 | -1,513 |
| 226060_at | RFT1 | RFT1 homolog (S. cerevisiae) | other | 3,40E-11 | -1,619 | 3,40E-11 | -1,848 |  |  |
| 226076_s_at | MBD6 | methyl-CpG binding domain protein 6 | other |  |  | 3,95E-09 | -2,800 |  |  |
| 226085_at | CBX5 | chromobox homolog 5 (HP1 alpha homolog, Drosophila) | other |  |  |  |  | 1,15E-07 | -1,533 |
| 226088_at | ZDHHC12 | zinc finger, DHHC-type containing 12 | other |  |  | 8,77E-09 | -2,816 |  |  |
| 226091_s_at | MRFAP1 | Mof4 family associated protein 1 | other |  |  | 8,63E-08 | -1,470 |  |  |
| 226092_at | MPP5 | membrane protein, palmitoylated 5 (MAGUK p55 subfamily member 5) | kinase |  |  | 5,16E-08 | -1,448 |  |  |
| 226099_at | ELL2 | elongation factor, RNA polymerase II, 2 | transcription regulator | 1,79E-08 | -1,408 | 1,79E-08 | -1,484 |  |  |
| 226104_at | RNF170 (includes EG:81790) | ring finger protein 170 | other | 1,62E-12 | -1,692 | 1,62E-12 | -1,997 | 1,62E-12 | -1,575 |
| 226106_at | RNF141 | ring finger protein 141 | other | 3,24E-08 | -1,402 | 3,24E-08 | -1,471 |  |  |
| 226118_at | CENPO | centromere protein O | other |  |  | 7,92E-10 | -1,742 | 7,92E-10 | -1,351 |
| 226120_at | TTC8 | tetratricopeptide repeat domain 8 | other |  |  | 2,45E-08 | -1,823 |  |  |
| 226121_at | DHRS13 | dehydrogenase/reductase (SDR family) member 13 | enzyme |  |  | 1,98E-07 | -1,717 |  |  |
| 226125_at | SLC9A3 | solute carrier family 9 (sodium/hydrogen exchanger), member 3 | ion channel | 3,82E-13 | -1,533 | 3,82E-13 | -2,517 | 3,82E-13 | -1,837 |
| 226132_s_at | MANEAL | mannosidase, endo-alpha-like | other |  |  | 2,53E-10 | -1,814 |  |  |
| 226145_s_at | FRAS1 | Fraser syndrome 1 | other | 8,71E-09 | -1,484 |  |  | 8,71E-09 | -1,439 |
| 226150_at | PPAPDC1B | phosphatidic acid phosphatase type 2 domain containing 1B | other |  |  | 1,61E-07 | -1,575 |  |  |
| 226165_at | C8ORF59 | chromosome 8 open reading frame 59 | other |  |  | 1,15E-11 | -1,653 |  |  |
| 226184_at | FMNL2 (includes EG:114793) | formin-like 2 | other |  |  |  |  | 1,83E-14 | 1,751 |
| 226190_at | MAP3K13 | mitogen-activated protein kinase kinase kinase 13 | kinase |  |  | 1,61E-09 | -1,579 |  |  |
| 226204_at | C22ORF29 | chromosome 22 open reading frame 29 | other |  |  | 1,03E-08 | -2,091 |  |  |
| 226213_at | ERBB3 | v-erb-b2 erythroblastic leukemia viral oncogene homolog 3 (avian) | kinase | 1,83E-14 | -1,570 | 1,83E-14 | -2,219 | 1,83E-14 | -1,993 |
| 226214_at | GDE1 | glycerophosphodiester phosphodiesterase 1 | enzyme | 2,11E-12 | -1,355 | 2,11E-12 | -2,002 |  |  |
| 226226_at | TMEM45B | transmembrane protein 45B | other |  |  | 5,30E-11 | -1,801 |  |  |
| 226236_at | LOC388789 | hypothetical LOC388789 | other |  |  | 1,51E-10 | -1,617 |  |  |
| 226242_at | C1ORF131 | chromosome 1 open reading frame 131 | other |  |  | 1,98E-10 | -1,708 |  |  |
| 226243_at | C2ORF79 | chromosome 2 open reading frame 79 | other | 3,52E-10 | -1,354 | 3,52E-10 | -1,695 | 3,52E-10 | -1,472 |
| 226259_at | EXOC6 | exocyst complex component 6 | transporter | 9,04E-10 | -1,688 | 9,04E-10 | -1,807 |  |  |
| 226264_at | SUSD1 | sushi domain containing 1 | other |  |  | 2,52E-08 | -1,684 |  |  |
| 226267_at | JDP2 | Jun dimerization protein 2 | transcription regulator | 6,01E-11 | -2,052 | 6,01E-11 | -2,448 | 6,01E-11 | -1,920 |
| 226274_at | CLCN5 | chloride channel 5 | ion channel |  |  | 1,79E-09 | -1,660 |  |  |
| 226276_at | TMEM167A | transmembrane protein 167A | other |  |  | 7,21E-10 | -1,580 |  |  |
| 226287_at | CCDC34 | coiled-coil domain containing 34 | other | 1,01E-13 | -1,391 | 1,01E-13 | -1,912 | 1,01E-13 | -1,468 |
| 226294_x_at | FAM91A1 | family with sequence similarity 91, member A1 | other | 6,26E-14 | -1,748 | 6,26E-14 | -1,945 | 6,26E-14 | -1,480 |
| 226296_s_at | MRPS15 | mitochondrial ribosomal protein S15 | other |  |  | 3,95E-10 | -1,647 |  |  |
| 226300_at | MED19 | mediator complex subunit 19 | other |  |  | 3,03E-10 | -1,756 |  |  |
| 226302_at | ATP8B1 | ATPase, class I, type 8B, member 1 | transporter | 5,25E-12 | -1,436 | 5,25E-12 | -1,670 | 5,25E-12 | -1,500 |
| 226329_s_at | MITD1 | MIT, microtubule interacting and transport, domain containing 1 | other | 1,83E-14 | -1,372 | 1,83E-14 | -1,924 | 1,83E-14 | -1,585 |
| 226336_at | PPIA (includes EG:5478) | peptidylprolyl isomerase A (cyclophilin A) | enzyme |  |  | 1,79E-09 | -1,673 |  |  |
| 226350_at | CHML | choroideremia-like (Rab escort protein 2) | enzyme | 1,14E-10 | -1,438 | 1,14E-10 | -1,648 | 1,14E-10 | -1,556 |
| 226353_at | SPPL2A | signal peptide peptidase-like 2A | peptidase | 7,34E-11 | -1,406 | 7,34E-11 | -1,619 | 7,34E-11 | -1,295 |
| 226360_at | ZNRF3 | zinc and ring finger 3 | other |  |  | 2,88E-09 | -1,809 | 2,88E-09 | -1,393 |
| 226366_at | SHPRH | SNF2 histone linker PHD RING helicase | transcription regulator | 1,17E-10 | -1,491 | 1,17E-10 | -1,533 | 1,17E-10 | -1,493 |
| 226370_at | KLHL15 | kelch-like 15 (Drosophila) | other | 3,25E-10 | -1,674 | 3,25E-10 | -1,809 |  |  |
| 226386_at | C7ORF30 | chromosome 7 open reading frame 30 | other |  |  | 1,32E-07 | -1,519 |  |  |
| 226395_at | HOOK3 | hook homolog 3 (Drosophila) | other |  |  | 9,50E-08 | -1,508 |  |  |
| 226400_at | CDC42 | cell division cycle 42 (GTP binding protein, 25kDa) | enzyme |  |  | 2,99E-13 | -2,007 |  |  |
| 226413_at | LOC400027 | hypothetical gene supported by BC047417 | other | 4,12E-13 | -1,797 | 4,12E-13 | -1,535 | 4,12E-13 | -1,742 |
| 226420_at | EVI1 | ecotropic viral integration site 1 | other | 1,83E-14 | -1,838 | 1,83E-14 | -2,164 | 1,83E-14 | -1,620 |
| 226421_at | AMMECR1 | Alport syndrome, mental retardation, midface hypoplasia and elliptocytosis chromosomal region gene 1 | other | 6,96E-11 | -1,505 | 6,96E-11 | -1,781 | 6,96E-11 | -1,507 |
| 226426_at | ADNP | activity-dependent neuroprotector homeobox | transcription regulator |  |  | 8,88E-14 | -2,017 | 8,88E-14 | -1,534 |
| 226440_at | DUSP22 | dual specificity phosphatase 22 | phosphatase |  |  | 1,40E-08 | -1,988 | 1,40E-08 | -1,513 |
| 226445_s_at | TRIM41 | tripartite motif-containing 41 | other |  |  | 1,93E-10 | -2,251 |  |  |
| 226447_at | ASH1L | ash1 (absent, small, or homeotic)-like (Drosophila) | transcription regulator |  |  | 2,64E-08 | -1,654 | 2,64E-08 | -1,361 |
| 226448_at | FAM89A | family with sequence similarity 89, member A | other |  |  | 1,20E-09 | -2,122 | 1,20E-09 | -1,573 |
| 226452_at | PDK1 | pyruvate dehydrogenase kinase, isozyme 1 | kinase |  |  | 3,26E-08 | -1,641 |  |  |
| 226453_at | RNASEH2C | ribonuclease H2, subunit C | other |  |  | 5,62E-08 | -1,576 |  |  |
| 226456_at | C16ORF75 | chromosome 16 open reading frame 75 | other | 2,60E-11 | -1,610 | 2,60E-11 | -1,796 | 2,60E-11 | -1,649 |
| 226472_at | PPIL4 | peptidylprolyl isomerase (cyclophilin)-like 4 | enzyme |  |  | 5,17E-09 | -1,667 |  |  |
| 226475_at | FAM118A | family with sequence similarity 118, member A | other |  |  | 1,35E-08 | -2,184 |  |  |
| 226481_at | VPRBP | Vpr (HIV-1) binding protein | other | 4,59E-10 | -1,562 | 4,59E-10 | -1,688 |  |  |
| 226482_s_at | HCG 20857 | thiosulfate sulfurtransferase KAT, putative | other |  |  | 2,18E-08 | -1,819 |  |  |
| 226487_at | C12ORF34 | chromosome 12 open reading frame 34 | other |  |  | 8,84E-09 | -2,079 |  |  |
| 226496_at | ZCCHC7 | zinc finger, CCHC domain containing 7 | other |  |  | 4,09E-11 | -1,724 |  |  |
| 226502_at | ELMOD2 | ELMO/CED-12 domain containing 2 | other | 3,20E-11 | -1,620 | 3,20E-11 | -1,761 |  |  |
| 226536_at | NSMCE2 | non-SMC element 2, MMS21 homolog (S. cerevisiae) | other | 5,10E-11 | -1,451 | 5,10E-11 | -1,805 | 5,10E-11 | -1,641 |
| 226547_at | MYST3 | MYST histone acetyltransferase (monocytic leukemia) 3 | enzyme | 2,29E-11 | -1,546 | 2,29E-11 | -1,734 | 2,29E-11 | -1,471 |
| 226553_at | TMPRSS2 | transmembrane protease, serine 2 | peptidase | 1,87E-10 | -2,098 | 1,87E-10 | -2,386 | 1,87E-10 | -2,477 |
| 226556_at | MAP3K13 | mitogen-activated protein kinase kinase kinase 13 | kinase | 4,83E-10 | -1,380 | 4,83E-10 | -1,763 |  |  |
| 226567_at | USP14 | ubiquitin specific peptidase 14 (tRNA-guanine transglycosylase) | peptidase |  |  | 1,52E-12 | -1,860 |  |  |
| 226569_s_at | CHTF18 | CTF18, chromosome transmission fidelity factor 18 homolog (S. cerevisiae) | other |  |  | 2,64E-09 | -1,999 |  |  |
| 226597_at | REEP6 | receptor accessory protein 6 | other |  |  | 1,14E-13 | -1,831 | 1,14E-13 | -1,622 |
| 226609_at | DCBLD1 | discoidin, CUB and LCCL domain containing 1 | other | 6,94E-12 | -1,675 | 6,94E-12 | -1,526 | 6,94E-12 | -1,420 |
| 226611_s_at | CENPV | centromere protein V | other |  |  | 4,10E-10 | -1,493 |  |  |
| 226616_s_at | NDUFV3 | NADH dehydrogenase (ubiquinone) flavoprotein 3, 10kDa | enzyme |  |  | 1,78E-12 | -1,818 |  |  |
| 226617_at | ARL5A | ADP-ribosylation factor-like 5A | enzyme | 1,83E-14 | -1,478 | 1,83E-14 | -1,964 |  |  |
| 226629_at | SLC43A2 | solute carrier family 43, member 2 | transporter | 7,33E-10 | -2,040 | 7,33E-10 | -1,668 | 7,33E-10 | -1,487 |
| 226631_at | METTL10 | methyltransferase like 10 | other |  |  | 4,21E-09 | -1,752 |  |  |
| 226635_at | LOC401504 | hypothetical gene supported by AK091718 | other |  |  | 1,65E-08 | -1,595 |  |  |
| 226638_at | ARHGAP23 | Rho GTPase activating protein 23 | other |  |  | 5,83E-09 | -1,830 |  |  |
| 226642_s_at | NUDCD2 | NudC domain containing 2 | other | 3,45E-11 | -1,393 | 3,45E-11 | -1,642 |  |  |
| 226648_at | HIF1AN | hypoxia inducible factor 1, alpha subunit inhibitor | enzyme | 2,05E-11 | -1,527 | 2,05E-11 | -1,875 |  |  |
| 226650_at | ZFAND2A | zinc finger, AN1-type domain 2A | other |  |  | 5,30E-09 | -1,819 |  |  |
| 226664_at | TBC1D20 | TBC1 domain family, member 20 | other |  |  | 2,44E-08 | -1,995 |  |  |
| 226665_at | AHSA2 (includes EG:130872) | AHA1, activator of heat shock 90kDa protein ATPase homolog 2 (yeast) | other |  |  | 1,15E-09 | -1,643 |  |  |
| 226667_x_at | EPN1 | epsin 1 | other | 9,54E-11 | -2,562 | 9,54E-11 | -3,662 | 9,54E-11 | -2,349 |
| 226675_s_at | MALAT1 | metastasis associated lung adenocarcinoma transcript 1 (non-protein coding) | other |  |  | 9,60E-13 | 1,766 | 9,60E-13 | 2,074 |
| 226685_at | SNTB2 | syntrophin, beta 2 (dystrophin-associated protein A1, 59kDa, basic component 2) | other |  |  | 3,95E-08 | -1,618 |  |  |
| 226686_at | CISD2 | CDGSH iron sulfur domain 2 | other |  |  |  |  | 5,31E-09 | -1,817 |
| 226715_at | FOXK1 | forkhead box K1 | transcription regulator |  |  | 1,16E-12 | -1,998 |  |  |
| 226720_at | PWWP2A | PWWP domain containing 2A | other |  |  | 1,36E-11 | -1,768 |  |  |
| 226732_at | RBM33 | RNA binding motif protein 33 | other | 1,85E-13 | -1,606 | 1,85E-13 | -1,895 |  |  |
| 226744_at | METT10D | methyltransferase 10 domain containing | other |  |  | 3,80E-08 | -1,521 |  |  |
| 226747_at | TXNDC16 | thioredoxin domain containing 16 | enzyme | 1,15E-08 | -1,602 | 1,15E-08 | -1,510 | 1,15E-08 | -1,455 |
| 226781_at | FMC1 | formation of mitochondrial complexes 1 homolog (S. cerevisiae) | other |  |  | 5,90E-09 | -2,249 | 5,90E-09 | -1,858 |
| 226784_at | TWISTNB | TWIST neighbor | other |  |  | 2,21E-09 | -1,581 |  |  |
| 226797_at | MBTD1 | mbt domain containing 1 | other |  |  | 6,39E-08 | -1,634 |  |  |
| 226800_at | EFCAB7 | EF-hand calcium binding domain 7 | other |  |  | 1,86E-07 | -2,257 |  |  |
| 226825_s_at | TMEM165 | transmembrane protein 165 | other |  |  | 1,70E-07 | -1,647 |  |  |
| 226835_s_at | C20ORF199 | chromosome 20 open reading frame 199 | other |  |  | 1,09E-12 | -1,634 | 1,09E-12 | -1,375 |
| 226845_s_at | MYEOV2 | myeloma overexpressed 2 | other |  |  | 1,14E-11 | -1,931 |  |  |
| 226860_at | TMEM19 | transmembrane protein 19 | other |  |  | 3,54E-09 | -1,706 | 3,54E-09 | -1,377 |
| 226867_at | DENND4C | DENN/MADD domain containing 4C | other |  |  | 3,52E-10 | -1,723 |  |  |
| 226868_at | GLT8D3 | glycosyltransferase 8 domain containing 3 | other | 1,59E-11 | -1,622 | 1,59E-11 | -1,738 |  |  |
| 226880_at | NUCKS1 | nuclear casein kinase and cyclin-dependent kinase substrate 1 | kinase |  |  | 7,27E-08 | -1,554 |  |  |
| 226896_at | CHCHD1 | coiled-coil-helix-coiled-coil-helix domain containing 1 | other | 1,65E-12 | -1,336 | 1,65E-12 | -1,730 |  |  |
| 226907_at | PPP1R14C | protein phosphatase 1, regulatory (inhibitor) subunit 14C | other | 3,59E-12 | -1,898 | 3,59E-12 | -1,858 |  |  |
| 226915_s_at | ARPC5L (includes EG:81873) | actin related protein 2/3 complex, subunit 5-like | other |  |  | 5,83E-09 | -1,606 |  |  |
| 226925_at | ACPL2 | acid phosphatase-like 2 | phosphatase |  |  | 2,44E-10 | -1,557 |  |  |
| 226926_at | DMKN | dermokine | other | 6,70E-10 | -1,359 | 6,70E-10 | -1,627 |  |  |
| 226936_at | C6ORF173 | chromosome 6 open reading frame 173 | other |  |  | 1,28E-08 | -1,548 |  |  |
| 226942_at | PHF20L1 | PHD finger protein 20-like 1 | other | 1,41E-11 | -2,094 | 1,41E-11 | -1,808 | 1,41E-11 | -1,416 |
| 226952_at | EAF1 | ELL associated factor 1 | transcription regulator |  |  | 6,35E-09 | -1,688 |  |  |
| 226965_at | FAM116A | family with sequence similarity 116, member A | other |  |  | 2,15E-08 | -1,618 |  |  |
| 226970_at | FBXO33 | F-box protein 33 | other |  |  | 2,95E-09 | -2,507 | 2,95E-09 | -1,654 |
| 226976_at | KPNA6 | karyopherin alpha 6 (importin alpha 7) | transporter |  |  | 2,48E-09 | -1,909 | 2,48E-09 | -1,434 |
| 226980_at | DEPDC1B | DEP domain containing 1B | other | 7,54E-10 | -1,481 | 7,54E-10 | -1,564 | 7,54E-10 | -1,381 |
| 226996_at | LYCAT | lysocardiolipin acyltransferase 1 | enzyme |  |  | 6,46E-10 | -1,583 |  |  |
| 227012_at | SLC25A40 | solute carrier family 25, member 40 | other |  |  | 1,84E-07 | -1,574 |  |  |
| 227056_at | KIAA0141 | KIAA0141 | other | 1,97E-09 | -1,629 | 1,97E-09 | -1,921 | 1,97E-09 | -1,715 |
| 227068_at | PGK1 | phosphoglycerate kinase 1 | kinase | 3,20E-13 | -1,411 | 3,20E-13 | -1,890 | 3,20E-13 | -1,416 |
| 227094_at | DHTKD1 | dehydrogenase E1 and transketolase domain containing 1 | enzyme |  |  | 8,67E-10 | -1,879 |  |  |
| 227105_at | CSPP1 | centrosome and spindle pole associated protein 1 | other |  |  | 1,15E-08 | -1,627 |  |  |
| 227109_at | CYP2R1 | cytochrome P450, family 2, subfamily R, polypeptide 1 | enzyme | 1,83E-14 | -1,909 | 1,83E-14 | -1,815 | 1,83E-14 | -1,914 |
| 227174_at | WDR72 | WD repeat domain 72 | other |  |  | 6,13E-09 | -1,580 |  |  |
| 227180_at | ELOVL7 | ELOVL family member 7, elongation of long chain fatty acids (yeast) | other |  |  |  |  | 2,41E-07 | -1,410 |
| 227186_s_at | MRPL41 | mitochondrial ribosomal protein L41 | other |  |  | 5,62E-10 | -1,662 |  |  |
| 227194_at | FAM3B | family with sequence similarity 3, member B | cytokine | 1,83E-14 | -1,952 | 1,83E-14 | -2,540 | 1,83E-14 | -2,244 |
| 227196_at | RHPN2 | rhophilin, Rho GTPase binding protein 2 | other | 1,92E-09 | -1,473 | 1,92E-09 | -1,562 |  |  |
| 227197_at | SGEF | Src homology 3 domain-containing guanine nucleotide exchange factor | other | 2,61E-11 | -1,655 | 2,61E-11 | -1,561 | 2,61E-11 | -1,448 |
| 227211_at | PHF19 | PHD finger protein 19 | other |  |  | 2,49E-10 | -2,181 |  |  |
| 227212_s_at | PHF19 | PHD finger protein 19 | other | 3,31E-12 | -1,457 | 3,31E-12 | -2,100 | 3,31E-12 | -1,776 |
| 227234_at | LOC100132815 | hypothetical protein LOC100132815 | other | 9,96E-11 | -1,727 | 9,96E-11 | -2,091 | 9,96E-11 | -1,775 |
| 227260_at | ANKRD10 | ankyrin repeat domain 10 | transcription regulator |  |  | 3,44E-14 | -1,568 | 3,44E-14 | -1,843 |
| 227261_at | KLF12 | Kruppel-like factor 12 | transcription regulator | 1,85E-09 | -1,573 |  |  |  |  |
| 227286_at | INO80E | INO80 complex subunit E | other |  |  | 3,78E-11 | -2,271 |  |  |
| 227294_at | ZNF689 | zinc finger protein 689 | transcription regulator |  |  | 1,07E-07 | -1,898 |  |  |
| 227295_at | IKIP | IKK interacting protein | other | 2,11E-10 | -1,520 | 2,11E-10 | -2,141 | 2,11E-10 | -1,658 |
| 227304_at | SMCR8 | Smith-Magenis syndrome chromosome region, candidate 8 | other |  |  | 4,64E-11 | -1,957 |  |  |
| 227305_s_at | SMCR8 | Smith-Magenis syndrome chromosome region, candidate 8 | other |  |  | 9,77E-10 | -1,644 |  |  |
| 227314_at | ITGA2 | integrin, alpha 2 (CD49B, alpha 2 subunit of VLA-2 receptor) | other |  |  |  |  | 3,20E-12 | 1,324 |
| 227335_at | DIDO1 | death inducer-obliterator 1 | other | 6,32E-13 | -1,915 | 6,32E-13 | -1,819 |  |  |
| 227345_at | TNFRSF10D | tumor necrosis factor receptor superfamily, member 10d, decoy with truncated death domain | transmembrane receptor | 2,39E-10 | -1,560 | 2,39E-10 | -1,748 |  |  |
| 227363_s_at | COX4NB | COX4 neighbor | other |  |  | 2,82E-09 | -1,618 |  |  |
| 227369_at | SERBP1 | SERPINE1 mRNA binding protein 1 | other |  |  | 2,04E-08 | -1,627 |  |  |
| 227370_at | FAM171B | family with sequence similarity 171, member B | other | 8,70E-09 | -1,372 | 8,70E-09 | -1,655 |  |  |
| 227373_at | ATXN1L | ataxin 1-like | other |  |  | 6,96E-11 | -1,670 | 6,96E-11 | -1,316 |
| 227377_at | IGF2BP1 | insulin-like growth factor 2 mRNA binding protein 1 | other |  |  | 7,55E-14 | -1,925 |  |  |
| 227399_at | VGLL3 | vestigial like 3 (Drosophila) | other | 2,56E-12 | -1,642 | 2,56E-12 | -1,523 |  |  |
| 227407_at | TAPT1 | transmembrane anterior posterior transformation 1 | G-protein coupled receptor |  |  | 2,30E-08 | -1,497 |  |  |
| 227416_s_at | ZCRB1 | zinc finger CCHC-type and RNA binding motif 1 | other |  |  | 2,36E-13 | -1,899 |  |  |
| 227420_at | TNFAIP8L1 | tumor necrosis factor, alpha-induced protein 8-like 1 | other | 5,20E-12 | -1,577 | 5,20E-12 | -2,361 |  |  |
| 227436_at | OTUD7B | OTU domain containing 7B | peptidase |  |  | 3,02E-08 | -2,077 | 3,02E-08 | -1,642 |
| 227449_at | EPHA4 | EPH receptor A4 | kinase | 1,83E-14 | -2,026 | 1,83E-14 | -1,931 | 1,83E-14 | -1,959 |
| 227473_at | CTTN | cortactin | other |  |  | 7,36E-11 | -1,888 |  |  |
| 227474_at | LOC654433 | hypothetical LOC654433 | other |  |  | 3,57E-10 | -1,630 | 3,57E-10 | -1,290 |
| 227510_x_at | MALAT1 | metastasis associated lung adenocarcinoma transcript 1 (non-protein coding) | other | 1,83E-14 | -1,276 | 1,83E-14 | -2,540 | 1,83E-14 | -4,773 |
| 227517_s_at | GAS5 | growth arrest-specific 5 (non-protein coding) | other |  |  | 1,55E-11 | -1,515 |  |  |
| 227521_at | FBXO33 | F-box protein 33 | other |  |  | 2,27E-07 | -2,039 |  |  |
| 227525_at | GLCCI1 | glucocorticoid induced transcript 1 | other | 5,19E-09 | -1,502 | 5,19E-09 | -1,494 | 5,19E-09 | -1,321 |
| 227558_at | CBX4 | chromobox homolog 4 (Pc class homolog, Drosophila) | transcription regulator |  |  | 1,03E-10 | -1,744 |  |  |
| 227560_at | SFXN2 | sideroflexin 2 | transporter | 1,06E-07 | -1,516 |  |  |  |  |
| 227609_at | EPSTI1 | epithelial stromal interaction 1 (breast) | other | 6,26E-14 | -1,885 | 6,26E-14 | -2,115 | 6,26E-14 | -1,918 |
| 227614_at | HKDC1 | hexokinase domain containing 1 | kinase |  |  | 3,17E-11 | -1,512 |  |  |
| 227628_at | GPX8 | glutathione peroxidase 8 (putative) | enzyme | 4,41E-10 | -1,571 | 4,41E-10 | -1,607 | 4,41E-10 | -1,586 |
| 227636_at | THAP5 | THAP domain containing 5 | other |  |  | 1,71E-08 | -1,803 |  |  |
| 227640_s_at | RP9 | retinitis pigmentosa 9 (autosomal dominant) | other |  |  | 3,48E-11 | -2,278 |  |  |
| 227647_at | KCNE3 | potassium voltage-gated channel, Isk-related family, member 3 | ion channel |  |  | 2,11E-09 | -1,914 |  |  |
| 227669_at | BRP44 | brain protein 44 | other | 1,03E-08 | -1,471 | 1,03E-08 | -1,884 |  |  |
| 227678_at | XRCC6BP1 | XRCC6 binding protein 1 | kinase | 8,21E-08 | -1,430 | 8,21E-08 | -1,702 |  |  |
| 227700_x_at | ATAD3A | ATPase family, AAA domain containing 3A | other |  |  | 1,11E-08 | -2,446 |  |  |
| 227722_at | RPS23 | ribosomal protein S23 | translation regulator |  |  | 3,89E-09 | -1,959 | 3,89E-09 | -1,758 |
| 227726_at | RNF166 | ring finger protein 166 | other | 3,12E-09 | -1,614 | 3,12E-09 | -1,992 | 3,12E-09 | -1,686 |
| 227740_at | UHMK1 | U2AF homology motif (UHM) kinase 1 | kinase |  |  | 7,91E-13 | 1,637 | 7,91E-13 | 2,246 |
| 227741_at | PTPLB | protein tyrosine phosphatase-like (proline instead of catalytic arginine), member b | phosphatase |  |  | 1,50E-13 | 1,542 | 1,50E-13 | 1,735 |
| 227753_at | TMEM139 | transmembrane protein 139 | other |  |  | 1,63E-09 | -2,066 |  |  |
| 227784_s_at | COG1 | component of oligomeric golgi complex 1 | transporter |  |  | 6,02E-08 | -1,804 |  |  |
| 227786_at | MED30 | mediator complex subunit 30 | transcription regulator |  |  | 8,52E-10 | -1,910 |  |  |
| 227792_at | ITPRIPL2 | inositol 1,4,5-triphosphate receptor interacting protein-like 2 | other |  |  |  |  | 3,72E-13 | 1,477 |
| 227812_at | TNFRSF19 | tumor necrosis factor receptor superfamily, member 19 | transmembrane receptor | 5,13E-09 | -1,587 |  |  |  |  |
| 227833_s_at | MBD6 | methyl-CpG binding domain protein 6 | other |  |  | 8,69E-10 | -1,792 | 8,69E-10 | -1,448 |
| 227846_at | GPR176 | G protein-coupled receptor 176 | G-protein coupled receptor | 1,40E-07 | -1,643 |  |  |  |  |
| 227861_at | TMEM161B | transmembrane protein 161B | other |  |  | 1,70E-09 | -1,504 |  |  |
| 227871_at | CHM | choroideremia (Rab escort protein 1) | enzyme | 3,87E-10 | -1,381 | 3,87E-10 | -1,733 | 3,87E-10 | -1,460 |
| 227878_s_at | ALKBH7 | alkB, alkylation repair homolog 7 (E. coli) | other |  |  | 8,09E-09 | -2,002 |  |  |
| 227896_at | BCCIP | BRCA2 and CDKN1A interacting protein | other |  |  | 1,90E-07 | -1,744 |  |  |
| 227911_at | ARHGAP28 | Rho GTPase activating protein 28 | other | 3,58E-12 | -1,846 | 3,58E-12 | -1,630 | 3,58E-12 | -1,666 |
| 227935_s_at | PCGF5 | polycomb group ring finger 5 | other | 2,49E-11 | -1,387 | 2,49E-11 | -1,899 | 2,49E-11 | -1,549 |
| 227936_at | TMEM68 | transmembrane protein 68 | other |  |  | 3,76E-11 | -2,028 |  |  |
| 227947_at | PHACTR2 | phosphatase and actin regulator 2 | other | 1,83E-14 | -1,456 | 1,83E-14 | -1,597 |  |  |
| 227960_s_at | FAHD1 | fumarylacetoacetate hydrolase domain containing 1 | enzyme | 8,44E-11 | -1,321 | 8,44E-11 | -1,644 | 8,44E-11 | -1,446 |
| 227990_at | SLU7 | SLU7 splicing factor homolog (S. cerevisiae) | enzyme | 1,83E-14 | -1,823 | 1,83E-14 | -2,029 |  |  |
| 227993_at | METAP2 (includes EG:10988) | methionyl aminopeptidase 2 | peptidase |  |  | 9,28E-09 | -1,691 | 9,28E-09 | -1,379 |
| 227998_at | S100A16 | S100 calcium binding protein A16 | other |  |  | 9,43E-08 | -1,681 |  |  |
| 228009_x_at | ZNRD1 | zinc ribbon domain containing 1 | transcription regulator |  |  | 1,01E-09 | -1,728 |  |  |
| 228033_at | E2F7 | E2F transcription factor 7 | transcription regulator | 3,28E-09 | -1,448 |  |  |  |  |
| 228038_at | SOX2 | SRY (sex determining region Y)-box 2 | transcription regulator | 1,83E-14 | -2,396 | 1,83E-14 | -2,107 | 1,83E-14 | -2,731 |
| 228053_s_at | TOMM5 (includes EG:401505) | translocase of outer mitochondrial membrane 5 homolog (yeast) | other |  |  | 2,67E-08 | -1,629 |  |  |
| 228092_at | CREM | cAMP responsive element modulator | transcription regulator |  |  | 2,00E-07 | -1,786 |  |  |
| 228106_at | C4ORF30 | chromosome 4 open reading frame 30 | other | 8,89E-13 | -1,378 | 8,89E-13 | -1,687 | 8,89E-13 | -1,722 |
| 228123_s_at | ABHD12 | abhydrolase domain containing 12 | other | 1,83E-14 | -1,611 | 1,83E-14 | -2,247 | 1,83E-14 | -1,622 |
| 228155_at | C10ORF58 | chromosome 10 open reading frame 58 | other |  |  | 1,85E-07 | -1,464 |  |  |
| 228183_s_at | RPAIN | RPA interacting protein | other |  |  | 1,07E-10 | -1,766 |  |  |
| 228196_s_at | LARP5 | La ribonucleoprotein domain family, member 5 | other |  |  | 2,55E-09 | -1,861 | 2,55E-09 | -2,286 |
| 228221_at | SLC44A3 | solute carrier family 44, member 3 | other | 3,95E-11 | -1,626 | 3,95E-11 | -1,828 | 3,95E-11 | -1,461 |
| 228222_at | PPP1CB | protein phosphatase 1, catalytic subunit, beta isoform | phosphatase |  |  | 1,83E-14 | -1,786 | 1,83E-14 | -1,324 |
| 228230_at | PRIC285 (includes EG:85441) | peroxisomal proliferator-activated receptor A interacting complex 285 | transcription regulator | 3,70E-07 | -1,937 |  |  |  |  |
| 228283_at | CMC1 | COX assembly mitochondrial protein homolog (S. cerevisiae) | other | 9,00E-09 | -1,441 | 9,00E-09 | -1,511 |  |  |
| 228332_s_at | C11ORF31 | chromosome 11 open reading frame 31 | other | 2,88E-08 | -1,364 | 2,88E-08 | -1,628 |  |  |
| 228353_x_at | UBASH3B | ubiquitin associated and SH3 domain containing, B | enzyme |  |  |  |  | 2,86E-06 | 1,905 |
| 228355_s_at | NDUFAF2 | NADH dehydrogenase (ubiquinone) 1 alpha subcomplex, assembly factor 2 | other |  |  | 1,30E-10 | -1,700 |  |  |
| 228357_at | UNK | unkempt homolog (Drosophila) | transporter | 3,80E-10 | -1,526 | 3,80E-10 | -1,717 | 3,80E-10 | -1,571 |
| 228370_at | SNRPN | small nuclear ribonucleoprotein polypeptide N | other | 9,87E-11 | -1,394 | 9,87E-11 | -1,937 | 9,87E-11 | -1,649 |
| 228381_at | ATF7IP2 | activating transcription factor 7 interacting protein 2 | other | 1,07E-09 | -1,991 | 1,07E-09 | -1,904 | 1,07E-09 | -1,945 |
| 228482_at | CDRT4 | CMT1A duplicated region transcript 4 | other | 4,59E-08 | -1,714 |  |  |  |  |
| 228523_at | NANOS1 | nanos homolog 1 (Drosophila) | other | 7,23E-08 | -1,571 |  |  | 7,23E-08 | -1,458 |
| 228544_s_at | CSRP2BP | CSRP2 binding protein | other | 1,78E-11 | -1,459 | 1,78E-11 | -2,107 |  |  |
| 228559_at | CENPN | centromere protein N | other | 3,91E-12 | -1,426 | 3,91E-12 | -1,815 | 3,91E-12 | -1,309 |
| 228582_x_at | MALAT1 | metastasis associated lung adenocarcinoma transcript 1 (non-protein coding) | other | 1,83E-14 | -1,315 | 1,83E-14 | -2,457 | 1,83E-14 | -3,017 |
| 228597_at | C21ORF45 | chromosome 21 open reading frame 45 | other | 1,41E-11 | -1,487 | 1,41E-11 | -1,861 | 1,41E-11 | -1,409 |
| 228606_at | TCTEX1D2 | Tctex1 domain containing 2 | other | 3,34E-12 | -1,527 | 3,34E-12 | -1,653 | 3,34E-12 | -1,817 |
| 228654_at | SPIN4 | spindlin family, member 4 | other | 6,48E-11 | -1,610 | 6,48E-11 | -1,510 | 6,48E-11 | -1,356 |
| 228680_at | KIF3A | kinesin family member 3A | other |  |  | 1,01E-12 | -2,040 |  |  |
| 228690_s_at | NDUFA11 | NADH dehydrogenase (ubiquinone) 1 alpha subcomplex, 11, 14.7kDa | enzyme |  |  | 2,82E-11 | -1,771 | 2,82E-11 | -1,329 |
| 228729_at | CCNB1 | cyclin B1 | other |  |  | 3,11E-09 | -1,428 |  |  |
| 228730_s_at | SCRN2 | secernin 2 | other |  |  | 2,59E-09 | -1,825 |  |  |
| 228751_at | CLK4 | CDC-like kinase 4 | kinase |  |  |  |  | 1,79E-07 | -1,487 |
| 228777_at | KBTBD3 | kelch repeat and BTB (POZ) domain containing 3 | other |  |  |  |  | 8,55E-07 | -1,739 |
| 228805_at | C5ORF25 | chromosome 5 open reading frame 25 | other |  |  | 3,58E-09 | -1,620 |  |  |
| 228822_s_at | USP16 | ubiquitin specific peptidase 16 | peptidase |  |  | 1,11E-10 | -1,885 |  |  |
| 228834_at | TOB1 | transducer of ERBB2, 1 | transcription regulator |  |  | 1,89E-12 | -1,820 | 1,89E-12 | -1,644 |
| 228841_at | LYRM7 | Lyrm7 homolog (mouse) | other |  |  | 1,38E-07 | -1,536 |  |  |
| 228851_s_at | ENSA | endosulfine alpha | transporter |  |  | 5,60E-09 | -1,774 |  |  |
| 228853_at | STYX | serine/threonine/tyrosine interacting protein | phosphatase | 1,83E-14 | -1,956 | 1,83E-14 | -2,140 |  |  |
| 228855_at | NUDT7 | nudix (nucleoside diphosphate linked moiety X)-type motif 7 | enzyme |  |  | 4,81E-09 | -1,927 |  |  |
| 228931_at | COQ4 | coenzyme Q4 homolog (S. cerevisiae) | other |  |  | 4,29E-09 | -1,888 |  |  |
| 228941_at | ALG10B | asparagine-linked glycosylation 10, alpha-1,2-glucosyltransferase homolog B (yeast) | transporter |  |  |  |  | 1,39E-07 | -1,588 |
| 228966_at | PANK2 | pantothenate kinase 2 | kinase |  |  |  |  | 3,88E-10 | 2,966 |
| 228970_at | ZBTB8OS | zinc finger and BTB domain containing 8 opposite strand | other |  |  | 1,30E-07 | -1,556 |  |  |
| 228980_at | RFFL | ring finger and FYVE-like domain containing 1 | enzyme |  |  | 3,56E-08 | -1,540 |  |  |
| 228988_at | ZNF711 | zinc finger protein 711 | other |  |  | 5,57E-10 | -1,664 |  |  |
| 229010_at | CBL | Cas-Br-M (murine) ecotropic retroviral transforming sequence | transcription regulator |  |  |  |  | 2,92E-08 | 1,879 |
| 229025_s_at | IMMP1L | IMP1 inner mitochondrial membrane peptidase-like (S. cerevisiae) | other |  |  | 2,54E-08 | -1,661 |  |  |
| 229058_at | ANKRD16 | ankyrin repeat domain 16 | other |  |  | 4,96E-09 | -2,036 |  |  |
| 229095_s_at | LOC440895 | LIM and senescent cell antigen-like domains 3-like | other |  |  | 6,48E-09 | -1,616 |  |  |
| 229097_at | DIAPH3 | diaphanous homolog 3 (Drosophila) | enzyme |  |  | 9,16E-13 | -1,828 |  |  |
| 229099_at | C11ORF83 | chromosome 11 open reading frame 83 | other |  |  | 1,68E-11 | -1,974 |  |  |
| 229126_at | TMEM19 | transmembrane protein 19 | other | 3,87E-10 | -1,484 | 3,87E-10 | -1,777 |  |  |
| 229145_at | C10ORF104 | chromosome 10 open reading frame 104 | other |  |  | 6,88E-08 | -1,615 |  |  |
| 229174_at | C3ORF38 | chromosome 3 open reading frame 38 | peptidase | 1,57E-10 | -1,463 | 1,57E-10 | -1,619 | 1,57E-10 | -1,378 |
| 229231_at | LRRC37B | leucine rich repeat containing 37B | other | 4,97E-08 | -2,133 |  |  |  |  |
| 229317_at | KPNA5 | karyopherin alpha 5 (importin alpha 6) | other |  |  | 1,04E-07 | -1,726 |  |  |
| 229332_at | HPDL | 4-hydroxyphenylpyruvate dioxygenase-like | other |  |  | 4,02E-13 | -2,008 |  |  |
| 229349_at | LIN28B | lin-28 homolog B (C. elegans) | other |  |  | 4,87E-14 | -1,602 |  |  |
| 229358_at | IHH | Indian hedgehog homolog (Drosophila) | enzyme |  |  | 1,83E-14 | -2,593 |  |  |
| 229374_at | EPHA4 | EPH receptor A4 | kinase |  |  |  |  | 6,29E-08 | -1,548 |
| 229377_at | GRTP1 | growth hormone regulated TBC protein 1 | other | 1,67E-06 | -1,460 |  |  |  |  |
| 229420_at | HCG 16001 | similar to ribosomal protein L23A | other |  |  | 1,83E-14 | 2,146 | 1,83E-14 | 1,876 |
| 229518_at | FAM46B | family with sequence similarity 46, member B | other | 2,03E-06 | -1,823 |  |  |  |  |
| 229526_at | AQP11 | aquaporin 11 | transporter | 5,30E-12 | -1,576 | 5,30E-12 | -1,678 | 5,30E-12 | -1,870 |
| 229551_x_at | ZNF367 | zinc finger protein 367 | transcription regulator | 3,48E-12 | -1,715 | 3,48E-12 | -1,730 | 3,48E-12 | -1,482 |
| 229574_at | TRA2A | transformer-2 alpha | other |  |  |  |  | 1,69E-07 | -1,478 |
| 229632_s_at | INTS10 | integrator complex subunit 10 | other |  |  | 1,31E-08 | -1,789 |  |  |
| 229666_s_at | CSTF3 | cleavage stimulation factor, 3' pre-RNA, subunit 3, 77kDa | other |  |  | 1,45E-07 | -1,666 |  |  |
| 229667_s_at | HOXB8 | homeobox B8 | transcription regulator | 1,83E-14 | 1,703 |  |  |  |  |
| 229704_at | PDS5B | PDS5, regulator of cohesion maintenance, homolog B (S. cerevisiae) | other |  |  | 2,75E-12 | -2,024 | 2,75E-12 | -1,661 |
| 229736_at | TMEM86B | transmembrane protein 86B | other | 1,29E-08 | -2,139 |  |  | 1,29E-08 | -4,207 |
| 229742_at | C15ORF61 | chromosome 15 open reading frame 61 | other |  |  | 4,38E-08 | -1,629 |  |  |
| 229970_at | KBTBD7 | kelch repeat and BTB (POZ) domain containing 7 | other | 4,83E-09 | -1,994 | 4,83E-09 | -2,108 | 4,83E-09 | -2,532 |
| 229982_at | QSER1 | glutamine and serine rich 1 | other | 2,99E-13 | 1,540 |  |  | 2,99E-13 | 1,493 |
| 229983_at | TIGD2 (includes EG:166815) | tigger transposable element derived 2 | other | 4,75E-09 | -1,543 | 4,75E-09 | -1,524 | 4,75E-09 | -1,414 |
| 230005_at | SVIP (includes EG:258010) | small VCP/p97-interacting protein | other | 5,06E-10 | -1,874 | 5,06E-10 | -2,036 | 5,06E-10 | -1,761 |
| 230006_s_at | SVIP (includes EG:258010) | small VCP/p97-interacting protein | other | 1,10E-07 | -1,393 |  |  |  |  |
| 230051_at | C10ORF47 | chromosome 10 open reading frame 47 | other |  |  | 1,23E-08 | -1,808 |  |  |
| 230129_at | PSTK | phosphoseryl-tRNA kinase | kinase |  |  |  |  | 1,51E-08 | -1,788 |
| 230165_at | SGOL2 | shugoshin-like 2 (S. pombe) | other | 2,23E-11 | -1,484 | 2,23E-11 | -1,623 | 2,23E-11 | -1,298 |
| 230257_s_at | TSEN15 | tRNA splicing endonuclease 15 homolog (S. cerevisiae) | other |  |  | 7,15E-09 | -1,896 |  |  |
| 230263_s_at | DOCK5 | dedicator of cytokinesis 5 | other | 4,40E-08 | -1,501 |  |  |  |  |
| 230264_s_at | AP1S2 | adaptor-related protein complex 1, sigma 2 subunit | transporter |  |  | 4,86E-08 | -1,732 |  |  |
| 230323_s_at | TMEM45B | transmembrane protein 45B | other | 3,83E-09 | -1,338 | 3,83E-09 | -1,587 |  |  |
| 230326_s_at | C11ORF73 | chromosome 11 open reading frame 73 | other |  |  | 1,60E-08 | -1,584 |  |  |
| 230352_at | PRPS2 | phosphoribosyl pyrophosphate synthetase 2 | kinase | 1,83E-14 | 1,382 |  |  |  |  |
| 230380_at | THAP2 | THAP domain containing, apoptosis associated protein 2 | other | 2,78E-08 | -1,792 | 2,78E-08 | -1,971 |  |  |
| 230448_at | SLC38A10 | solute carrier family 38, member 10 | other |  |  | 9,50E-11 | -2,106 |  |  |
| 230508_at | DKK3 | dickkopf homolog 3 (Xenopus laevis) | other |  |  | 2,27E-08 | -1,861 | 2,27E-08 | -1,534 |
| 230521_at | C9ORF100 | chromosome 9 open reading frame 100 | other | 2,00E-09 | -2,260 | 2,00E-09 | -2,276 | 2,00E-09 | -1,904 |
| 230588_s_at | LOC285074 | hypothetical protein LOC285074 | other |  |  | 6,89E-08 | -1,663 |  |  |
| 230621_at | IAH1 | isoamyl acetate-hydrolyzing esterase 1 homolog (S. cerevisiae) | other |  |  | 3,47E-13 | -1,793 | 3,47E-13 | -1,480 |
| 230656_s_at | CIRH1A | cirrhosis, autosomal recessive 1A (cirhin) | other |  |  | 4,99E-08 | -1,523 |  |  |
| 230681_at | TBRG1 (includes EG:84897) | transforming growth factor beta regulator 1 | other | 1,95E-07 | 2,690 |  |  |  |  |
| 230716_at | LOC285733 | hypothetical LOC285733 | other | 3,93E-12 | -1,591 | 3,93E-12 | -1,804 | 3,93E-12 | -1,864 |
| 230788_at | GCNT2 | glucosaminyl (N-acetyl) transferase 2, I-branching enzyme (I blood group) | enzyme | 9,59E-09 | -1,638 |  |  | 9,59E-09 | -1,390 |
| 230863_at | LRP2 | low density lipoprotein-related protein 2 | transporter | 3,18E-12 | -2,130 | 3,18E-12 | -1,703 | 3,18E-12 | -2,124 |
| 230871_at | DHX30 | DEAH (Asp-Glu-Ala-His) box polypeptide 30 | enzyme |  |  | 1,81E-07 | -1,858 |  |  |
| 230951_at | EPB41L5 | erythrocyte membrane protein band 4.1 like 5 | other |  |  | 6,89E-08 | -1,711 |  |  |
| 230954_at | C20ORF112 | chromosome 20 open reading frame 112 | other | 1,83E-08 | -1,700 |  |  | 1,83E-08 | -1,982 |
| 230960_at | PUNC | putative neuronal cell adhesion molecule | other | 3,75E-10 | -1,827 | 3,75E-10 | -2,091 | 3,75E-10 | -2,131 |
| 231003_at | SLC35B3 | solute carrier family 35, member B3 | other | 2,07E-11 | -2,032 | 2,07E-11 | -1,988 | 2,07E-11 | -1,618 |
| 231008_at | UNC5CL | unc-5 homolog C (C. elegans)-like | other | 1,36E-10 | -2,058 | 1,36E-10 | -1,899 | 1,36E-10 | -2,567 |
| 231059_x_at | SCAND1 | SCAN domain containing 1 | transcription regulator |  |  | 2,18E-08 | -1,685 |  |  |
| 231183_s_at | JAG1 | jagged 1 (Alagille syndrome) | growth factor |  |  |  |  | 3,31E-09 | 1,657 |
| 231579_s_at | TIMP2 | TIMP metallopeptidase inhibitor 2 | other |  |  | 7,93E-09 | -1,724 |  |  |
| 231727_s_at | MIF4GD | MIF4G domain containing | other |  |  | 2,03E-08 | -1,877 |  |  |
| 231735_s_at | MALAT1 | metastasis associated lung adenocarcinoma transcript 1 (non-protein coding) | other | 1,83E-14 | -1,347 | 1,83E-14 | -2,257 | 1,83E-14 | -2,732 |
| 231769_at | FBXO6 | F-box protein 6 | enzyme |  |  | 8,98E-10 | -2,026 |  |  |
| 231779_at | IRAK2 | interleukin-1 receptor-associated kinase 2 | kinase |  |  |  |  | 1,85E-13 | 1,799 |
| 231846_at | FOXRED2 | FAD-dependent oxidoreductase domain containing 2 | other |  |  | 3,46E-08 | -2,068 |  |  |
| 231853_at | TUBD1 | tubulin, delta 1 | other | 6,73E-11 | -1,784 | 6,73E-11 | -1,875 | 6,73E-11 | -1,532 |
| 231855_at | KIAA1524 | KIAA1524 | other | 4,88E-12 | -1,608 | 4,88E-12 | -1,730 |  |  |
| 231864_at | ZNF33A | zinc finger protein 33A | transcription regulator | 2,65E-08 | -1,527 | 2,65E-08 | -1,693 |  |  |
| 231866_at | LNPEP | leucyl/cystinyl aminopeptidase | peptidase |  |  |  |  | 4,17E-08 | 1,406 |
| 231967_at | PHF20L1 | PHD finger protein 20-like 1 | other |  |  | 4,37E-08 | -1,965 | 4,37E-08 | -1,612 |
| 231973_s_at | ANAPC1 | anaphase promoting complex subunit 1 | other |  |  | 6,17E-09 | -1,790 |  |  |
| 231982_at | LOC284422 | similar to HSPC323 | other |  |  | 1,83E-14 | -2,009 | 1,83E-14 | -1,699 |
| 231984_at | MTAP (includes EG:4507) | methylthioadenosine phosphorylase | enzyme |  |  | 1,17E-07 | -2,403 |  |  |
| 232032_x_at | SDF4 | stromal cell derived factor 4 | other |  |  | 1,10E-07 | -1,711 |  |  |
| 232053_x_at | RHBDD2 | rhomboid domain containing 2 | other | 1,88E-10 | -1,698 | 1,88E-10 | -2,689 | 1,88E-10 | -1,882 |
| 232060_at | ROR1 | receptor tyrosine kinase-like orphan receptor 1 | kinase | 1,66E-12 | -2,018 | 1,66E-12 | -1,592 | 1,66E-12 | -1,606 |
| 232168_x_at | MACF1 | microtubule-actin crosslinking factor 1 | other | 1,34E-08 | -2,458 |  |  |  |  |
| 232181_at | LOC153346 | hypothetical protein LOC153346 | other |  |  |  |  | 1,83E-14 | 1,952 |
| 232209_x_at | HM13 | histocompatibility (minor) 13 | peptidase |  |  | 3,64E-09 | -2,027 |  |  |
| 232244_at | KIAA1161 | KIAA1161 | other | 4,76E-09 | -1,641 |  |  | 4,76E-09 | -1,718 |
| 232278_s_at | DEPDC1 | DEP domain containing 1 | other | 7,89E-11 | -1,641 | 7,89E-11 | -1,948 |  |  |
| 232322_x_at | STARD10 | StAR-related lipid transfer (START) domain containing 10 | other |  |  | 2,43E-13 | -2,032 | 2,43E-13 | -1,420 |
| 232370_at | LOC254057 | hypothetical protein LOC254057 | other |  |  | 4,82E-09 | -2,398 |  |  |
| 232383_at | TFEC | transcription factor EC | transcription regulator | 2,16E-13 | -2,220 | 2,16E-13 | -2,439 | 2,16E-13 | -2,883 |
| 232395_x_at | AGBL3 | ATP/GTP binding protein-like 3 | other |  |  |  |  | 3,27E-07 | -1,610 |
| 232397_at | HECW2 | HECT, C2 and WW domain containing E3 ubiquitin protein ligase 2 | enzyme | 2,49E-09 | -1,499 | 2,49E-09 | -1,561 | 2,49E-09 | -1,447 |
| 232432_s_at | SLC30A5 | solute carrier family 30 (zinc transporter), member 5 | transporter |  |  | 1,79E-08 | -1,533 |  |  |
| 232510_s_at | BBS1 | Bardet-Biedl syndrome 1 | other |  |  | 6,24E-08 | -1,612 |  |  |
| 232591_s_at | TMEM30A | transmembrane protein 30A | other |  |  | 9,58E-08 | -1,554 |  |  |
| 232611_at | GOLGA2L1 | golgi autoantigen, golgin subfamily a, 2-like 1 | other |  |  | 1,28E-08 | -2,085 | 1,28E-08 | -2,097 |
| 232652_x_at | SCAND1 | SCAN domain containing 1 | transcription regulator | 3,44E-14 | -1,504 | 3,44E-14 | -2,033 | 3,44E-14 | -1,571 |
| 232707_at | ISX | intestine-specific homeobox | other | 3,44E-14 | -2,483 | 3,44E-14 | -2,471 | 3,44E-14 | -2,794 |
| 232909_s_at | BPTF | bromodomain PHD finger transcription factor | transcription regulator | 7,33E-12 | -1,588 | 7,33E-12 | -1,806 | 7,33E-12 | -1,419 |
| 233049_x_at | STUB1 | STIP1 homology and U-box containing protein 1 | enzyme |  |  | 9,43E-08 | -1,607 |  |  |
| 233093_s_at | BIRC6 | baculoviral IAP repeat-containing 6 | enzyme |  |  | 1,55E-08 | -1,510 |  |  |
| 233168_s_at | RP3-402G11.5 | selenoprotein O | enzyme |  |  | 1,75E-08 | -1,908 |  |  |
| 233173_x_at | GTF3C5 | general transcription factor IIIC, polypeptide 5, 63kDa | transcription regulator |  |  | 6,98E-09 | -1,846 |  |  |
| 233320_at | TCAM1 | testicular cell adhesion molecule 1 homolog (mouse) | other |  |  | 2,69E-09 | -1,766 |  |  |
| 233496_s_at | CFL2 | cofilin 2 (muscle) | other | 4,07E-09 | -1,442 | 4,07E-09 | -1,730 | 4,07E-09 | -1,634 |
| 233532_x_at | IFT52 | intraflagellar transport 52 homolog (Chlamydomonas) | other |  |  | 2,06E-11 | -2,493 |  |  |
| 233557_s_at | MON1B | MON1 homolog B (yeast) | other |  |  | 6,35E-08 | -1,876 |  |  |
| 233625_x_at | CPSF3L | cleavage and polyadenylation specific factor 3-like | other |  |  | 3,85E-08 | -1,923 |  |  |
| 233656_s_at | VPS54 | vacuolar protein sorting 54 homolog (S. cerevisiae) | other |  |  | 4,06E-09 | -1,535 |  |  |
| 233898_s_at | FGFR1OP2 | FGFR1 oncogene partner 2 | other | 1,01E-13 | -1,653 | 1,01E-13 | -1,750 | 1,01E-13 | -1,376 |
| 233903_s_at | SGEF | Src homology 3 domain-containing guanine nucleotide exchange factor | other | 1,20E-12 | -1,700 | 1,20E-12 | -1,659 | 1,20E-12 | -2,327 |
| 233929_x_at | WASH1 | WAS protein family homolog 1 | other |  |  | 2,41E-07 | -1,756 |  |  |
| 233955_x_at | CXXC5 | CXXC finger 5 | other |  |  | 1,15E-09 | -1,838 |  |  |
| 233970_s_at | TRMT6 | tRNA methyltransferase 6 homolog (S. cerevisiae) | other | 2,41E-09 | -1,395 | 2,41E-09 | -1,562 | 2,41E-09 | -1,534 |
| 234294_x_at | GATAD2A | GATA zinc finger domain containing 2A | transcription regulator |  |  | 7,32E-12 | -2,151 |  |  |
| 234295_at | DBR1 | debranching enzyme homolog 1 (S. cerevisiae) | enzyme |  |  | 6,47E-09 | -1,622 |  |  |
| 234312_s_at | ACSS2 | acyl-CoA synthetase short-chain family member 2 | enzyme |  |  | 6,48E-08 | -1,888 |  |  |
| 234339_s_at | GLTSCR2 | glioma tumor suppressor candidate region gene 2 | other | 5,45E-11 | -1,567 | 5,45E-11 | -2,279 | 5,45E-11 | -1,899 |
| 234405_s_at | PHAX | phosphorylated adaptor for RNA export | other | 3,81E-12 | -1,641 | 3,81E-12 | -1,763 |  |  |
| 234660_s_at | DIS3 | DIS3 mitotic control homolog (S. cerevisiae) | enzyme | 7,13E-08 | -1,395 |  |  |  |  |
| 234697_x_at | C3ORF31 | chromosome 3 open reading frame 31 | other | 3,38E-13 | -2,334 | 3,38E-13 | -2,665 | 3,38E-13 | -2,458 |
| 234942_s_at | DNTTIP1 | deoxynucleotidyltransferase, terminal, interacting protein 1 | ligand-dependent nuclear receptor |  |  | 3,43E-10 | -1,681 |  |  |
| 234947_s_at | C10ORF84 | chromosome 10 open reading frame 84 | other |  |  | 3,09E-10 | -2,051 |  |  |
| 234981_x_at | CMBL | carboxymethylenebutenolidase homolog (Pseudomonas) | enzyme |  |  | 2,06E-13 | -1,886 | 2,06E-13 | -1,844 |
| 234994_at | TMEM200A | transmembrane protein 200A | other |  |  | 1,24E-07 | -1,530 |  |  |
| 234995_at | CCDC52 | coiled-coil domain containing 52 | other |  |  | 2,70E-08 | -1,807 |  |  |
| 235003_at | UHMK1 | U2AF homology motif (UHM) kinase 1 | kinase |  |  |  |  | 5,90E-12 | 2,188 |
| 235005_at | DIS3L | DIS3 mitotic control homolog (S. cerevisiae)-like | other |  |  | 8,04E-08 | -1,659 |  |  |
| 235006_at | CDKN2AIPNL | CDKN2A interacting protein N-terminal like | other | 9,84E-10 | -1,605 | 9,84E-10 | -1,844 |  |  |
| 235020_at | TAF4B | TAF4b RNA polymerase II, TATA box binding protein (TBP)-associated factor, 105kDa | transcription regulator |  |  | 9,47E-10 | -1,969 |  |  |
| 235033_at | NPEPL1 | aminopeptidase-like 1 | peptidase |  |  | 9,33E-11 | -2,074 |  |  |
| 235093_at | PEX13 | peroxisomal biogenesis factor 13 | transporter | 3,80E-08 | -1,739 |  |  |  |  |
| 235103_at | MAN2A1 | mannosidase, alpha, class 2A, member 1 | enzyme | 2,89E-08 | -1,444 |  |  |  |  |
| 235113_at | PPIL5 | peptidylprolyl isomerase (cyclophilin)-like 5 | enzyme | 1,82E-11 | -1,406 | 1,82E-11 | -1,648 | 1,82E-11 | -1,315 |
| 235125_x_at | FAM73A | family with sequence similarity 73, member A | other |  |  |  |  | 1,74E-07 | -1,401 |
| 235158_at | TMEM209 | transmembrane protein 209 | other | 4,92E-12 | -1,504 | 4,92E-12 | -1,904 | 4,92E-12 | -1,352 |
| 235165_at | PARD6B | par-6 partitioning defective 6 homolog beta (C. elegans) | other | 1,83E-14 | -1,826 | 1,83E-14 | -2,190 | 1,83E-14 | -1,617 |
| 235177_at | FAM119A | family with sequence similarity 119, member A | other |  |  | 1,49E-08 | -1,613 |  |  |
| 235239_at | QSOX2 | quiescin Q6 sulfhydryl oxidase 2 | enzyme |  |  |  |  | 3,43E-08 | -1,943 |
| 235253_at | RAD1 | RAD1 homolog (S. pombe) | enzyme |  |  | 4,97E-08 | -1,839 |  |  |
| 235327_x_at | UBXN2A | UBX domain protein 2A | other |  |  | 4,27E-12 | -1,810 | 4,27E-12 | -1,616 |
| 235346_at | FUNDC1 | FUN14 domain containing 1 | other |  |  | 1,26E-07 | -1,632 |  |  |
| 235387_at | GSTCD | glutathione S-transferase, C-terminal domain containing | enzyme |  |  | 5,23E-10 | -2,030 |  |  |
| 235409_at | MGA (includes EG:23269) | MAX gene associated | transcription regulator |  |  | 1,83E-14 | 2,361 | 1,83E-14 | 3,116 |
| 235412_at | ARHGEF7 | Rho guanine nucleotide exchange factor (GEF) 7 | other |  |  |  |  | 2,39E-10 | -4,917 |
| 235432_at | NPHP3 | nephronophthisis 3 (adolescent) | other |  |  |  |  | 4,83E-07 | -1,435 |
| 235509_at | C8ORF38 | chromosome 8 open reading frame 38 | other | 2,36E-08 | -1,324 | 2,36E-08 | -1,580 |  |  |
| 235521_at | HOXA3 (includes EG:3200) | homeobox A3 | transcription regulator | 1,26E-13 | -1,705 | 1,26E-13 | -1,446 | 1,26E-13 | -1,677 |
| 235552_at | KIAA1627 | KIAA1627 protein | other | 6,26E-14 | -1,888 | 6,26E-14 | -2,207 | 6,26E-14 | -1,931 |
| 235572_at | SPC24 | SPC24, NDC80 kinetochore complex component, homolog (S. cerevisiae) | other | 1,83E-14 | -1,613 | 1,83E-14 | -2,613 | 1,83E-14 | -2,047 |
| 235645_at | ESCO1 | establishment of cohesion 1 homolog 1 (S. cerevisiae) | other |  |  |  |  | 5,94E-09 | 2,472 |
| 235749_at | UGCGL2 | UDP-glucose ceramide glucosyltransferase-like 2 | enzyme |  |  | 1,23E-09 | -1,770 |  |  |
| 235792_x_at | PIK3C2A | phosphoinositide-3-kinase, class 2, alpha polypeptide | kinase |  |  | 9,26E-12 | -1,949 |  |  |
| 235812_at | TMEM188 | transmembrane protein 188 | other | 2,92E-09 | -1,355 | 2,92E-09 | -1,495 | 2,92E-09 | -1,608 |
| 235874_at | PRSS35 | protease, serine, 35 | peptidase | 7,36E-10 | -1,400 | 7,36E-10 | -1,535 |  |  |
| 235911_at | LOC440995 | hypothetical gene supported by BC034933; BC068085 | other | 1,83E-14 | -1,780 | 1,83E-14 | -2,281 | 1,83E-14 | -1,700 |
| 236058_at | C1ORF172 | chromosome 1 open reading frame 172 | other | 5,93E-08 | -2,237 | 5,93E-08 | -1,942 |  |  |
| 236118_at | LOC728473 | hypothetical LOC728473 | other |  |  | 1,12E-09 | -1,496 | 1,12E-09 | -1,894 |
| 236518_at | KIAA1984 | KIAA1984 | other |  |  | 1,17E-09 | -1,866 | 1,17E-09 | -2,097 |
| 236565_s_at | LARP6 | La ribonucleoprotein domain family, member 6 | other | 1,43E-12 | -1,615 | 1,43E-12 | -2,151 | 1,43E-12 | -1,743 |
| 236594_at | LLGL1 | lethal giant larvae homolog 1 (Drosophila) | other |  |  | 1,19E-10 | -2,116 |  |  |
| 236620_at | RIF1 | RAP1 interacting factor homolog (yeast) | other |  |  |  |  | 1,14E-13 | 1,810 |
| 236641_at | KIF14 | kinesin family member 14 | other | 1,83E-14 | -1,811 | 1,83E-14 | -1,998 | 1,83E-14 | -1,803 |
| 236646_at | C12ORF59 | chromosome 12 open reading frame 59 | other | 9,19E-12 | -1,960 | 9,19E-12 | -2,071 |  |  |
| 236656_s_at | LOC100130506 | hypothetical protein LOC100130506 | other | 4,30E-11 | -1,609 | 4,30E-11 | -1,943 | 4,30E-11 | -1,705 |
| 236769_at | LOC158402 | hypothetical protein LOC158402 | other | 8,23E-10 | -1,520 | 8,23E-10 | -1,732 |  |  |
| 237159_x_at | AP1S3 (includes EG:130340) | adaptor-related protein complex 1, sigma 3 subunit | transporter | 1,01E-13 | -1,472 | 1,01E-13 | -1,926 | 1,01E-13 | -1,640 |
| 237215_s_at | TFRC | transferrin receptor (p90, CD71) | transporter |  |  | 4,87E-14 | -2,189 |  |  |
| 237515_at | TMEM56 | transmembrane protein 56 | other |  |  | 1,86E-09 | -1,661 | 1,86E-09 | -2,294 |
[truncated: 12,076 more chars]
